# Stereoselective Palladium-Catalyzed C–F Bond Alkenylation of Tetrasubstituted *gem*-Difluoroalkenes via Mizoroki–Heck Reaction

**DOI:** 10.1021/acs.orglett.3c02452

**Published:** 2023-08-16

**Authors:** Yanhui Wang, Gavin Chit Tsui

**Affiliations:** † Department of Chemistry, The Chinese University of Hong Kong, Shatin, New Territories, Hong Kong SAR 999077, China

## Abstract

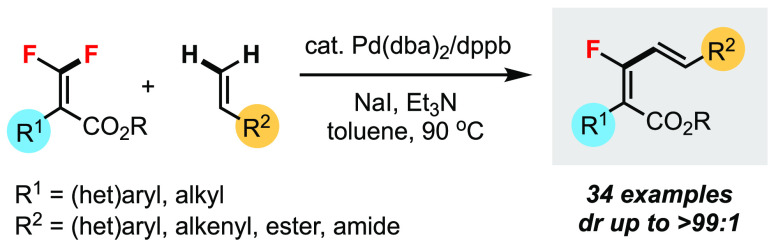

A highly diastereoselective Pd(0)-catalyzed Mizoroki–Heck reaction of *gem*-difluoroalkenes is described. Unlike previously reported C–F bond functionalization with organometallic reagents, this reaction takes place between two different alkenes to achieve a formal C–F and C–H bond cross-coupling via a distinct pathway. Monofluorinated 1,3-diene products can be synthesized with control of the geometry of each alkene and good functional group tolerability.

The Mizoroki–Heck reaction is a palladium-catalyzed cross-coupling between organic halides (usually I, Br, and Cl) and alkenes via oxidative addition/carbopalladation/β-H elimination in the presence of a base. Since its discovery over half a century ago,^1^ this reaction has become state-of-the-art for installing carbon–carbon double bonds with wide applications in industry and academia,^2^ which culminated in the 2010 Nobel Prize in Chemistry.^3^ The key features of the Mizoroki–Heck reaction include high efficiency, ready availability, low-cost alkene feedstocks, and good stereo- and chemoselectivity. Tremendous progress has been made in modern versions of this classic reaction by improving the catalytic systems and broadening the substrate scopes.

In recent years, the Mizoroki–Heck reaction has been utilized to introduce fluorine-containing groups to alkenes.^4^ Organofluorine compounds play a crucial role in pharmaceuticals, agrochemicals, and materials.^5^ The demand for synthesizing fluorinated or perfluoroalkylated alkenes has increased significantly because they are highly versatile building blocks for a range of applications.^6^ For instance, cross-couplings of perfluoroalkyl halides and alkenes or organic halides and perfluoroalkylated alkenes are effective for accessing alkenes with perfluoroalkyl groups (R_f_).^7^ However, examples of Mizoroki–Heck-type reactions of fluorinated alkenes such as *gem*-difluoroalkenes are very limited.^8^

Heitz and co-workers in 1991 first reported a Pd-catalyzed defluorinative coupling of vinylidene difluoride with aryl iodides (Scheme 1a).^9^ Ichikawa and co-workers in 2005 described an intramolecular Pd-catalyzed 5-*endo-trig* cyclization of oxime derivatives bearing the difluoroalkene unit (Scheme 1b).^10^ In these two Heck-type reactions of *gem*-difluoroalkenes, a key β-F elimination, instead of β-H elimination, takes place in the final step. Such transformations belong to the strategy of transition-metal-catalyzed C–F bond functionalization of *gem*-difluoroalkenes for the synthesis of valuable monofluoroalkenes.^11^ Monofluoroalkenes are versatile synthons for organic synthesis and potential peptide bond isosteres for drug discovery.^12^

**Scheme 1:**
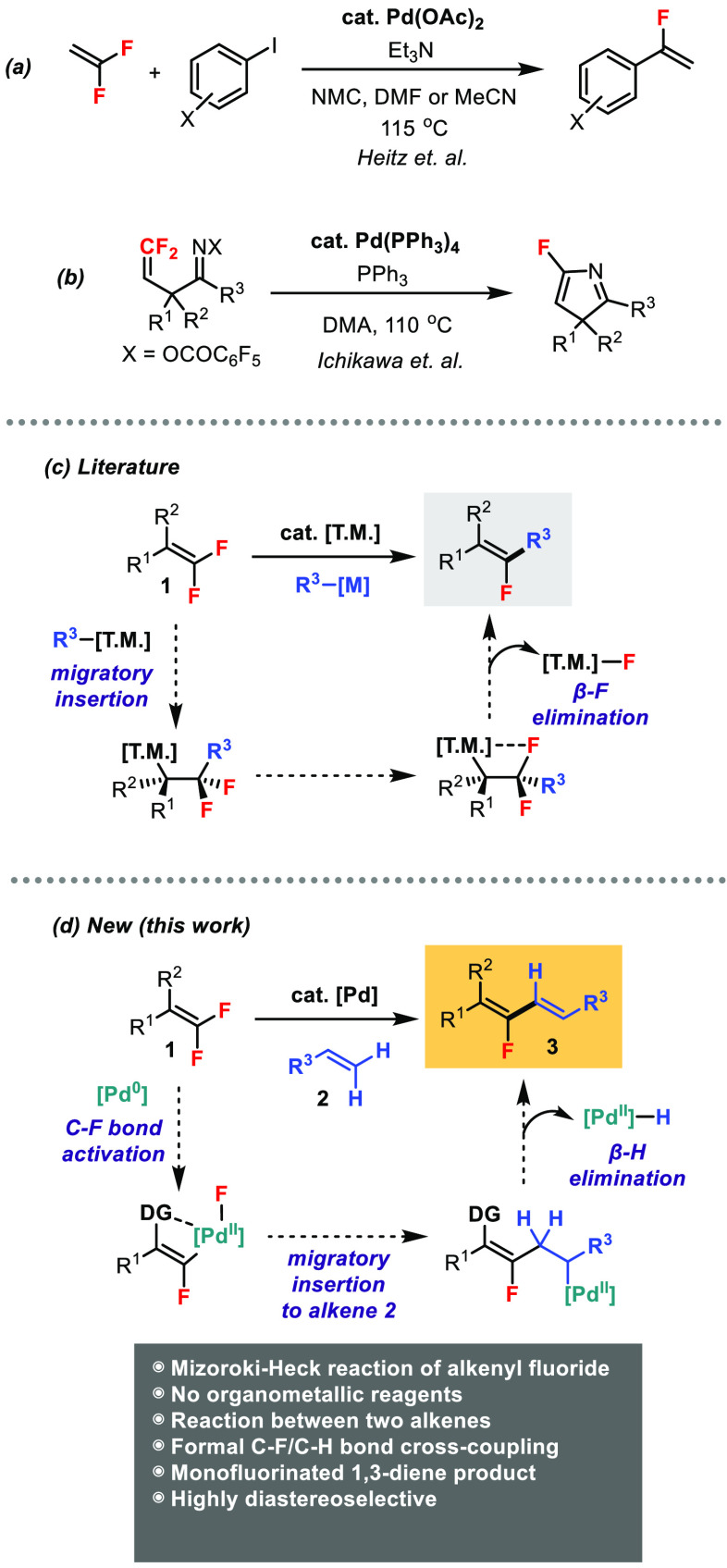
Mizoroki–Heck Reaction of *gem*-Difluoroalkenes

Various transition metal catalysts (T.M.), including Cu, Pd, Ni, Rh, Co, Mn, Ru, Ir, and Fe, have been successfully employed in the C–F bond functionalization of *gem*-difluoroalkenes.^11a^ The general reaction design involves migratory insertion of *gem*-difluoroalkene **1** followed by β-F elimination (Scheme 1c).^13^ High diastereoselectivities can be achieved with trisubstituted *gem*-difluoroalkenes because of steric bias (e.g., R^1^ = aryl, R^2^ = H).^14^ An excellent example of this reaction type is the Pd(II)-catalyzed C–F bond arylation of β,β-difluorostyrenes with boronic acids by Toste and co-workers.^14a^ Our group has previously reported the Pd(0)-catalyzed C–F bond coupling of tetrasubstituted *gem*-difluoroalkenes with organometallic reagents R-[M] (M = B, Si, Sn).^15^ Herein, we present a new reaction motif that is distinct from previous ones where *gem*-difluoroalkene **1** reacts with another alkene **2** in a Mizoroki–Heck fashion without organometallic reagents (Scheme 1d). This unprecedented transformation involves the following sequence: (1) Pd(0) participates in the directing group (DG)-assisted C–F bond activation; (2) the resulting vinylpalladium(II) species undergoes migratory insertion to alkene **2**; and (3) β-H elimination generates the monofluorinated diene product **3**. Hence, the formal stereoselective C–F and C–H bond coupling of two types of alkenes can be achieved.

We began our studies by using β,β-difluoroacrylate **1a**, a tetrasubstituted *gem*-difluoroalkene, with 4-methylstyrene **2a** as standard substrates under previously developed catalytic conditions for C–F bond alkynylation (Table 1).^16,17^ Through the use of Pd(PPh_3_)_4_ as the catalyst in the presence of additive NaI and base Et_3_N in toluene at 80 °C, the desired Heck product **3a** was obtained in 41% yield as the (*E,E*)-diastereomer (dr > 99:1) (entry 1). The remaining mass balance was mainly the unreacted **1a** and hydrodefluorinated side product.^15b^ Reactions without either NaI (entry 2) or Et_3_N (entry 3) gave very poor yields. Raising the temperature to 90 °C improved the conversion (entry 4). Subsequent ligand screening using Pd(dba)_2_ revealed that dppb was an effective ligand (entries 5–9). The use of Pd(dba)_2_ alone gave no reaction (entry 10). A lower catalyst loading caused a yield decrease (entry 11). Screening of other Pd(0) or Pd(II) catalysts with dppb showed inferior reactivities (entries 12–15). Finally, an increase in the reaction concentration significantly enhanced the yield, and (*E,E*)-**3a** was isolated in 81% yield (entry 16). Isomerization of the tetrasubstituted double bond was observed during column chromatography, which resulted in a small amount of the (*Z,E*)-diastereomer **3a′** (**3a**/**3a′** = 96:4). Other reaction parameters, including additives, bases, and solvents, were also screened.^17^ Salt additives, such as NaF, NaCl, LiI, or KI, were not as effective as NaI. Other bases, such as K_3_PO_4_, K_2_CO_3_, tetramethylethylenediamine (TMEDA), or *N*,*N*-diisopropylethylamine (DIPEA), gave lower yields than Et_3_N.

**Table 1:**
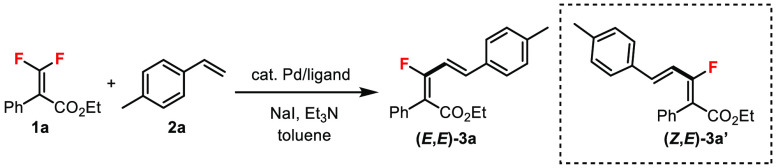
Screening of Pd Catalysts and Ligands^a^

entry	Pd (*x* mol %)/ligand (*y* mol %)	yield of (*E,E*)-**3a** (%)^b^/ratio of **3a**/**3a′**^b^
1^c^	Pd(PPh_3_)_4_ (10)/-	41/>99:1
2^c^^,^^d^	Pd(PPh_3_)_4_ (10)/-	0/-
3^c^^,^^e^	Pd(PPh_3_)_4_ (10)/-	8/-
4	Pd(PPh_3_)_4_ (10)/-	58/>99:1
5	Pd(dba)_2_ (10)/PPh_3_ (20)	55/>99:1
6	Pd(dba)_2_ (10)/dppm (10)	0/-
7	Pd(dba)_2_ (10)/dppe (10)	22/>99:1
8	Pd(dba)_2_ (10)/dppp (10)	26/>99:1
9	Pd(dba)_2_ (10)/dppb (10)	62/>99:1
10	Pd(dba)_2_ (10)/-	0/-
11	Pd(dba)_2_ (5)/dppb (5)	50/99:1
12	Pd_2_(dba)_3_ (5)/dppb (10)	8/-
13	Pd(PPh_3_)_4_ (10)/dppb (10)	44/>99:1
14	Pd(OAc)_2_ (10)/dppb (10)	8/-
15	Pd(TFA)_2_ (10)/dppb (10)	25/99:1
**16^f^**	**Pd(dba)_2_(10)/dppb (10)**	**87 (81)^g^**/**>99:1 (96:4)^g^**

a Unless specified otherwise, reactions were carried out using **1a** (0.1 mmol), **2a** (0.3 mmol), NaI (0.3 mmol), and Et_3_N (0.3 mmol) in toluene (0.2 M) at 90 °C for 18 h under argon.

b Determined by ^19^F NMR analysis using benzotrifluoride as the internal standard.

c At 80 °C.

d Without NaI.

e Without Et_3_N.

f Concn = 0.67 M.

g Isolated yield and ratio in parentheses at 0.2 mmol scale.

The scope of the alkene component was subsequently investigated in the Mizoroki–Heck reaction of **1a** under the optimized conditions (Scheme 2). Commercial and easily prepared styrenes were employed to study the functional group tolerability of the aromatic substituent groups (**3b**–**m**). Electron-donating/-withdrawing groups and halogens were tolerated, which provided products in moderate to good yields. Even sensitive chloro (**3g**) and bromo (**3h**) groups were tolerated at the *para* position; however, the *ortho*-bromo (**3i**) group gave a lower yield. The diastereoselectivities were excellent and favored the (*E,E*)-isomer regardless of the substituents. Reaction at a 1.0 mmol scale was also demonstrated (**3b**). The structure of the product and the *E* configuration of both double bonds were unambiguously confirmed through the X-ray structure of **3j**. Moreover, multisubstituted arene (**3n**) and heteroarene (**3o**) groups and dienes (**3p**) were tolerated. Electron-deficient alkenes, such as α,β-unsaturated esters (**3q**–**r**) and amides (**3s**), could also be used, albeit in lower diastereomeric ratios. Unactivated alkenes, such as 1-octene, and 1,1-/1,2-disubstituted alkenes, such as *α-/β*-methylstyrene and 1,1-diphenylethylene, were unreactive in this reaction.

**Scheme 2:**
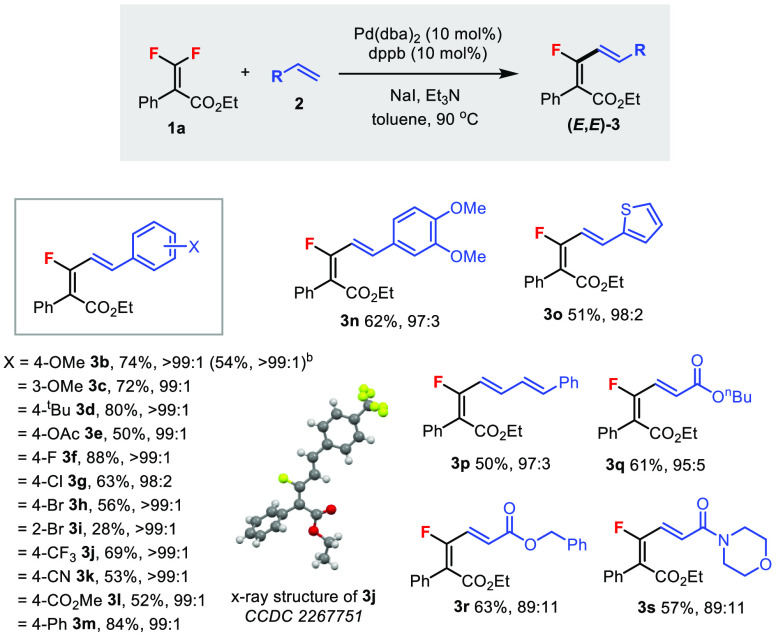
Scope of Alkenes **2** in the Mizoroki–Heck Reaction Unless specified otherwise, reactions were carried out using **1a** (0.2 mmol), **2** (0.6 mmol), NaI (0.6 mmol), and Et_3_N (0.6 mmol) in toluene (0.3 mL) for 18 h under argon. Isolated yields. Diastereomeric ratios of (*E*,*E*)-**3**/(*Z*,*E*)-**3′** were determined by ^19^F NMR analysis of the isolated products. At 1.0 mmol scale.

Next, the scope of the *gem*-difluoroalkene component was explored (Scheme 3). Aryl substituents (R^1^), including electron-donating/-withdrawing and halo groups, were tolerated (**4a**–**f**). Naphthyl and thienyl groups were also demonstrated (**4g**–**h**). Varying the ester substituent group (R^2^) did not lead to much change in the dr values (**4i**–**j**). With benzyl substituents (**4k**–**n**), tripropylamine was found to be a more suitable base. Long chain alkyl groups were also compatible (**4o**). Overall, good to excellent diastereoselectivities (92:8 to >99:1) were obtained across various *gem*-difluoroalkenes **1**.

**Scheme 3:**
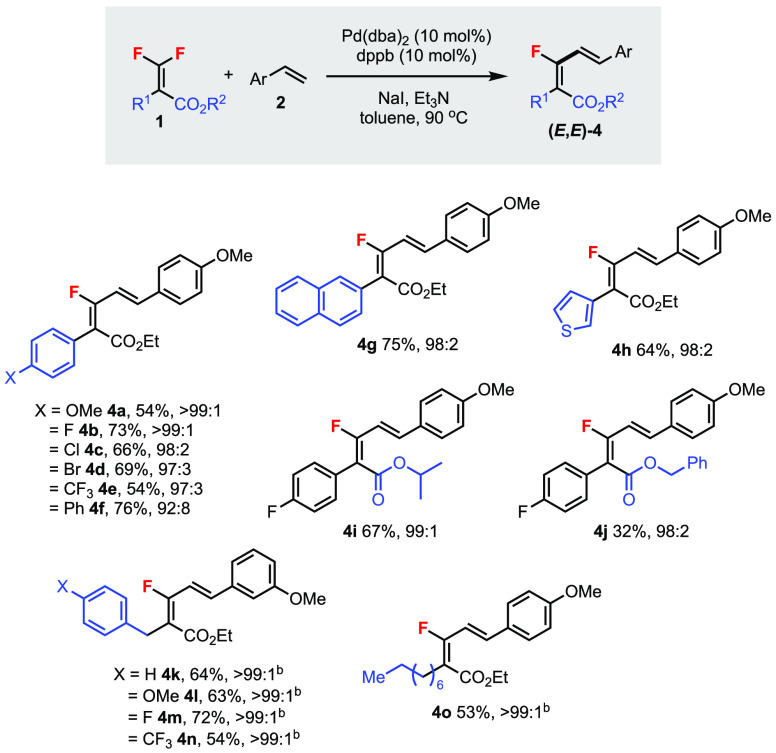
Scope of *gem*-Difluoroalkenes **1** in the Mizoroki–Heck Reaction Unless specified otherwise, reactions were carried out using **1a** (0.2 mmol), **2** (0.6 mmol), NaI (0.6 mmol), and Et_3_N (0.6 mmol) in toluene (0.3 mL) for 18 h under argon. Isolated yields. Diastereomeric ratios of (*E*,*E*)-**4**/(*Z*,*E*)-**4′** were determined by ^19^F NMR analysis of the isolated products. Used tripropylamine instead of Et_3_N, 36 h.

Further experiments were performed to shed light on the reaction details (Scheme 4). Under standard conditions (Scheme 2), trisubstituted difluorostyrene derivative **5** and difluoroacrylate derivative **6** gave no alkenylation products because of poor reactivity and substrate decomposition, respectively (Scheme 4a,b), which showed that the tetrasubstituted *gem*-difluoroalkenes **1** were both reactive and stable for this transformation. The monofluorovinylpalladium(II) intermediate **Int-1** (dr > 99:1) was prepared according to a previous procedure (Scheme 4c).^16^ Subjecting **Int-1** to 4-methoxystyrene and base afforded the desired product **3b** in 71% yield (dr > 99:1). Furthermore, by applying the current catalytic conditions using dppb as the ligand, a signal corresponding to the Pd(II) intermediate **Int-2** was detected by ^19^F NMR (Scheme 4d).^17^ In a similar fashion, **Int-2** also led to product **3b** in >99:1 dr. These experiments provided support for the proposed mechanism involving the formation of a Pd(II) intermediate via chelation-controlled C–F bond activation prior to migratory insertion of alkene (Scheme 1d).

**Scheme 4:**
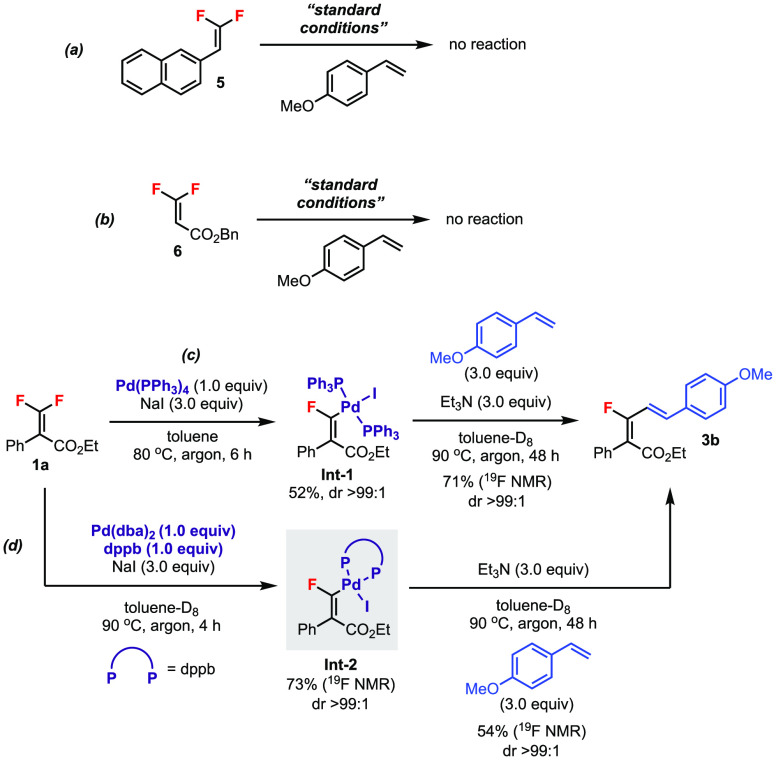
Control Experiments

In conclusion, we developed a Pd(0)-catalyzed Mizoroki–Heck reaction of *gem*-difluoroalkenes to accomplish highly diastereoselective C–F bond alkenylation. The reaction takes place between two different alkenes without organometallic reagents in a formal C–F/C-H bond cross-coupling. The products are monofluorinated 1,3-dienes, which are challenging to synthesize with control of each alkene geometry. Further exploration of this reaction motif is ongoing in our laboratory.

## Data Availability

The data underlying this study are available in the published article and its Supporting Information.

## Supporting Information Available

Experimental procedures, optimization data, characterization data, and spectral data (PDF)
