# Supplementary material for: Stereoselective Palladium-Catalyzed C–F Bond Alkenylation of Tetrasubstituted gem-Difluoroalkenes via Mizoroki–Heck Reaction
Source: Org Lett. 2023 Aug 16;25(33):6217–21. doi: 10.1021/acs.orglett.3c02452 (PMC10463225; doi:10.1021/acs.orglett.3c02452)

Supporting Information

Stereoselective Palladium-Catalyzed C-F Bond Alkenylation of Tetrasubstituted *gem*-Difluoroalkenes via Mizoroki-Heck Reaction

Yanhui Wang, Gavin Chit Tsui*[a]

[a] Y. Wang, Prof. Gavin Chit Tsui
Department of Chemistry, The Chinese University of Hong Kong, Shatin, New Territories, Hong Kong SAR, China
E-mail: gctsui@cuhk.edu.hk

Experimental Procedures and Spectral Data

Table of Contents:

I. General Experimental **S2**
II. Materials **S2**
III. Instrumentation **S2**
IV. Experimental Procedures **S4**
V. X-Ray Structure of **3j** **S6**
VI. Optimization Studies **S8**
VII. Mechanistic studies **S10**
VIII. References **S12**
IX. Characterization Data **S13**
X. Spectra **S25**

I. General Experimental.

Unless otherwise noted, C-F bond activation reactions were carried out under argon in a 10 mL glass tube with magnetic stirring. Reactions that require heating were carried out in the oil bath. Analytical thin layer chromatography (TLC) was performed with Merck silica gel 60 F_{254} aluminum plates. Visualization was done under a UV lamp (254 nm) and by immersion in potassium permanganate ($KMnO_4$), followed by heating using a heat gun. Organic solutions were concentrated by rotary evaporation at 23-35 °C. Purification of reaction products were generally done by flash column chromatography with Silicycle 60-230 mesh silica gel.

II. Materials.

Anhydrous NaI, $TMSCF_3$, $TMSCF_2Br$, *n*-Bu_4NB_r, $Pd(dba)_2$, dppb, Et_3N, $(n\text{-propyl})_3N$ were purchased from J&K Scientific, Aladdin, Acros, Energy Chemical. Diazo compounds for substates synthesis were prepared according to literature procedure. Other chemicals for substrates preparation were purchased from Acros, J&K Scientific, Aldrich and Dikemann.

III. Instrumentation.

Proton nuclear magnetic resonance spectra (^1H NMR), carbon nuclear magnetic resonance spectra (^{13}C NMR) and fluorine nuclear magnetic resonance spectra (^{19}F NMR) were recorded at 23 °C on Bruker 400 MHz or 500 MHz spectrometer in $CDCl_3$. Chemical shifts of ^1H NMR spectra were reported as parts per million in δ scale using residual solvent signal ($CDCl_3$: 7.26 ppm) or tetramethylsilane (0.00 ppm) as internal standard. Chemical shifts of ^{13}C NMR spectra were reported using residual solvent signal of $CDCl_3$ (77.16 ppm) on the δ scale. Chemical shifts of ^{19}F NMR were reported as parts per million in δ scale using benzotrifluoride (-63.72 ppm) as internal standard. Data are represented as follows: chemical shift (δ ppm), multiplicity (s = singlet, d = doublet, t = triplet, q = quartet, m = multiplet), coupling constant (J, Hz) and integration. High resolution mass spectra (HRMS) were obtained on a Finnigan MAT 95XL GC Mass Spectrometer or a Thermo Scientific Q Exactive Focus Mass Spectrometer or a Bruker SolariX 9.4T FTMS with Q Exactive Focus Orbitrap. X-ray crystallography was conducted on a Bruker D8 Venture X-Ray Diffractometer.

Substrates 1 (tetrasubstituted *gem*-difluoroalkenes)

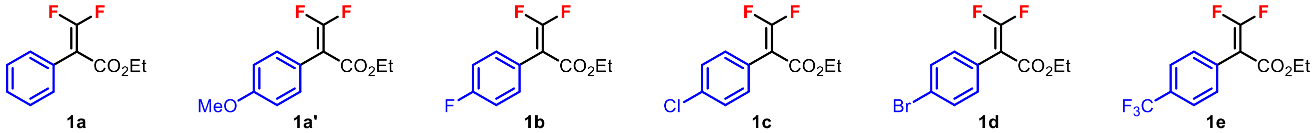

1a 1a' 1b 1c 1d 1e

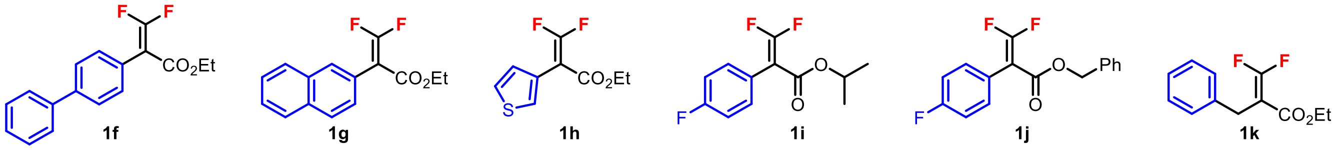

1f 1g 1h 1i 1j 1k

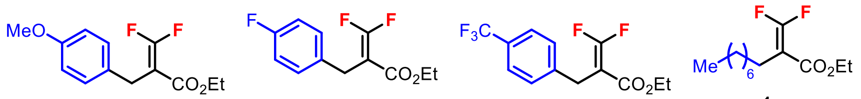

1l 1m 1n 1o

Note: substrates **1** are known compounds and prepared according to literature procedures.[1, 2]

Commercial alkene 2

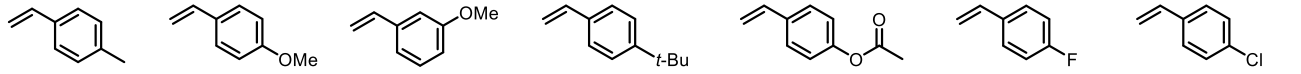

2a 2b 2c 2d 2e 2f 2g

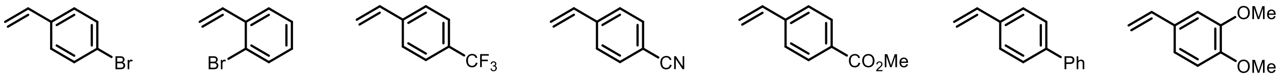

2h 2i 2j 2k 2l 2m 2n

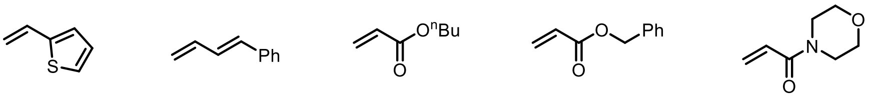

2o 2p 2q 2r 2s

IV. Experimental Procedures.

General procedure (I) for the synthesis of *gem*-difluoroalkenes 1 (using 1a as an example):

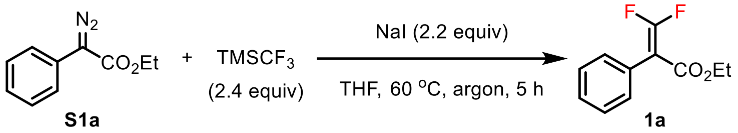

gem-Difluoroalkenes **1** (except **1d** and **1e**) were synthesized according to literature procedure.[1] For example, to an oven-dried 100 mL flask equipped with a stir bar was added dry NaI (989.3 mg, 6.6 mmol, 2.2 equiv), followed by the addition of **S1a** (570.3 mg, 3 mmol, 1.0 equiv) and $TMSCF_3$ (1.0238 g, 7.2 mmol, 2.4 equiv) in 60 mL anhydrous THF under argon. The resulting mixture was heated at 60 °C with an oil bath for 5 h with rigorous stirring. After cooling to room temperature, the reaction mixture was extracted with 200 mL CH_2Cl_2, washed with H_2O (80 mL) then brine (80 mL), dried over $MgSO_4$ and concentrated in vacuo. The residue was purified by flash column chromatography on silica gel to afford *gem*-difluoroalkene **1a** as a colorless oil (420.5mg, 66% yield).

General procedure (II) for the synthesis of *gem*-difluoroalkenes 1 (using 1e as an example):

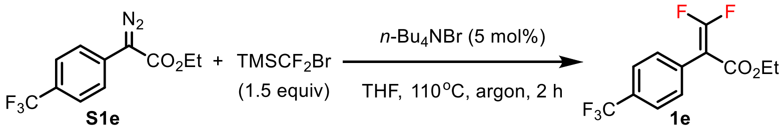

gem-Difluoroalkenes **1d** and **1e** were synthesized according to literature procedure.[2] For example, to an oven-dried 10 mL pressure tube equipped with a stir bar was added *n*-Bu_4NBr (16.1 mg, 0.05 mmol, 5 mol%), the tube was sealed with the septum, evacuated and refilled with argon three times. Under argon protection, the tube was opened, diazo **S1e** (258.2 mg, 1.0 mmol, 1.0 equiv) and $TMSCF_2Br$ (304.6 mg, 1.5 mmol, 1.5 equiv) in THF (10 mL, 0.1 M) were added via syringe. Then the tube was sealed again and heated to 110 °C for 2 h. After cooling to room temperature, the reaction mixture was extracted with 50 mL CH_2Cl_2, washed with H_2O (40 mL) then brine (40 mL), dried over $MgSO_4$ and concentrated in vacuo. The residue was purified by flash column chromatography on silica gel to afford *gem*-difluoroalkene **1e** as a yellow oil (159.7 mg, 57% yield).

General procedure (III) for the stereoselective synthesis of (*E*,*E*)-monofluoroalkenes (*cf.* Scheme 2 and Scheme 3, 4a-4j) :

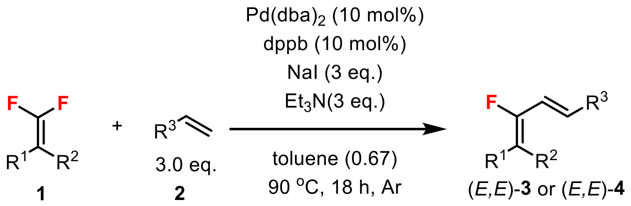

To an oven-dried glass tube equipped with a stir bar was added $Pd(dba)_2$ (11.6 mg, 0.02 mmol, 10 mol%), dppb (8.6 mg, 0.02 mmol, 10 mol%) and dry NaI (90 mg, 0.6 mmol, 3.0 equiv). The tube was sealed with a septum, evacuated and refilled with argon three times. Then 0.3 mL (0.67 M) toluene was added under argon through syringe. The resulting mixture was stirred at room temperature for 5 mins, then substrate **1** (0.2 mmol, 1.0 equiv), alkene **2** (0.6 mmol, 3.0 equiv)) and Et_3N (60.7 mg, 83.6 μL, 0.6 mmol, 3.0 equiv)) was added and heated at 90 °C with stirring in an oil bath for 18 h. After cooling to room temperature, the diastereomeric ratio (d.r.) was determined by ^{19}F NMR using benzotrifluoride (12 μL, 0.1 mmol) as internal standard with the crude sample. The reaction mixture was extracted with CH_2Cl_2 (3 × 10 mL). The combined organic layers were washed with H_2O (2 × 10 mL), then brine (2 × 10 mL), dried over $MgSO_4$ and concentrated in vacuo. The residue was purified by flash column chromatography on silica gel to afford products (*E*,*E*)-**3** and (*E*,*E*)-**4.**

General procedure (IV) for the stereoselective synthesis of (*E*,*E*)-monofluoroalkenes (*cf.* Scheme 3, 4k-4o):

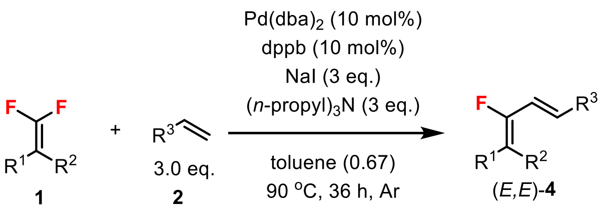

To an oven-dried glass tube equipped with a stir bar was added $Pd(dba)_2$ (11.6 mg, 0.02 mmol, 10 mol%), dppb (8.6 mg, 0.02 mmol, 10% mol) and dry NaI (90 mg, 0.6 mmol, 3.0 equiv). The tube was sealed with a septum, evacuated and refilled with argon three times. Then 0.3 mL toluene (0.67 M) was added under argon through syringe. The resulting mixture was stirred at room temperature for 5 mins, then substrate **1** (0.2 mmol, 1.0 equiv), alkene **2** (0.6 mmol, 3.0 equiv) and (n-propyl)$_3$N (86.0 mg, 114 µL, 0.6 mmol, 3.0 equiv) was added and heated at 90 °C with stirring in an oil bath for 36 h. After cooling to room temperature, the diastereomeric ratio (d.r.) was determined by ^{19}F NMR using benzotrifluoride (12 µL, 0.1 mmol) as internal standard with the crude sample. The reaction mixture was extracted with CH_2Cl_2 (3 × 10 mL). The combined organic layers were washed with H_2O (2 × 10 mL), then brine (2 × 10 mL), dried over $MgSO_4$ and concentrated in vacuo. The residue was purified by flash column chromatography on silica gel to afford products (*E*,*E*)-**4**.

Larger-scale synthesis of (*E*,*E*)-3c (*cf.* Scheme 2, 3b):

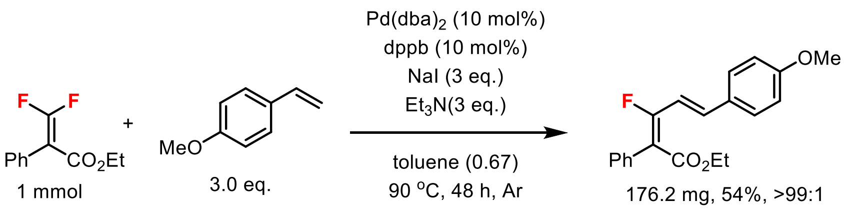

To an oven-dried glass tube equipped with a stir bar was added $Pd(dba)_2$ (57.5 mg, 0.1 mmol), dppb (42.6 mg, 0.02 mmol) and dry NaI (449.7 mg, 3 mmol). The tube was sealed with a septum, evacuated and refilled with argon three times. Then 3 mL toluene was added under argon through syringe. The resulting mixture was stirred at room temperature for 5 mins, then substrate **1a** (212.2 mg, 1.0 mmol), 4-vinylanisole **2b** (402.5 mg, 400 µL, 3.0 mmol) and Et_3N (303.6 mg, 418 µL, 3.0 mmol) was added and heated at 90 °C with stirring in an oil bath for 48 h. After cooling to room temperature, the diastereomeric ratio (d.r.) (>99:1) was determined by ^{19}F NMR using benzotrifluoride (12 µL, 0.1 mmol) as internal standard with the crude sample. The reaction mixture was extracted with CH_2Cl_2 (3 × 10 mL). The combined organic layers were washed with H_2O (2 × 10 mL), then brine (2 × 10 mL), dried over $MgSO_4$ and concentrated in vacuo. The residue was purified by flash column chromatography on silica gel (ethyl acetate: hexane = 1 : 50) to afford products (*E*,*E*)-**3b** (176.2 mg, 54%; dr>99:1, ^{19}F NMR).

V. X-Ray Structure of 3j

Crystals of **3j** were obtained by slow diffusion from the solution in $CHCl_3$ layered *n*-hexane. The crystal was kept at 296 K during data collection. Crystallographic data for **3j** has been deposited with the Cambridge Crystallographic Data Centre (CCDC) under deposition number 2267751.

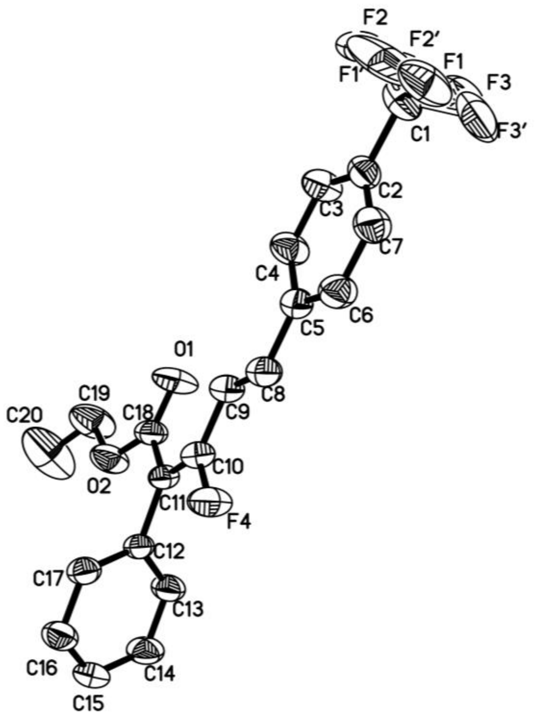

Molecular structure of **3j** with thermal ellipsoids at the 50% probability level

Crystal data and structure refinement for **3j.**

Identification code	2267751	
Empirical formula	$C_{20}H_{16}F_4O_2$	
Formula weight	364.34	
Temperature	296(2) K	
Wavelength	0.71073 Å	
Crystal system	Monoclinic	
Space group	$P2_1/c$	
Unit cell dimensions	a = 11.4890(14) Å	α= 90°.
	b = 17.983(2) Å	β= 96.792(4)°.
	c = 8.8566(10) Å	γ = 90°.
Volume	1817.0(4) $Å^3$	
Z	4	
Density (calculated)	1.332 Mg/m^3	
Absorption coefficient	0.112 mm^{-1}	
F(000)	752	
Crystal size	0.400 x 0.300 x 0.200 mm^3	
Theta range for data collection	2.578 to 25.246°.	
Index ranges	-13<=h<=13, -21<=k<=21, -10<=l<=10	
Reflections collected	27989	
Independent reflections	3288 [R(int) = 0.0454]	
Completeness to theta = 25.242°	99.5 %	

Absorption correction	multi-scan
Max. and min. transmission	0.7456 and 0.6202
Refinement method	Full-matrix least-squares on F^2
Data / restraints / parameters	3288 / 0 / 263
Goodness-of-fit on F^2	1.039
Final R indices [I>2sigma(I)]	R1 = 0.0623, wR2 = 0.1663
R indices (all data)	R1 = 0.0828, wR2 = 0.1841
Extinction coefficient	n/a
Largest diff. peak and hole	0.365 and -0.217 e.Å^{-3}

VI. Optimization studies using substrate 1a.[a] (*cf.* Table 1)

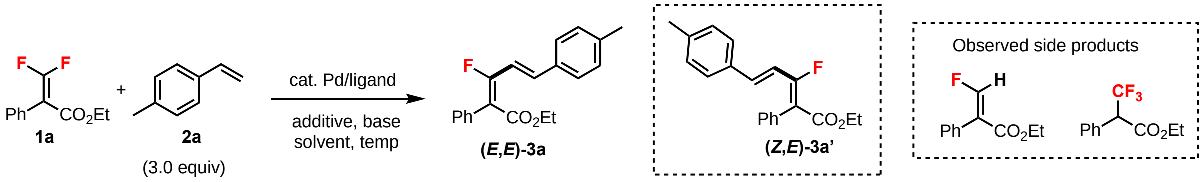

entry	Pd (x mol %)	Ligand (x mol %)	Additive (equiv)	Base (equiv)	Temp (°C)/time (h)	Solvent (M)	(*E*,*E*)-**3a** (%)[b]	ratio of **3a/3a'**
1	$Pd(PPh_3)_4$ (10)	none	NaI (3.0)	Et_3N (3.0)	80/18	toluene (0.2)	41	>99:1
2	none	none	NaI (3.0)	Et_3N (3.0)	80/18	toluene (0.2)	0	-
3	$Pd(PPh_3)_4$ (10)	none	none	Et_3N (3.0)	80/18	toluene (0.2)	0	-
4	$Pd(PPh_3)_4$ (10)	none	NaI (3.0)	none	80/18	toluene (0.2)	8	-
5	$Pd(PPh_3)_4$ (10)	none	NaI (3.0)	Et_3N (3.0)	90/18	toluene (0.2)	58	>99:1
6	$Pd(PPh_3)_4$ (10)	none	NaI (3.0)	Et_3N (3.0)	100/18	toluene (0.2)	57	>99:1
7	$Pd(dba)_2$ (10)	PPh_3 (20)	NaI (3.0)	Et_3N (3.0)	90/18	toluene (0.2)	55	>99:1
8	$Pd(dba)_2$ (10)	dppm (10)	NaI (3.0)	Et_3N (3.0)	90/18	toluene (0.2)	0	-
9	$Pd(dba)_2$ (10)	dppe (10)	NaI (3.0)	Et_3N (3.0)	90/18	toluene (0.2)	22	>99:1
10	$Pd(dba)_2$ (10)	dppp (10)	NaI (3.0)	Et_3N (3.0)	90/18	toluene (0.2)	26	>99:1
11	$Pd(dba)_2$ (10)	dppb (10)	NaI (3.0)	Et_3N (3.0)	90/18	toluene (0.2)	62	>99:1
12	$Pd(dba)_2$ (10)	dppbz (10)	NaI (3.0)	Et_3N (3.0)	90/18	toluene (0.2)	0	-
13	$Pd_2(dba)_3$ (5)	dppb (10)	NaI (3.0)	Et_3N (3.0)	90/18	toluene (0.2)	8	-
14	$Pd(PPh_3)_4$ (10)	dppb (10)	NaI (3.0)	Et_3N (3.0)	90/18	toluene (0.2)	44	>99:1
15	$Pd(OAc)_2$ (10)	dppb (10)	NaI (3.0)	Et_3N (3.0)	90/18	toluene (0.2)	8	-
16	$Pd(TFA)_2$ (10)	dppb (10)	NaI (3.0)	Et_3N (3.0)	90/18	toluene (0.2)	25	99:1
17	$Pd(dba)_2$ (10)	dppb (10)	NaF (3.0)	Et_3N (3.0)	90/18	toluene (0.2)	0	-
18	$Pd(dba)_2$ (10)	dppb (10)	NaCl (3.0)	Et_3N (3.0)	90/18	toluene (0.2)	16	>99:1
19	$Pd(dba)_2$ (10)	dppb (10)	NaBr (3.0)	Et_3N (3.0)	90/18	toluene (0.2)	62	>99:1
20	$Pd(dba)_2$ (10)	dppb (10)	LiI (3.0)	Et_3N (3.0)	90/18	toluene (0.2)	0	-
21	$Pd(dba)_2$ (10)	dppb (10)	KI (3.0)	Et_3N (3.0)	90/18	toluene (0.2)	20	>99:1
22	$Pd(dba)_2$ (10)	dppb (10)	NaI (1.5)	Et_3N (3.0)	90/18	toluene (0.2)	48	98:2
23	$Pd(dba)_2$ (10)	dppb (20)	NaI (3.0)	Et_3N (3.0)	90/18	toluene (0.2)	30	>99:1
24	$Pd(dba)_2$ (10)	dppb (15)	NaI (3.0)	Et_3N (3.0)	90/18	toluene (0.2)	25	>99:1
25	$Pd(dba)_2$ (10)	dppb (5)	NaI (3.0)	Et_3N (3.0)	90/18	toluene (0.2)	29	99:1
26	$Pd(dba)_2$ (10)	none	NaI (3.0)	Et_3N (3.0)	90/18	toluene (0.2)	0	-
27	$Pd(dba)_2$ (5)	dppb (5)	NaI (3.0)	Et_3N (3.0)	90/18	toluene (0.2)	50	99:1
28	$Pd(dba)_2$ (2)	dppb (2)	NaI (3.0)	Et_3N (3.0)	90/18	toluene (0.2)	29	97:3
29	$Pd(dba)_2$ (10)	dppb (10)	NaI (3.0)	Et_3N (3.0)	90/48	toluene (0.2)	80	>99:1
30	$Pd(dba)_2$ (10)	dppb (10)	NaI (3.0)	Et_3N (3.0)	90/18	toluene (0.4)	82	>99:1
**31**	**$Pd(dba)_2$ (10)**	**dppb (10)**	**NaI (3.0)**	**Et_3N (3.0)**	**90/18**	**toluene (0.67)**	**87 (81)[c]**	**>99:1 (96:4)[c]**
32	$Pd(dba)_2$ (10)	dppb (10)	NaI (3.0)	Et_3N (1.5)	90/18	toluene (0.4)	72	99:1
33[d]	$Pd(dba)_2$ (10)	dppb (10)	NaI (3.0)	Et_3N (3.0)	90/18	toluene (0.4)	46	98:2
34	$Pd(dba)_2$ (10)	dppb (10)	NaI (3.0)	Et_3N (3.0)	90/18	m-xylene (0.4)	77	>99:1
35	$Pd(dba)_2$ (10)	dppb (10)	NaI (3.0)	Et_3N (3.0)	90/18	mesitylene (0.4)	17	>99:1
36	$Pd(dba)_2$ (10)	dppb (10)	NaI (3.0)	Et_3N (3.0)	90/18	1,4-dioxane(0.4)	59	99:1
37	$Pd(dba)_2$ (10)	dppb (10)	NaI (3.0)	Et_3N (3.0)	90/18	DMF (0.4)	65	92:8

38	$Pd(dba)_2$ (10)	dppb (10)	NaI (3.0)	K_3PO_4 (3.0)	90/18	toluene (0.4)	16	>99:1
39	$Pd(dba)_2$ (10)	dppb (10)	NaI (3.0)	K_2CO_3 (3.0)	90/18	toluene (0.4)	15	>99:1
40	$Pd(dba)_2$ (10)	dppb (10)	NaI (3.0)	TMEDA (3.0)	90/18	toluene (0.4)	trace	-
41	$Pd(dba)_2$ (10)	dppb (10)	NaI (3.0)	DIPEA (3.0)	90/18	toluene (0.4)	51	>99:1
42	$Pd(dba)_2$ (10)	dppb (10)	NaI (3.0)	$(n\text{-}Pr)_3N$ (3.0)	90/18	toluene (0.4)	77	>99:1
43	$Pd(dba)_2$ (10)	dppb (10)	NaI (3.0)	$(n\text{-}Bu)_3N$ (3.0)	90/18	toluene (0.4)	88	98:2
44	$Pd(dba)_2$ (10)	dppb (10)	NaI (3.0)	$(i\text{-}Bu)_3N$ (3.0)	90/18	toluene (0.4)	13	>99:1
45	$Pd(dba)_2$ (10)	dppb (10)	NaI (3.0)	Et_3N (2.0) DIPEA (1.0)	90/18	toluene (0.4)	71	>99:1

[a]Unless specified otherwise, reactions were carried out using **1a** (0.1 mmol) under argon. [b]Determined by ^{19}F NMR analysis using benzotrifluoride as internal standard. [c]Isolated yield and ratio in parentheses at 0.2 mmol scale. [d]Used 1.5 equiv **2a**.

Optimization studies using substrate 1k.[a]

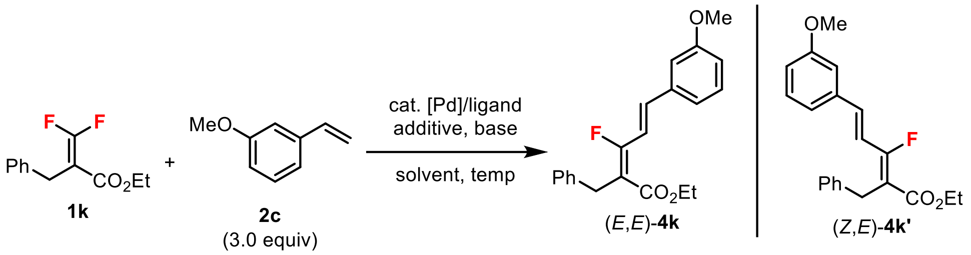

entry	[Pd] (x mol %)	Ligand (x mol %)	Additive (equiv)	Base (equiv)	Temp(°C) /time (h)	Solvent (M)	(*E*,*E*)-**4k** (^{19}F %)[b]	(*E*,*E*)-**4k**/(*Z*,*E*)-**4k'**[c]
1	$Pd(dba)_2$ (10)	dppb (10)	NaI (3)	Et_3N(3)	90/18	toluene (0.67)	40	>99:1
2	$Pd(dba)_2$ (10)	dppb (10)	NaI (3)	$(n\text{-}Pr)_3N$(3)	90/18	toluene (0.4)	38	>99:1
3	$Pd(dba)_2$ (10)	dppb (10)	NaI (3)	$(n\text{-}Bu)_3N$(3)	90/18	toluene (0.4)	39	>99:1
4	$Pd(dba)_2$ (10)	dppb (10)	NaI (3)	$(n\text{-}Pr)_3N$(3)	90/18	toluene (0.67)	71 (46)	>99:1(>99:1)
5	$Pd(dba)_2$ (10)	dppb (10)	NaI (3)	$(n\text{-}Bu)_3N$(3)	90/18	toluene (0.67)	67	>99:1
6	$Pd(dba)_2$ (10)	dppb (10)	NaI (3)	$(n\text{-}Pr)_3N$(3)	100/18	toluene (0.67)	64	>99:1
**7**	**$Pd(dba)_2$ (10)**	**dppb (10)**	**NaI (3)**	**$(n\text{-}Pr)_3N$(3)**	**90/36**	**toluene (0.67)**	**85 (64)[d]**	**>99:1(>99:1)**

[a]Unless specified otherwise, reactions were carried out using **1k** (0.2 mmol) under argon. [b]Yield was determined by ^{19}F NMR using benzotrifluoride as internal standard. [c]Diastereomeric ratio was determined by ^{19}F NMR analysis. [d]Isolated yield in parenthesis at 0.4 mmol scale.

VII. Mechanistic studies. (*cf.* Scheme 4)

Preparation of monofluorovinylpalladium(II) iodide complex Int-1 and stoichiometric reaction between Int-1 and alkene:

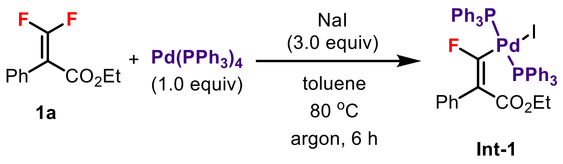

The complex **Int-1** was prepared according to a reported procedure.[2] To an oven-dried 10 mL glass tube equipped with a stir bar was added $Pd(PPh_3)_4$ (116 mg, 0.1 mmol) and NaI (45 mg, 0.3 mmol). The tube was sealed with a septum, evacuated and refilled with argon three times. Then a solution of **1a** (21.2 mg, 0.1 mmol) in 1.5 mL toluene was added through syringe. The resulting mixture was heated at 80 °C with stirring under argon for 6 h, color of the solution changed from light yellow to dark red. The mixture was cooled to room temperature, then filtered with cotton to remove solid residues, which were rinsed with 1 mL toluene resulting in a clear red filtrate solution (~2.5 mL). To this solution was added 25 mL dry hexane, an orange solid precipitated out upon ultrasonication. The solid was then collected by filtration as a crude product (~80 mg). To the crude product was added 4 mL dry hexane followed by ultrasonication, a mixture of yellow and red solids was visible. To this mixture was carefully added CH_2Cl_2 dropwise until most yellow solids dissolved (red solids remained). The red solids were filtered off and the filtrate (clear yellow solution) was collected, and the solvent was removed *in vauo* to give **Int-1** (49.4 mg, 52% yield, light yellow solid). The diastereomeric ratio (d.r. >99:1) was determined by NMR analyses.

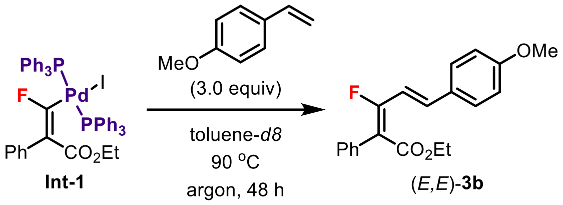

To an oven-dried NMR tube was added **Int-1 (**29 mg, 0.03 mmol) and 4-vinylanisole (12.1 mg, 12.0 µL, 0.09 mmol), the tube was sealed with a septum, evacuated and refilled with argon three times. 0.5 mL toluene-*d8* was then added through syringe under argon. The resulting mixture was heated at 90 °C for 48 h, then cooled to room temperature. (*E*, *E*)-**3b** was obtained in 71% yield (d.r. > 99:1) by ^{19}F NMR analysis using benzotrifluoride (1.8 µL, 0.015 mmol) as internal standard.

Monitoring the reaction by ^{19}F NMR (471 MHz, toluene-*d*8) over time:

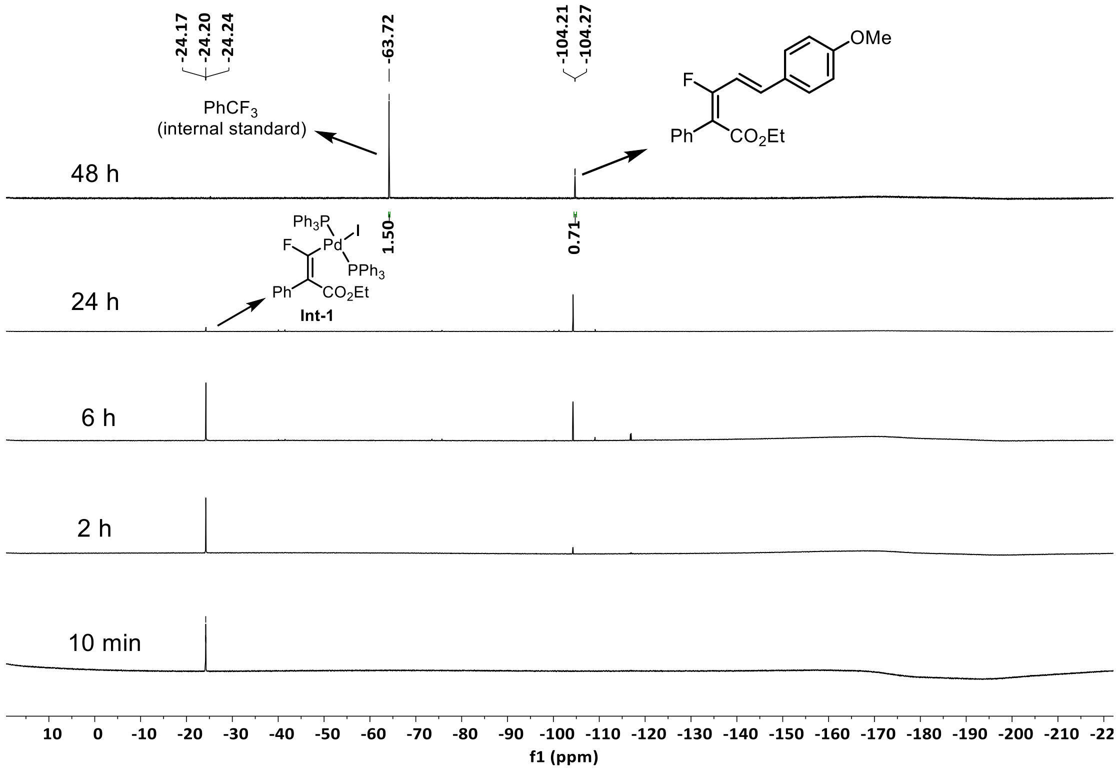

Preparation of monofluorovinylpalladium(II) iodide complex Int-2 and stoichiometric reaction between Int-2 and alkene:

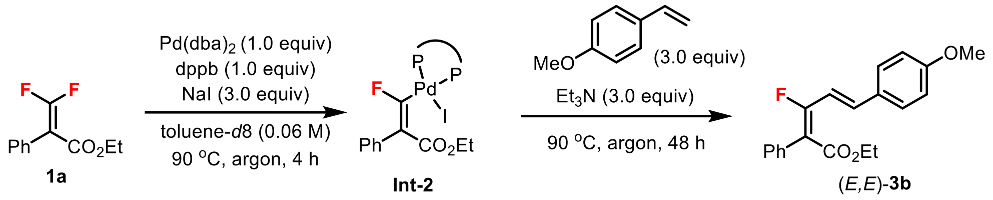

To oven-dried NMR tube equipped was added $Pd(dba)_2$ (11.5 mg, 0.02 mmol), dppb (8.5 mg, 0.02 mmol) and NaI (9.0 mg, 0.06 mmol). The NMR tube was sealed with a septum, evacuated and refilled with argon three times. Then a solution of **1a** (4.2 mg, 0.02 mmol) in 0.5 mL toluene-*d*8 was added through syringe. The resulting mixture was heated at 90 °C under argon for 4 h. The mixture was cooled to room temperature, then cooled to room temperature. **Int-2** was obtained in 73% yield (d.r. > 99:1) by ^{19}F NMR analysis using benzotrifluoride (2.5 μL, 0.02 mmol) as internal standard.

To the NMR tube with **Int-2** was added 4-vinylanisole (8.0 mg, 8.0 μL, 0.06 mmol) and Et_3N (6.1 mg, 8.4 μL, 0.06 mmol) through syringe under argon. The resulting mixture was heated at 90 °C for 48 h. The reaction was monitored by ^{19}F NMR (471 MHz) at 12 h, 24 h and 48 h intervals.

Monitoring the reaction by ^{19}F NMR (471 MHz, toluene-*d*8) over time:

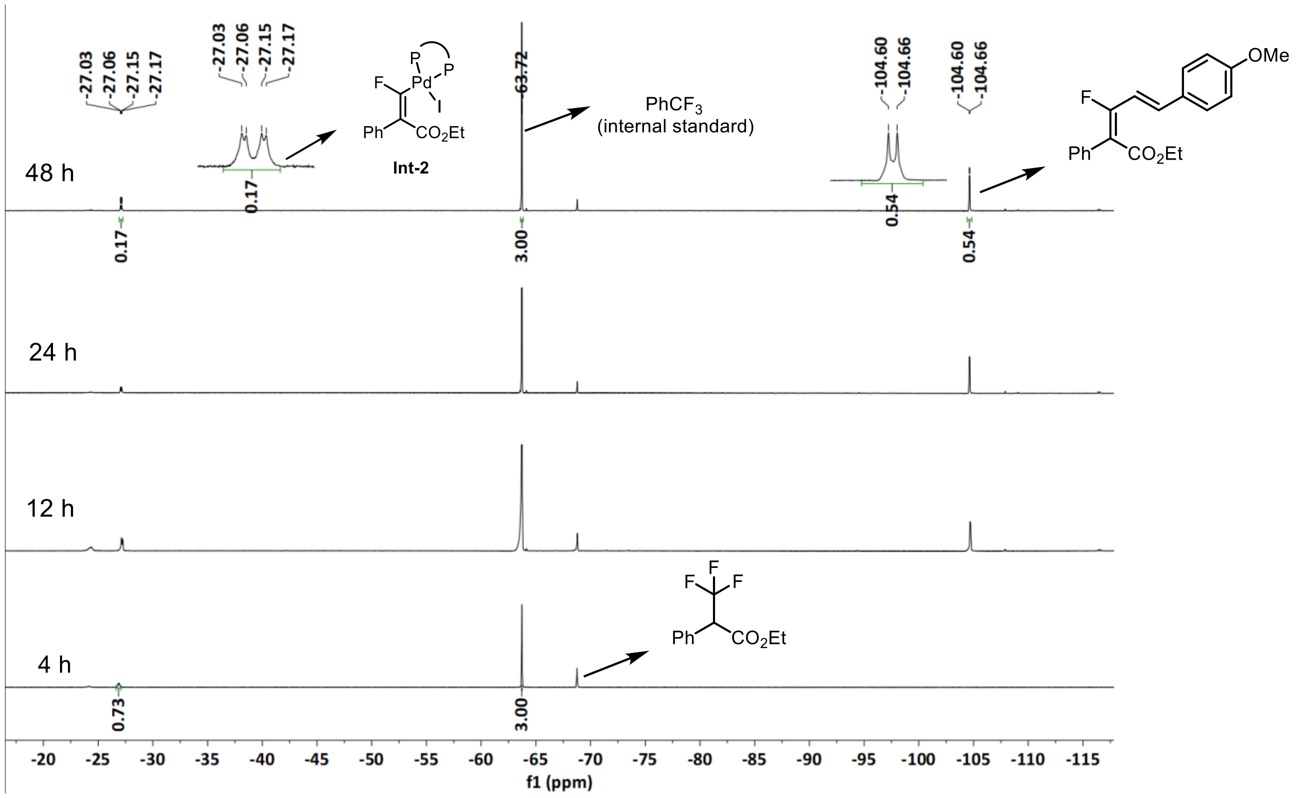

References.

1. Hu, M.; Ni, C.; Li, L.; Han, Y.; Hu, J. *gem*-Difluoroolefination of diazo compounds with $TMSCF_3$ or $TMSCF_2Br$: transition-metal-free cross-coupling of two carbene precursors. *J. Am. Chem. Soc.* **2015**, *137*, 14496-14501.
2. Ma, Q.; Wang, Y.; Tsui, G. C. Stereoselective palladium-catalyzed C-F Bond alkynylation of tetrasubstituted *gem*-difluoroalkenes. *Angew. Chem., Int. Ed.* **2020**, *59*, 11293-11297.

VIII. Characterization Data.

Characterization data of products

ethyl (2*E*,4*E*)-3-fluoro-2-phenyl-5-(p-tolyl)penta-2,4-dienoate (3a)

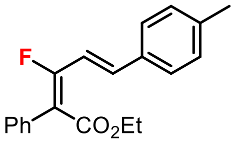

Following the general procedure (III), reaction was run using *gem*-difluoroalkene **1a** (42.4 mg, 0.2 mmol), 4-methylstyrene (70.9 mg, 0.6 mmol), Pd(dba)$_2$ (11.6 mg, 0.02 mmol), dppb (8.6 mg, 0.02 mmol), dry NaI (90 mg, 0.6 mmol) and Et$_3$N (60.7 mg, 83.6 μL, 0.6 mmol) in 0.3 mL toluene in a 90 °C oil bath for 18 h ((*E*,*E*)/(*Z*,*E*) > 99:1). The product was purified by flash column chromatography on silica gel (ethyl acetate : hexane = 1 : 20) and obtained as a colorless oil (50.2 mg, 81% yield, (*E*,*E*)/(*Z*,*E*) = 96:4), R_f = 0.21 (DCM : hexane = 1 : 20). **^{1}H NMR** (500 MHz, CDCl$_3$): δ (ppm) 7.66 (dd, *J* = 27.6, 16.0 Hz, 1H), 7.38 – 7.11 (m, 10H), 4.20 (q, *J* = 7.1 Hz, 2H), 2.30 (s, 3H), 1.20 (t, *J* = 7.1 Hz, 3H). **^{13}C NMR** (126 MHz, CDCl$_3$): δ (ppm) 167.1 (d, *J* = 16.4 Hz), 163.4 (d, *J* = 261.4 Hz), 139.9, 136.2 (d, *J* = 7.7 Hz), 133.1, 133.0, 130.1 (d, *J* = 2.8 Hz), 129.7, 128.0, 127.8, 127.8, 117.0 (d, *J* = 19.4 Hz), 115.1 (d, *J* = 21.4 Hz), 61.3, 21.6, 14.3. **^{19}F NMR** (471 MHz, CDCl$_3$): δ (ppm) -105.15 (d, *J* = 27.7 Hz, 1F). **HRMS** (ESI) m/z: $[M+Na]^+$ Calcd for $C_{20}H_{19}FO_2Na$ 333.1261; Found 333.1257.

ethyl (2*E*,4*E*)-3-fluoro-5-(4-methoxyphenyl)-2-phenylpenta-2,4-dienoate (3b)

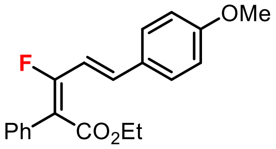

Following the general procedure (III), reaction was run using *gem*-difluoroalkene **1a** (42.4 mg, 0.2 mmol), 4-methoxystyrene (80.5 mg, 0.6 mmol), Pd(dba)$_2$ (11.6 mg, 0.02 mmol), dppb (8.6 mg, 0.02 mmol), dry NaI (90 mg, 0.6 mmol) and Et$_3$N (60.7 mg, 83.6 μL, 0.6 mmol) in 0.3 mL toluene in a 90 °C oil bath for 18 h ((*E*,*E*)/(*Z*,*E*) > 99:1). The product was purified by flash column chromatography on silica gel (ethyl acetate : hexane = 1 : 50) and obtained as a colorless solid (48.3 mg, 74% yield, (*E*,*E*)/(*Z*,*E*) > 99:1), R_f = 0.13 (ethyl acetate : hexane = 1 : 100). **^{1}H NMR** (500 MHz, CDCl$_3$): δ (ppm) 7.69 (dd, *J* = 27.7, 16.0 Hz, 1H), 7.51 (d, *J* = 8.8 Hz, 2H), 7.41 – 7.32 (m, 5H), 7.21 (d, *J* = 16.0 Hz, 1H), 6.92 (d, *J* = 8.8 Hz, 2H), 4.28 (q, *J* = 7.2 Hz, 2H), 3.85 (s, 3H), 1.29 (t, *J* = 7.1 Hz, 3H). **^{13}C NMR** (126 MHz, CDCl$_3$): δ (ppm) 167.2 (d, *J* = 16.5 Hz), 163.7 (d, *J* = 260.9 Hz), 160.9, 135.9 (d, *J* = 8.1 Hz), 133.2 (d, *J* = 1.8 Hz), 130.1 (d, *J* = 2.7 Hz), 129.4, 128.5, 128.0, 127.7, 115.8 (d, *J* = 19.3 Hz), 114.5 (d, *J* = 21.7 Hz), 114.4, 61.2, 55.5, 14.3. **^{19}F NMR** (471 MHz, CDCl$_3$): δ (ppm) -104.74 (d, *J* = 27.7 Hz, 1F). **HRMS** (ESI) m/z: $[M+Na]^+$ Calcd for $C_{20}H_{19}FO_3Na$ 349.1210; Found 349.1208.

ethyl (2*E*,4*E*)-3-fluoro-5-(3-methoxyphenyl)-2-phenylpenta-2,4-dienoate (3c)

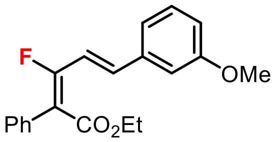

Following the general procedure (III), reaction was run using *gem*-difluoroalkene **1a** (42.4 mg, 0.2 mmol), 3-methoxystyrene (80.5 mg, 0.6 mmol), Pd(dba)$_2$ (11.6 mg, 0.02 mmol), dppb (8.6 mg, 0.02 mmol), dry NaI (90 mg, 0.6 mmol) and Et$_3$N (60.7 mg, 83.6 μL, 0.6 mmol) in 0.3 mL toluene in a 90 °C oil bath for 18 h ((*E*,*E*)/(*Z*,*E*) = 99:1). The product was purified by flash column chromatography on silica gel (ethyl acetate : hexane = 1 : 50) and obtained as a colorless solid (46.9 mg, 72% yield, (*E*,*E*)/(*Z*,*E*) = 99:1), R_f = 0.12 (ethyl acetate : hexane = 1 : 100). **^{1}H NMR** (500 MHz, CDCl$_3$): δ (ppm) 7.67 (dd, *J* = 27.4, 16.0 Hz, 1H), 7.33 – 7.07 (m, 8H), 6.98 (s, 1H), 6.82 (d, *J* = 8.1 Hz, 1H), 4.20 (q, *J* = 7.1 Hz, 2H), 3.77 (s, 3H), 1.21 (t, *J* = 7.1 Hz, 3H). **^{13}C NMR** (126 MHz, CDCl$_3$): δ (ppm) 167.0 (d, *J* = 16.3 Hz), 162.9 (d, *J* = 261.4 Hz), 160.0, 137.1, 136.0 (d, *J* = 7.5 Hz), 132.9, 130.0 (d, *J* = 2.8 Hz), 129.9, 128.1, 127.9, 120.5, 118.3 (d, *J* = 19.7 Hz), 115.8 (d, *J* = 21.3 Hz), 115.3, 112.9, 61.3, 55.4, 14.3. **^{19}F NMR** (471 MHz, CDCl$_3$): δ (ppm) -105.53 (d, *J* = 27.3 Hz, 1F). **HRMS** (ESI) m/z: $[M+Na]^+$ Calcd for $C_{20}H_{19}FO_3Na$ 349.1210; Found 349.1207.

ethyl (2*E*,4*E*)-5-(4-(*tert*-butyl)phenyl)-3-fluoro-2-phenylpenta-2,4-dienoate (3d)

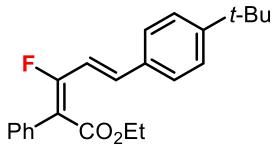

Following the general procedure (III), reaction was run using *gem*-difluoroalkene **1a** (42.4 mg, 0.2 mmol), 4-*t*-butylstyrene (96.2 mg, 0.6 mmol), Pd(dba)$_2$ (11.6 mg, 0.02 mmol), dppb (8.6 mg, 0.02 mmol), dry NaI (90 mg, 0.6 mmol) and Et$_3$N (60.7 mg, 83.6 μL, 0.6 mmol) in 0.3 mL toluene in a 90 ºC oil bath for 18 h ((*E*,*E*)/(*Z*,*E*) > 99:1). The product was purified by flash column chromatography on silica gel (ethyl acetate : hexane = 1 : 100) and obtained as a colorless oil (56.3 mg, 80% yield, (*E*,*E*)/(*Z*,*E*) > 99:1), R_f = 0.19 (ethyl acetate : hexane = 1 : 200). **^{1}H NMR** (500 MHz, CDCl$_3$): δ (ppm) 7.74 (dd, *J* = 27.6, 16.0 Hz, 1H), 7.49 (d, *J* = 8.4 Hz, 2H), 7.42 – 7.32 (m, 7H), 7.23 (d, *J* = 16.1 Hz, 1H), 4.28 (q, *J* = 7.1 Hz, 2H), 1.34 (s, 9H), 1.29 (t, *J* = 7.1 Hz, 3H). **^{13}C NMR** (126 MHz, CDCl$_3$): δ (ppm) 167.1 (d, *J* = 16.4 Hz), 163.3 (d, *J* = 261.2 Hz), 153.0, 136.0 (d, *J* = 7.7 Hz), 133.1 (d, *J* = 1.7 Hz), 133.0, 130.1 (d, *J* = 2.7 Hz), 128.1, 127.8, 127.7, 126.0, 117.2 (d, *J* = 19.5 Hz), 115.1 (d, *J* = 21.3 Hz), 61.3, 35.0, 31.3, 14.4. **^{19}F NMR** (471 MHz, CDCl$_3$): δ (ppm) -105.16 (d, *J* = 27.6 Hz, 1F). **HRMS** (ESI) m/z: [M+Na]$^+$ Calcd for $C_{23}H_{25}FO_2Na$ 375.1731; Found 375.1726.

ethyl (2*E*,4*E*)-5-(4-acetoxyphenyl)-3-fluoro-2-phenylpenta-2,4-dienoate (3e)

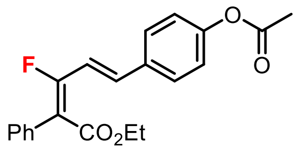

Following the general procedure (III), reaction was run using *gem*-difluoroalkene **1a** (42.4 mg, 0.2 mmol), 4-acetoxystyrene (97.3 mg, 0.6 mmol), Pd(dba)$_2$ (11.6 mg, 0.02 mmol), dppb (8.6 mg, 0.02 mmol), dry NaI (90 mg, 0.6 mmol) and Et$_3$N (60.7 mg, 83.6 μL, 0.6 mmol) in 0.3 mL toluene in a 90 ºC oil bath for 18 h ((*E*,*E*)/(*Z*,*E*) = 96:4). The product was purified by flash column chromatography on silica gel (ethyl acetate : hexane = 1 : 100) and obtained as a colorless oil (35.4 mg, 50% yield, (*E*,*E*)/(*Z*,*E*) = 99:1), R_f = 0.14 (ethyl acetate : hexane = 1 : 200). **^{1}H NMR** (500 MHz, CDCl$_3$): δ (ppm) 7.74 (dd, *J* = 27.3, 16.0 Hz, 1H), 7.56 (d, *J* = 8.7 Hz, 2H), 7.39 (d, *J* = 7.1 Hz, 2H), 7.34 (t, *J* = 6.1 Hz, 3H), 7.21 (d, *J* = 16.1 Hz, 1H), 7.12 (d, *J* = 8.6 Hz, 2H), 4.28 (q, *J* = 7.1 Hz, 2H), 2.32 (s, 3H), 1.28 (t, *J* = 7.1 Hz, 3H).**^{13}C NMR** (126 MHz, CDCl$_3$): δ (ppm) 169.4, 167.0 (d, *J* = 16.2 Hz), 162.9 (d, *J* = 261.2 Hz), 151.5, 134.9 (d, *J* = 7.6 Hz), 133.5, 132.9, 130.0 (d, *J* = 2.7 Hz), 128.9, 128.1, 127.9, 122.2, 118.2 (d, *J* = 19.6 Hz), 115.8 (d, *J* = 21.2 Hz), 61.4, 21.3, 14.3. **^{19}F NMR** (471 MHz, CDCl$_3$): δ (ppm) -105.55 (d, *J* = 27.4 Hz, 1F). **HRMS** (ESI) m/z: [M+Na]$^+$ Calcd for $C_{21}H_{19}FO_4Na$ 377.1160; Found 377.1155.

ethyl (2*E*,4*E*)-3-fluoro-5-(4-fluorophenyl)-2-phenylpenta-2,4-dienoate (3f)

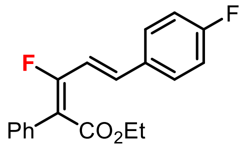

Following the general procedure (III), reaction was run using *gem*-difluoroalkene **1a** (42.4 mg, 0.2 mmol), 4-fluorostyrenee (73.3 mg, 0.6 mmol), Pd(dba)$_2$ (11.6 mg, 0.02 mmol), dppb (8.6 mg, 0.02 mmol), dry NaI (90 mg, 0.6 mmol) and Et$_3$N (60.7 mg, 83.6 μL, 0.6 mmol) in 0.3 mL toluene in a 90 ºC oil bath for 18 h ((*E*,*E*)/(*Z*,*E*) = 99:1). The product was purified by flash column chromatography on silica gel (ethyl acetate : hexane = 1 : 100) and obtained as a colorless oil (55.3 mg, 88% yield, (*E*,*E*)/(*Z*,*E*) > 99:1), R_f = 0.13 (ethyl acetate : hexane = 1 : 200). **^{1}H NMR** (500 MHz, CDCl$_3$): δ (ppm) 7.72 (dd, *J* = 27.3, 16.1 Hz, 1H), 7.53 (dd, *J* = 8.6, 5.5 Hz, 2H), 7.41 – 7.32 (m, 5H), 7.20 (d, *J* = 16.1 Hz, 1H), 7.08 (t, *J* = 8.6 Hz, 2H), 4.28 (q, *J* = 7.1 Hz, 2H), 1.28 (t, *J* = 7.1 Hz, 3H). **^{13}C NMR** (126 MHz, CDCl$_3$): δ (ppm) 167.0 (d, *J* = 16.3 Hz), 163.5 (d, *J* = 250.5 Hz), 163.0 (d, *J* = 261.5 Hz), 134.8 (d, *J* = 7.9 Hz), 132.9 (d, *J* = 1.8 Hz), 132.0 (d, *J* = 3.5 Hz), 130.1 (d, *J* = 2.9 Hz), 129.5 (d, *J* = 8.2 Hz), 128.1, 127.9, 117.8 (dd, *J* = 19.6, 2.5 Hz), 116.1 (d, *J* = 21.9 Hz), 115.6 (d, *J* = 21.4 Hz), 61.4, 14.3. **^{19}F NMR** (471 MHz, CDCl$_3$): δ (ppm) -105.51 (d, *J* = 27.1 Hz, 1F), -113.19 – -113.24 (m, 1F). **HRMS** (ESI) m/z: [M+Na]$^+$ Calcd for $C_{19}H_{16}F_2O_2Na$ 337.1011; Found 337.1009.

ethyl (2*E*,4*E*)-5-(4-chlorophenyl)-3-fluoro-2-phenylpenta-2,4-dienoate (3g)

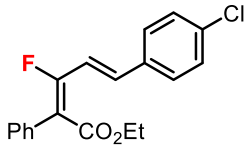

Following the general procedure (III), reaction was run using *gem*-difluoroalkene **1a** (42.4 mg, 0.2 mmol), 4-chlorostyrene (83.1 mg, 0.6 mmol), Pd(dba)$_2$ (11.6 mg, 0.02 mmol), dppb (8.6 mg, 0.02 mmol), dry NaI (90 mg, 0.6 mmol) and Et$_3$N (60.7 mg, 83.6 μL, 0.6 mmol) in 0.3 mL toluene in a 90 ºC oil bath for 18 h ((*E*,*E*)/(*Z*,*E*) = 99:1). The product was purified by flash column chromatography on silica gel (ethyl acetate : hexane = 1 : 100) and obtained as a colorless oil (41.6 mg, 63% yield, (*E*,*E*)/(*Z*,*E*) = 98:2), R_f = 0.15 (ethyl acetate : hexane = 1 : 200). **^{1}H NMR** (400 MHz, CDCl$_3$): δ (ppm) 7.77 (dd, *J* = 27.2, 16.1 Hz, 1H), 7.48 (d, *J* = 8.5 Hz, 2H), 7.42 – 7.33 (m, 7H), 7.18 (d, *J* = 16.0 Hz, 1H), 4.28 (q, *J* = 7.1 Hz, 2H), 1.29 (t, *J* = 7.1 Hz, 3H). **^{13}C NMR** (101 MHz, CDCl$_3$): δ (ppm) 167.0 (d, *J* = 16.3 Hz), 162.8 (d, *J* = 261.4 Hz), 135.3, 134.6 (d, *J* = 7.7 Hz), 134.3, 132.8 (d, *J* = 1.7 Hz), 130.0 (d, *J* = 2.9 Hz), 129.2, 129.0, 128.1, 128.0, 118.6 (d, *J* = 19.7 Hz), 116.1 (d, *J* = 21.2 Hz), 61.4, 14.3. **^{19}F NMR** (471 MHz, CDCl$_3$): δ (ppm) -105.73 (d, *J* = 27.4 Hz, 1F). **HRMS** (ESI) m/z: $[M+Na]^+$ Calcd for $C_{19}H_{16}ClFO_2Na$ 353.0715; Found 353.0713.

ethyl (2*E*,4*E*)-5-(4-bromophenyl)-3-fluoro-2-phenylpenta-2,4-dienoate (3h)

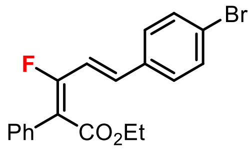

Following the general procedure (III), reaction was run using *gem*-difluoroalkene **1a** (42.4 mg, 0.2 mmol), 4-bromostyrene (109.8 mg, 0.6 mmol), Pd(dba)$_2$ (11.6 mg, 0.02 mmol), dppb (8.6 mg, 0.02 mmol), dry NaI (90 mg, 0.6 mmol) and Et$_3$N (60.7 mg, 83.6 μL, 0.6 mmol) in 0.3 mL toluene in a 90 ºC oil bath for 18 h ((*E*,*E*)/(*Z*,*E*) = 99:1). The product was purified by flash column chromatography on silica gel (ethyl acetate : hexane = 1 : 100) and obtained as a colorless oil (41.9 mg, 56% yield, (*E*,*E*)/(*Z*,*E*) > 99:1), R_f = 0.18 (ethyl acetate : hexane = 1 : 200). **^{1}H NMR** (500 MHz, CDCl$_3$): δ (ppm) 7.77 (dd, *J* = 27.2, 16.1 Hz, 1H), 7.51 (d, *J* = 8.5 Hz, 2H), 7.41 – 7.32 (m, 7H), 7.16 (d, *J* = 16.0 Hz, 1H), 4.27 (q, *J* = 7.1 Hz, 2H), 1.28 (t, *J* = 7.1 Hz, 3H). **^{13}C NMR** (126 MHz, CDCl$_3$): δ (ppm) 167.0 (d, *J* = 16.3 Hz), 162.7 (d, *J* = 261.4 Hz), 134.7, 134.6, 132.8 (d, *J* = 1.3 Hz), 132.2, 130.0 (d, *J* = 2.8 Hz), 129.2, 128.1, 128.0, 123.6, 118.6 (d, *J* = 19.8 Hz), 116.1 (d, *J* = 21.3 Hz), 61.4, 14.3. **^{19}F NMR** (471 MHz, CDCl$_3$): δ (ppm) -105.77 (d, *J* = 27.1 Hz, 1F). **HRMS** (ESI) m/z: $[M+Na]^+$ Calcd for $C_{19}H_{16}BrFO_2Na$ 397.0210; Found 397.0208.

ethyl (2*E*,4*E*)-5-(2-bromophenyl)-3-fluoro-2-phenylpenta-2,4-dienoate (3i)

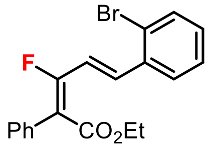

Following the general procedure (III), reaction was run using *gem*-difluoroalkene **1a** (42.4 mg, 0.2 mmol), 2-bromostyrene (109.8 mg, 0.6 mmol), Pd(dba)$_2$ (11.6 mg, 0.02 mmol), dppb (8.6 mg, 0.02 mmol), dry NaI (90 mg, 0.6 mmol) and Et$_3$N (60.7 mg, 83.6 μL, 0.6 mmol) in 0.3 mL toluene in a 90 ºC oil bath for 18 h ((*E*,*E*)/(*Z*,*E*) = 93:7). The product was purified by flash column chromatography on silica gel (ethyl acetate : hexane = 1 : 100) and obtained as a colorless oil (20.9 mg, 28% yield, (*E*,*E*)/(*Z*,*E*) > 99:1), R_f = 0.22 (ethyl acetate : hexane = 1 : 200). **^{1}H NMR** (500 MHz, CDCl$_3$): δ (ppm) 7.76 – 7.67 (m, 2H), 7.62 – 7.59 (m, 2H), 7.41 – 7.32 (m, 6H), 7.19 (td, *J* = 7.7, 1.6 Hz, 1H), 4.28 (q, *J* = 7.1 Hz, 2H), 1.28 (t, *J* = 7.1 Hz, 3H). **^{13}C NMR** (126 MHz, CDCl$_3$): δ (ppm) 167.0 (d, *J* = 16.0 Hz), 162.5 (d, *J* = 262.4 Hz), 135.7, 134.5 (d, *J* = 8.0 Hz), 133.5, 132.7 (d, *J* = 1.2 Hz), 130.5, 130.0 (d, *J* = 3.0 Hz), 128.1, 128.0, 127.8, 127.6, 125.2, 120.5 (d, *J* = 19.7 Hz), 116.6 (d, *J* = 20.9 Hz), 61.5, 14.3. **^{19}F NMR** (471 MHz, CDCl$_3$): δ (ppm) -105.65 (d, *J* = 26.6 Hz, 1F). **HRMS** (ESI) m/z: $[M+Na]^+$ Calcd for $C_{19}H_{16}BrFO_2Na$ 397.0210; Found 397.0205.

ethyl (2*E*,4*E*)-3-fluoro-2-phenyl-5-(4-(trifluoromethyl)phenyl)penta-2,4-dienoate (3j)

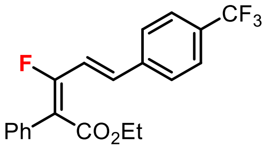

Following the general procedure (III), reaction was run using *gem*-difluoroalkene **1a** (42.4 mg, 0.2 mmol), 4-(trifluoromethyl)styrene (103.3 mg, 0.6 mmol), Pd(dba)$_2$ (11.6 mg, 0.02 mmol), dppb (8.6 mg, 0.02 mmol), dry NaI (90 mg, 0.6 mmol) and Et$_3$N (60.7 mg, 83.6 μL, 0.6 mmol) in 0.3 mL toluene in a 90 ^{o}C oil bath for 18 h ((*E*,*E*)/(*Z*,*E*) = 96:4). The product was purified by flash column chromatography on silica gel (ethyl acetate : hexane = 1 : 100) and obtained as a colorless oil (50.2 mg, 69% yield, (*E*,*E*)/(*Z*,*E*) > 99:1), R_f = 0.20 (ethyl acetate : hexane = 1 : 200). **^{1}H NMR** (500 MHz, CDCl$_3$): δ (ppm) 7.75 (dd, *J* = 27.0, 16.0 Hz, 1H), 7.53 (s, 4H), 7.32 – 7.24 (m, 5H), 7.14 (d, *J* = 16.1 Hz, 1H), 4.20 (q, *J* = 7.1 Hz, 2H), 1.19 (t, *J* = 7.1 Hz, 3H). **^{13}C NMR** (101 MHz, CDCl$_3$): δ (ppm) 166.9 (d, *J* = 16.0 Hz), 162.2 (d, *J* = 261.6 Hz), 139.2, 134.1 (d, *J* = 7.5 Hz), 132.6 (d, *J* = 1.5 Hz), 130.9 (q, *J* = 32.6 Hz), 130.0 (d, *J* = 3.0 Hz), 128.1, 128.1, 127.9, 125.9 (q, *J* = 3.8 Hz), 124.1 (q, *J* = 272.0 Hz), 120.4 (d, *J* = 20.1 Hz), 117.1 (d, *J* = 20.9 Hz), 61.5, 14.3. **^{19}F NMR** (471 MHz, CDCl$_3$): δ (ppm) -64.93 (s, 3F), -106.52 (d, *J* = 26.9 Hz, 1F). **HRMS** (ESI) m/z: [M+Na]$^+$ Calcd for $C_{20}H_{16}F_4O_2Na$ 387.0979; Found 387.0974.

ethyl (2*E*,4*E*)-5-(4-cyanophenyl)-3-fluoro-2-phenylpenta-2,4-dienoate (3k)

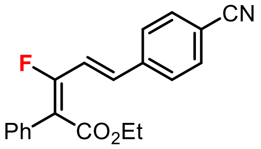

Following the general procedure (III), reaction was run using *gem*-difluoroalkene **1a** (42.4 mg, 0.2 mmol), 4-cyanostyrene (77.5 mg, 0.6 mmol), Pd(dba)$_2$ (11.6 mg, 0.02 mmol), dppb (8.6 mg, 0.02 mmol), dry NaI (90 mg, 0.6 mmol) and Et$_3$N (60.7 mg, 83.6 μL, 0.6 mmol) in 0.3 mL toluene in a 90 ^{o}C oil bath for 18 h ((*E*,*E*)/(*Z*,*E*) = 97:3). The product was purified by flash column chromatography on silica gel (ethyl acetate : hexane = 1 : 50) and obtained as a colorless solid (34 mg, 53% yield, (*E*,*E*)/(*Z*,*E*) > 99:1), R_f = 0.23 (ethyl acetate : hexane = 1 : 100). **^{1}H NMR** (400 MHz, CDCl$_3$): δ (ppm) 7.85 (dd, *J* = 26.8, 16.0 Hz, 1H), 7.64 (q, *J* = 12.1 Hz, 4H), 7.42 – 7.32 (m, 5H), 7.19 (d, *J* = 16.1 Hz, 1H), 4.29 (q, *J* = 7.1 Hz, 2H), 1.28 (t, *J* = 7.1 Hz, 3H). **^{13}C NMR** (101 MHz, CDCl$_3$): 166.8 (d, *J* = 15.9 Hz), 161.9 (d, *J* = 261.6 Hz), 140.1, 133.5 (d, *J* = 7.4 Hz), 132.7, 132.4, 129.9 (d, *J* = 3.0 Hz), 128.2, 128.2, 128.1, 121.4 (d, *J* = 20.2 Hz), 118.8, 117.7 (d, *J* = 20.7 Hz), 112.4, 61.6, 14.3. **^{19}F NMR** (471 MHz, CDCl$_3$): δ (ppm) -106.71 (d, *J* = 26.8 Hz, 1F). **HRMS** (ESI) m/z: [M+Na]$^+$ Calcd for $C_{20}H_{16}FNO_2Na$ 344.1057; Found 344.1056.

methyl 4-((1*E*,3*E*)-5-ethoxy-3-fluoro-5-oxo-4-phenylpenta-1,3-dien-1-yl)benzoate (3l)

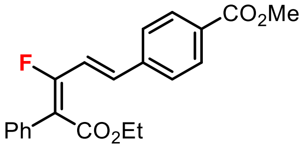

Following the general procedure (III), reaction was run using *gem*-difluoroalkene **1a** (42.4 mg, 0.2 mmol), 4-styrenecarboxylic acid methyl ester (97.3 mg, 0.6 mmol), Pd(dba)$_2$ (11.6 mg, 0.02 mmol), dppb (8.6 mg, 0.02 mmol), dry NaI (90 mg, 0.6 mmol) and Et$_3$N (60.7 mg, 83.6 μL, 0.6 mmol) in 0.3 mL toluene in a 90 ^{o}C oil bath for 18 h ((*E*,*E*)/(*Z*,*E*) = 99:1). The product was purified by flash column chromatography on silica gel (ethyl acetate : hexane = 1 : 50) and obtained as a colorless solid (36.8 mg, 52% yield, (*E*,*E*)/(*Z*,*E*) = 99:1), R_f = 0.26 (ethyl acetate : hexane = 1 : 100). **^{1}H NMR** (400 MHz, CDCl$_3$): δ (ppm) 7.96 (d, *J* = 8.5 Hz, 2H), 7.77 (dd, *J* = 27.1, 16.1 Hz, 1H), 7.51 (d, *J* = 8.4 Hz, 2H), 7.34 – 7.24 (m, 5H), 7.16 (d, *J* = 16.7 Hz, 1H), 4.21 (q, *J* = 7.1 Hz, 2H), 3.85 (s, 3H), 1.20 (t, *J* = 7.1 Hz, 3H). **^{13}C NMR** (101 MHz, CDCl$_3$): δ (ppm) 166.9 (d, *J* = 16.1 Hz), 166.7, 162.4 (d, *J* = 261.9 Hz), 140.1, 134.6 (d, *J* = 7.6 Hz), 132.7, 130.6, 130.2, 130.0 (d, *J* = 2.9 Hz), 128.1, 128.1, 127.6, 120.3 (d, *J* = 20.0 Hz), 116.9 (d, *J* = 21.0 Hz), 61.5, 52.3, 14.3. **^{19}F NMR** (471 MHz, CDCl$_3$): δ (ppm) -106.26 (d, *J* = 26.9 Hz, 1F). **HRMS** (ESI) m/z: [M+Na]$^+$ Calcd for $C_{21}H_{19}FO_4Na$ 377.1160; Found 377.1156.

ethyl (2*E*,4*E*)-5-([1,1'-biphenyl]-4-yl)-3-fluoro-2-phenylpenta-2,4-dienoate (3m)

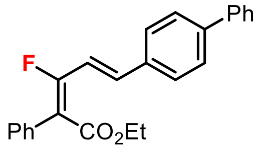

Following the general procedure (III), reaction was run using *gem*-difluoroalkene **1a** (42.4 mg, 0.2 mmol), 4-vinylbiphenyl (108.2 mg, 0.6 mmol), Pd(dba)$_2$ (11.6 mg, 0.02 mmol), dppb (8.6 mg, 0.02 mmol), dry NaI (90 mg, 0.6 mmol) and Et$_3$N (60.7 mg, 83.6 μL, 0.6 mmol) in 0.3 mL toluene in a 90 ºC oil bath for 18 h ((*E*,*E*)/(*Z*,*E*) = 99:1). The product was purified by flash column chromatography on silica gel (ethyl acetate : hexane = 1 : 100) and obtained as a colorless solid (62.5 mg, 84% yield, (*E*,*E*)/(*Z*,*E*) = 99:1), R_f = 0.23 (ethyl acetate : hexane = 1 : 200). **^{1}H NMR** (500 MHz, CDCl$_3$): δ (ppm) 7.86 (dd, *J* = 27.5, 16.0 Hz, 1H), 7.66 – 7.65 (m, 6H), 7.48 (t, *J* = 7.6 Hz, 2H), 7.44 – 7.36 (m, 6H), 7.30 (d, *J* = 16.1 Hz, 1H), 4.31 (q, *J* = 7.1 Hz, 2H), 1.32 (t, *J* = 7.1 Hz, 3H). **^{13}C NMR** (126 MHz, CDCl$_3$): δ (ppm) 167.1 (d, *J* = 16.4 Hz), 163.1 (d, *J* = 261.4 Hz), 142.2, 140.4, 135.6 (d, *J* = 7.7 Hz), 134.8, 133.0, 130.1 (d, *J* = 2.7 Hz), 129.0, 128.3, 128.1, 127.9, 127.8, 127.6, 127.1, 117.9 (d, *J* = 19.7 Hz), 115.6 (d, *J* = 21.1 Hz), 61.3, 14.3. **^{19}F NMR** (471 MHz, CDCl$_3$): δ (ppm) -105.40 (d, *J* = 27.6 Hz, 1F). **HRMS** (ESI) m/z: [M+Na]$^+$ Calcd for $C_{25}H_{21}FO_2Na$ 395.1418; Found 395.1413.

ethyl (2*E*,4*E*)-5-(3,4-dimethoxyphenyl)-3-fluoro-2-phenylpenta-2,4-dienoate (3n)

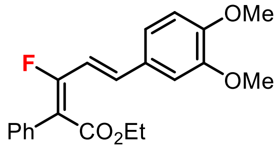

Following the general procedure (III), reaction was run using *gem*-difluoroalkene **1a** (42.4 mg, 0.2 mmol), 3,4-dimethoxystyrene (98.5 mg, 0.6 mmol), Pd(dba)$_2$ (11.6 mg, 0.02 mmol), dppb (8.6 mg, 0.02 mmol), dry NaI (90 mg, 0.6 mmol) and Et$_3$N (60.7 mg, 83.6 μL, 0.6 mmol) in 0.3 mL toluene in a 90 ºC oil bath for 18 h ((*E*,*E*)/(*Z*,*E*) = 98:2). The product was purified by flash column chromatography on silica gel (ethyl acetate : hexane = 1 : 50) and obtained as a yellow solid (44.1 mg, 62% yield, (*E*,*E*)/(*Z*,*E*) = 97:3), R_f = 0.10 (ethyl acetate : hexane = 1 : 100). **^{1}H NMR** (500 MHz, CDCl$_3$): δ (ppm) 7.68 (dd, *J* = 27.5, 16.0 Hz, 1H), 7.40 – 7.31 (m, 5H), 7.19 (d, *J* = 16.0 Hz, 1H), 7.12 (dd, *J* = 8.3, 2.0 Hz, 1H), 7.07 (d, *J* = 2.0 Hz, 1H), 6.87 (d, *J* = 8.4 Hz, 1H), 4.27 (q, *J* = 7.1 Hz, 2H), 3.94 (s, 3H), 3.92 (s, 3H), 1.28 (t, *J* = 7.1 Hz, 3H). **^{13}C NMR** (126 MHz, CDCl$_3$): δ (ppm) 167.1 (d, *J* = 16.7 Hz), 163.6 (d, *J* = 261.1 Hz), 150.6, 149.3, 136.2 (d, *J* = 8.0 Hz), 133.2 (d, *J* = 1.8 Hz), 130.1 (d, *J* = 2.7 Hz), 128.8, 128.0, 127.7, 122.0, 116.0 (d, *J* = 19.2 Hz), 114.5 (d, *J* = 21.6 Hz), 111.2, 109.7, 61.2, 56.1, 56.0, 14.3. **^{19}F NMR** (471 MHz, CDCl$_3$): δ (ppm) -104.56 (d, *J* = 27.5 Hz, 1F). **HRMS** (ESI) m/z: [M+Na]$^+$ Calcd for $C_{21}H_{21}FO_4Na$ 379.1316; Found 379.1313.

ethyl (2*E*,4*E*)-3-fluoro-2-phenyl-5-(thiophen-2-yl)penta-2,4-dienoate (3o)

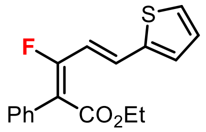

Following the general procedure (III), reaction was run using *gem*-difluoroalkene **1a** (42.4 mg, 0.2 mmol), 2-vinylthiophene (66.1 mg, 0.6 mmol), Pd(dba)$_2$ (11.6 mg, 0.02 mmol), dppb (8.6 mg, 0.02 mmol), dry NaI (90 mg, 0.6 mmol) and Et$_3$N (60.7 mg, 83.6 μL, 0.6 mmol) in 0.3 mL toluene in a 90 ºC oil bath for 18 h ((*E*,*E*)/(*Z*,*E*) > 99:1). The product was purified by flash column chromatography on silica gel (ethyl acetate : hexane = 1 : 100) and obtained as a colorless oil (30.8 mg, 51% yield, (*E*,*E*)/(*Z*,*E*) = 98:2), R_f = 0.26 (ethyl acetate : hexane = 1 : 200). **^{1}H NMR** (500 MHz, CDCl$_3$): δ (ppm) 7.59 (dd, *J* = 27.4, 15.8 Hz, 1H), 7.41 – 7.32 (m, 7H), 7.20 (d, *J* = 3.6 Hz, 1H), 7.05 (dd, *J* = 5.1, 3.6 Hz, 1H), 4.29 (q, *J* = 7.1 Hz, 2H), 1.31 (t, *J* = 7.1 Hz, 3H). **^{13}C NMR** (101 MHz, CDCl$_3$): δ (ppm) 166.9 (d, *J* = 16.2 Hz), 162.7 (d, *J* = 260.7 Hz), 141.4, 133.0 (d, *J* = 1.7 Hz), 130.1 (d, *J* = 2.9 Hz), 129.5, 128.9 (d, *J* = 8.8 Hz), 128.2, 128.0, 127.8, 127.6, 117.3 (d, *J* = 19.9 Hz), 115.2 (d, *J* = 20.9 Hz), 61.3, 14.3. **^{19}F NMR** (471 MHz, CDCl$_3$): δ (ppm) -105.64 (d, *J* = 27.6 Hz, 1F). **HRMS** (ESI) m/z: [M+Na]$^+$ Calcd for $C_{17}H_{15}FO_2SNa$ 325.0669; Found 325.0665.

ethyl (2*E*,4*E*,6*E*)-3-fluoro-2,7-diphenylhepta-2,4,6-trienoate (3p)

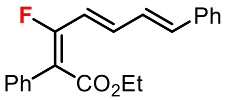

Following the general procedure (III), reaction was run using *gem*-difluoroalkene **1a** (42.4 mg, 0.2 mmol), 1-phenyl-1,3-butadiene (78.1 mg, 0.6 mmol), $Pd(dba)_2$ (11.6 mg, 0.02 mmol), dppb (8.6 mg, 0.02 mmol), dry NaI (90 mg, 0.6 mmol) and Et_3N (60.7 mg, 83.6 μL, 0.6 mmol) in 0.3 mL toluene in a 90 ºC oil bath for 18 h ((*E*,*E*)/(*Z*,*E*) = 83:17). The product was purified by flash column chromatography on silica gel (ethyl acetate : hexane = 1 : 100) and obtained as a colorless oil (32.2 mg, 50% yield, (*E*,*E*)/(*Z*,*E*) = 97:3), R_f = 0.30 (ethyl acetate : hexane = 1 : 200). **^{1}H NMR** (500 MHz, $CDCl_3$): δ (ppm) 7.39 (d, *J* = 7.3 Hz, 2H), 7.32 – 7.17 (m, 9H), 7.00 – 6.88 (m, 2H), 6.74 (d, *J* = 15.0 Hz, 1H), 4.19 (q, *J* = 7.1 Hz, 2H), 1.19 (t, *J* = 7.1 Hz, 3H). **^{13}C NMR** (126 MHz, $CDCl_3$): δ (ppm) 167.1 (d, *J* = 16.4 Hz), 162.9 (d, *J* = 260.2 Hz), 138.2, 136.7, 136.6, 133.0, 130.1 (d, *J* = 3.0 Hz), 128.9, 128.8, 128.1, 128.0, 127.8, 127.1, 121.5 (d, *J* = 20.1 Hz), 115.3 (d, *J* = 21.3 Hz), 61.3, 14.3. **^{19}F NMR** (471 MHz, $CDCl_3$): δ (ppm) -105.57 (d, *J* = 28.2 Hz, 1F). **HRMS** (ESI) m/z: $[M+Na]^+$ Calcd for $C_{21}H_{19}FO_2$ 345.1261; Found 345.1259.

6-butyl 1-ethyl (2*E*,4*E*)-3-fluoro-2-phenylhexa-2,4-dienedioate (3q)

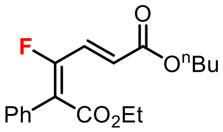

Following the general procedure (III), reaction was run using *gem*-difluoroalkene **1a** (42.4 mg, 0.2 mmol), butyl acrylate (76.9 mg, 0.6 mmol), $Pd(dba)_2$ (11.6 mg, 0.02 mmol), dppb (8.6 mg, 0.02 mmol), dry NaI (90 mg, 0.6 mmol) and Et_3N (60.7 mg, 83.6 μL, 0.6 mmol) in 0.3 mL toluene in a 90 ºC oil bath for 18 h ((*E*,*E*)/(*Z*,*E*) = 95:5). The product was purified by flash column chromatography on silica gel (ethyl acetate : hexane = 1 : 50) and obtained as a colorless oil (39.0 mg, 61% yield, (*E*,*E*)/(*Z*,*E*) = 95:5), R_f = 0.25 (ethyl acetate : hexane = 1 : 100). **^{1}H NMR** (500 MHz, $CDCl_3$): δ (ppm) 7.88 (dd, *J* = 28.1, 15.6 Hz, 1H), 7.41 – 7.34 (m, 5H), 6.43 (d, *J* = 15.6 Hz, 1H), 4.32 (q, *J* = 7.1 Hz, 2H), 4.21 (t, *J* = 6.7 Hz, 2H), 1.71 – 1.64 (m, 2H), 1.46 – 1.39 (m, 2H), 1.32 (t, *J* = 7.2 Hz, 3H), 0.96 (t, *J* = 7.4 Hz, 3H). **^{13}C NMR** (126 MHz, $CDCl_3$): δ (ppm) 166.2 (d, *J* = 14.9 Hz), 166.0, 158.7 (d, *J* = 262.1 Hz), 132.7 (d, *J* = 21.8 Hz), 131.7, 129.6 (d, *J* = 3.9 Hz), 128.8, 128.4, 124.2 (d, *J* = 5.3 Hz), 121.9 (d, *J* = 18.6 Hz), 65.1, 62.1, 30.8, 19.3, 14.2, 13.8. **^{19}F NMR** (471 MHz, $CDCl_3$): δ (ppm) -110.39 (d, *J* = 28.0 Hz, 1F). **HRMS** (ESI) m/z: $[M+Na]^+$ Calcd for $C_{18}H_{21}FO_4Na$ 343.1316; Found 343.1313.

6-benzyl 1-ethyl (2*E*,4*E*)-3-fluoro-2-phenylhexa-2,4-dienedioate (3r)

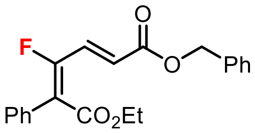

Following the general procedure (III), reaction was run using *gem*-difluoroalkene **1a** (42.4 mg, 0.2 mmol), benzyl acrylate (97.3 mg, 0.6 mmol), $Pd(dba)_2$ (11.6 mg, 0.02 mmol), dppb (8.6 mg, 0.02 mmol), dry NaI (90 mg, 0.6 mmol) and Et_3N (60.7 mg, 83.6 μL, 0.6 mmol) in 0.3 mL toluene in a 90 ºC oil bath for 18 h ((*E*,*E*)/(*Z*,*E*) > 91:9). The product was purified by flash column chromatography on silica gel (ethyl acetate : hexane = 1 : 50) and obtained as a colorless oil (44.6 mg, 63% yield, (*E*,*E*)/(*Z*,*E*) = 89:11), R_f = 0.25 (ethyl acetate : hexane = 1 : 100). **^{1}H NMR** (500 MHz, $CDCl_3$): δ (ppm) 7.94 (dd, *J* = 28.0, 15.6 Hz, 1H, **3r**), 7.76 (d, *J* = 16.0 Hz, **3r'**), 7.63 (dd, *J* = 6.5, 3.0 Hz, **3r'**), 7.43 – 7.34 (m, 10H, **3r**+**3r'**), 7.17 – 7.08 (m, **3r'**), 6.48 (d, *J* = 15.6 Hz, 1H, **3r**), 6.49 (d, *J* = 15.6 Hz, **3r'**), 5.26 (s, 2H, **3r**), 5.18 (s, **3r'**), 4.31 (q, *J* = 7.1 Hz, 2H, **3r+3r'**), 1.29 (t, *J* = 7.1 Hz, 3H, **3r**+**3r'**). **^{13}C NMR** (126 MHz, $CDCl_3$): δ (ppm) 166.1 (d, *J* = 15.0 Hz), 165.7, 158.5 (d, *J* = 262.2 Hz), 143.5, 135.7, 133.3 (d, *J* = 21.8 Hz), 131.6, 129.6 (d, *J* = 3.9 Hz), 128.9, 128.5, 128.5, 128.4, 123.7 (d, *J* = 5.3 Hz), 122.2 (d, *J* = 18.4 Hz), 66.9, 66.9 (**3r'**), 62.2, 61.9 (**3r'**), 14.2, 14.2 (**3r'**). **^{19}F NMR** (471 MHz, $CDCl_3$): δ (ppm) -110.54 (d, *J* = 27.9 Hz, 1F), -111.17 (d, *J* = 26.1 Hz, **3r'**). **HRMS** (ESI) m/z: $[M+Na]^+$ Calcd for $C_{21}H_{19}FO_4Na$ 377.1160; Found 377.1157.

ethyl (2*E*,4*E*)-3-fluoro-6-morpholino-6-oxo-2-phenylhexa-2,4-dienoate (3s)

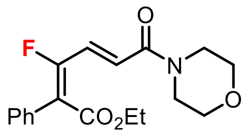

Following the general procedure (III), reaction was run using *gem*-difluoroalkene **1a** (42.4 mg, 0.2 mmol), 4-acryloylmorpholine (84.7 mg, 0.6 mmol), Pd(dba)$_2$ (11.6 mg, 0.02 mmol), dppb (8.6 mg, 0.02 mmol), dry NaI (90 mg, 0.6 mmol) and Et_3N (60.7 mg, 83.6 µL, 0.6 mmol) in 0.3 mL toluene in a 90 ºC oil bath for 18 h ((*E*,*E*)/(*Z*,*E*) = 89:11). The product was purified by flash column chromatography on silica gel (ethyl acetate : hexane = 1 : 5) and obtained as a colorless oil (38.0 mg, 57% yield, (*E*,*E*)/(*Z*,*E*) = 89:11), R_f = 0.12 (ethyl acetate : hexane = 1 : 50). **^{1}H NMR** (500 MHz, $CDCl_3$): δ (ppm) 7.83 (dd, *J* = 28.4, 15.1 Hz, 1H), 7.40 – 7.32 (m, 5H), 6.84 (d, *J* = 15.1 Hz, 1H), 4.31 (q, *J* = 7.1 Hz, 2H), 3.72 – 3.59 (m, 8H), 1.31 (t, *J* = 7.1 Hz, 3H). **^{13}C NMR** (126 MHz, $CDCl_3$): δ (ppm) 166.2 (d, *J* = 15.1 Hz), 164.3, 159.0 (d, *J* = 261.2 Hz), 131.8, 131.4 (d, *J* = 20.8 Hz), 129.5 (d, *J* = 3.9 Hz), 128.7, 128.3, 122.4 (d, *J* = 3.8 Hz), 121.1 (d, *J* = 18.9 Hz), 66.9, 62.1, 46.4, 42.6, 14.2. **^{19}F NMR** (471 MHz, $CDCl_3$): δ (ppm) -109.96 (d, *J* = 28.4 Hz, 1F). **HRMS** (ESI) m/z: $[M+Na]^+$ Calcd for $C_{18}H_{20}FNO_4Na$ 356.1269; Found 356.1264.

ethyl (2*E*,4*E*)-3-fluoro-2,5-bis(4-methoxyphenyl)penta-2,4-dienoate (4a)

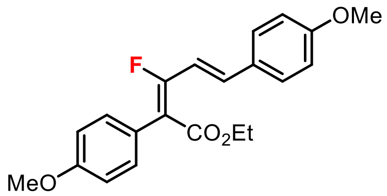

Following the general procedure (III), reaction was run using *gem*-difluoroalkene **1a'** (48.4 mg, 0.2 mmol), 4-methoxystyrene (80.5 mg, 0.6 mmol), Pd(dba)$_2$ (11.6 mg, 0.02 mmol), dppb (8.6 mg, 0.02 mmol), dry NaI (90 mg, 0.6 mmol) and Et_3N (60.7 mg, 83.6 µL, 0.6 mmol) in 0.3 mL toluene in a 90 ºC oil bath for 18 h ((*E*,*E*)/(*Z*,*E*) > 99:1). The product was purified by flash column chromatography on silica gel (ethyl acetate : hexane = 1 : 20) and obtained as a colorless solid (38.5 mg, 54% yield, (*E*,*E*)/(*Z*,*E*) > 99:1), R_f = 0.18 (ethyl acetate : hexane = 1 : 40). **^{1}H NMR** (500 MHz, $CDCl_3$): δ (ppm) 7.54 (dd, *J* = 27.8, 16.0 Hz, 1H), 7.41 (d, *J* = 8.8 Hz, 2H), 7.18 (d, *J* = 8.2 Hz, 2H), 7.09 (d, *J* = 16.0 Hz, 1H), 6.83 (dd, *J* = 8.7, 6.3 Hz, 4H), 4.20 (q, *J* = 7.1 Hz, 2H), 3.76 (d, *J* = 1.9 Hz, 6H), 1.21 (t, *J* = 7.1 Hz, 3H). **^{13}C NMR** (126 MHz, $CDCl_3$): δ (ppm) 167.5 (d, *J* = 16.4 Hz), 163.2 (d, *J* = 259.8 Hz), 160.8, 159.1, 135.3 (d, *J* = 7.8 Hz), 131.3 (d, *J* = 2.9 Hz), 129.3, 128.7, 125.4, 115.9 (d, *J* = 19.5 Hz), 114.4, 114.2 (d, *J* = 21.2 Hz), 113.5, 61.2, 55.5, 55.3, 14.4. **^{19}F NMR** (471 MHz, $CDCl_3$): δ (ppm) -106.40 (d, *J* = 27.7 Hz, 1F). **HRMS** (ESI) m/z: $[M+Na]^+$ Calcd for $C_{21}H_{21}FO_4Na$ 379.1316; Found 379.1313.

ethyl (2*E*,4*E*)-3-fluoro-2-(4-fluorophenyl)-5-(4-methoxyphenyl)penta-2,4-dienoate (4b)

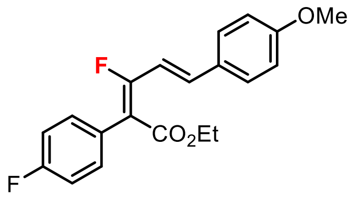

Following the general procedure (III), reaction was run using *gem*-difluoroalkene **1b** (46.0 mg, 0.2 mmol), 4-methoxystyrene (80.5 mg, 0.6 mmol), Pd(dba)$_2$ (11.6 mg, 0.02 mmol), dppb (8.6 mg, 0.02 mmol), dry NaI (90 mg, 0.6 mmol) and Et_3N (60.7 mg, 83.6 µL, 0.6 mmol) in 0.3 mL toluene in a 90 ºC oil bath for 18 h ((*E*,*E*)/(*Z*,*E*) > 99:1). The product was purified by flash column chromatography on silica gel (ethyl acetate : hexane = 1 : 20) and obtained as a colorless solid (50.2 mg, 73% yield, (*E*,*E*)/(*Z*,*E*) > 99:1), R_f = 0.21 (ethyl acetate : hexane = 1 : 40). **^{1}H NMR** (500 MHz, $CDCl_3$): δ (ppm) 7.70 (dd, *J* = 27.8, 16.0 Hz, 1H), 7.50 (d, *J* = 8.8 Hz, 2H), 7.30 – 7.26 (m, 2H), 7.21 (d, *J* = 16.1 Hz, 1H), 7.07 (t, *J* = 8.7 Hz, 2H), 6.91 (d, *J* = 8.8 Hz, 2H), 4.27 (q, *J* = 7.1 Hz, 2H), 3.84 (s, 3H), 1.28 (t, *J* = 7.1 Hz, 3H). **^{13}C NMR** (126 MHz, $CDCl_3$): δ (ppm) 166.9 (d, *J* = 16.4 Hz), 164.0 (d, *J* = 260.9 Hz), 162.2 (d, *J* = 246.9 Hz), 161.0, 136.2 (d, *J* = 8.1 Hz), 131.9 (dd, *J* = 8.2, 2.6 Hz), 129.4, 129.1 (t, *J* = 3.6, 2.3 Hz), 128.5, 115.6 (d, *J* = 19.1 Hz), 115.0 (d, *J* = 21.4 Hz), 114.5, 113.3 (d, *J* = 21.7 Hz), 61.3, 55.5, 14.3. **^{19}F NMR** (471 MHz, $CDCl_3$): δ (ppm) -103.93 (d, *J* = 27.8 Hz, 1F), -116.40 – -116.46 (m, 1F). **HRMS** (ESI) m/z: $[M+Na]^+$ Calcd for $C_{20}H_{18}F_2O_3Na$ 367.1116; Found 367.1114.

ethyl (2*E*,4*E*)-2-(4-chlorophenyl)-3-fluoro-5-(4-methoxyphenyl)penta-2,4-dienoate (4c)

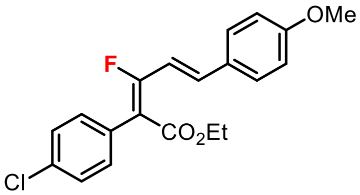

Following the general procedure (III), reaction was run using *gem*-difluoroalkene **1c** (49.3 mg, 0.2 mmol), 4-methoxystyrene (80.5 mg, 0.6 mmol), Pd(dba)$_2$ (11.6 mg, 0.02 mmol), dppb (8.6 mg, 0.02 mmol), dry NaI (90 mg, 0.6 mmol) and Et$_3$N (60.7 mg, 83.6 μL, 0.6 mmol) in 0.3 mL toluene in a 90 ºC oil bath for 18 h ((*E*,*E*)/(*Z*,*E*) = 99:1). The product was purified by flash column chromatography on silica gel (ethyl acetate : hexane = 1 : 20) and obtained as a colorless solid (47.5 mg, 66% yield, (*E*,*E*)/(*Z*,*E*) = 98:2), R_f = 0.22 (ethyl acetate : hexane = 1 : 40). **^{1}H NMR** (500 MHz, CDCl$_3$): δ (ppm) 7.61 (dd, *J* = 27.8, 16.0 Hz, 1H), 7.42 (d, *J* = 8.8 Hz, 2H), 7.26 (d, *J* = 8.5 Hz, 2H), 7.17 – 7.12 (m, 3H), 6.83 (d, *J* = 8.7 Hz, 2H), 4.18 (q, *J* = 7.1 Hz, 2H), 3.76 (s, 3H), 1.19 (t, *J* = 7.1 Hz, 3H). **^{13}C NMR** (126 MHz, CDCl$_3$): δ (ppm) 166.7 (d, *J* = 16.4 Hz), 164.2 (d, *J* = 261.6 Hz), 161.0, 136.5 (d, *J* = 8.0 Hz), 133.6, 131.7 (d, *J* = 2.1 Hz), 131.6 (d, *J* = 2.7 Hz), 129.5, 128.4, 128.2, 115.5 (d, *J* = 19.0 Hz), 114.5, 113.2 (d, *J* = 21.5 Hz), 61.3, 55.5, 14.3. **^{19}F NMR** (471 MHz, CDCl$_3$): δ (ppm) -101.33 (d, *J* = 27.9 Hz, 1F). **HRMS** (ESI) m/z: $[M+Na]^+$ Calcd for $C_{20}H_{18}ClFO_3Na$ 383.0821; Found 383.0816.

ethyl (2*E*,4*E*)-2-(4-bromophenyl)-3-fluoro-5-(4-methoxyphenyl)penta-2,4-dienoate (4d)

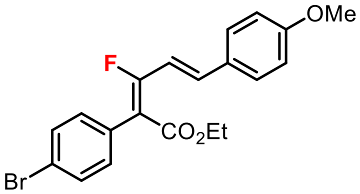

Following the general procedure (III), reaction was run using *gem*-difluoroalkene **1d** (58.2 mg, 0.2 mmol), 4-methoxystyrene (80.5 mg, 0.6 mmol), Pd(dba)$_2$ (11.6 mg, 0.02 mmol), dppb (8.6 mg, 0.02 mmol), dry NaI (90 mg, 0.6 mmol) and Et$_3$N (60.7 mg, 83.6 μL, 0.6 mmol) in 0.3 mL toluene in a 90 ºC oil bath for 18 h ((*E*,*E*)/(*Z*,*E*) = 99:1). The product was purified by flash column chromatography on silica gel (ethyl acetate : hexane = 1 : 20) and obtained as a colorless solid (55.8 mg, 69% yield, (*E*,*E*)/(*Z*,*E*) = 97:3), R_f = 0.22 (ethyl acetate : hexane = 1 : 40). **^{1}H NMR** (500 MHz, CDCl$_3$): δ (ppm) 7.69 (dd, *J* = 27.9, 16.0 Hz, 1H), 7.50 (dd, *J* = 8.6, 1.6 Hz, 4H), 7.24 – 7.18 (m, 3H), 6.93 – 6.90 (m, 2H), 4.26 (q, *J* = 7.1 Hz, 2H), 3.84 (s, 3H), 1.28 (t, *J* = 7.1 Hz, 3H). **^{13}C NMR** (126 MHz, CDCl$_3$): δ (ppm) 166.7 (d, *J* = 16.3 Hz), 164.1 (d, *J* = 261.7 Hz), 161.0, 136.6 (d, *J* = 8.2 Hz), 132.2 (d, *J* = 2.1 Hz), 131.9 (d, *J* = 2.7 Hz), 131.2, 129.5, 128.4, 121.8, 115.5 (d, *J* = 18.8 Hz), 114.5, 113.2 (d, *J* = 21.4 Hz), 61.3, 55.5, 14.3. **^{19}F NMR** (471 MHz, CDCl$_3$): δ (ppm) -103.34 (d, *J* = 27.8 Hz, 1F). **HRMS** (ESI) m/z: $[M+Na]^+$ Calcd for $C_{20}H_{18}BrFO_3Na$ 427.0316; Found 427.0314.

ethyl (2*E*,4*E*)-3-fluoro-5-(4-methoxyphenyl)-2-(4-(trifluoromethyl)phenyl)penta-2,4-dienoate (4e)

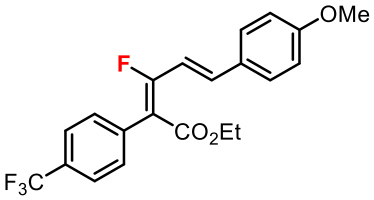

Following the general procedure (III), reaction was run using *gem*-difluoroalkene **1e** (56.0 mg, 0.2 mmol), 4-methoxystyrene (80.5 mg, 0.6 mmol), Pd(dba)$_2$ (11.6 mg, 0.02 mmol), dppb (8.6 mg, 0.02 mmol), dry NaI (90 mg, 0.6 mmol) and Et$_3$N (60.7 mg, 83.6 μL, 0.6 mmol) in 0.3 mL toluene in a 90 ºC oil bath for 18 h ((*E*,*E*)/(*Z*,*E*) = 92:8). The product was purified by flash column chromatography on silica gel (ethyl acetate : hexane = 1 : 20) and obtained as a colorless solid (42.6 mg, 54% yield, (*E*,*E*)/(*Z*,*E*) = 97:3), R_f = 0.17 (ethyl acetate : hexane = 1 : 40). **^{1}H NMR** (500 MHz, CDCl$_3$): δ (ppm) 7.76 (dd, *J* = 27.9, 16.0 Hz, 1H), 7.66 (d, *J* = 8.1 Hz, 2H), 7.54 (d, *J* = 8.8 Hz, 2H), 7.46 (d, *J* = 8.1 Hz, 2H), 7.29 (s, 1H), 6.95 (d, *J* = 8.8 Hz, 2H), 4.30 (q, *J* = 7.1 Hz, 2H), 3.88 (s, 3H), 1.31 (t, *J* = 7.2 Hz, 3H). **^{13}C NMR** (126 MHz, CDCl$_3$): δ (ppm) 166.5 (d, *J* = 16.4 Hz), 164.7 (d, *J* = 262.7 Hz), 161.2, 137.1 (d, *J* = 8.2 Hz), 137.1, 131.4 (d, *J* = 2.7 Hz), 130.7 (d, *J* = 2.7 Hz), 129.6, 128.3, 124.9 (q, *J* = 3.7 Hz), 124.3 (q, *J* = 272.1 Hz), 115.3 (d, *J* = 18.6 Hz), 114.5, 113.0 (d, *J* = 21.3 Hz), 61.4, 55.5, 14.3. **^{19}F NMR** (471 MHz,

$CDCl_3$): δ (ppm) -64.73(s, 3F), -102.60 (d, J = 27.8 Hz, 1F). **HRMS** (ESI) m/z: $[M+Na]^+$ Calcd for $C_{21}H_{18}F_4O_3Na$ 417.1084; Found 417.1078.

ethyl (2*E*,4*E*)-2-([1,1'-biphenyl]-4-yl)-3-fluoro-5-(4-methoxyphenyl)penta-2,4-dienoate (4f)

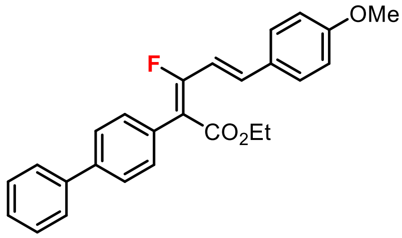

Following the general procedure (III), reaction was run using *gem*-difluoroalkene **1f** (57.6 mg, 0.2 mmol), 4-methoxystyrene (80.5 mg, 0.6 mmol), $Pd(dba)_2$ (11.6 mg, 0.02 mmol), dppb (8.6 mg, 0.02 mmol), dry NaI (90 mg, 0.6 mmol) and Et_3N (60.7 mg, 83.6 μL, 0.6 mmol) in 0.3 mL toluene in a 90 °C oil bath for 18 h ((*E*,*E*)/(*Z*,*E*) > 99:1). The product was purified by flash column chromatography on silica gel (ethyl acetate : hexane = 1 : 20) and obtained as a colorless solid (61.1 mg, 76% yield, (*E*,*E*)/(*Z*,*E*) = 92:8), R_f = 0.22 (ethyl acetate : hexane = 1 : 40). **^{1}H NMR** (500 MHz, $CDCl_3$): δ (ppm) 7.71 – 7.61 (m, 5H), 7.52 – 7.34 (m, 7H), 7.26 – 7.21 (m, 1H), 6.92 (d, J = 8.8 Hz, 2H), 4.31 (q, J = 7.2 Hz, 2H), 3.85 (s, 3H), 1.31 (t, J = 7.1 Hz, 3H). **^{13}C NMR** (126 MHz, $CDCl_3$): δ (ppm) 167.2 (d, J = 16.4 Hz), 163.7 (d, J = 261.2 Hz), 160.9, 143.5, 140.7 (d, J = 51.4 Hz), 135.9 (d, J = 7.9 Hz), 132.2, 130.6 (d, J = 2.9 Hz), 129.4, 128.9, 128.6, 127.5, 127.2, 126.8, 115.8 (d, J = 19.3 Hz), 114.5, 114.2 (d, J = 21.0 Hz), 61.3, 55.5, 14.4. **^{19}F NMR** (471 MHz, $CDCl_3$): δ (ppm) -104.89 (d, J = 27.7 Hz, 1F). **HRMS** (ESI) m/z: $[M+Na]^+$ Calcd for $C_{26}H_{23}FO_3Na$ 425.1523; Found 425.1520.

ethyl (2*E*,4*E*)-3-fluoro-5-(4-methoxyphenyl)-2-(naphthalen-2-yl)penta-2,4-dienoate (4g)

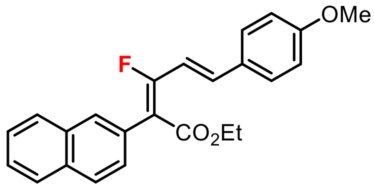

Following the general procedure (III), reaction was run using *gem*-difluoroalkene **1g** (52.4 mg, 0.2 mmol), 4-methoxystyrene (80.5 mg, 0.6 mmol), $Pd(dba)_2$ (11.6 mg, 0.02 mmol), dppb (8.6 mg, 0.02 mmol), dry NaI (90 mg, 0.6 mmol) and Et_3N (60.7 mg, 83.6 μL, 0.6 mmol) in 0.3 mL toluene in a 90 °C oil bath for 18 h ((*E*,*E*)/(*Z*,*E*) = 99:1). The product was purified by flash column chromatography on silica gel (ethyl acetate : hexane = 1 : 20) and obtained as a colorless solid (56.4 mg, 75% yield, (*E*,*E*)/(*Z*,*E*) = 98:2), R_f = 0.17 (ethyl acetate : hexane = 1 : 40). **^{1}H NMR** (500 MHz, $CDCl_3$): δ (ppm) 7.86 – 7.82 (m, 4H), 7.73 (dd, J = 27.7, 16.0 Hz, 1H), 7.54 – 7.48 (m, 4H), 7.42 (d, J = 8.6 Hz, 1H), 7.24 (d, J = 16.0 Hz, 1H), 6.92 (d, J = 8.8 Hz, 2H), 4.29 (q, J = 7.1 Hz, 2H), 3.85 (s, 3H), 1.27 (t, J = 7.1 Hz, 3H). **^{13}C NMR** (126 MHz, $CDCl_3$): δ (ppm) 167.2 (d, J = 16.4 Hz), 164.0 (d, J = 261.1 Hz), 160.9, 136.0 (d, J = 7.9 Hz), 133.0 (d, J = 52.9 Hz), 130.7 (d, J = 1.8 Hz), 129.4, 129.4 (d, J = 3.0 Hz), 129.1, 128.6, 128.3, 128.1 (d, J = 2.3 Hz), 127.8, 127.4, 126.3, 126.2, 115.8 (d, J = 19.3 Hz), 114.5, 114.4 (d, J = 21.2 Hz), 61.3, 55.5, 14.4. **^{19}F NMR** (471 MHz, $CDCl_3$): δ (ppm) -104.67 (d, J = 27.7 Hz, 1F). **HRMS** (ESI) m/z: $[M+Na]^+$ Calcd for $C_{24}H_{21}FO_3Na$ 399.1367; Found 399.1364.

ethyl (2*E*,4*E*)-3-fluoro-5-(4-methoxyphenyl)-2-(thiophen-3-yl)penta-2,4-dienoate (4h)

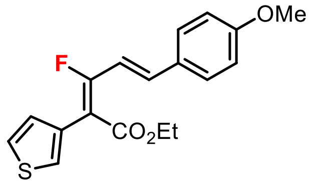

Following the general procedure (III), reaction was run using *gem*-difluoroalkene **1h** (43.6 mg, 0.2 mmol), 4-methoxystyrene (80.5 mg, 0.6 mmol), $Pd(dba)_2$ (11.6 mg, 0.02 mmol), dppb (8.6 mg, 0.02 mmol), dry NaI (90 mg, 0.6 mmol) and Et_3N (60.7 mg, 83.6 μL, 0.6 mmol) in 0.3 mL toluene in a 90 °C oil bath for 18 h. ((*E*,*E*)/(*Z*,*E*) = 98:2) The product was purified by flash column chromatography on silica gel (ethyl acetate : hexane = 1 : 20) and obtained as a colorless solid (42.5 mg, 64% yield, (*E*,*E*)/(*Z*,*E*) = 98:2), R_f = 0.25 (ethyl acetate : hexane = 1 : 40). **^{1}H NMR** (500 MHz, $CDCl_3$): δ (ppm) 7.48 – 7.39 (m, 4H), 7.30

(dd, J = 5.0, 3.0 Hz, 1H), 7.21 – 7.16 (m, 2H), 6.92 – 6.89 (m, 2H), 4.34 (q, J = 7.1 Hz, 2H), 3.84 (s, 3H), 1.36 (t, J = 7.1 Hz, 3H). **^{13}C NMR** (126 MHz, $CDCl_3$): δ (ppm) 167.2 (d, J = 16.7 Hz), 162.4 (d, J = 262.2 Hz), 160.8, 135.0 (d, J = 7.8 Hz), 132.3, 129.2, 129.2 (d, J = 4.4 Hz), 128.6, 125.3 (d, J = 6.0 Hz), 124.3, 115.7 (d, J = 19.4 Hz), 114.5, 110.2 (d, J = 20.0 Hz), 61.5, 55.5, 14.4. **^{19}F NMR** (471 MHz, $CDCl_3$): δ (ppm) -107.15 (d, J = 27.6 Hz, 1F). **HRMS** (ESI) m/z: $[M+Na]^+$ Calcd for $C_{18}H_{17}FO_3SNa$ 355.0775; Found 355.0772.

isopropyl (2*E*,4*E*)-3-fluoro-2-(4-fluorophenyl)-5-(4-methoxyphenyl)penta-2,4-dienoate (4i)

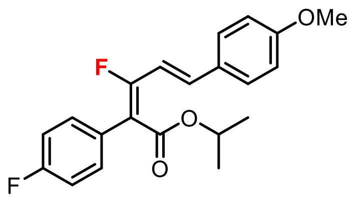

Following the general procedure (III), reaction was run using *gem*-difluoroalkene **1i** (48.8 mg, 0.2 mmol), 4-methoxystyrene (80.5 mg, 0.6 mmol), $Pd(dba)_2$ (11.6 mg, 0.02 mmol), dppb (8.6 mg, 0.02 mmol), dry NaI (90 mg, 0.6 mmol) and Et_3N (60.7 mg, 83.6 μL, 0.6 mmol) in 0.3 mL toluene in a 90 ºC oil bath for 18 h ((*E*,*E*)/(*Z*,*E*) > 99:1). The product was purified by flash column chromatography on silica gel (ethyl acetate : hexane = 1 : 20) and obtained as a colorless solid (48.0 mg, 67% yield, (*E*,*E*)/(*Z*,*E*) = 99:1), R_f = 0.28 (ethyl acetate : hexane = 1 : 40). **^{1}H NMR** (500 MHz, $CDCl_3$): δ (ppm) 7.64 (dd, J = 27.8, 16.0 Hz, 1H), 7.49 (d, J = 8.8 Hz, 2H), 7.30 – 7.26 (m, 2H), 7.19 (d, J = 16.1 Hz, 1H), 7.06 (t, J = 8.7 Hz, 2H), 6.91 (d, J = 8.8 Hz, 2H), 5.19 – 5.14 (m, 1H), 3.84 (s, 3H), 1.28 (d, J = 6.3 Hz, 6H). **^{13}C NMR** (126 MHz, $CDCl_3$): δ (ppm) 166.5 (d, J = 16.1 Hz), 163.5 (d, J = 260.7 Hz), 162.2 (d, J = 246.8 Hz), 160.9, 135.8 (d, J = 8.0 Hz), 131.9 (dd, J = 8.1, 2.8 Hz), 129.3, 129.2 (dd, J = 3.5, 1.9 Hz), 128.5, 115.7 (d, J = 19.5 Hz), 115.0 (d, J = 21.6 Hz), 114.5, 113.8 (d, J = 20.8 Hz), 68.9, 55.5, 21.9. **^{19}F NMR** (471 MHz, $CDCl_3$): δ (ppm) -105.20 (d, J = 27.7 Hz, 1F), -116.49 – -116.55 (m, 1F). **HRMS** (ESI) m/z: $[M+Na]^+$ Calcd for $C_{21}H_{20}F_2O_3Na$ 381.1273; Found 381.1267.

benzyl (2*E*,4*E*)-3-fluoro-2-(4-fluorophenyl)-5-(4-methoxyphenyl)penta-2,4-dienoate (4j)

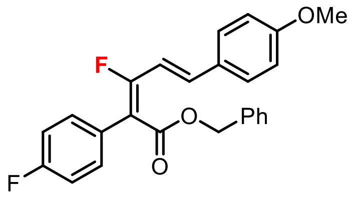

Following the general procedure (III), reaction was run using *gem*-difluoroalkene **1j** (58.4 mg, 0.2 mmol), 4-methoxystyrene (80.5 mg, 0.6 mmol), $Pd(dba)_2$ (11.6 mg, 0.02 mmol), dppb (8.6 mg, 0.02 mmol), dry NaI (90 mg, 0.6 mmol) and Et_3N (60.7 mg, 83.6 μL, 0.6 mmol) in 0.3 mL toluene in a 90 ºC oil bath for 18 h ((*E*,*E*)/(*Z*,*E*) = 98:2). The product was purified by flash column chromatography on silica gel (ethyl acetate : hexane = 1 : 20) and obtained as a colorless solid (26.0 mg, 32% yield, (*E*,*E*)/(*Z*,*E*) = 98:2), R_f = 0.29 (ethyl acetate : hexane = 1 : 40). **^{1}H NMR** (500 MHz, $CDCl_3$): δ (ppm) 7.53 (dd, J = 27.9, 16.0 Hz, 1H), 7.31 – 7.18 (m, 9H), 7.10 (d, J = 16.0 Hz, 1H), 6.98 (t, J = 8.7 Hz, 2H), 6.79 (d, J = 8.7 Hz, 2H), 5.18 (s, 2H), 3.76 (s, 3H). **^{13}C NMR** (126 MHz, $CDCl_3$): δ (ppm) 166.7 (d, J = 16.4 Hz), 164.3 (d, J = 261.8 Hz), 162.3 (d, J = 247.3 Hz), 161.0, 136.5 (d, J = 8.1 Hz), 135.9, 132.0 (dd, J = 8.2, 2.6 Hz), 129.5, 128.8, 128.4, 128.4, 128.2, 115.5 (d, J = 19.2 Hz), 115.2, 115.0, 114.4, 113.0 (d, J = 21.9 Hz), 66.9, 55.5. **^{19}F NMR** (471 MHz, $CDCl_3$): δ (ppm) -102.59 (d, J = 27.9 Hz, 1F), -115.95 – -116.01 (m, 1F). **HRMS** (ESI) m/z: $[M+Na]^+$ Calcd for $C_{25}H_{20}F_2O_3Na$ 429.1273; Found 429.1269.

ethyl (2*E*,4*E*)-2-benzyl-3-fluoro-5-(3-methoxyphenyl)penta-2,4-dienoate (4k)

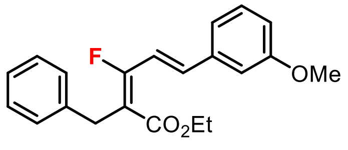

Following the general procedure (IV), reaction was run using *gem*-difluoroalkene **1k** (45.2mg, 0.2 mmol), 3-methoxystyrene (80.5 mg, 0.6 mmol), $Pd(dba)_2$ (11.6 mg, 0.02 mmol), dppb (8.6 mg, 0.02 mmol), dry NaI (90 mg, 0.6 mmol) and (*n*-propyl)$_3$N (86.0 mg, 114 μL, 0.6 mmol) in 0.3 mL toluene in a 90 ºC oil bath for 36 h ((*E*,*E*)/(*Z*,*E*) > 99:1). The product was purified by flash column chromatography on silica gel (ethyl acetate : hexane = 1 : 20) and obtained as a colorless oil (43.5 mg,

64% yield, (*E*,*E*)/(*Z*,*E*) > 99:1), R_f = 0.23 (ethyl acetate : hexane = 1 : 40). **^{1}H NMR** (500 MHz, $CDCl_3$): δ (ppm) 7.76 (dd, *J* = 28.4, 16.1 Hz, 1H), 7.21 – 7.17 (m, 5H), 7.12 – 7.09 (m, 2H), 7.04 (d, *J* = 7.6 Hz, 1H), 6.96 (s, 1H), 6.80 (d, *J* = 8.2 Hz, 1H), 4.14 (q, *J* = 7.1 Hz, 2H), 3.75 – 3.74 (m, 5H), 1.20 (t, *J* = 7.2 Hz, 3H). **^{13}C NMR** (126 MHz, $CDCl_3$): δ (ppm) 166.9 (d, *J* = 18.2 Hz), 164.7 (d, *J* = 259.4 Hz), 160.0, 139.8 (d, *J* = 1.7 Hz), 137.3, 135.6 (d, *J* = 8.1 Hz), 129.9, 128.7, 128.5, 126.3, 120.4, 118.5 (d, *J* = 19.8 Hz), 115.3, 113.7 (d, *J* = 22.4 Hz), 112.7, 61.0, 55.4, 31.9 (d, *J* = 7.2 Hz), 14.3. **^{19}F NMR** (471 MHz, $CDCl_3$): δ (ppm) -102.45 (d, *J* = 28.1 Hz, 1F). **HRMS** (ESI) m/z: $[M+Na]^+$ Calcd for $C_{21}H_{21}FO_3Na$ 363.1367; Found 363.1363.

ethyl (2*E*,4*E*)-3-fluoro-2-(4-methoxybenzyl)-5-(3-methoxyphenyl)penta-2,4-dienoate (4l)

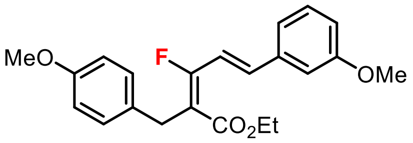

Following the general procedure (IV), reaction was run using *gem*-difluoroalkene **1l** (51.2 mg, 0.2 mmol), 3-methoxystyrene (80.5 mg, 0.6 mmol), $Pd(dba)_2$ (11.6 mg, 0.02 mmol), dppb (8.6 mg, 0.02 mmol), dry NaI (90 mg, 0.6 mmol) and (*n*- propyl)$_3$N (86.0 mg, 114 µL, 0.6 mmol) in 0.3 mL toluene in a 90 ºC oil bath for 36 h ((*E*,*E*)/(*Z*,*E*) > 99:1). The product was purified by flash column chromatography on silica gel (ethyl acetate : hexane = 1 : 10) and obtained as a colorless oil (46.6 mg, 63% yield, (*E*,*E*)/(*Z*,*E*) > 99:1), R_f = 0.26 (ethyl acetate : hexane = 1 : 20). **^{1}H NMR** (500 MHz, $CDCl_3$) δ (ppm) 7.83 (dd, *J* = 28.4, 16.1 Hz, 1H), 7.28 (t, *J* = 7.9 Hz, 1H), 7.21 – 7.12 (m, 4H), 7.04 (s, 1H), 6.88 (dd, *J* = 8.2, 2.5 Hz, 1H), 6.83 (d, *J* = 8.5 Hz, 2H), 4.23 (q, *J* = 7.1 Hz, 2H), 3.84 – 3.76 (m, 8H), 1.31 (t, *J* = 7.1 Hz, 3H). **^{13}C NMR** (126 MHz, $CDCl_3$) δ (ppm) 166.9 (d, *J* = 18.2 Hz), 164.4 (d, *J* = 259.0 Hz), 160.0, 158.1, 137.3, 135.4 (d, *J* = 8.0 Hz), 131.8, 129.9, 129.7, 120.4, 118.5 (d, *J* = 19.9 Hz), 115.2, 114.1 (d, *J* = 22.5 Hz), 113.8, 112.7, 61.0, 55.4, 55.3, 31.0 (d, *J* = 7.2 Hz), 14.3. **^{19}F NMR** (471 MHz, $CDCl_3$) δ (ppm) -105.12 (d, *J* = 28.6 Hz, 1F). **HRMS** (ESI) m/z: $[M+Na]^+$ Calcd for $C_{22}H_{23}FO_4Na$ 393.1473; Found 393.1465.

ethyl (2*E*,4*E*)-3-fluoro-2-(4-fluorobenzyl)-5-(3-methoxyphenyl)penta-2,4-dienoate (4m)

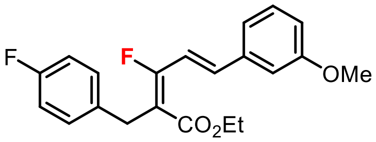

Following the general procedure (IV), reaction was run using *gem*-difluoroalkene **1m** (48.8 mg, 0.2 mmol), 3-methoxystyrene (80.5 mg, 0.6 mmol), $Pd(dba)_2$ (11.6 mg, 0.02 mmol), dppb (8.6 mg, 0.02 mmol), dry NaI (90 mg, 0.6 mmol) and (*n*- propyl)$_3$N (86.0 mg, 114 µL, 0.6 mmol) in 0.3 mL toluene in a 90 ºC oil bath for 36 h ((*E*,*E*)/(*Z*,*E*) > 99:1). The product was purified by flash column chromatography on silica gel (ethyl acetate : hexane = 1 : 20) and obtained as a colorless oil (51.6 mg, 72% yield, (*E*,*E*)/(*Z*,*E*) > 99:1), R_f = 0.20 (ethyl acetate : hexane = 1 : 40). **^{1}H NMR** (500 MHz, $CDCl_3$): δ (ppm) 7.74 (dd, *J* = 28.5, 16.1 Hz, 1H), 7.21 – 7.03 (m, 5H), 6.95 (s, 1H), 6.87 (t, *J* = 8.7 Hz, 2H), 6.80 (dd, *J* = 8.2, 2.5 Hz, 1H), 4.13 (q, *J* = 7.1 Hz, 2H), 3.74 (s, 3H), 3.69 (d, *J* = 3.5 Hz, 2H), 1.20 (t, *J* = 7.1 Hz, 3H). **^{13}C NMR** (126 MHz, $CDCl_3$): δ (ppm) 166.8 (d, *J* = 18.2 Hz), 164.7 (d, *J* = 259.5 Hz), 161.6 (d, *J* = 243.9 Hz), 160.0, 137.2, 135.8 (d, *J* = 8.0 Hz), 135.4 (dd, *J* = 3.0, 1.4 Hz), 130.1 (d, *J* = 7.8 Hz), 129.9, 120.4, 118.3 (d, *J* = 19.8 Hz), 115.3 (d, *J* = 3.5 Hz), 115.1, 113.5 (d, *J* = 22.4 Hz), 112.8, 61.1, 55.4, 31.1 (d, *J* = 7.5 Hz), 14.3. **^{19}F NMR** (471 MHz, $CDCl_3$) δ (ppm) -104.39 (d, *J* = 28.6 Hz, 1F), -119.04 – -119.10 (m, 1F). **HRMS** (ESI) m/z: $[M+CH_3OH+Na]^+$ Calcd for $C_{21}H_{20}F_2O_3Na$ 381.1273; Found 381.1266.

ethyl (2*E*,4*E*)-3-fluoro-5-(3-methoxyphenyl)-2-(4-(trifluoromethyl)benzyl)penta-2,4-dienoate (4n)

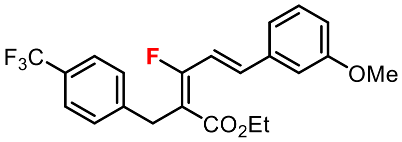

Following the general procedure (IV), reaction was run using *gem*-difluoroalkene **1n** (58.8 mg, 0.2 mmol), 3-methoxystyrene (80.5 mg, 0.6 mmol), $Pd(dba)_2$ (11.6 mg, 0.02 mmol), dppb (8.6 mg, 0.02 mmol), dry NaI (90 mg, 0.6 mmol) and (*n*- propyl)$_3$N (86.0 mg, 114 µL, 0.6 mmol) in 0.3 mL toluene in a 90 ºC oil bath for 36 h ((*E*,*E*)/(*Z*,*E*) > 99:1). The product was purified by flash column chromatography on silica gel (ethyl acetate : hexane = 1 : 20) and obtained as a colorless oil (42.6 mg, 54% yield,

(E,E)/(Z,E) > 99:1), R_f = 0.24 (ethyl acetate : hexane = 1 : 40). **^{1}H NMR** (500 MHz, $CDCl_3$): 7.85 (dd, J = 28.6, 16.1 Hz, 1H), 7.53 (d, J = 7.9 Hz, 2H), 7.38 (d, J = 7.9 Hz, 2H), 7.29 (t, J = 8.0 Hz, 1H), 7.21 (d, J = 16.1 Hz, 1H), 7.13 (d, J = 7.7 Hz, 1H), 7.05 (s, 1H), 6.90 (dd, J = 8.3, 2.5 Hz, 1H), 4.23 (q, J = 7.1 Hz, 2H), 3.87 (d, J = 3.5 Hz, 2H), 3.84 (s, 3H), 1.29 (t, J = 7.1 Hz, 3H). **^{13}C NMR** (126 MHz, $CDCl_3$): δ (ppm) 166.6 (d, J = 17.9 Hz), 165.2 (d, J = 260.2 Hz), 160.1, 144.0, 137.1, 136.2 (d, J = 8.2 Hz), 130.0, 129.0, 128.6 (q, J = 32.3 Hz), 125.4 (q, J = 3.8 Hz), 124.4 (q, J = 271.8 Hz), 120.5, 118.2 (d, J = 19.6 Hz), 115.4, 112.8, 112.6 (d, J = 22.2 Hz), 61.2, 55.4, 31.8 (d, J = 7.4 Hz), 14.3. **^{19}F NMR** (471 MHz, $CDCl_3$) δ (ppm) -62.32 (s, 3F), -101.36 (d, J = 28.6 Hz, 1F). **HRMS** (ESI) m/z: $[M+Na]^+$ Calcd for $C_{22}H_{20}F_4O_3Na$ 431.1241; Found 431.1234.

ethyl (*E*)-2-((*E*)-1-fluoro-3-(4-methoxyphenyl)allylidene)decanoate (4o)

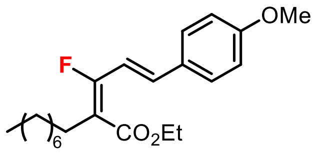

Following the general procedure (IV), reaction was run using *gem*-difluoroalkene **1o** (35.6 mg, 0.2 mmol), 4-methoxystyrene (80.5 mg, 0.6 mmol), Pd(dba)$_2$ (11.6 mg, 0.02 mmol), dppb (8.6 mg, 0.02 mmol), dry NaI (90 mg, 0.6 mmol) and (n- propyl)$_3$N (86.0 mg, 114 μL, 0.6 mmol) in 0.3 mL toluene in a 90 °C oil bath for 36 h ((E,E)/(Z,E) > 99:1). The product was purified by flash column chromatography on silica gel (ethyl acetate : hexane = 1 : 20) and obtained as a colorless oil (38.4 mg, 53% yield, (E,E)/(Z,E) > 99:1), R_f = 0.21 (ethyl acetate : hexane = 1 : 40). **^{1}H NMR** (500 MHz, $CDCl_3$): δ (ppm) 7.66 (dd, J = 28.5, 16.1 Hz, 1H), 7.45 (d, J = 8.8 Hz, 2H), 7.08 (d, J = 16.0 Hz, 1H), 6.88 (d, J = 8.8 Hz, 2H), 4.26 (q, J = 7.1 Hz, 2H), 3.83 (s, 3H), 2.46 – 2.42 (m, 2H), 1.49 – 1.41 (m, 2H), 1.37 – 1.28 (m, 13H), 0.88 (t, J = 6.9 Hz, 3H). **^{13}C NMR** (126 MHz, $CDCl_3$): δ (ppm) 167.7 (d, J = 19.0 Hz), 164.6 (d, J = 257.6 Hz), 160.5, 134.2 (d, J = 8.1 Hz), 129.1, 128.9, 116.3 (d, J = 20.0 Hz), 114.3, 113.9 (d, J = 23.4 Hz), 60.7, 55.5, 32.0, 29.6, 29.5, 29.4, 26.1 (d, J = 6.8 Hz), 22.8, 14.4, 14.3. **^{19}F NMR** (471 MHz, $CDCl_3$) δ (ppm) -106.44 (dt, J = 28.7, 4.0 Hz, 1F). **HRMS** (ESI) m/z: $[M+Na]^+$ Calcd for $C_{22}H_{31}FO_3Na$ 385.2149; Found 385.2144.

IX. Spectra.

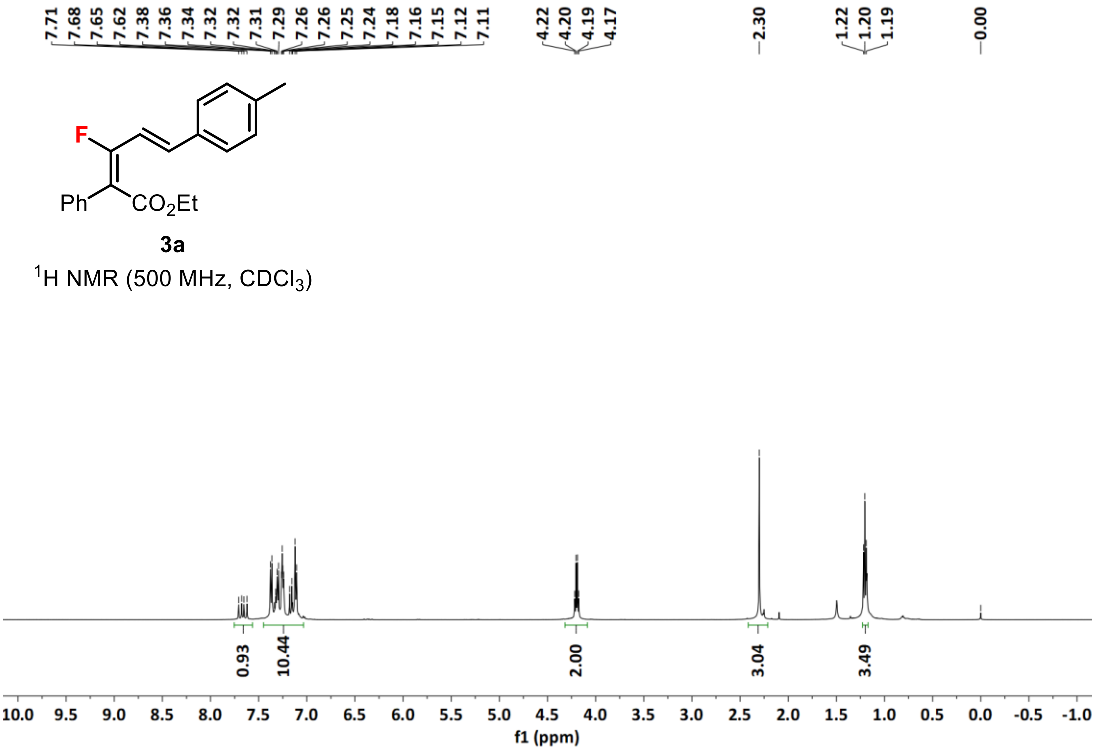

7.71
7.68
7.65
7.62
7.38
7.36
7.34
7.32
7.32
7.31
7.29
7.26
7.25
7.24
7.18
7.16
7.15
7.12
7.11
4.22
4.20
4.19
4.17
2.30
1.22
1.20
1.19
0.00
F
Ph
CO2Et
3a
1H NMR (500 MHz, CDCl3)
0.93
10.44
2.00
3.04
3.49
f1 (ppm)

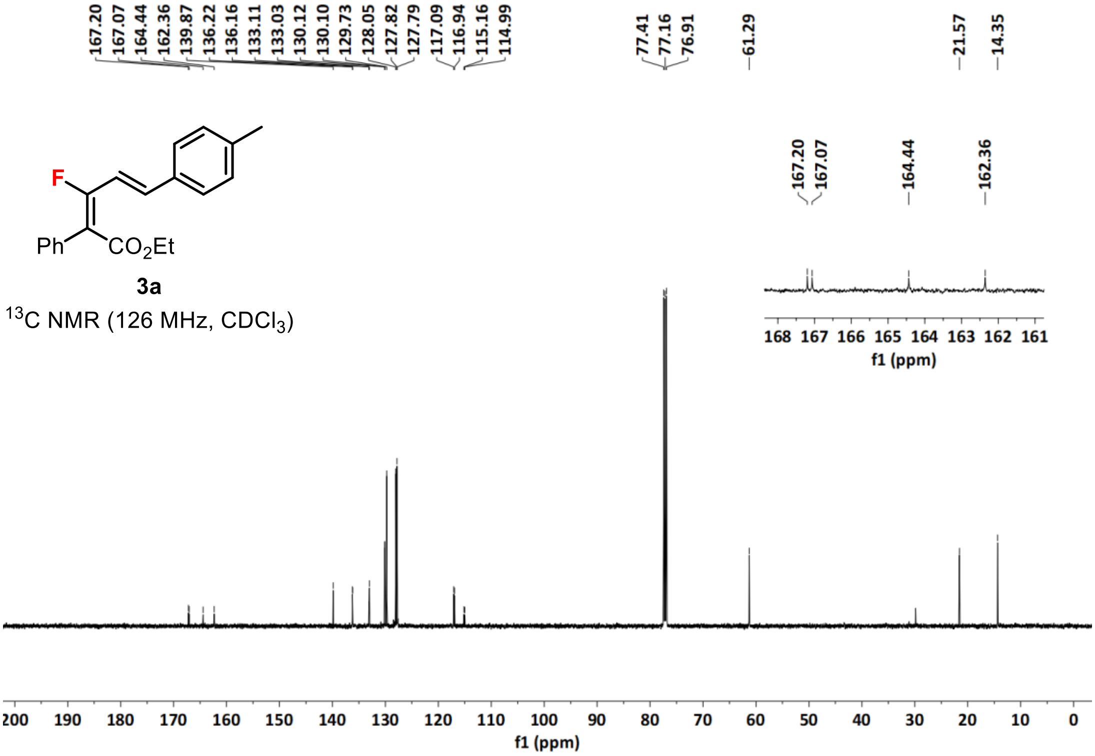

167.20
167.07
164.44
162.36
139.87
136.22
136.16
133.11
133.03
130.12
130.10
129.73
128.05
127.82
127.79
117.09
116.94
115.16
114.99
77.41
77.16
76.91
61.29
21.57
14.35
F
Ph
CO2Et
3a
13C NMR (126 MHz, CDCl3)
167.20
167.07
164.44
162.36
f1 (ppm)
f1 (ppm)

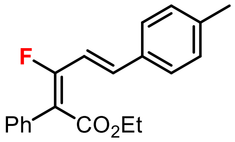

3a

^{19}F NMR (471 MHz, $CDCl_3$)

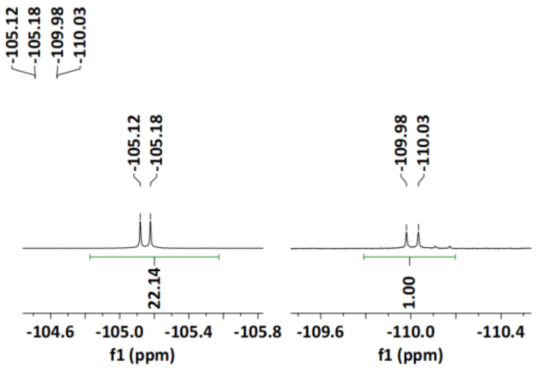

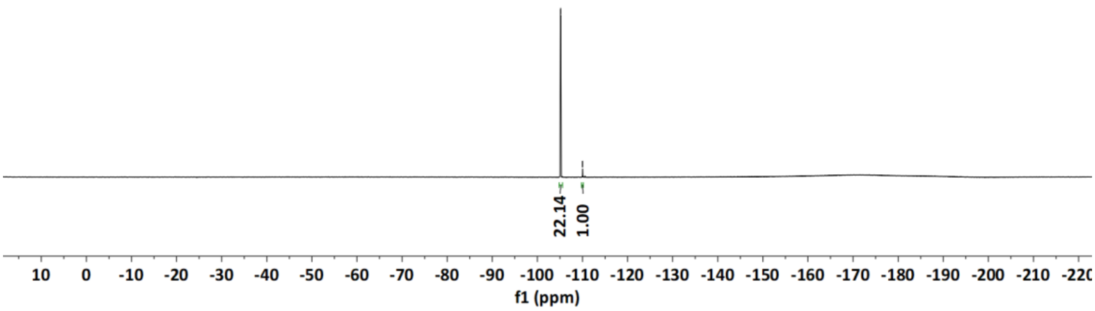

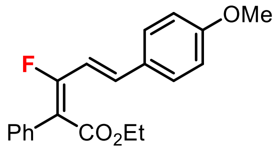

3b

^{1}H NMR (500 MHz, $CDCl_3$)

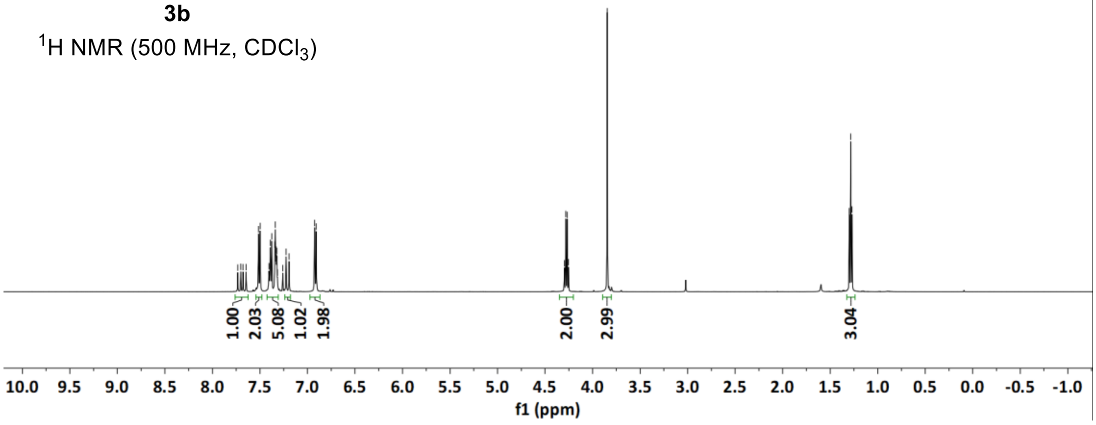

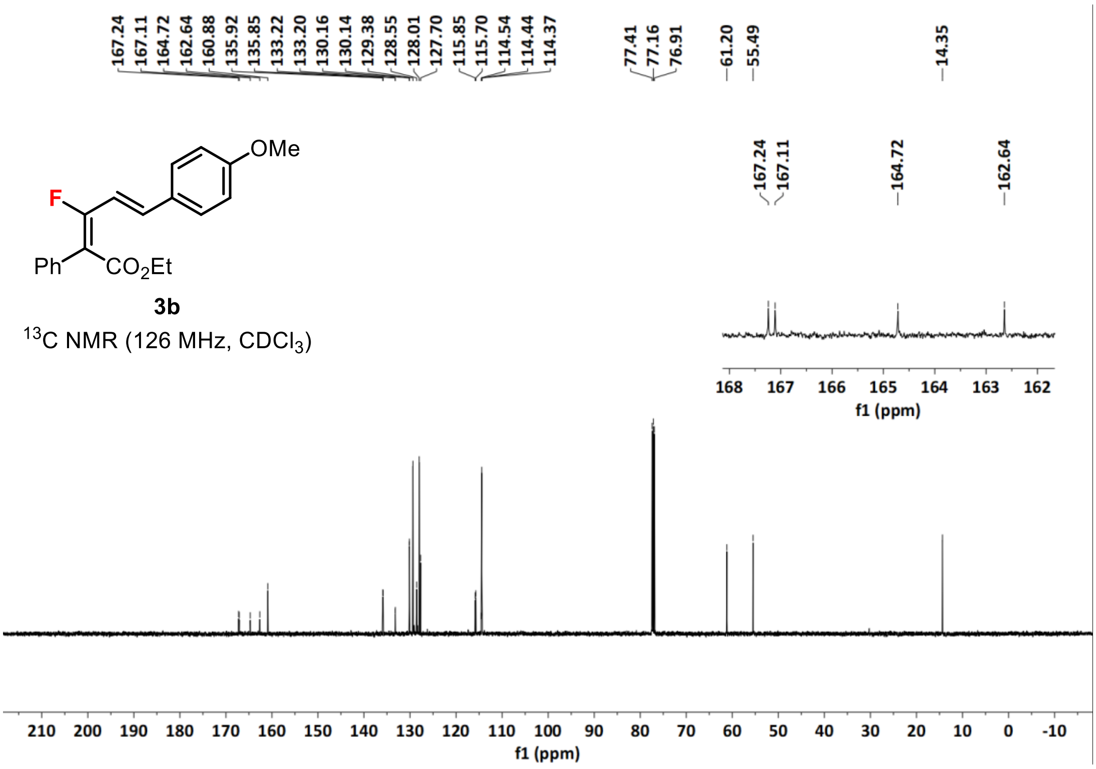
167.24
167.11
164.72
162.64
160.88
135.92
135.85
133.22
133.20
130.16
130.14
129.38
128.55
128.01
127.70
115.85
115.70
114.54
114.44
114.37
77.41
77.16
76.91
61.20
55.49
14.35
OMe
F
Ph
CO2Et
3b
13C NMR (126 MHz, CDCl3)
f1 (ppm)

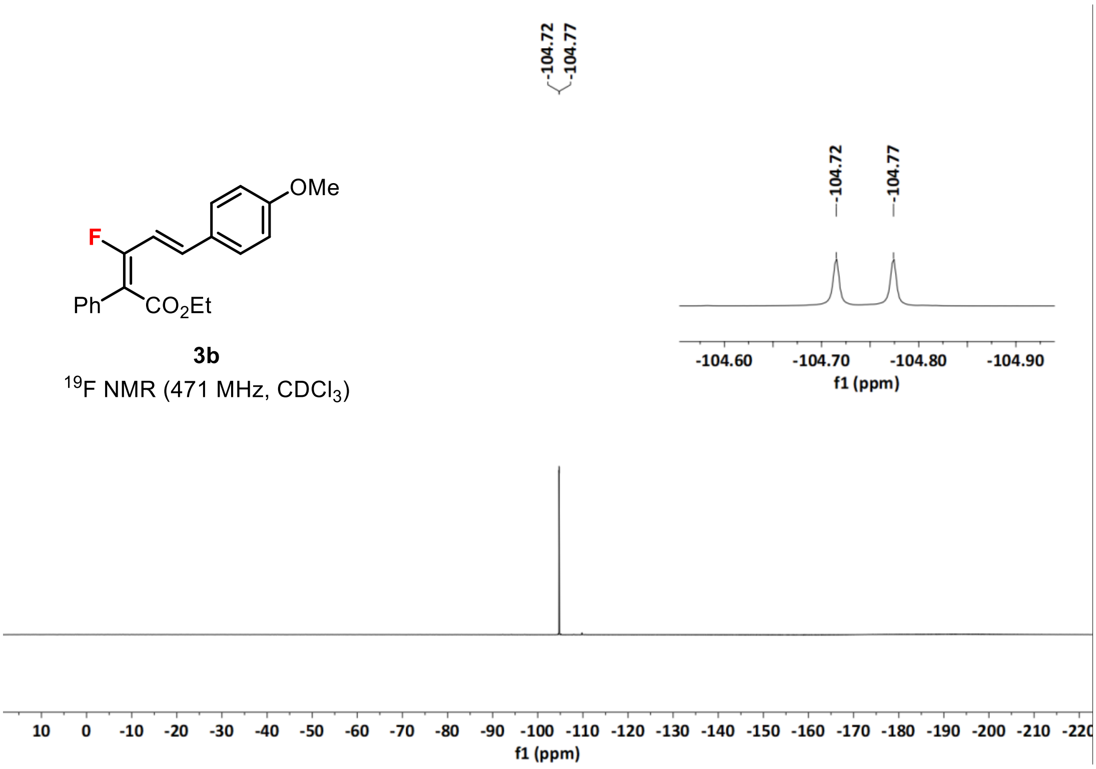
-104.72
-104.77
OMe
F
Ph
CO2Et
3b
19F NMR (471 MHz, CDCl3)
f1 (ppm)

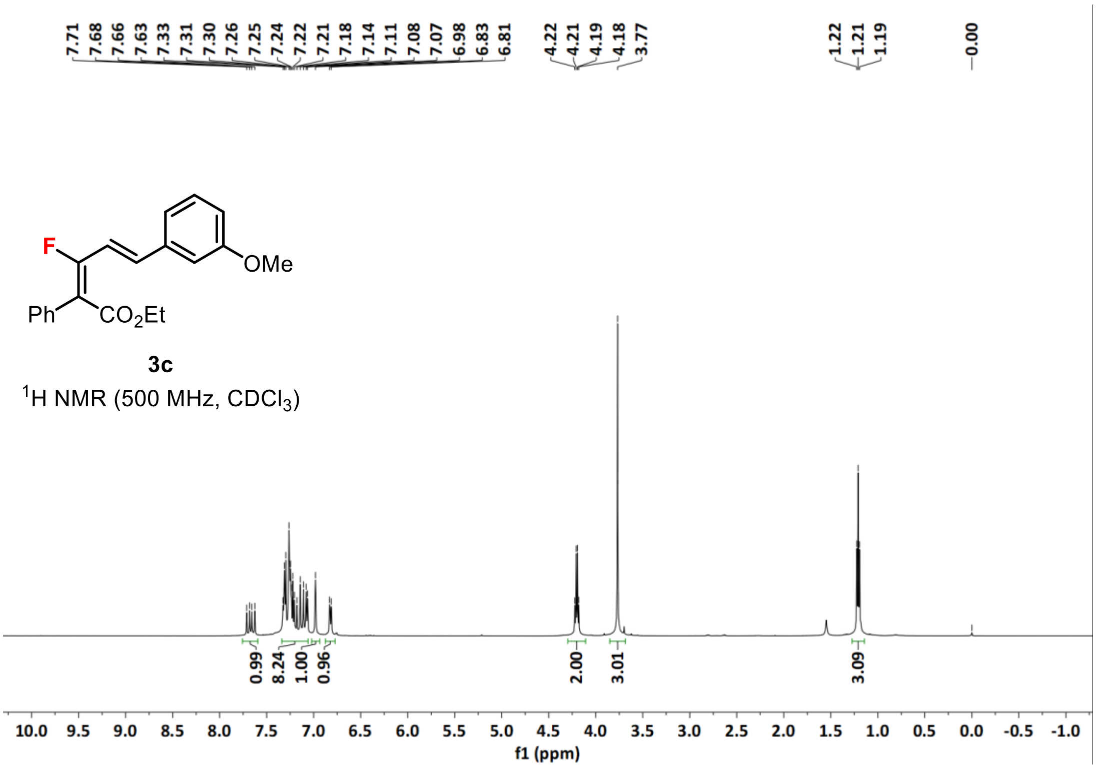
7.71
7.68
7.66
7.63
7.33
7.31
7.30
7.26
7.25
7.24
7.22
7.21
7.18
7.14
7.11
7.08
7.07
6.98
6.83
6.81
4.22
4.21
4.19
4.18
3.77
1.22
1.21
1.19
0.00
F
OMe
Ph
CO2Et
3c
1H NMR (500 MHz, CDCl3)
0.99
8.24
1.00
0.96
2.00
3.01
3.09
f1 (ppm)

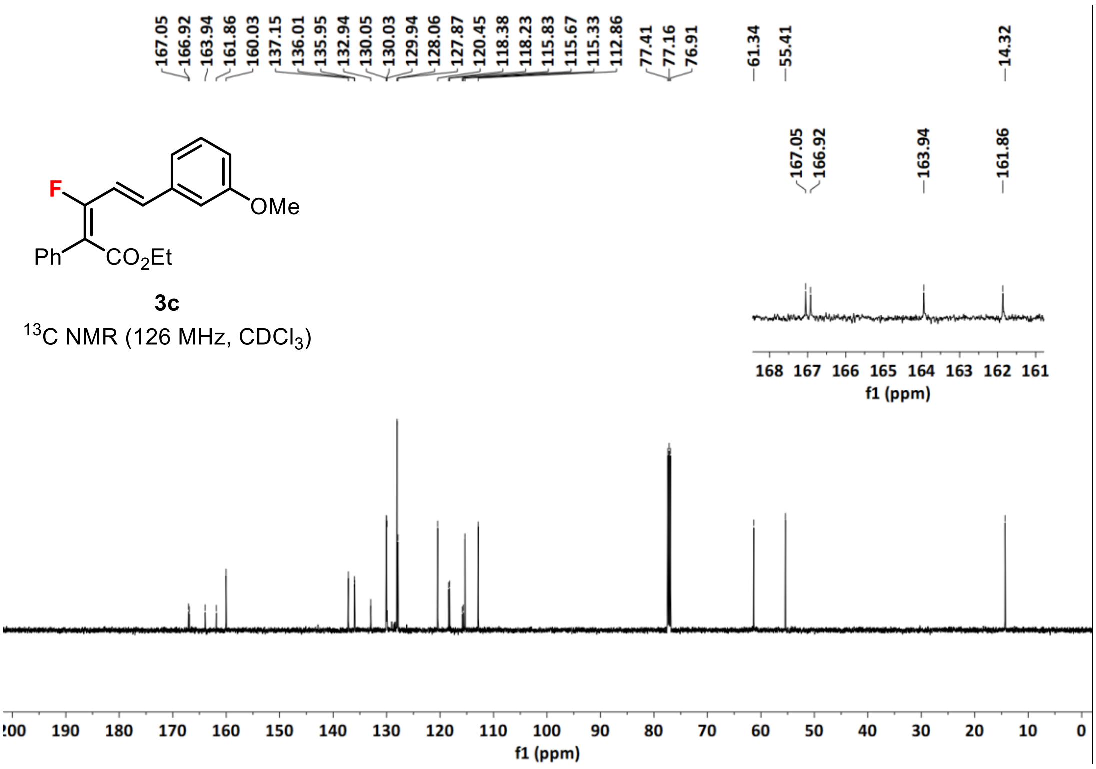
167.05
166.92
163.94
161.86
160.03
137.15
136.01
135.95
132.94
130.05
130.03
129.94
128.06
127.87
120.45
118.38
118.23
115.83
115.67
115.33
112.86
77.41
77.16
76.91
61.34
55.41
14.32
F
OMe
Ph
CO2Et
3c
13C NMR (126 MHz, CDCl3)
167.05
166.92
163.94
161.86
f1 (ppm)
f1 (ppm)

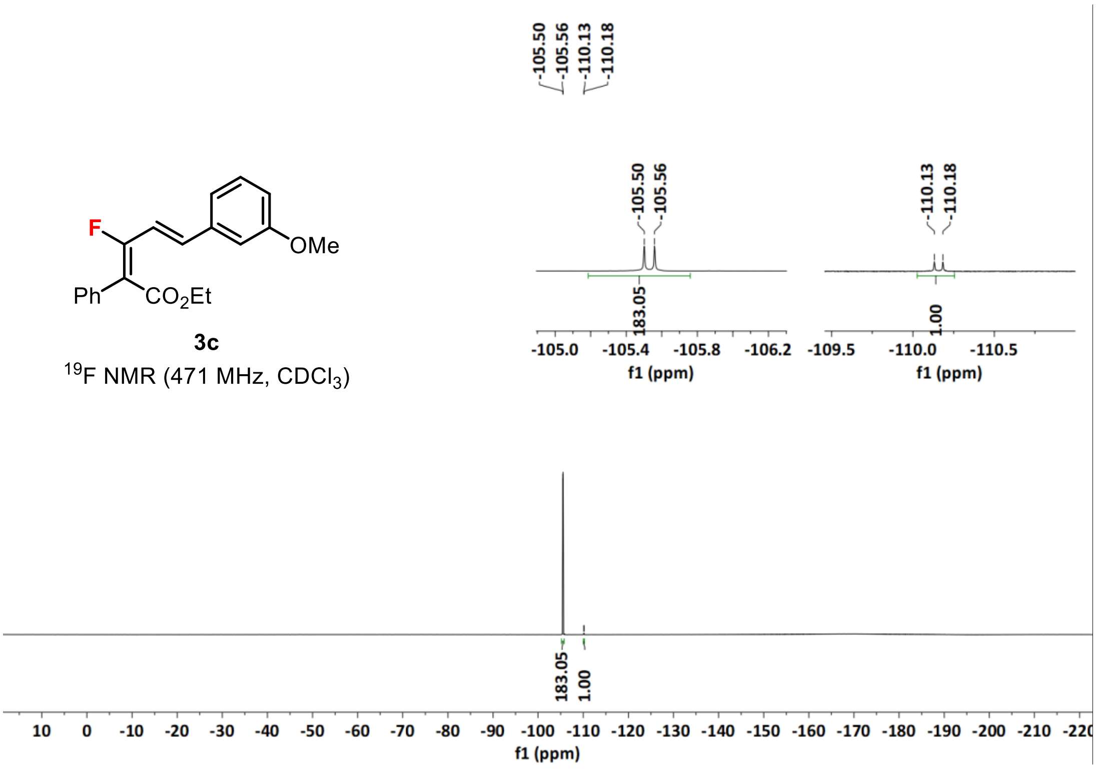

3c

^{19}F NMR (471 MHz, $CDCl_3$)

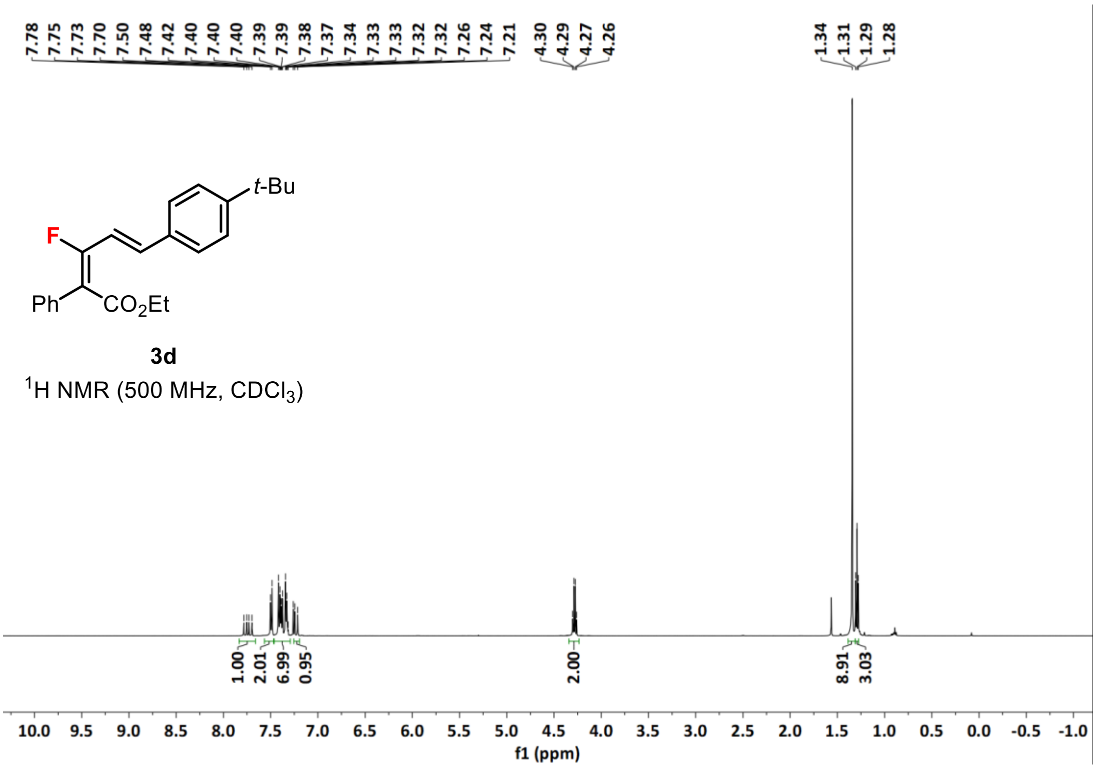

3d

^{1}H NMR (500 MHz, $CDCl_3$)

3d

^{13}C NMR (126 MHz, $CDCl_3$)

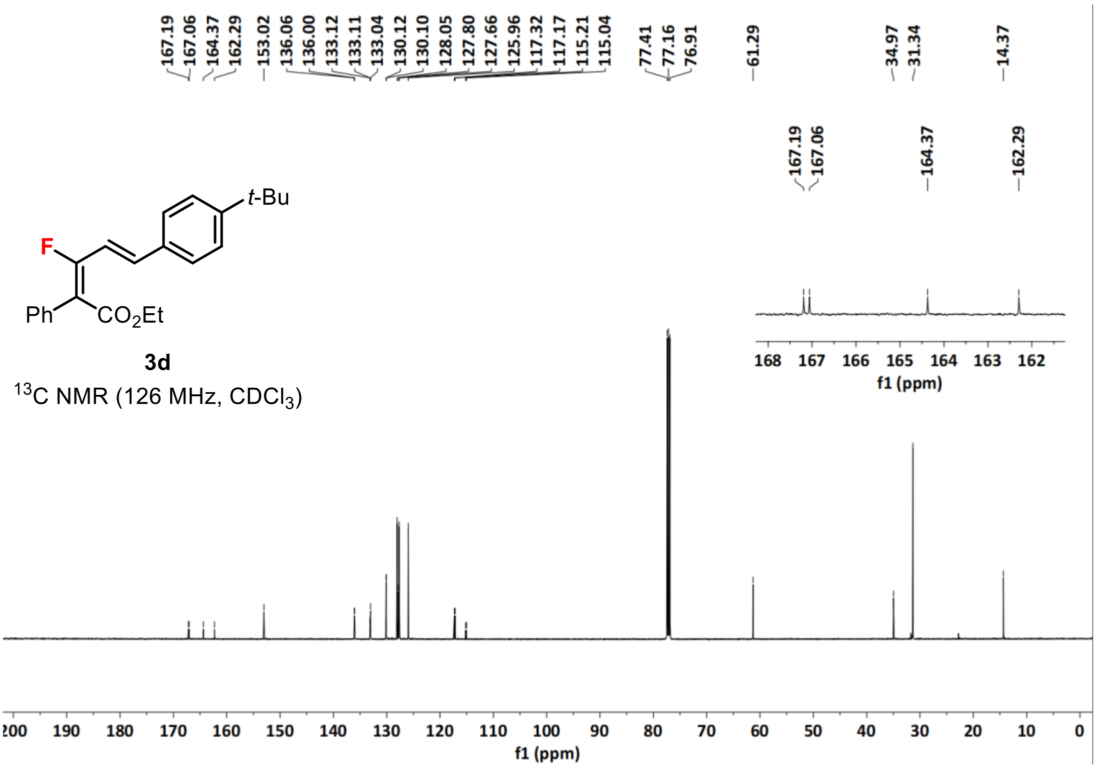

3d

^{19}F NMR (471 MHz, $CDCl_3$)

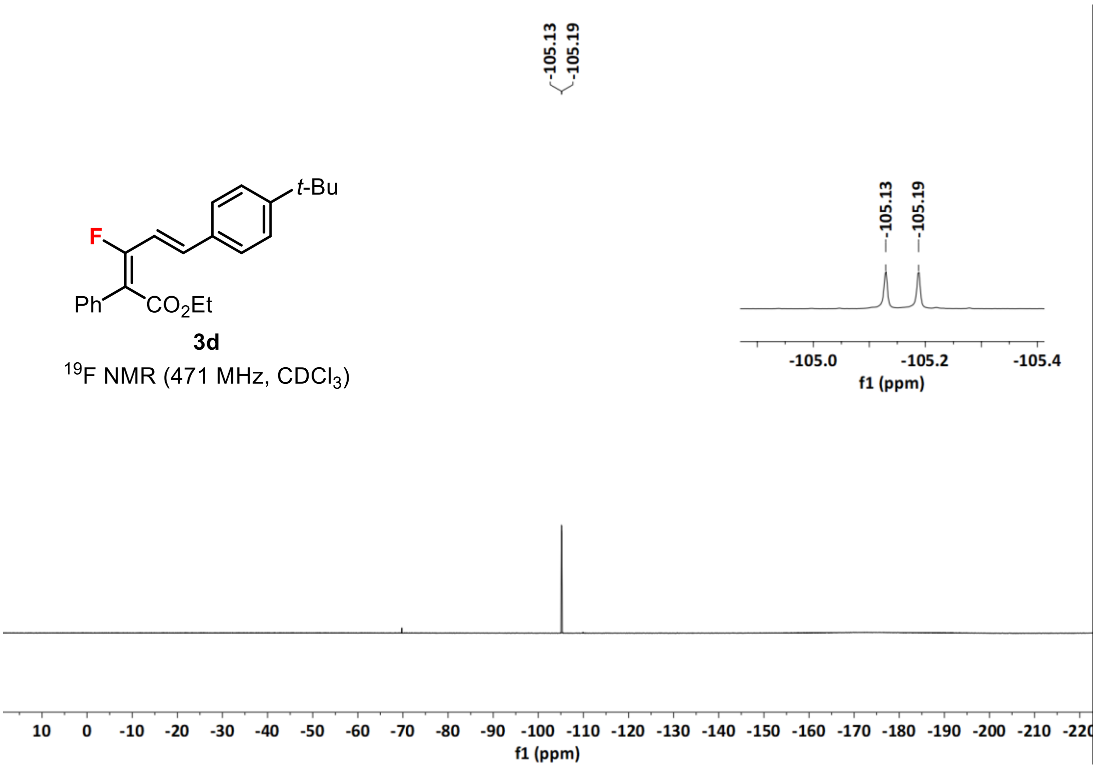

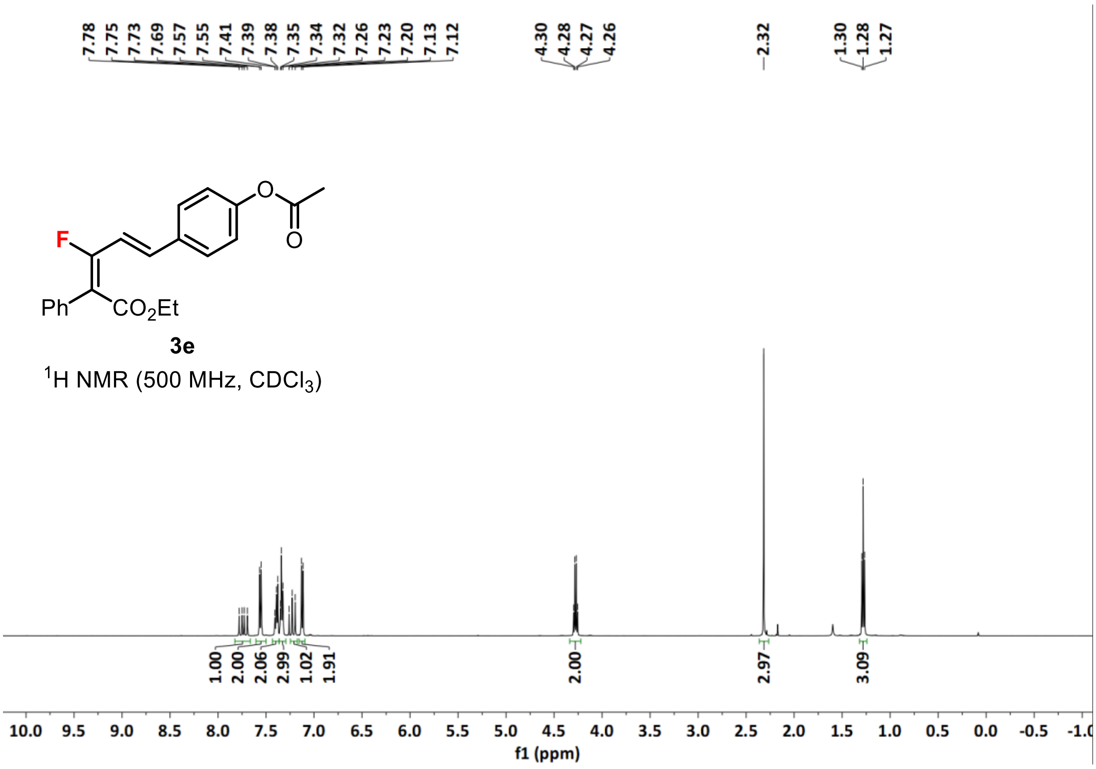
7.78 7.75 7.73 7.69 7.57 7.55 7.41 7.39 7.38 7.35 7.34 7.32 7.26 7.23 7.20 7.13 7.12
4.30 4.28 4.27 4.26
2.32
1.30 1.28 1.27
F
Ph
O
O
CO_2Et
3e
^1H NMR (500 MHz, $CDCl_3$)
1.00 2.00 2.06 2.99 1.02 1.91
2.00
2.97
3.09
10.0 9.5 9.0 8.5 8.0 7.5 7.0 6.5 6.0 5.5 5.0 4.5 4.0 3.5 3.0 2.5 2.0 1.5 1.0 0.5 0.0 -0.5 -1.0
f1 (ppm)

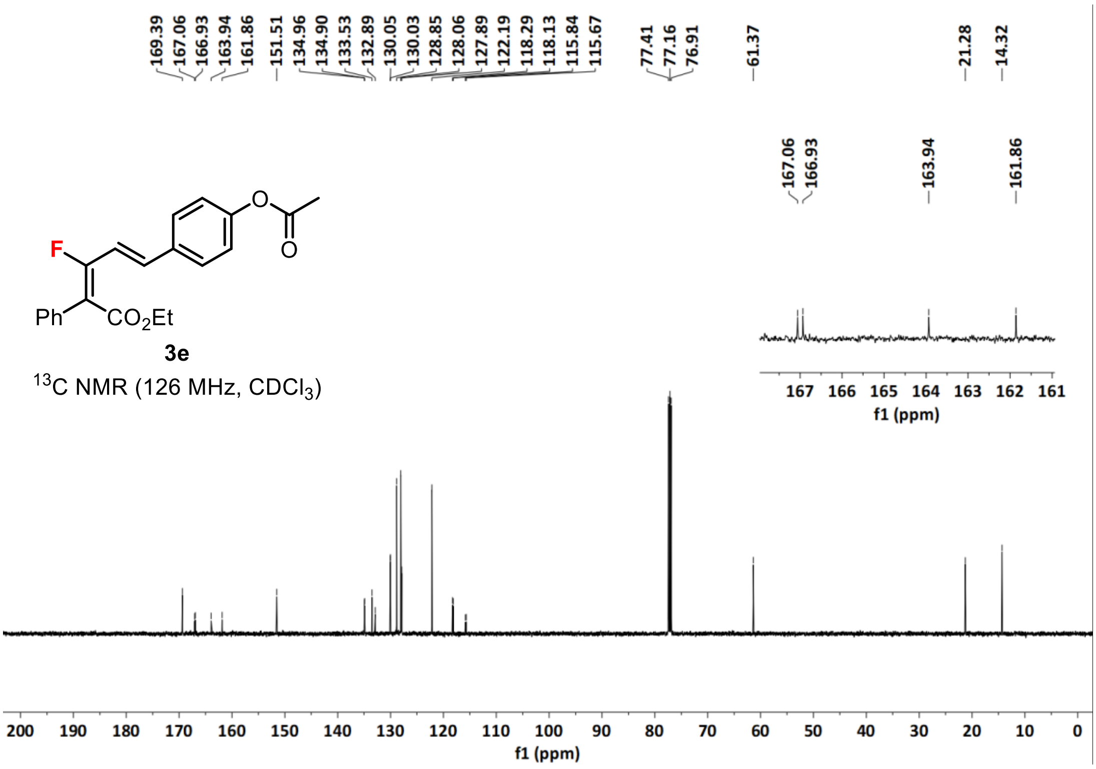
169.39 167.06 166.93 163.94 161.86 151.51 134.96 134.90 133.53 132.89 130.05 130.03 128.85 128.06 127.89 122.19 118.29 118.13 115.84 115.67
77.41 77.16 76.91
61.37
21.28 14.32
167.06 166.93 163.94 161.86
167 166 165 164 163 162 161
f1 (ppm)
F
Ph
O
O
CO_2Et
3e
^{13}C NMR (126 MHz, $CDCl_3$)
200 190 180 170 160 150 140 130 120 110 100 90 80 70 60 50 40 30 20 10 0
f1 (ppm)

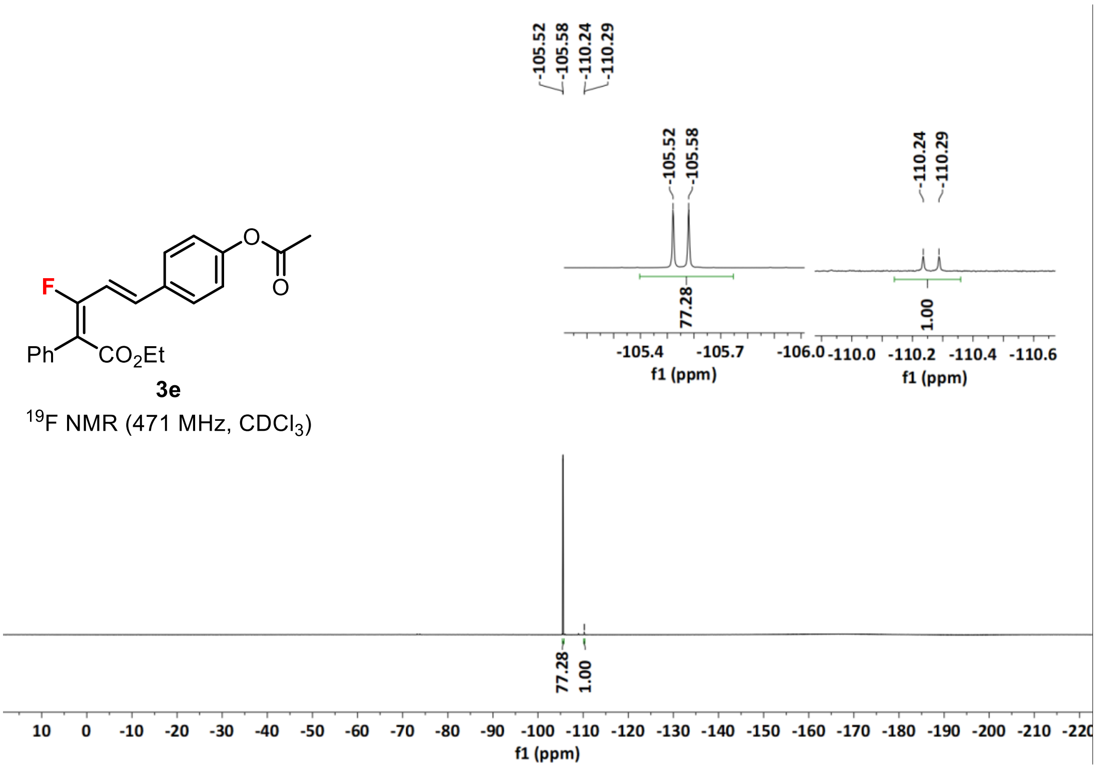

3e

^{19}F NMR (471 MHz, $CDCl_3$)

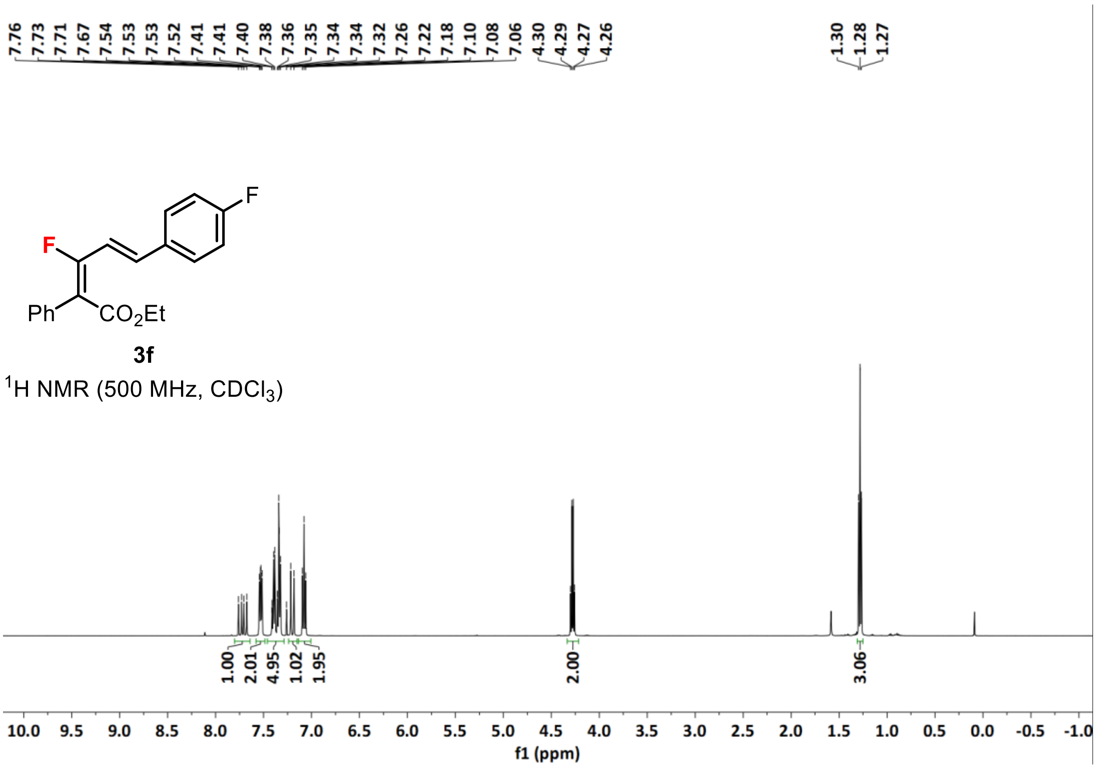

3f

^{1}H NMR (500 MHz, $CDCl_3$)

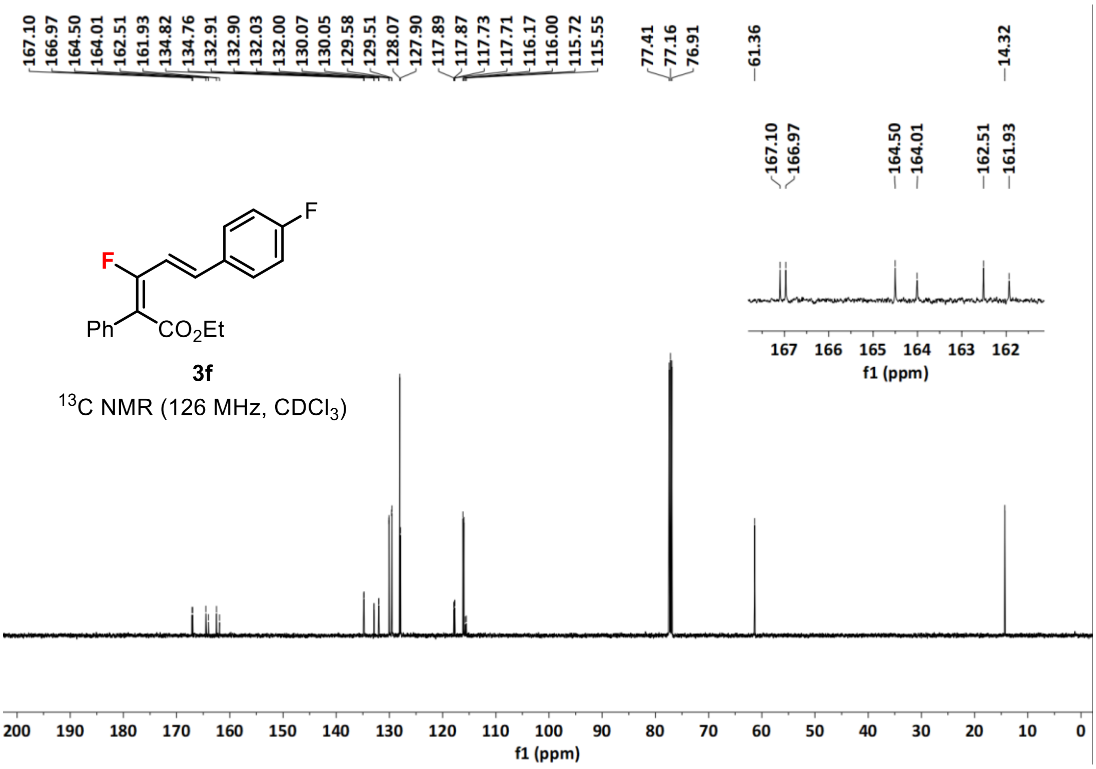
3f
^{13}C NMR (126 MHz, CDCl$_3$)
f1 (ppm)

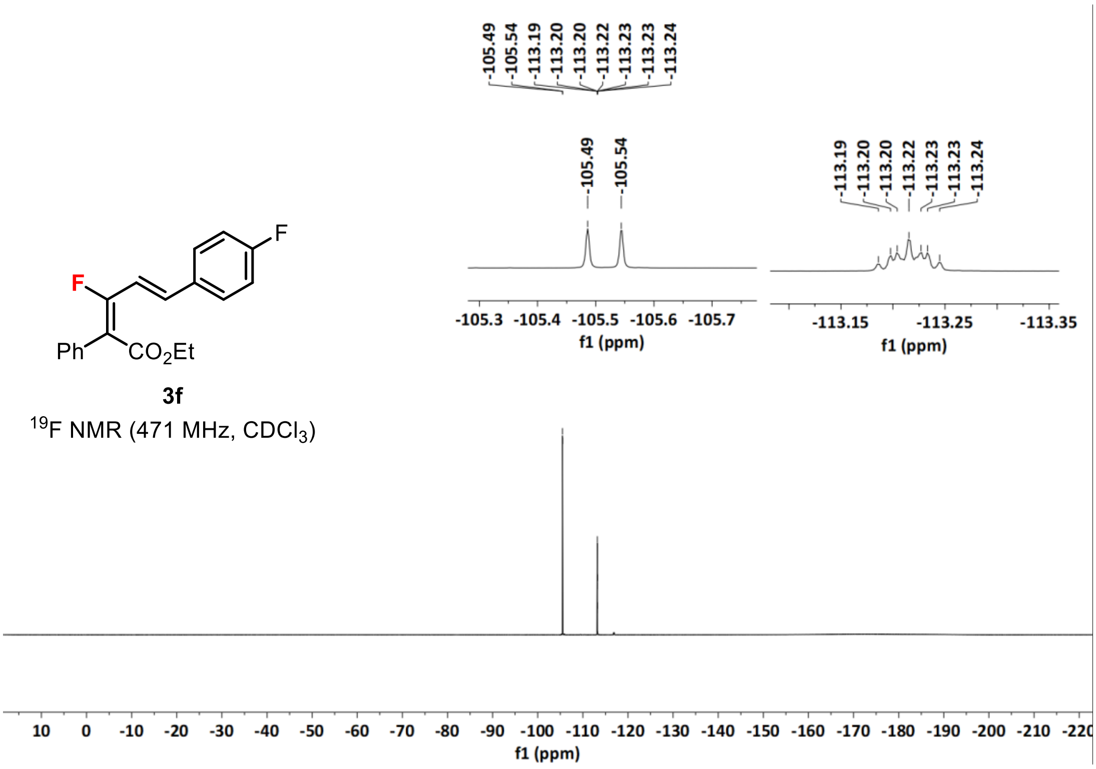
3f
^{19}F NMR (471 MHz, CDCl$_3$)
f1 (ppm)

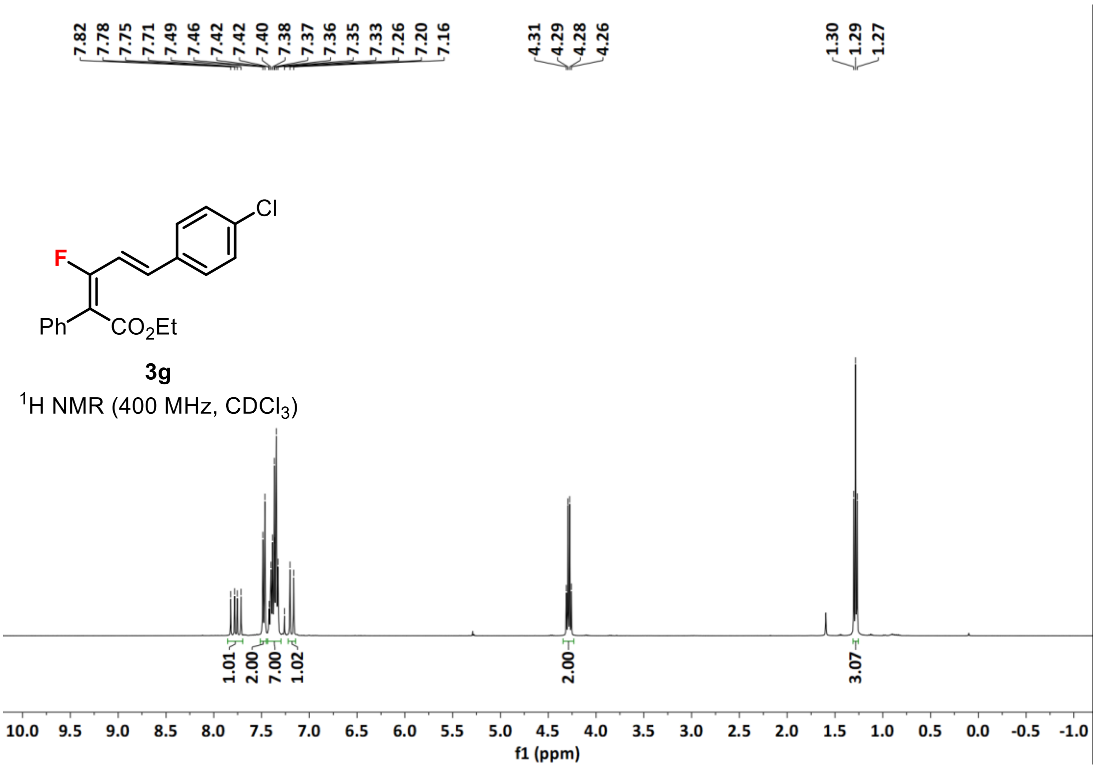
7.82 7.78 7.75 7.71 7.49 7.46 7.42 7.42 7.40 7.38 7.37 7.36 7.35 7.33 7.26 7.20 7.16
4.31 4.29 4.28 4.26
1.30 1.29 1.27
Cl
F
Ph
CO_2Et
3g
^1H NMR (400 MHz, $CDCl_3$)
1.01 2.00 7.00 1.02
2.00
3.07
10.0 9.5 9.0 8.5 8.0 7.5 7.0 6.5 6.0 5.5 5.0 4.5 4.0 3.5 3.0 2.5 2.0 1.5 1.0 0.5 0.0 -0.5 -1.0
f1 (ppm)

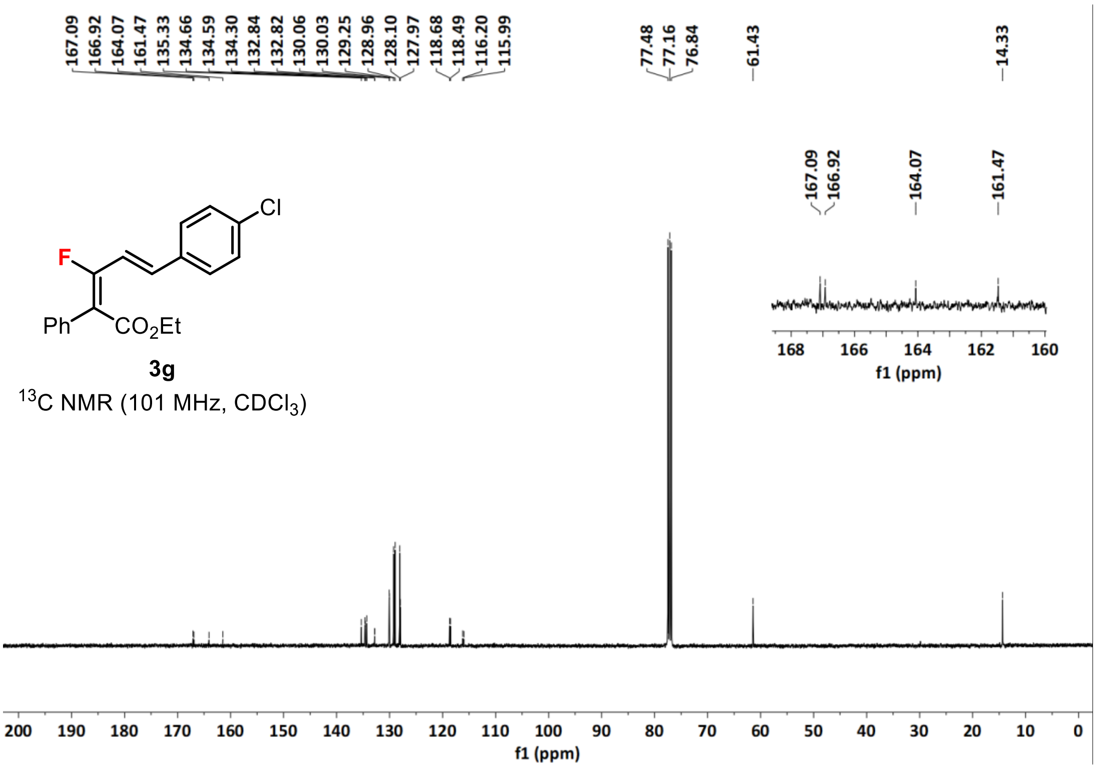
167.09 166.92 164.07 161.47 135.33 134.66 134.59 134.30 132.84 132.82 130.06 130.03 129.25 128.96 128.10 127.97 118.68 118.49 116.20 115.99
77.48 77.16 76.84
61.43
14.33
167.09 166.92
164.07
161.47
168 166 164 162 160
f1 (ppm)
Cl
F
Ph
CO_2Et
3g
^{13}C NMR (101 MHz, $CDCl_3$)
200 190 180 170 160 150 140 130 120 110 100 90 80 70 60 50 40 30 20 10 0
f1 (ppm)

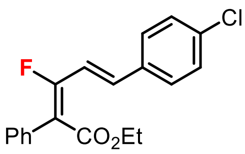

3g

^{19}F NMR (471 MHz, $CDCl_3$)

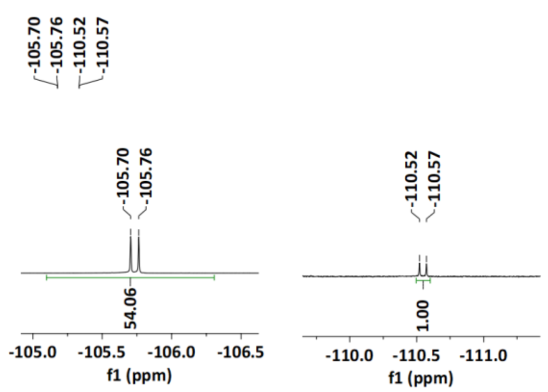

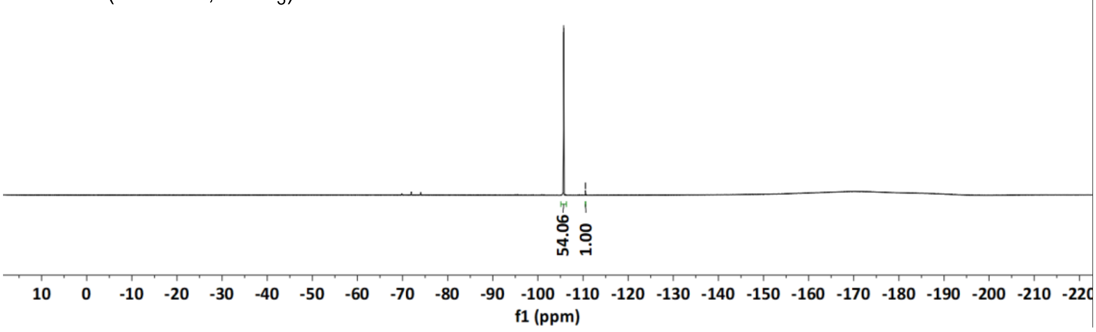

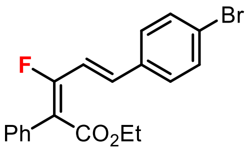

3h

^{1}H NMR (500 MHz, $CDCl_3$)

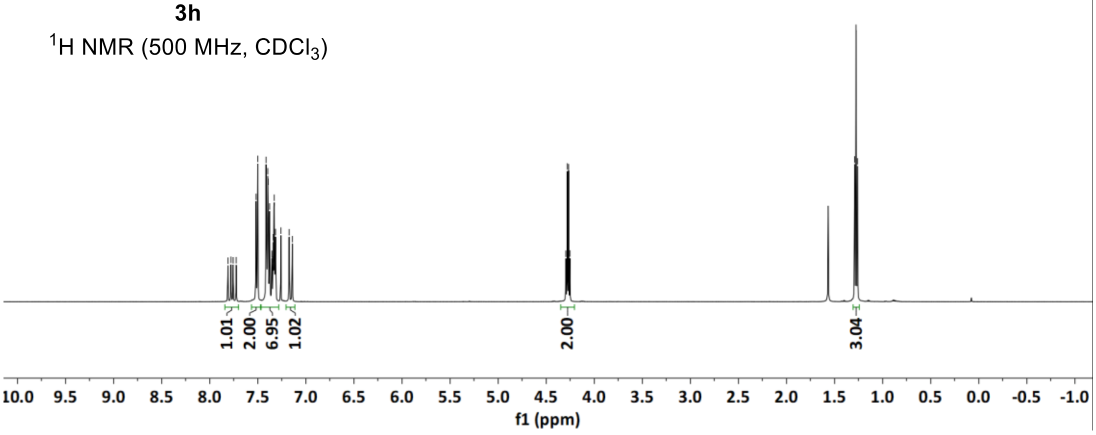

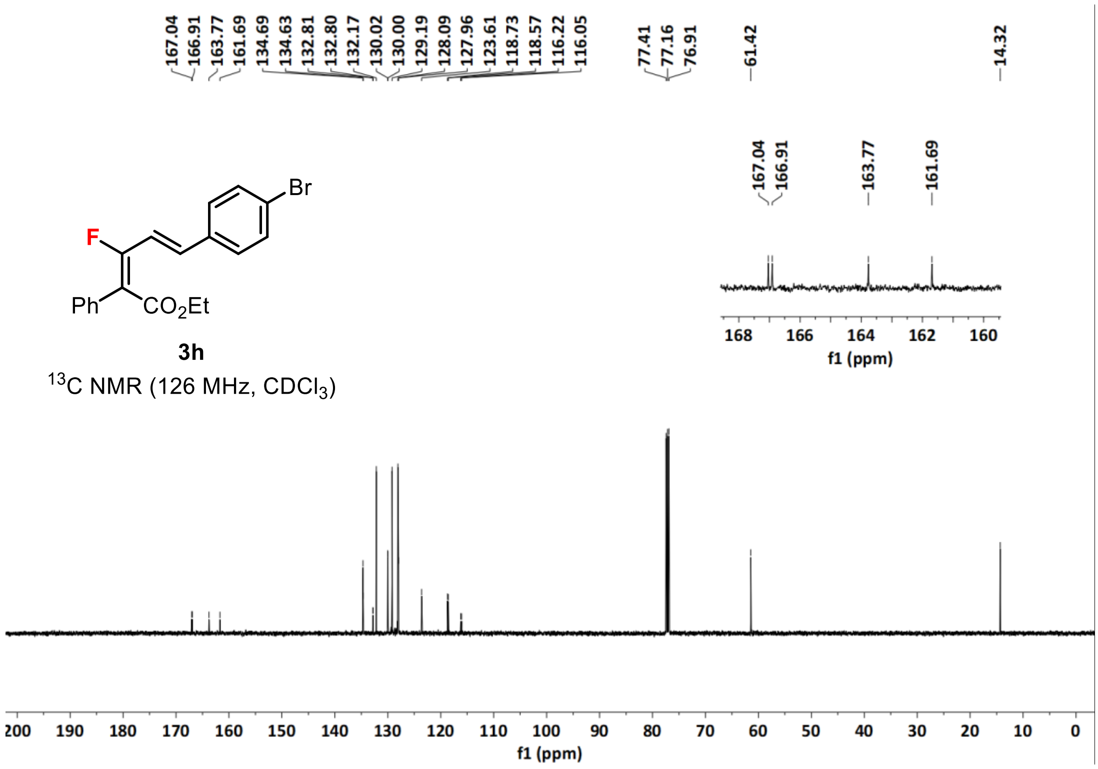
167.04
166.91
163.77
161.69
134.69
134.63
132.81
132.80
132.17
130.02
130.00
129.19
128.09
127.96
123.61
118.73
118.57
116.22
116.05
77.41
77.16
76.91
61.42
14.32
Br
F
Ph
CO_2Et
3h
^{13}C NMR (126 MHz, $CDCl_3$)
f1 (ppm)

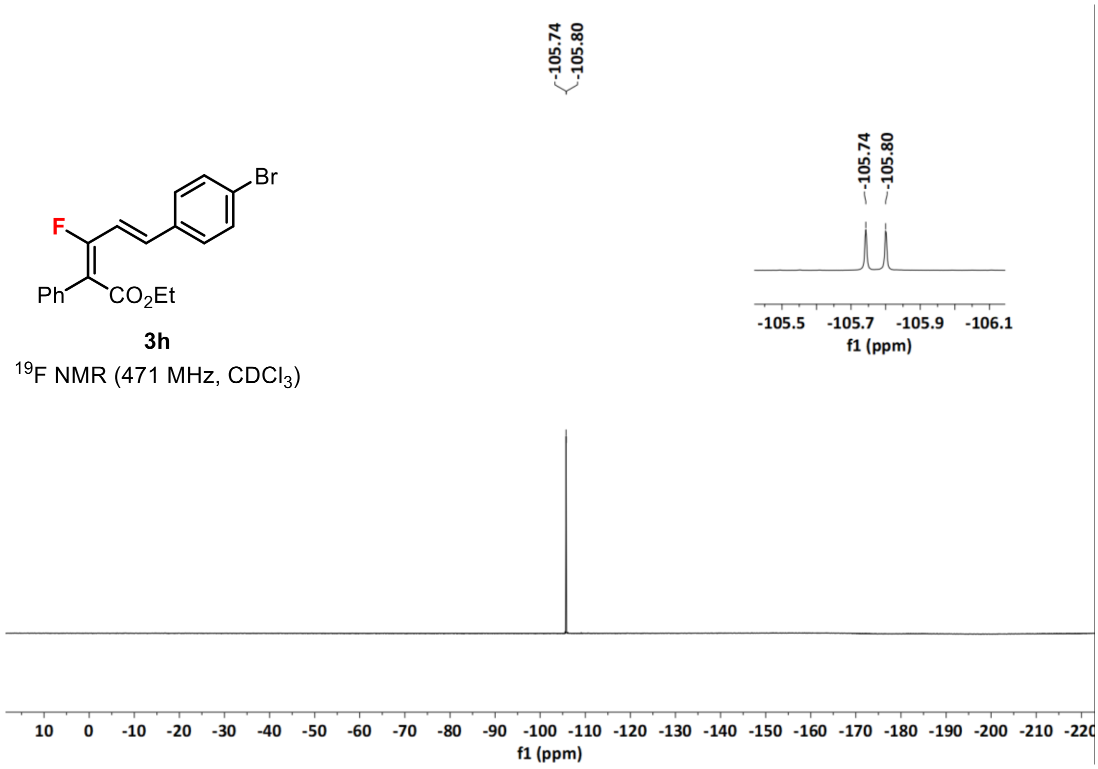
-105.74
-105.80
Br
F
Ph
CO_2Et
3h
^{19}F NMR (471 MHz, $CDCl_3$)
f1 (ppm)

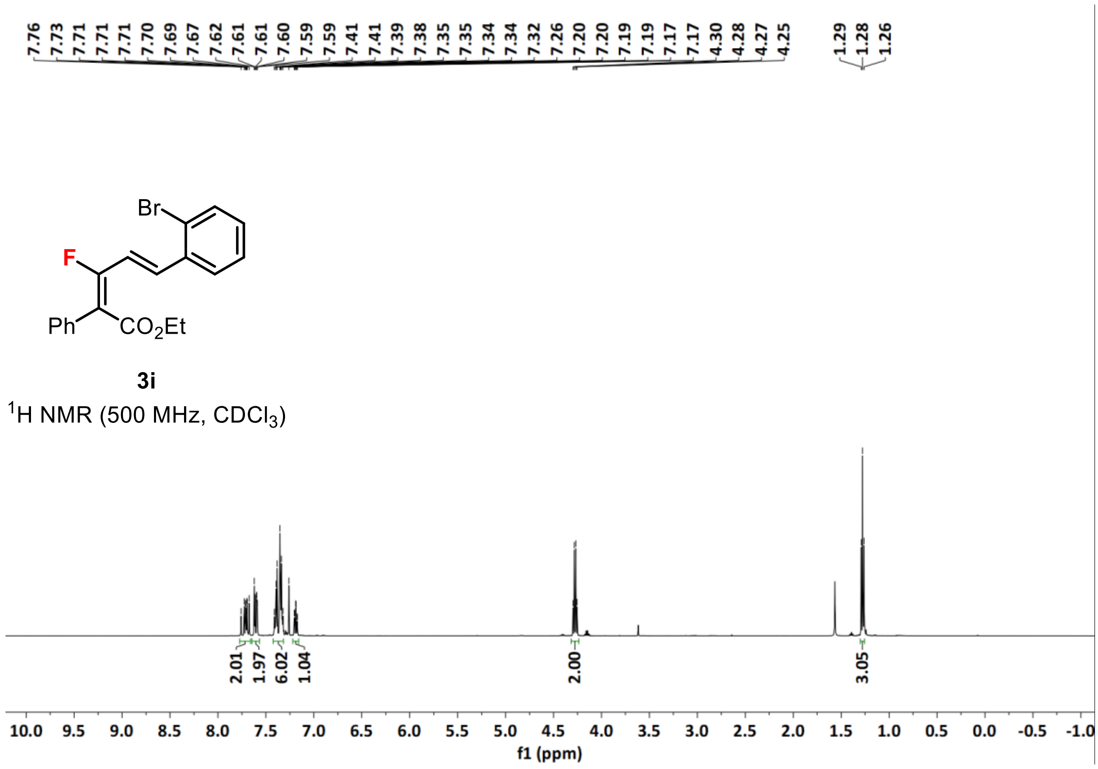
7.76 7.73 7.71 7.71 7.71 7.70 7.69 7.67 7.62 7.61 7.61 7.60 7.59 7.59 7.41 7.41 7.39 7.38 7.35 7.35 7.34 7.34 7.32 7.26 7.20 7.20 7.19 7.19 7.17 7.17 4.30 4.28 4.27 4.25
1.29 1.28 1.26
Br
F
Ph
CO_2Et
3i
^1H NMR (500 MHz, $CDCl_3$)
2.01 1.97 6.02 1.04
2.00
3.05
10.0 9.5 9.0 8.5 8.0 7.5 7.0 6.5 6.0 5.5 5.0 4.5 4.0 3.5 3.0 2.5 2.0 1.5 1.0 0.5 0.0 -0.5 -1.0
f1 (ppm)

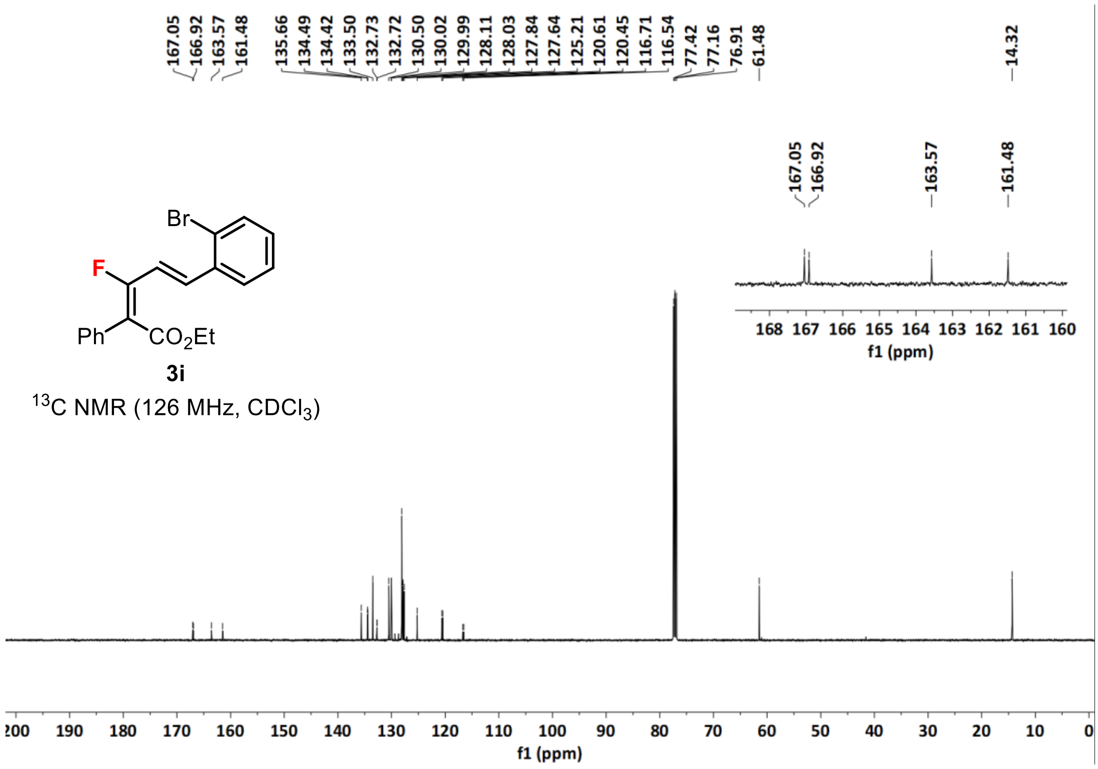
167.05 166.92 163.57 161.48
135.66 134.49 134.42 133.50 132.73 132.72 130.50 130.02 129.99 128.11 128.03 127.84 127.64 125.21 120.61 120.45 116.71 116.54 77.42 77.16 76.91
61.48
14.32
167.05 166.92
163.57
161.48
168 167 166 165 164 163 162 161 160
f1 (ppm)
Br
F
Ph
CO_2Et
3i
^{13}C NMR (126 MHz, $CDCl_3$)
200 190 180 170 160 150 140 130 120 110 100 90 80 70 60 50 40 30 20 10 0
f1 (ppm)

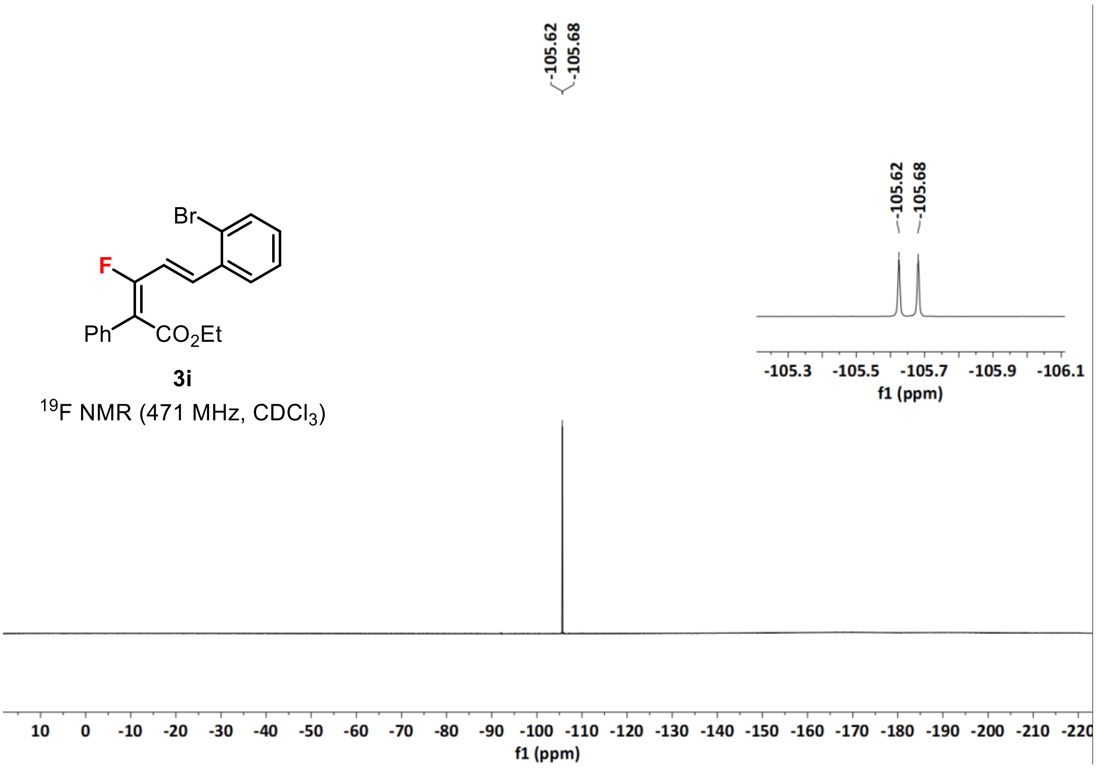
-105.62
-105.68
Br
F
Ph
CO2Et
3i
19F NMR (471 MHz, CDCl3)
f1 (ppm)

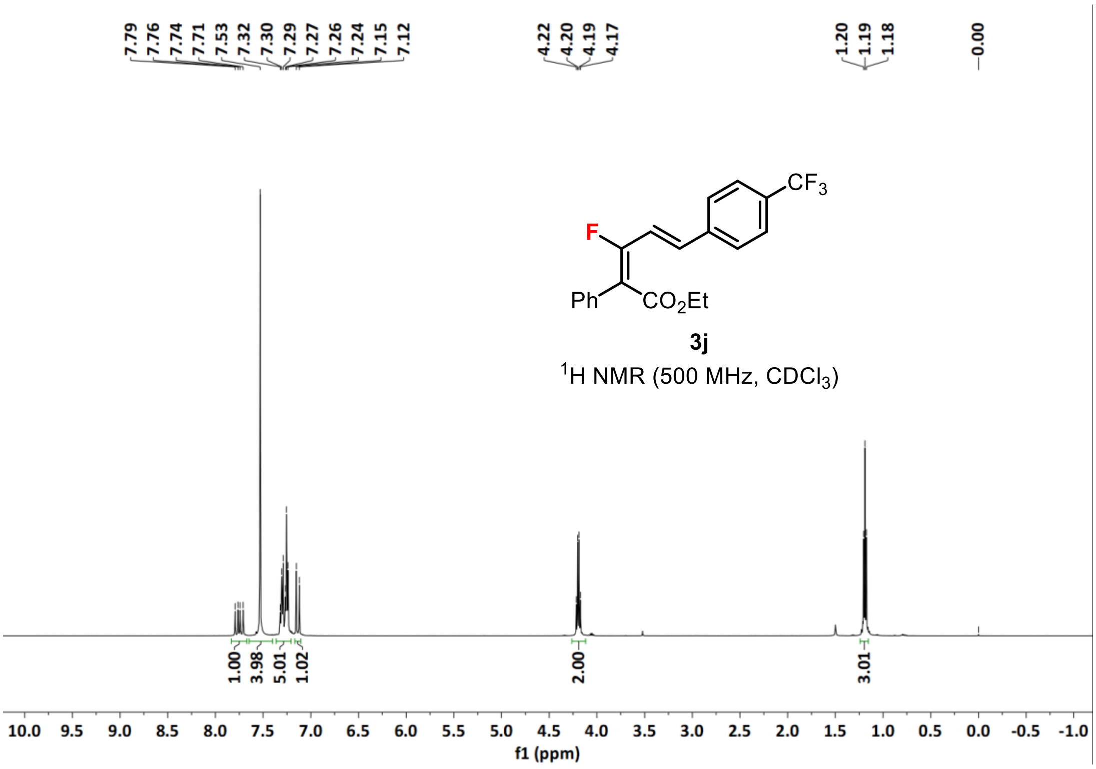
7.79
7.76
7.74
7.71
7.53
7.32
7.30
7.29
7.27
7.26
7.24
7.15
7.12
4.22
4.20
4.19
4.17
1.20
1.19
1.18
0.00
CF3
F
Ph
CO2Et
3j
1H NMR (500 MHz, CDCl3)
1.00
3.98
5.01
1.02
2.00
3.01
f1 (ppm)

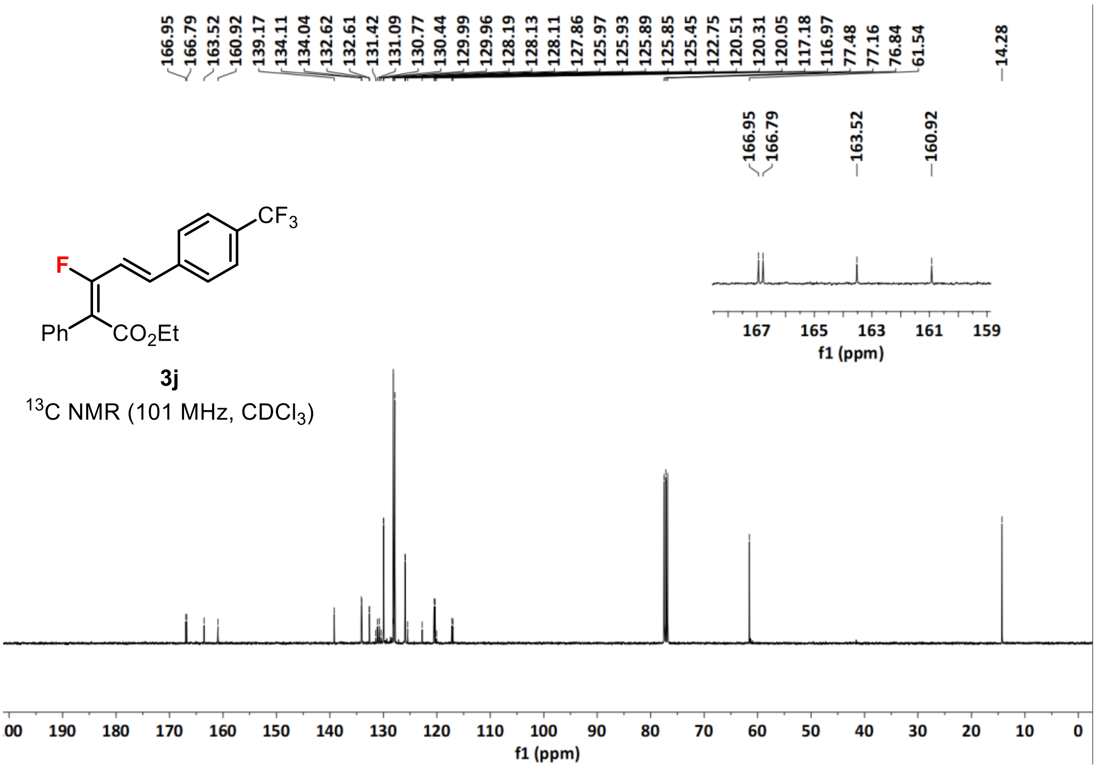

3j

^{13}C NMR (101 MHz, $CDCl_3$)

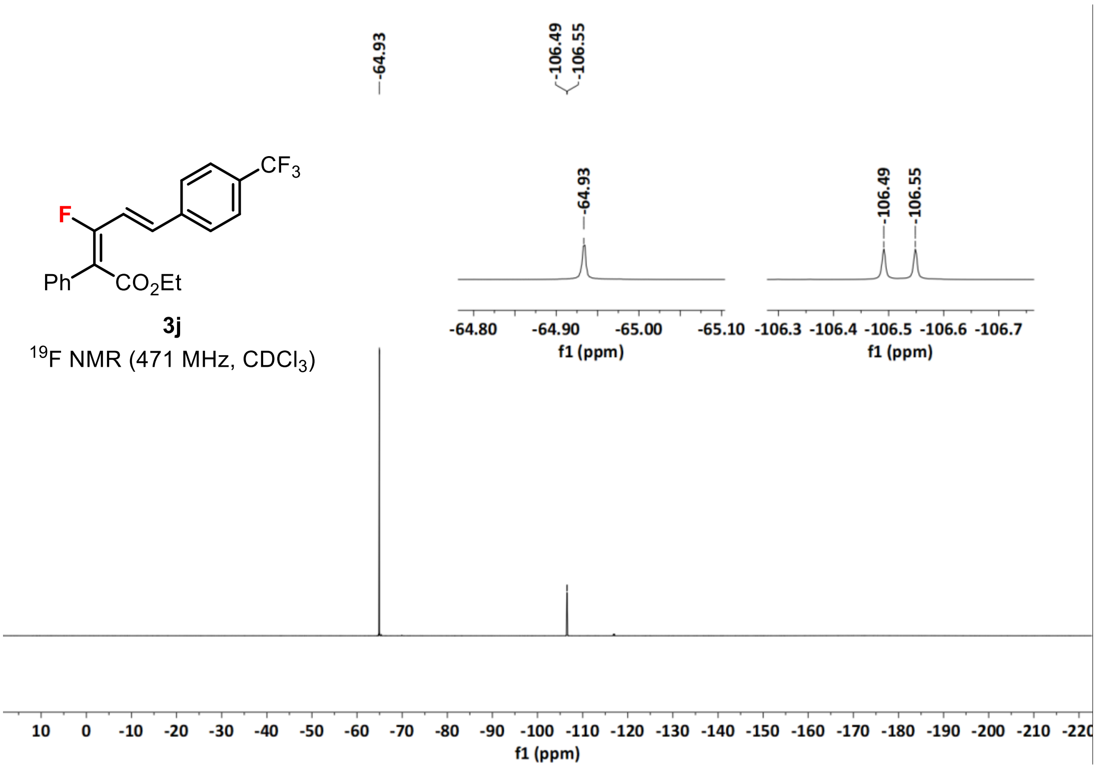

3j

^{19}F NMR (471 MHz, $CDCl_3$)

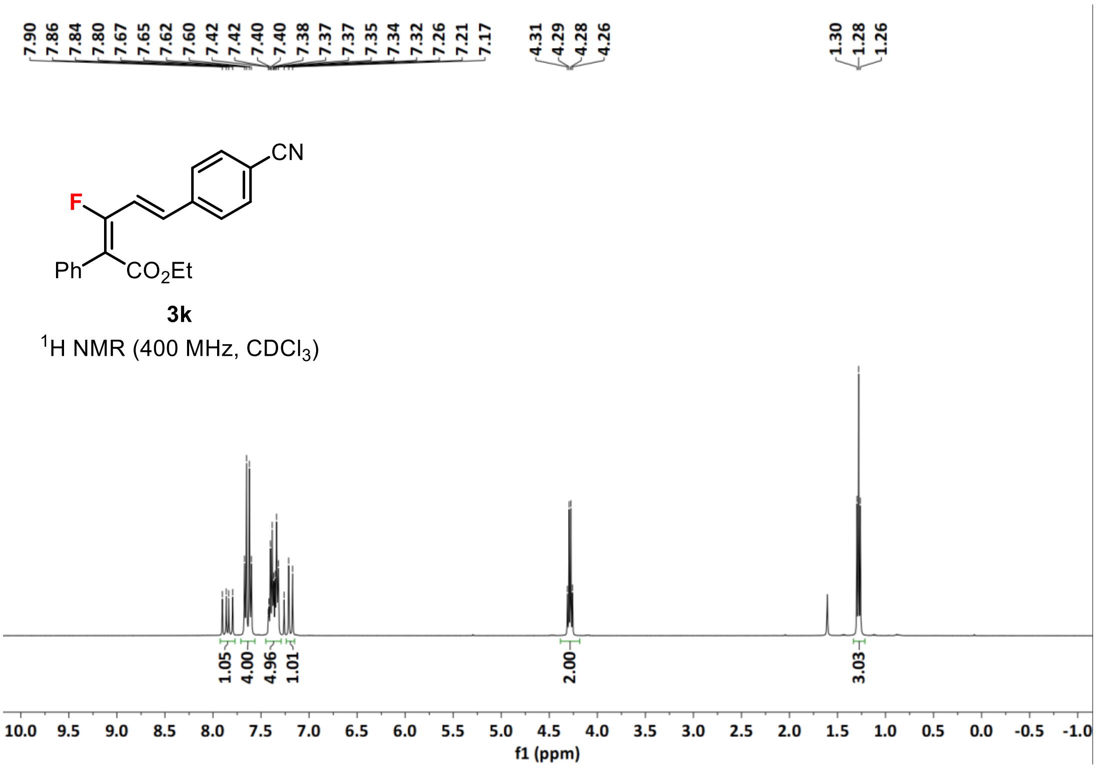
7.90 7.86 7.84 7.80 7.67 7.65 7.62 7.60 7.42 7.42 7.40 7.40 7.38 7.37 7.37 7.35 7.34 7.32 7.26 7.21 7.17
4.31 4.29 4.28 4.26
1.30 1.28 1.26
CN
F
Ph
CO_2Et
3k
^{1}H NMR (400 MHz, $CDCl_3$)
1.05 4.00 4.96 1.01
2.00
3.03
10.0 9.5 9.0 8.5 8.0 7.5 7.0 6.5 6.0 5.5 5.0 4.5 4.0 3.5 3.0 2.5 2.0 1.5 1.0 0.5 0.0 -0.5 -1.0
f1 (ppm)

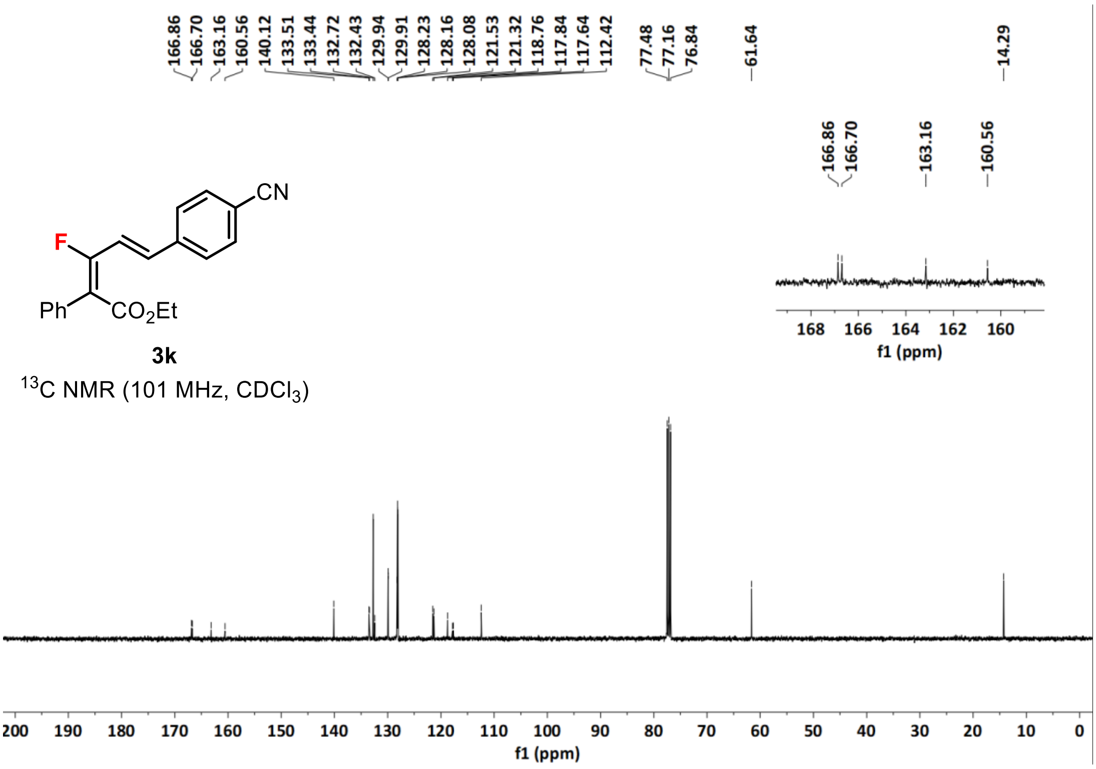
166.86 166.70 163.16 160.56 140.12 133.51 133.44 132.72 132.43 129.94 129.91 128.23 128.16 128.08 121.53 121.32 118.76 117.84 117.64 112.42
77.48 77.16 76.84
61.64
14.29
166.86 166.70
163.16
160.56
168 166 164 162 160
f1 (ppm)
CN
F
Ph
CO_2Et
3k
^{13}C NMR (101 MHz, $CDCl_3$)
200 190 180 170 160 150 140 130 120 110 100 90 80 70 60 50 40 30 20 10 0
f1 (ppm)

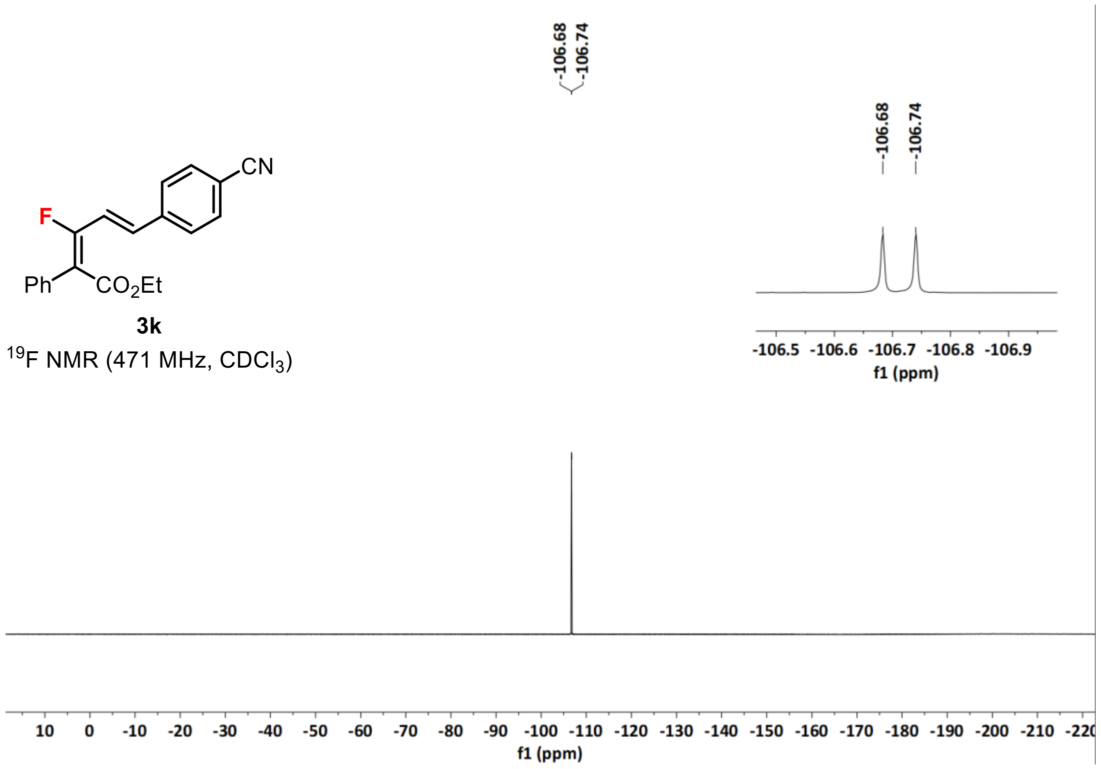
F
CN
Ph
CO2Et
3k
19F NMR (471 MHz, CDCl3)
-106.68
-106.74
-106.5 -106.6 -106.7 -106.8 -106.9
f1 (ppm)
10 0 -10 -20 -30 -40 -50 -60 -70 -80 -90 -100 -110 -120 -130 -140 -150 -160 -170 -180 -190 -200 -210 -220
f1 (ppm)

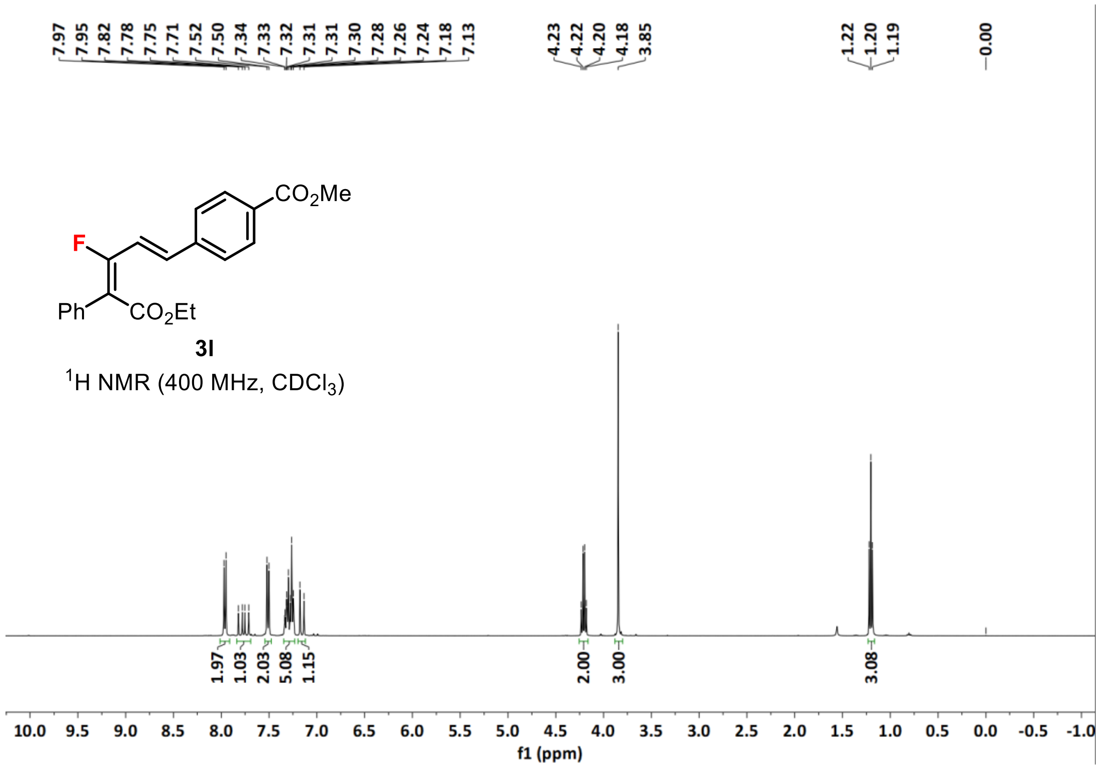
7.97 7.95 7.82 7.78 7.75 7.71 7.52 7.50 7.34 7.33 7.32 7.31 7.31 7.30 7.28 7.26 7.24 7.18 7.13
4.23 4.22 4.20 4.18 3.85
1.22 1.20 1.19
0.00
CO2Me
F
Ph
CO2Et
3l
1H NMR (400 MHz, CDCl3)
1.97 1.03 2.03 5.08 1.15
2.00 3.00
3.08
10.0 9.5 9.0 8.5 8.0 7.5 7.0 6.5 6.0 5.5 5.0 4.5 4.0 3.5 3.0 2.5 2.0 1.5 1.0 0.5 0.0 -0.5 -1.0
f1 (ppm)

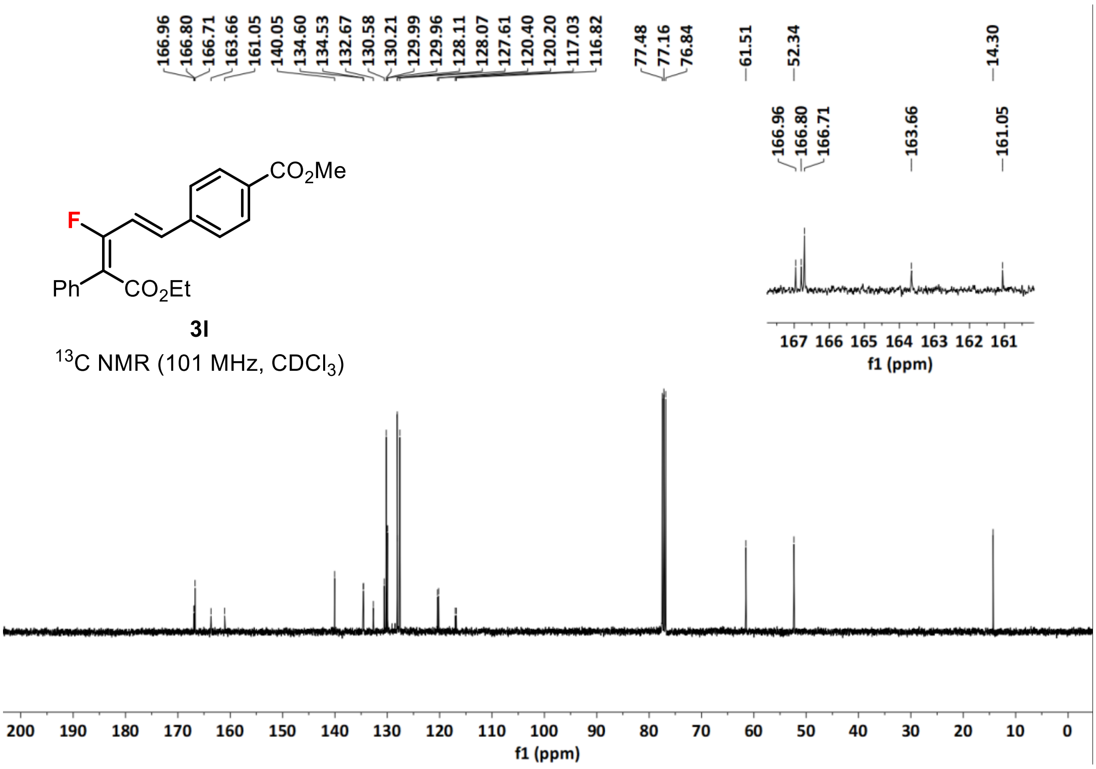
CO2Me
F
Ph
CO2Et
3l
13C NMR (101 MHz, CDCl3)
166.96
166.80
166.71
163.66
161.05
140.05
134.60
134.53
132.67
130.58
130.21
129.99
129.96
128.11
128.07
127.61
120.40
120.20
117.03
116.82
77.48
77.16
76.84
61.51
52.34
14.30
f1 (ppm)

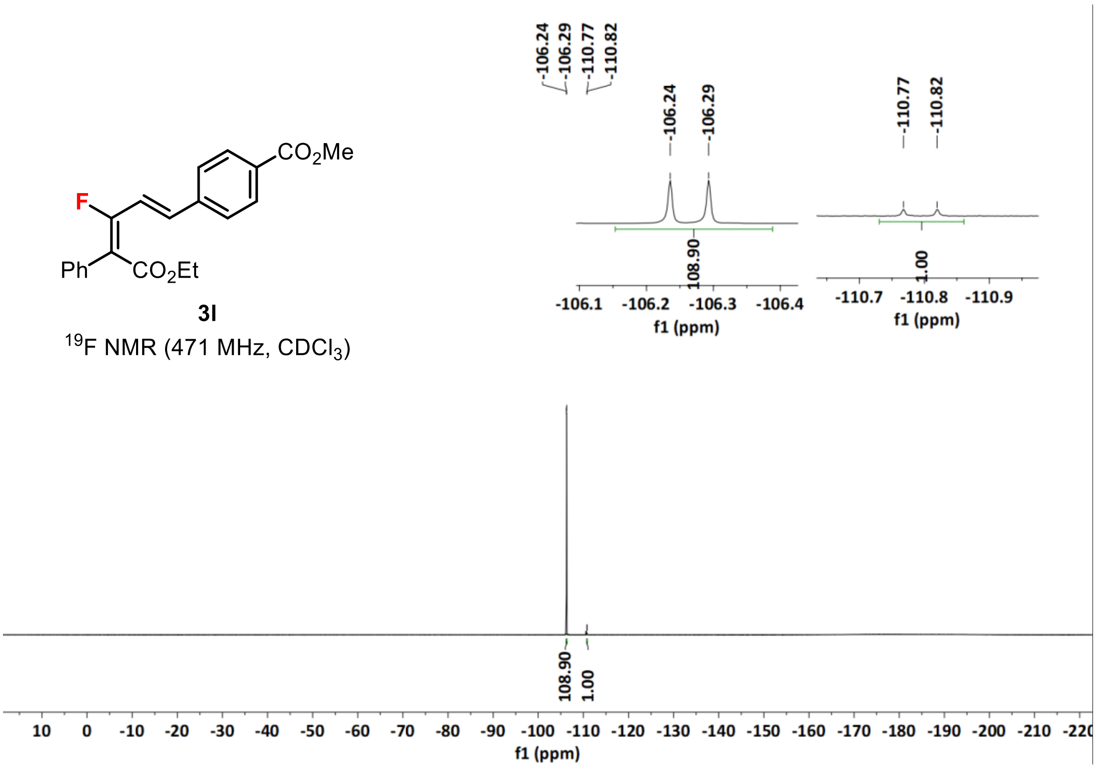
CO2Me
F
Ph
CO2Et
3l
19F NMR (471 MHz, CDCl3)
-106.24
-106.29
-110.77
-110.82
108.90
1.00
f1 (ppm)

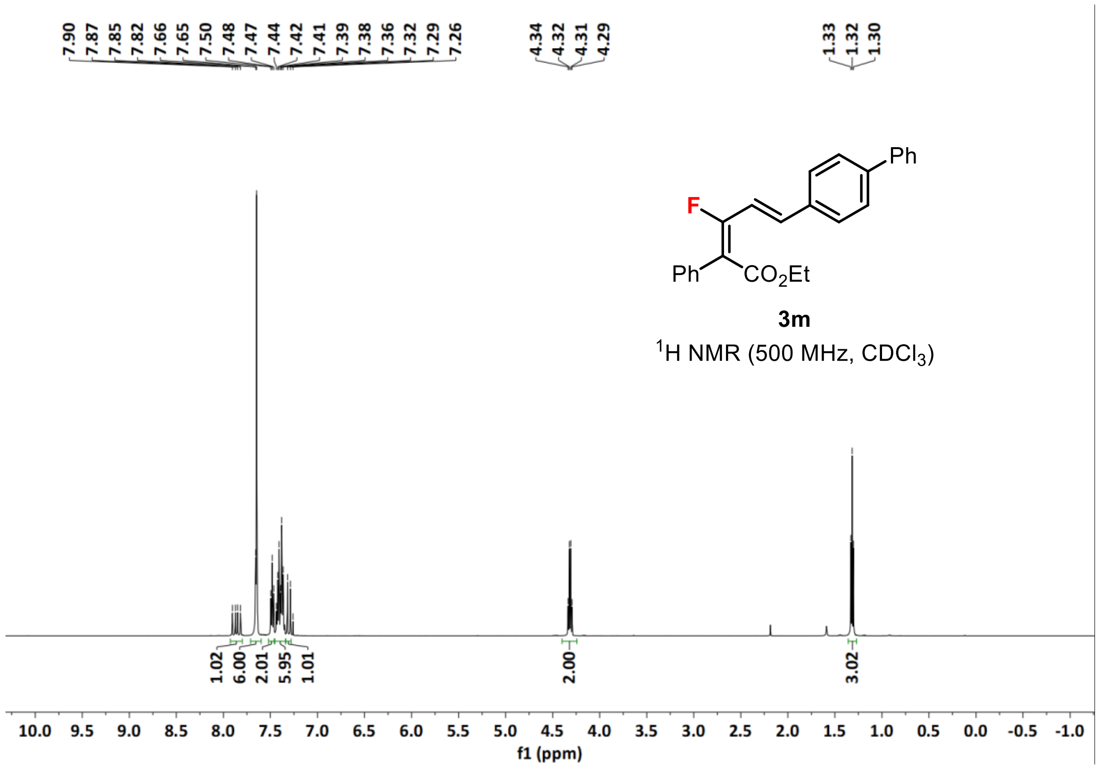
7.90
7.87
7.85
7.82
7.66
7.65
7.50
7.48
7.47
7.44
7.42
7.41
7.39
7.38
7.36
7.32
7.29
7.26
4.34
4.32
4.31
4.29
1.33
1.32
1.30
F
Ph
Ph
CO_2Et
3m
^1H NMR (500 MHz, $CDCl_3$)
1.02
6.00
2.01
5.95
1.01
2.00
3.02
10.0 9.5 9.0 8.5 8.0 7.5 7.0 6.5 6.0 5.5 5.0 4.5 4.0 3.5 3.0 2.5 2.0 1.5 1.0 0.5 0.0 -0.5 -1.0
f1 (ppm)

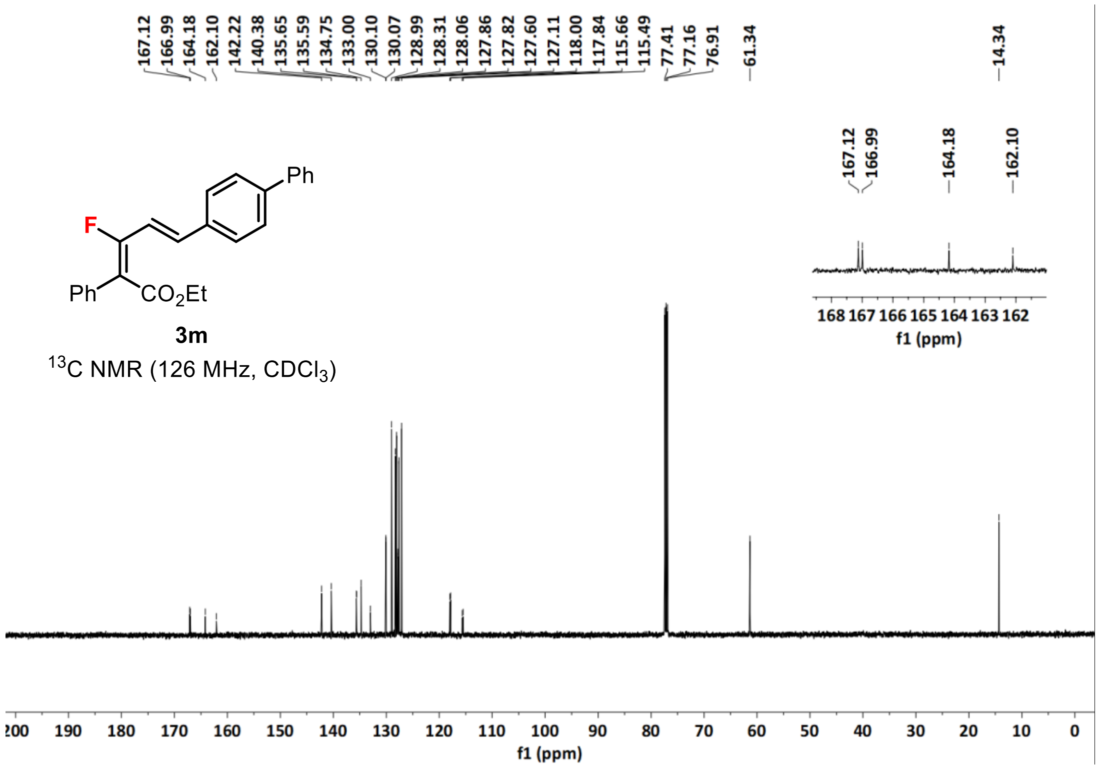
167.12
166.99
164.18
162.10
142.22
140.38
135.65
135.59
134.75
133.00
130.10
130.07
128.99
128.31
128.06
127.86
127.82
127.60
127.11
118.00
117.84
115.66
115.49
77.41
77.16
76.91
61.34
14.34
F
Ph
Ph
CO_2Et
3m
^{13}C NMR (126 MHz, $CDCl_3$)
167.12
166.99
164.18
162.10
168 167 166 165 164 163 162
f1 (ppm)
200 190 180 170 160 150 140 130 120 110 100 90 80 70 60 50 40 30 20 10 0
f1 (ppm)

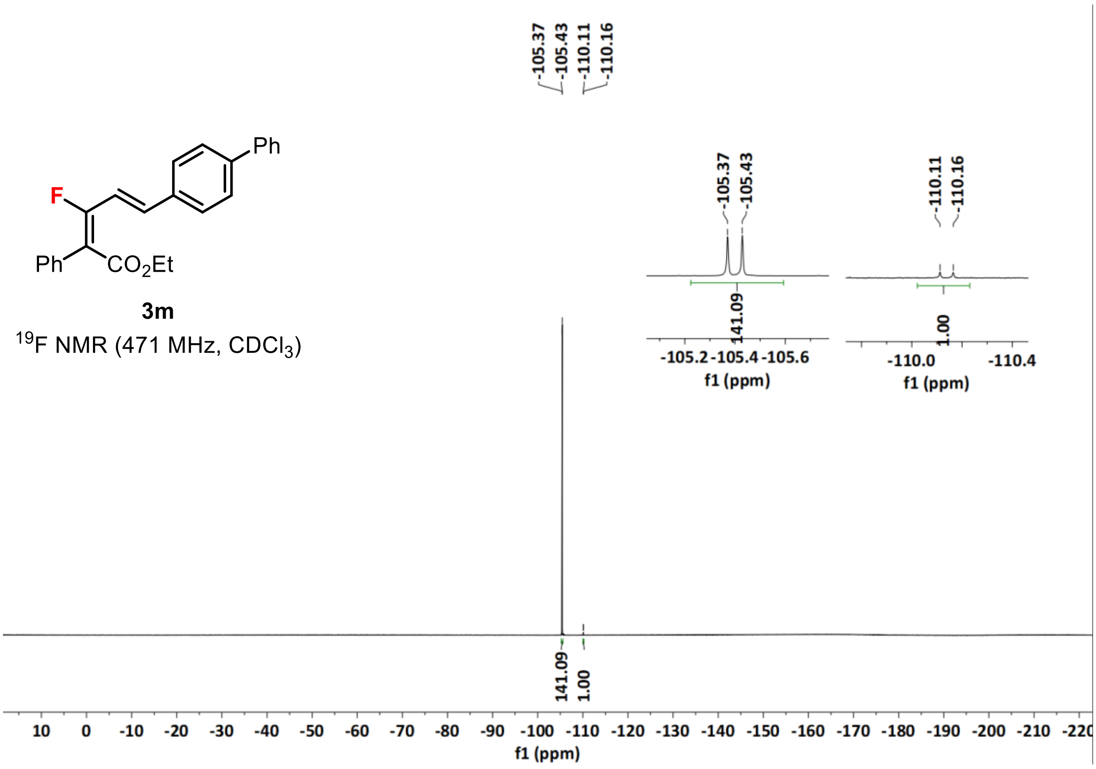

3m

^{19}F NMR (471 MHz, $CDCl_3$)

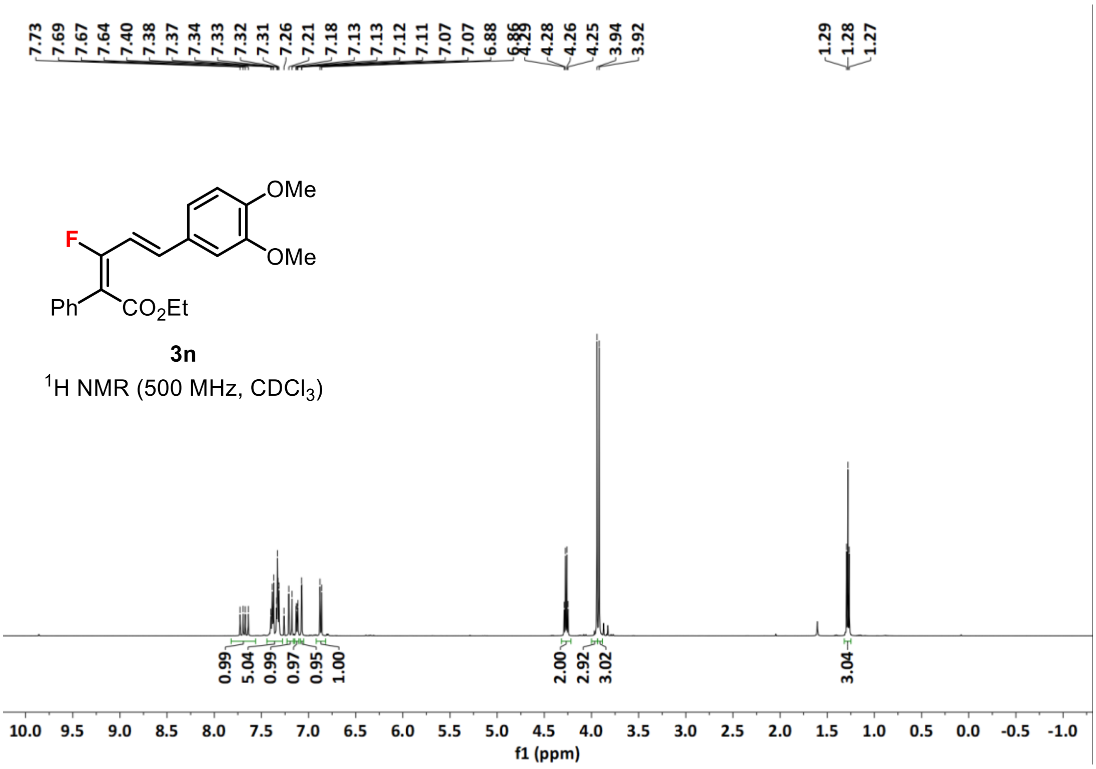

3n

^{1}H NMR (500 MHz, $CDCl_3$)

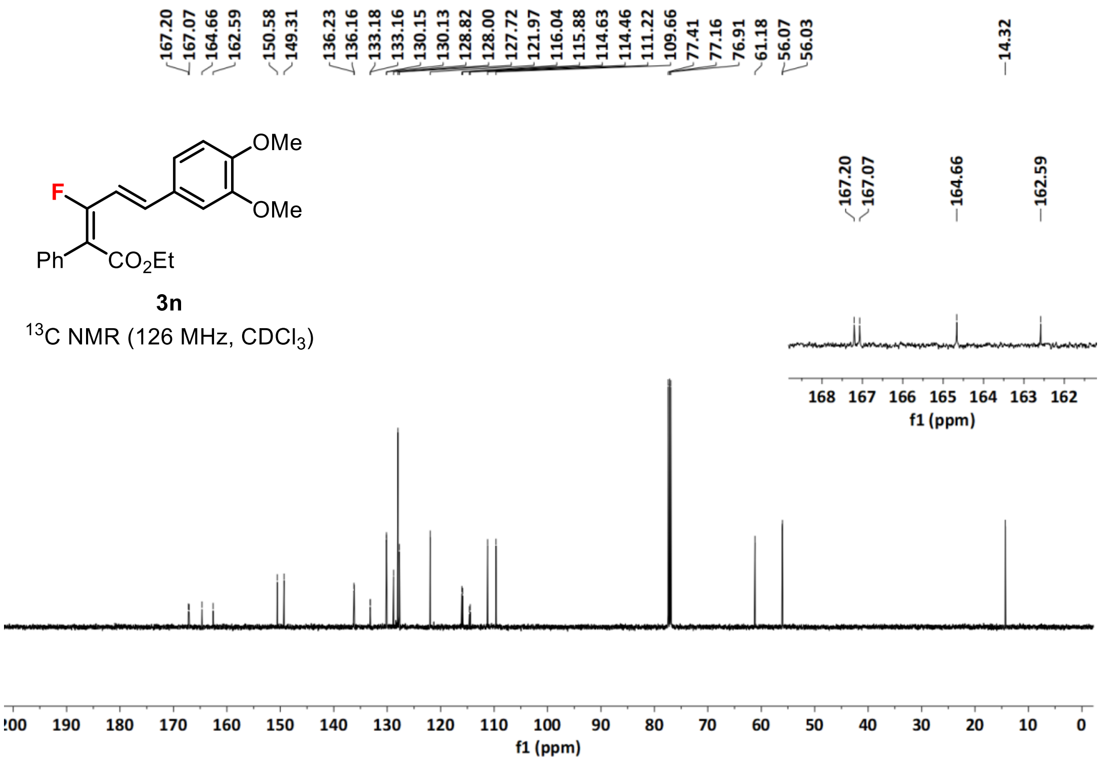
167.20
167.07
164.66
162.59
150.58
149.31
136.23
136.16
133.18
133.16
130.15
130.13
128.82
128.00
127.72
121.97
116.04
115.88
114.63
114.46
111.22
109.66
77.41
77.16
76.91
61.18
56.07
56.03
14.32
OMe
OMe
F
Ph
CO_2Et
3n
^{13}C NMR (126 MHz, $CDCl_3$)
f1 (ppm)

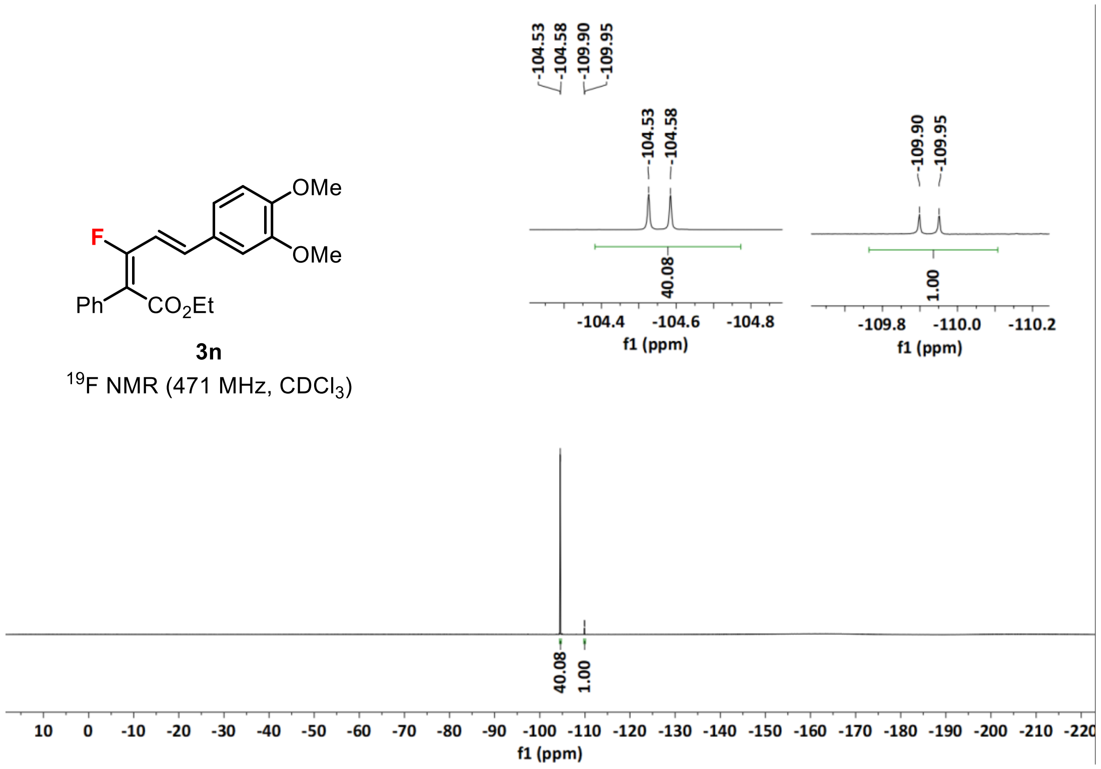
-104.53
-104.58
-109.90
-109.95
OMe
OMe
F
Ph
CO_2Et
3n
^{19}F NMR (471 MHz, $CDCl_3$)
40.08
1.00
f1 (ppm)

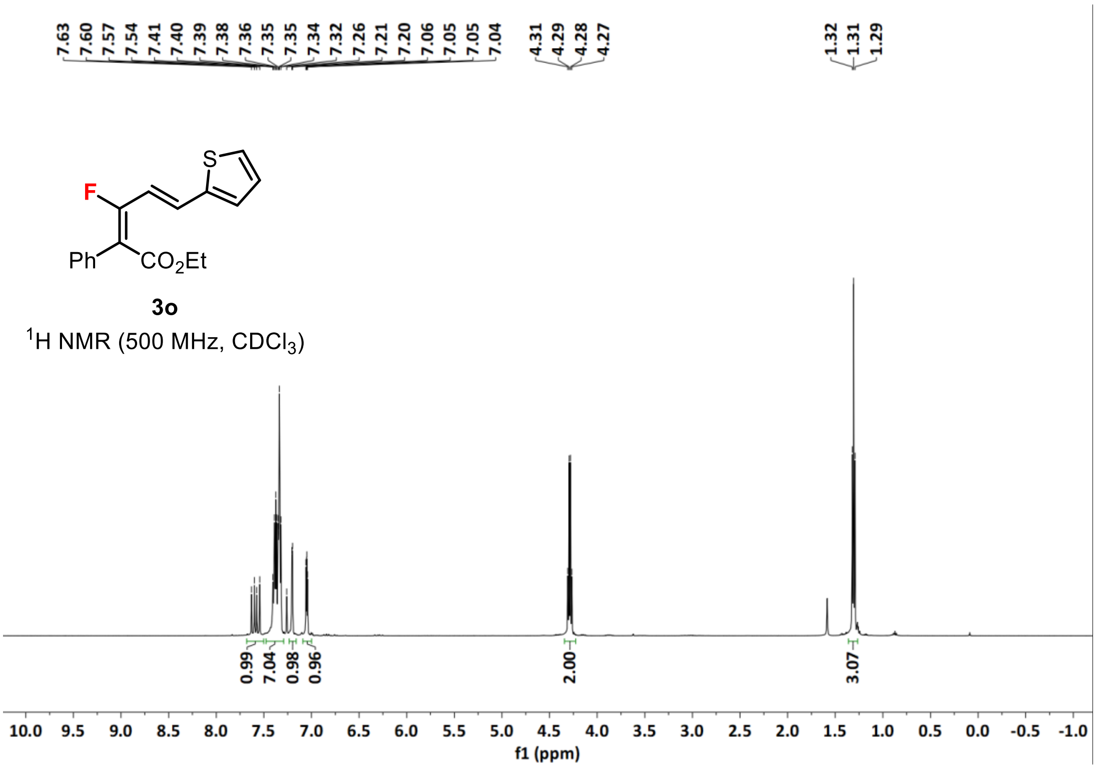
7.63 7.60 7.57 7.54 7.41 7.40 7.39 7.38 7.36 7.35 7.35 7.34 7.32 7.26 7.21 7.20 7.06 7.05 7.05 7.04
4.31 4.29 4.28 4.27
1.32 1.31 1.29
F
S
Ph
CO_2Et
3o
^{1}H NMR (500 MHz, $CDCl_3$)
0.99 7.04 0.98 0.96
2.00
3.07
10.0 9.5 9.0 8.5 8.0 7.5 7.0 6.5 6.0 5.5 5.0 4.5 4.0 3.5 3.0 2.5 2.0 1.5 1.0 0.5 0.0 -0.5 -1.0
f1 (ppm)

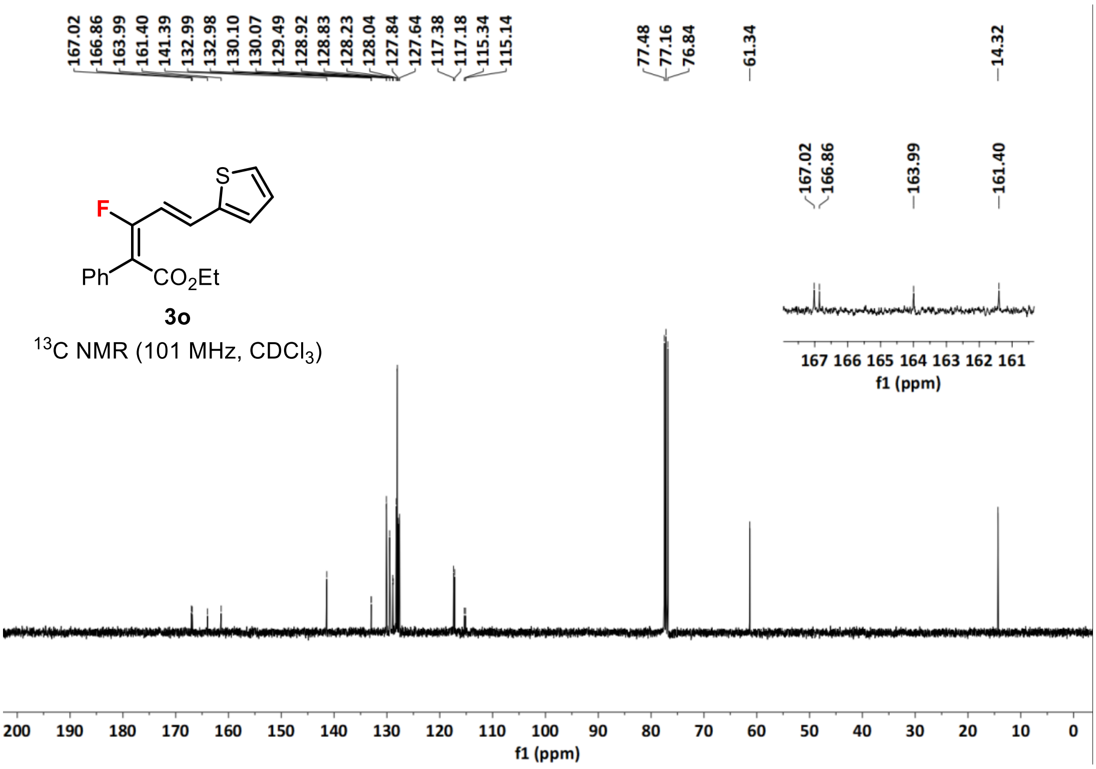
167.02 166.86 163.99 161.40 141.39 132.99 132.98 130.10 130.07 129.49 128.92 128.83 128.23 128.04 127.84 127.64 117.38 117.18 115.34 115.14
77.48 77.16 76.84
61.34
14.32
167.02 166.86
163.99
161.40
167 166 165 164 163 162 161
f1 (ppm)
F
S
Ph
CO_2Et
3o
^{13}C NMR (101 MHz, $CDCl_3$)
200 190 180 170 160 150 140 130 120 110 100 90 80 70 60 50 40 30 20 10 0
f1 (ppm)

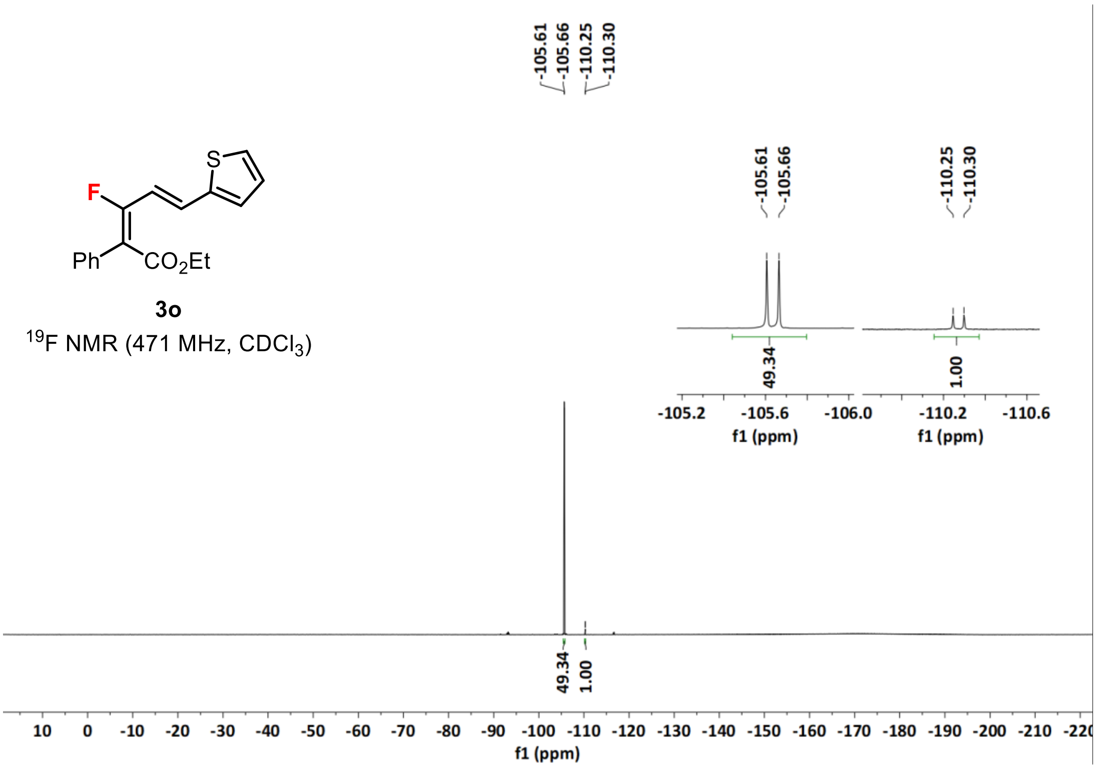
-105.61
-105.66
-110.25
-110.30
F
S
Ph
CO2Et
3o
19F NMR (471 MHz, CDCl3)
49.34
1.00
-105.2
-105.6
-106.0
-110.2
-110.6
f1 (ppm)
10 0 -10 -20 -30 -40 -50 -60 -70 -80 -90 -100 -110 -120 -130 -140 -150 -160 -170 -180 -190 -200 -210 -220
f1 (ppm)

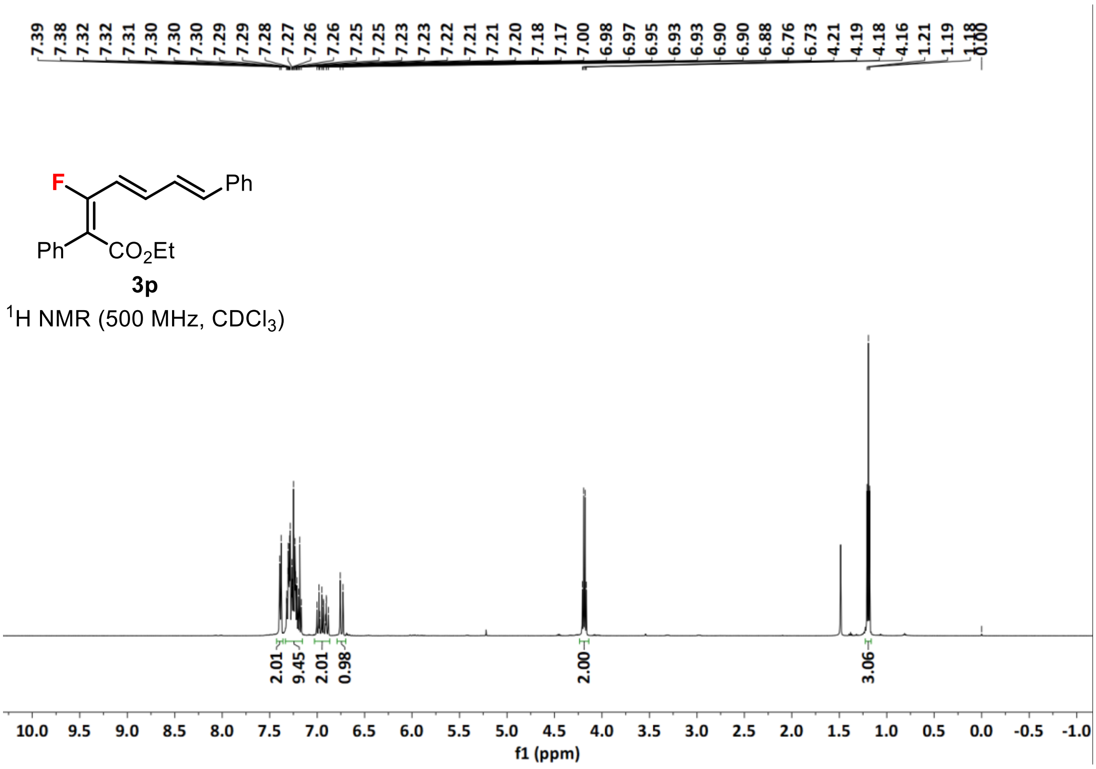
7.39 7.38 7.32 7.32 7.31 7.30 7.30 7.30 7.29 7.29 7.28 7.27 7.26 7.26 7.25 7.25 7.23 7.23 7.22 7.21 7.21 7.20 7.18 7.17 7.00 6.98 6.97 6.95 6.93 6.93 6.90 6.90 6.88 6.76 6.73 4.21 4.19 4.18 4.16 1.21 1.19 1.18 0.00
F
Ph
Ph
CO2Et
3p
1H NMR (500 MHz, CDCl3)
2.01
9.45
2.01
0.98
2.00
3.06
10.0 9.5 9.0 8.5 8.0 7.5 7.0 6.5 6.0 5.5 5.0 4.5 4.0 3.5 3.0 2.5 2.0 1.5 1.0 0.5 0.0 -0.5 -1.0
f1 (ppm)

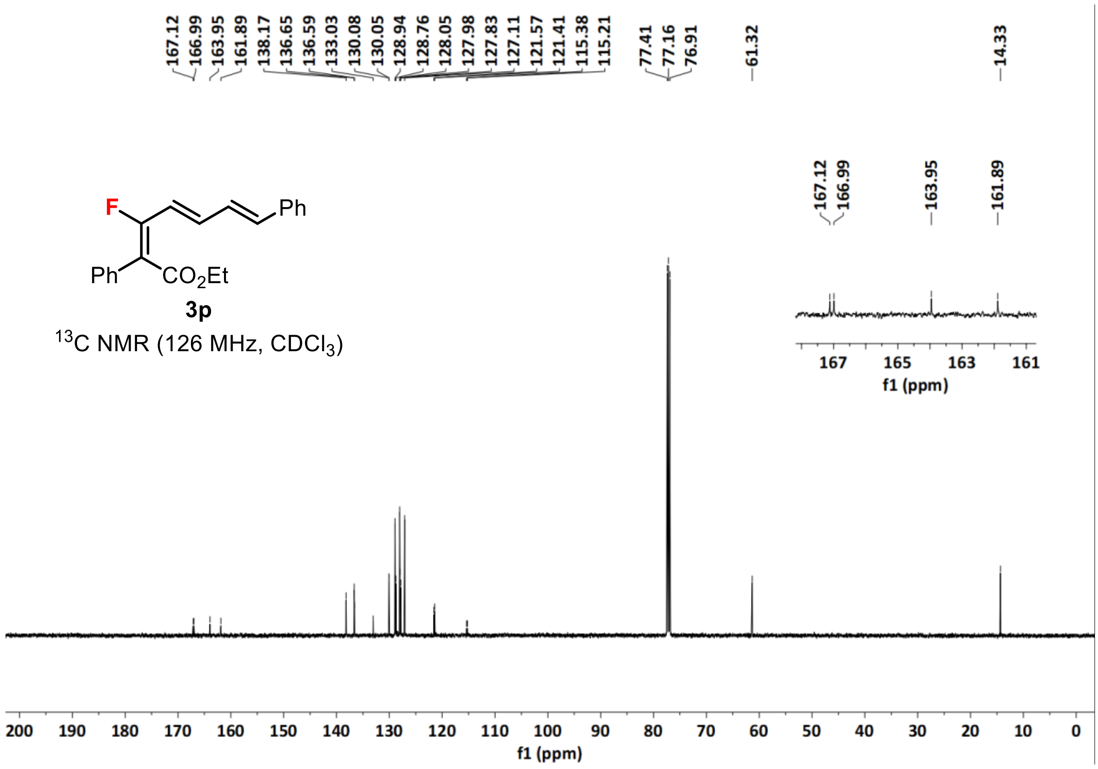
167.12
166.99
163.95
161.89
138.17
136.65
136.59
133.03
130.08
130.05
128.94
128.76
128.05
127.98
127.83
127.11
121.57
121.41
115.38
115.21
77.41
77.16
76.91
61.32
14.33
F
Ph
Ph
CO2Et
3p
13C NMR (126 MHz, CDCl3)
f1 (ppm)

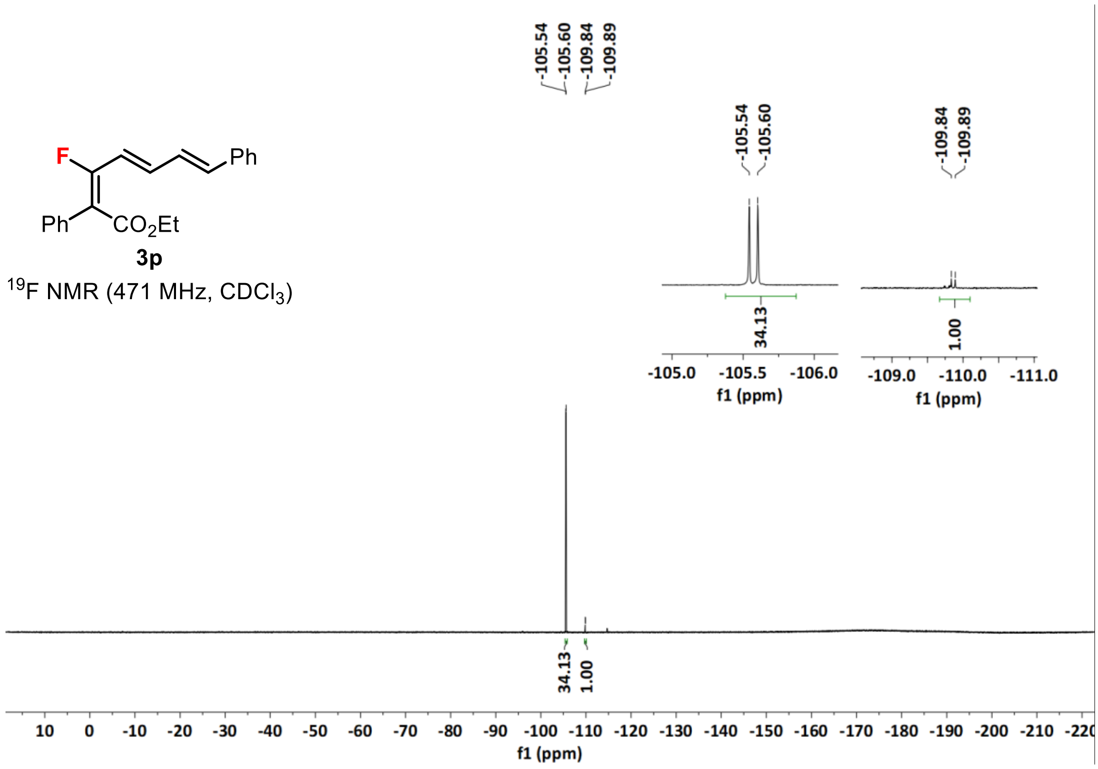
-105.54
-105.60
-109.84
-109.89
F
Ph
Ph
CO2Et
3p
19F NMR (471 MHz, CDCl3)
34.13
1.00
f1 (ppm)

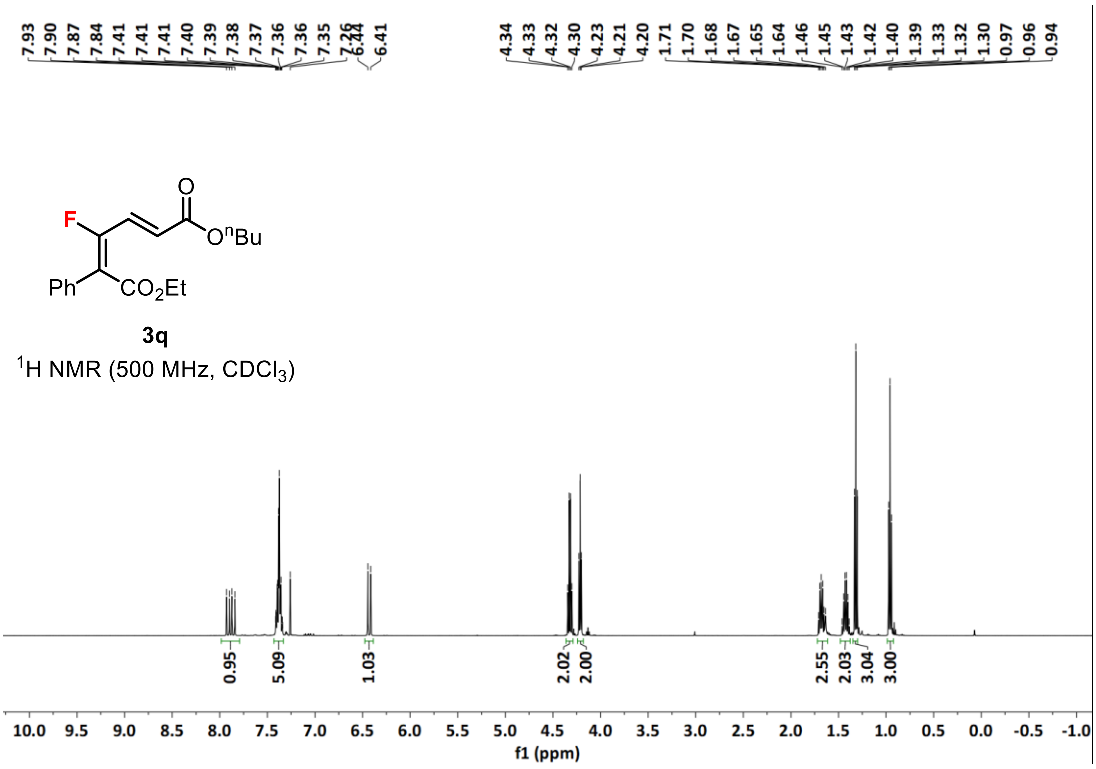

3q

^{1}H NMR (500 MHz, $CDCl_3$)

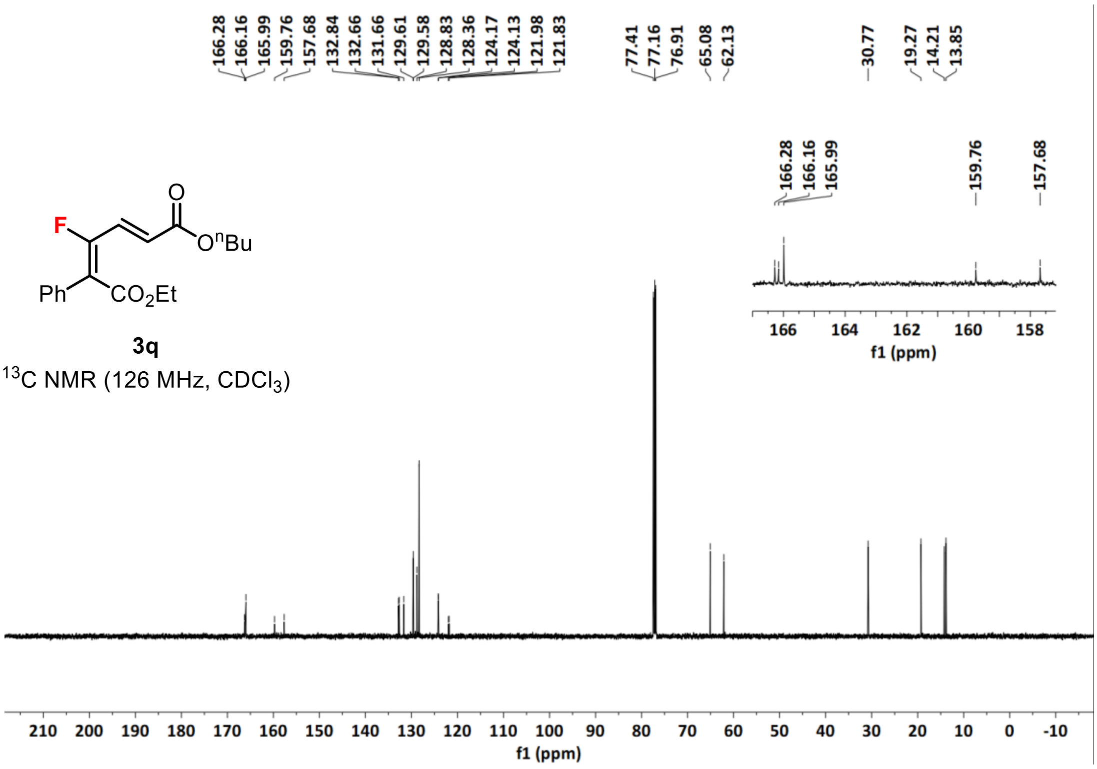

3q

^{13}C NMR (126 MHz, $CDCl_3$)

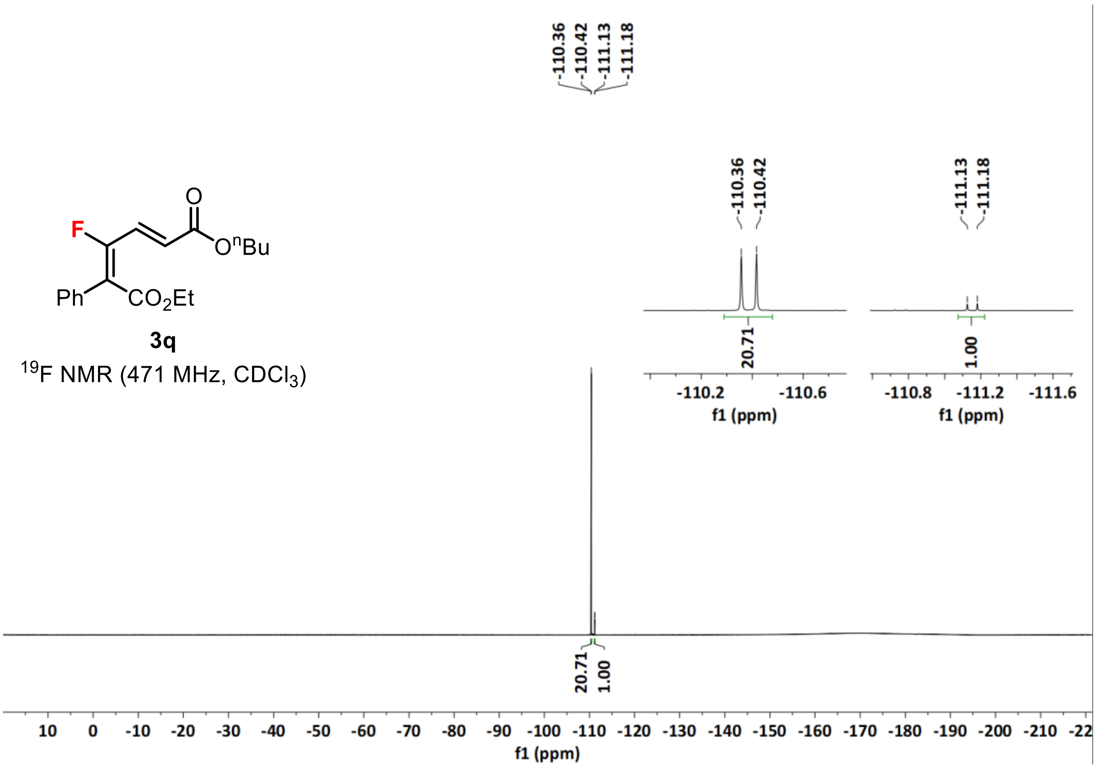

3q

^{19}F NMR (471 MHz, $CDCl_3$)

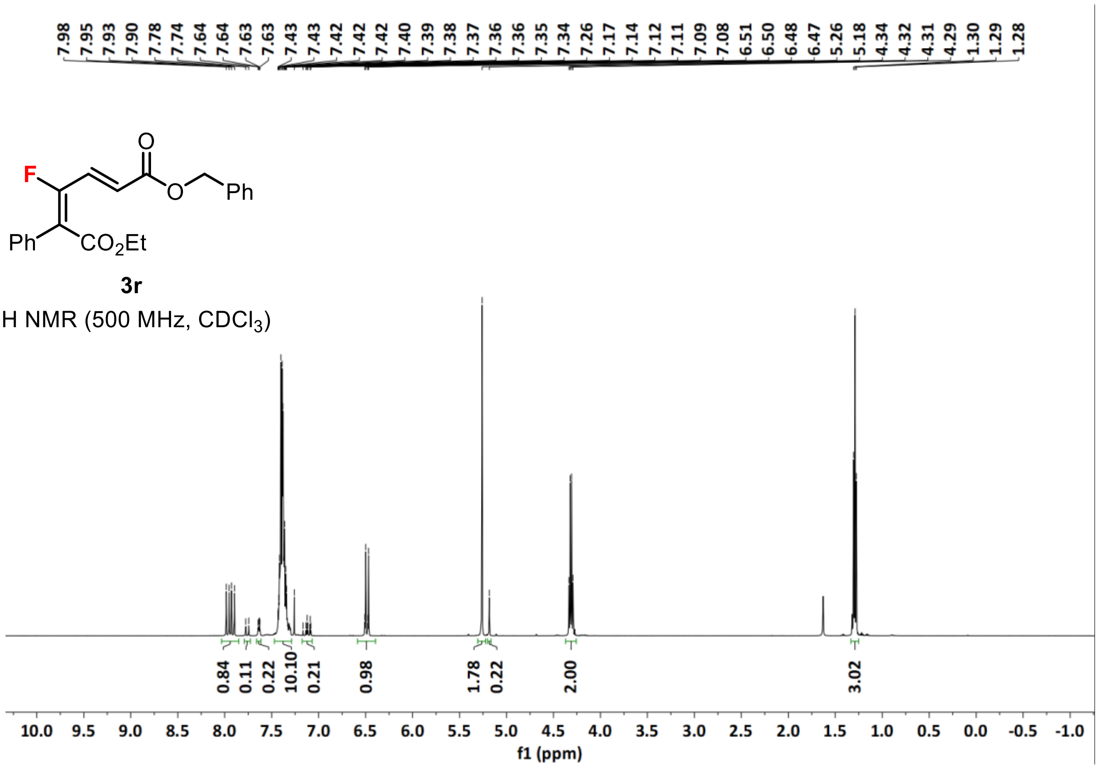

3r

^{1}H NMR (500 MHz, $CDCl_3$)

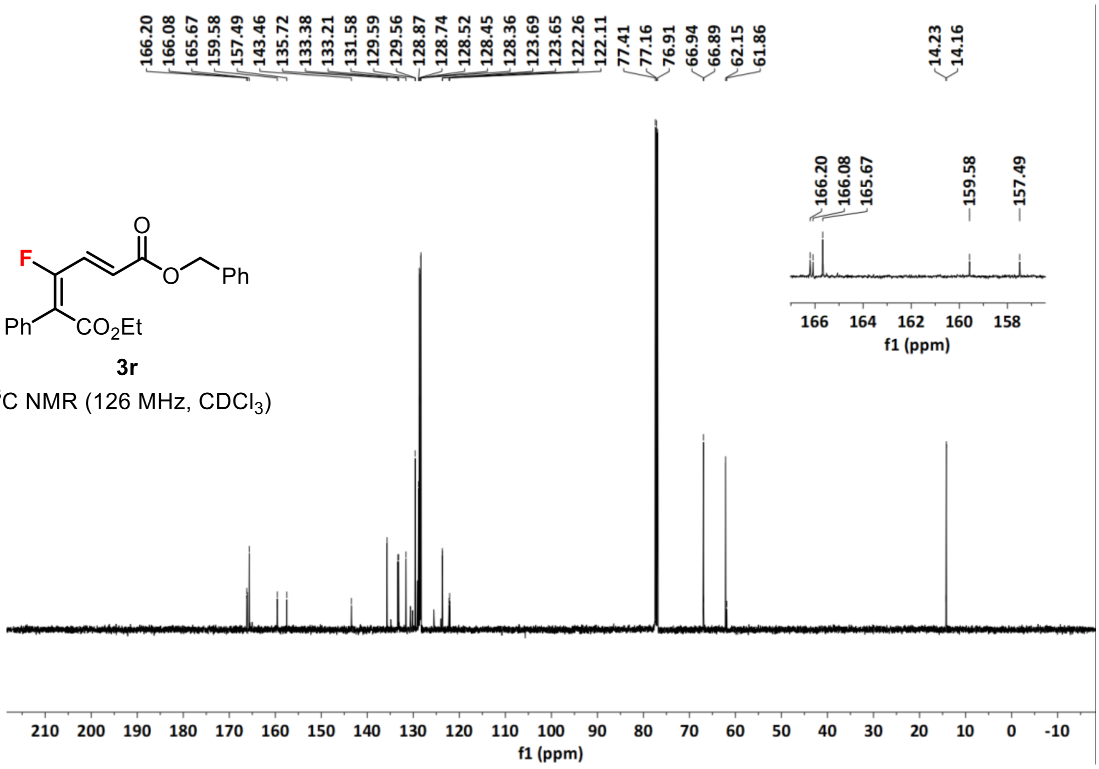

3r

^{13}C NMR (126 MHz, $CDCl_3$)

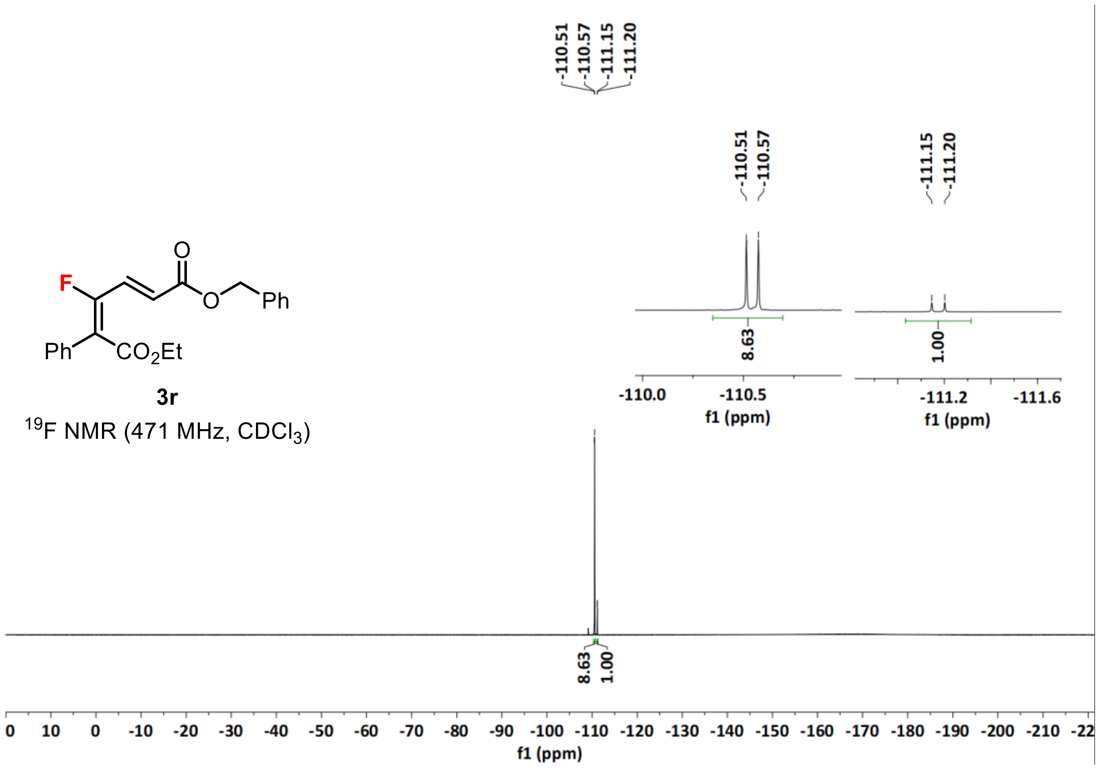

3r

^{19}F NMR (471 MHz, $CDCl_3$)

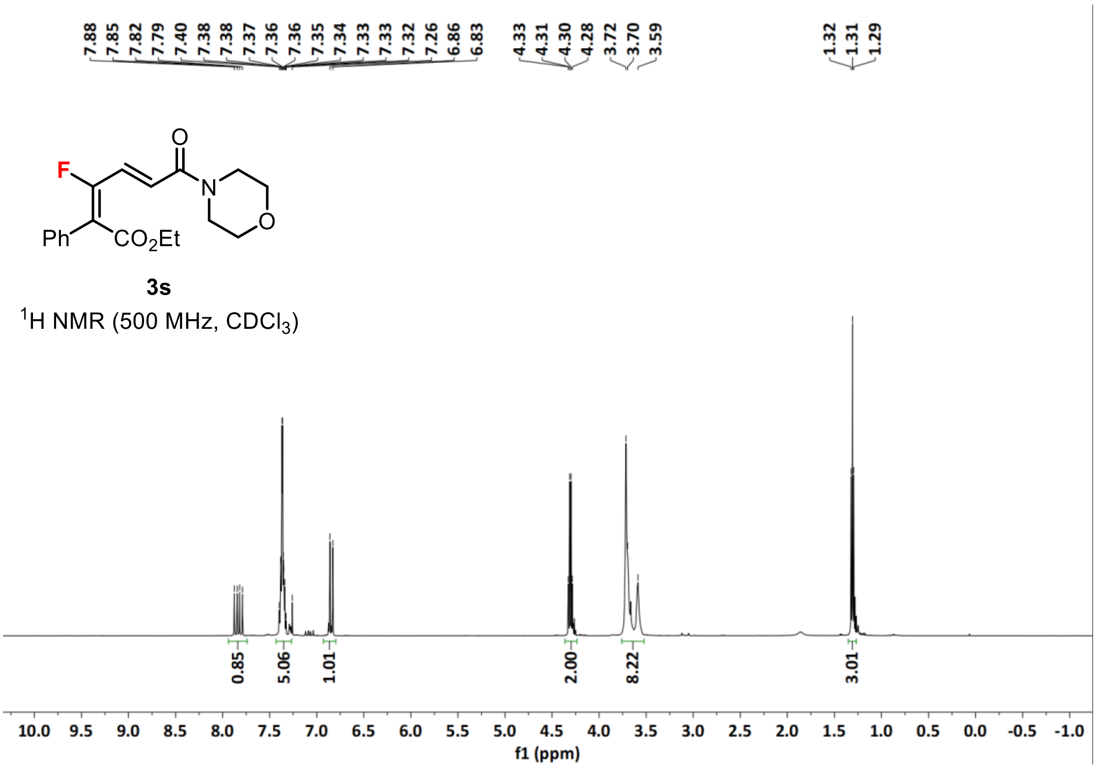

7.88
7.85
7.82
7.79
7.40
7.38
7.38
7.37
7.36
7.36
7.35
7.34
7.33
7.33
7.32
7.26
6.86
6.83
4.33
4.31
4.30
4.28
3.72
3.70
3.59
1.32
1.31
1.29
F
O
N
O
Ph
CO_2Et
3s
^1H NMR (500 MHz, $CDCl_3$)
0.85
5.06
1.01
2.00
8.22
3.01
f1 (ppm)

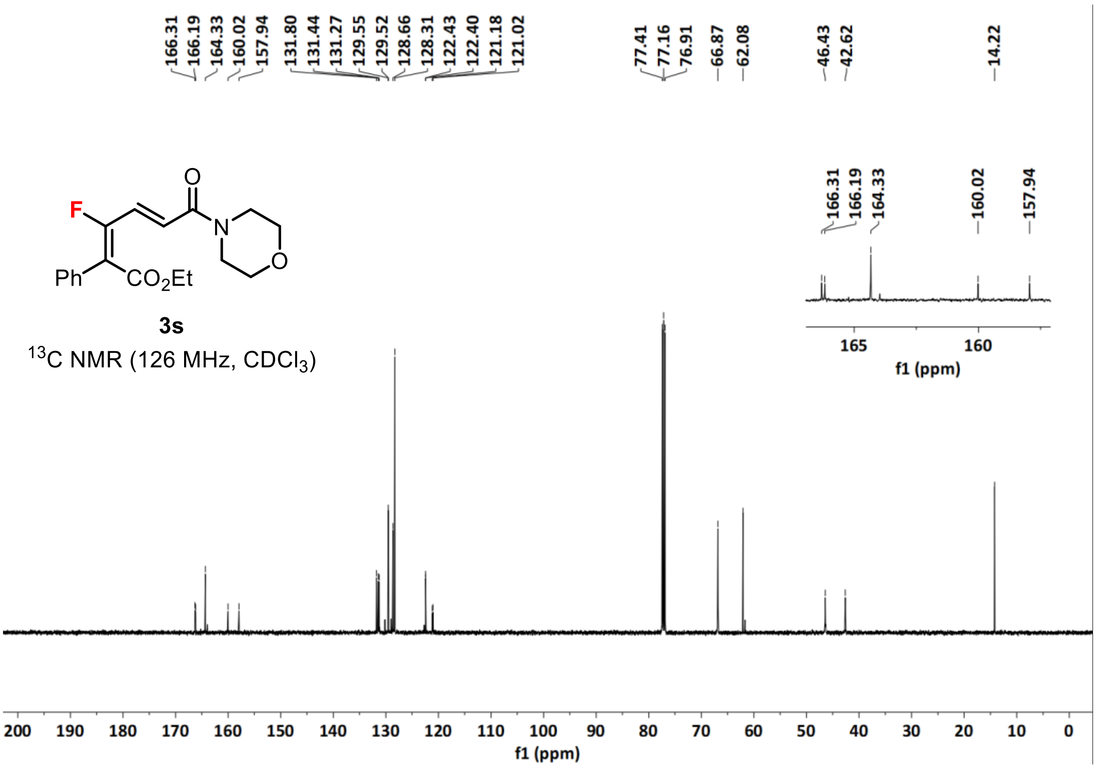

166.31
166.19
164.33
160.02
157.94
131.80
131.44
131.27
129.55
129.52
128.66
128.31
122.43
122.40
121.18
121.02
77.41
77.16
76.91
66.87
62.08
46.43
42.62
14.22
F
O
N
O
Ph
CO_2Et
3s
^{13}C NMR (126 MHz, $CDCl_3$)
f1 (ppm)

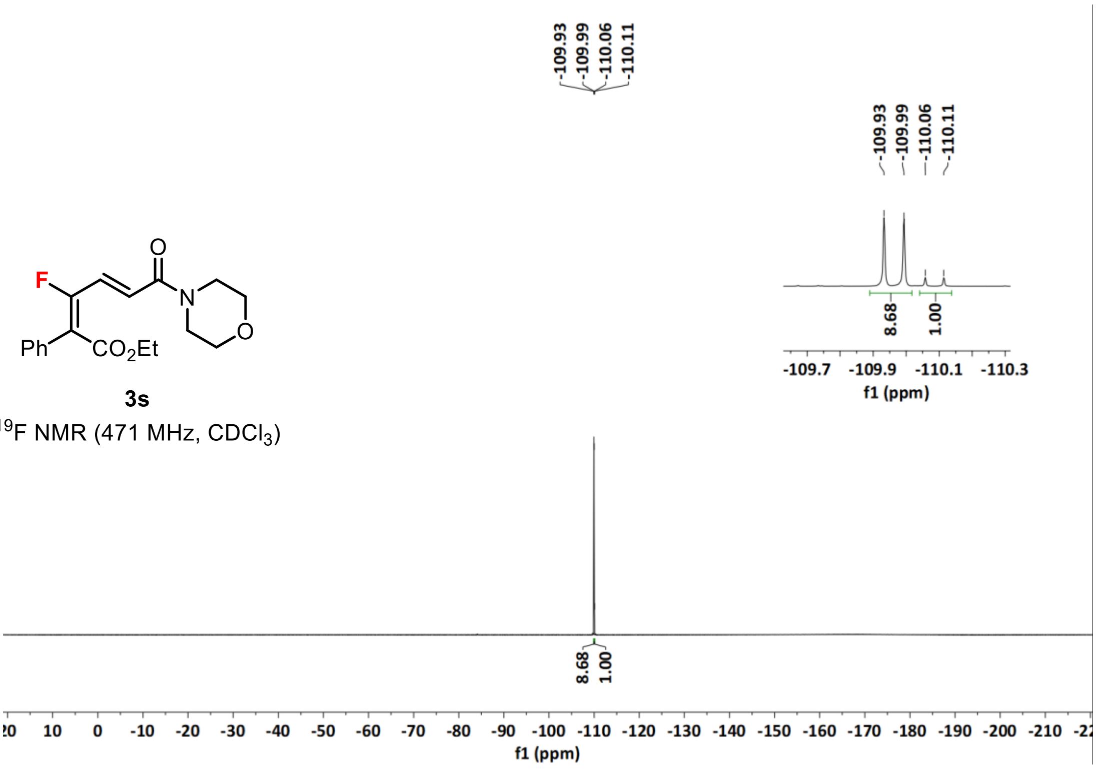

3s

^{19}F NMR (471 MHz, $CDCl_3$)

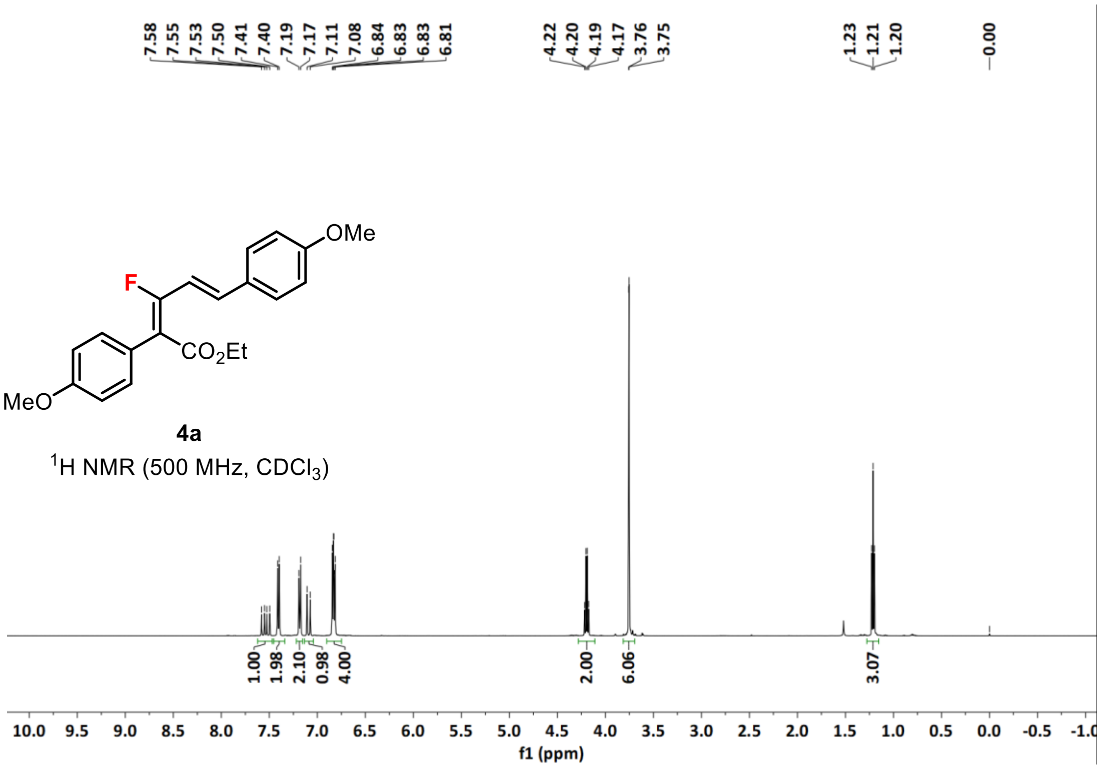

4a

^{1}H NMR (500 MHz, $CDCl_3$)

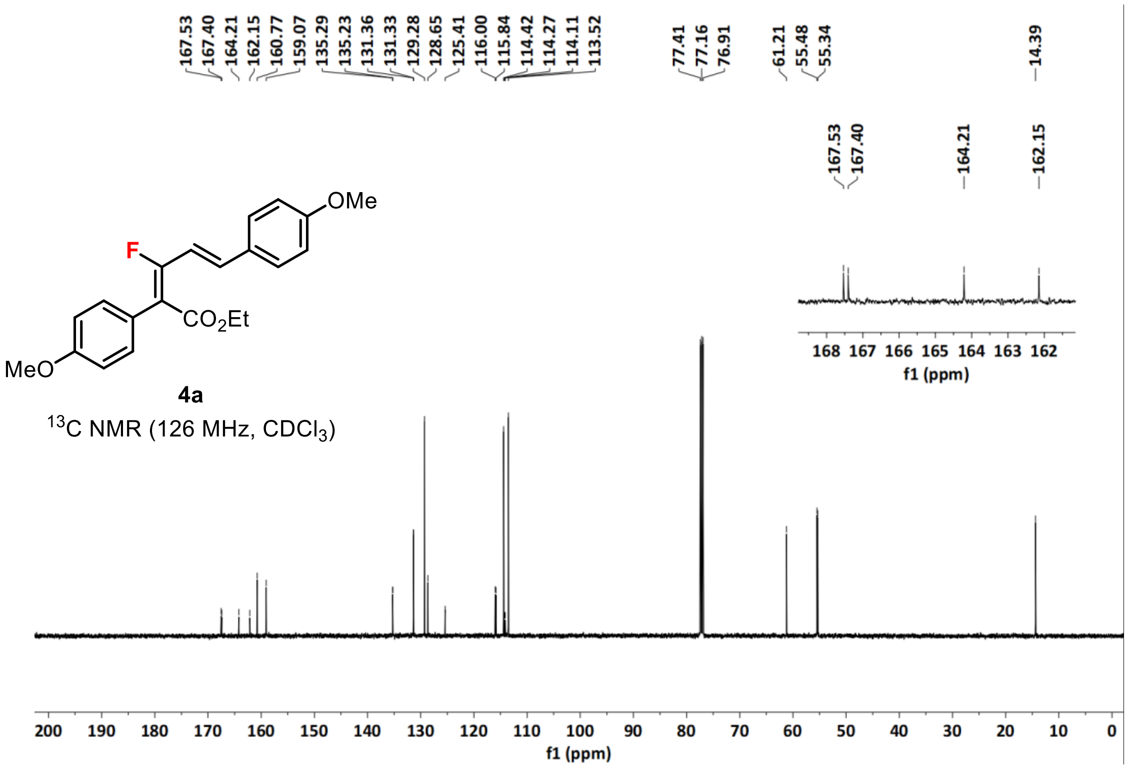

167.53
167.40
164.21
162.15
160.77
159.07
135.29
135.23
131.36
131.33
129.28
128.65
125.41
116.00
115.84
114.42
114.27
114.11
113.52
77.41
77.16
76.91
61.21
55.48
55.34
14.39
F
OMe
CO_2Et
MeO
4a
^{13}C NMR (126 MHz, $CDCl_3$)
f1 (ppm)

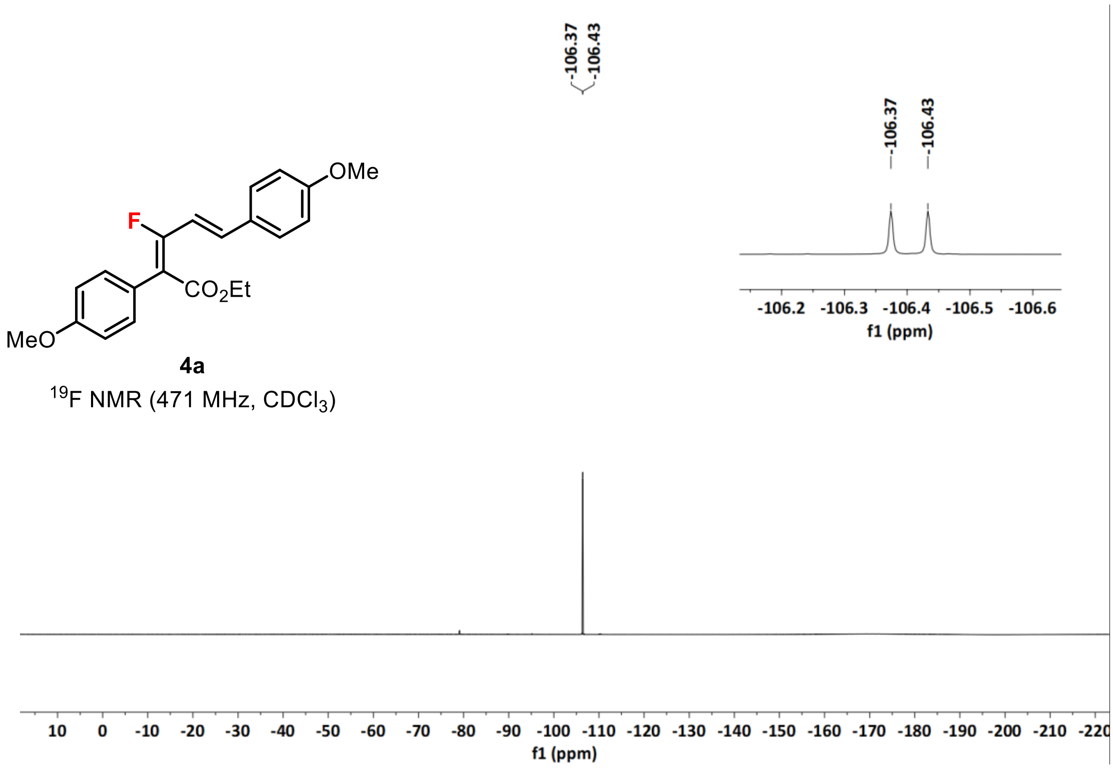

-106.37
-106.43
F
OMe
CO_2Et
MeO
4a
^{19}F NMR (471 MHz, $CDCl_3$)
f1 (ppm)

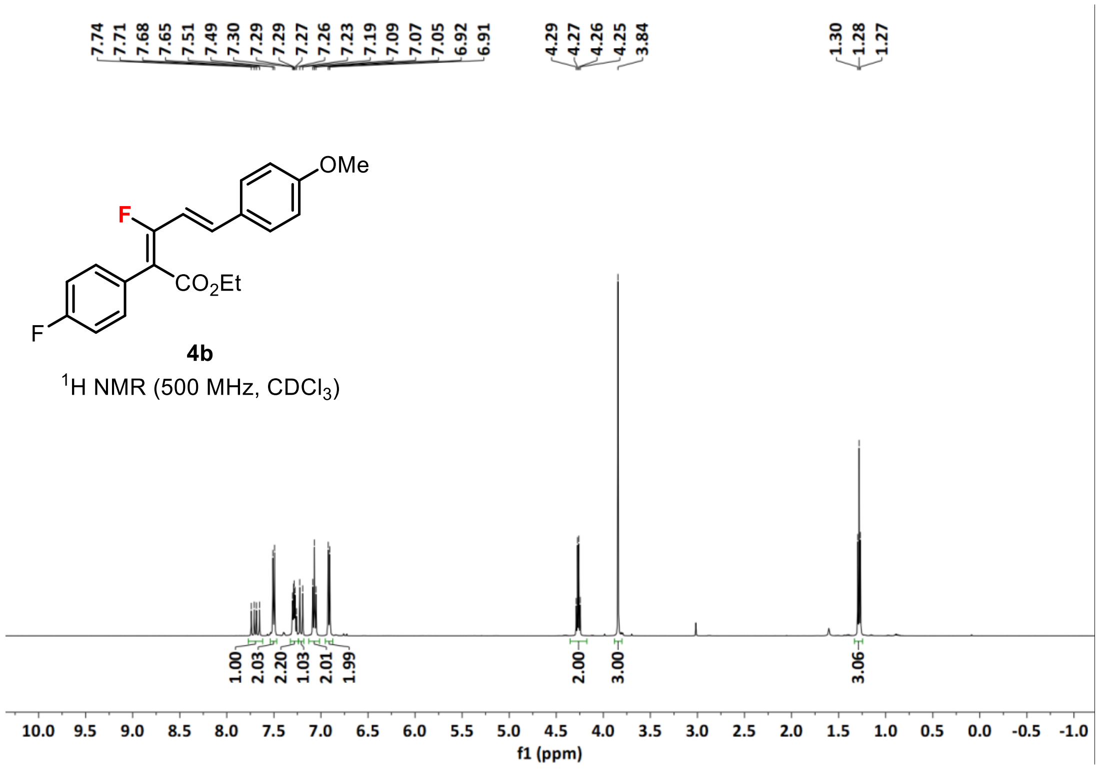
7.74 7.71 7.68 7.65 7.51 7.49 7.30 7.29 7.29 7.27 7.26 7.23 7.19 7.09 7.07 7.05 6.92 6.91
4.29 4.27 4.26 4.25 3.84
1.30 1.28 1.27
OMe
F
CO_2Et
F
4b
^1H NMR (500 MHz, $CDCl_3$)
1.00 2.03 2.20 1.03 2.01 1.99
2.00 3.00
3.06
10.0 9.5 9.0 8.5 8.0 7.5 7.0 6.5 6.0 5.5 5.0 4.5 4.0 3.5 3.0 2.5 2.0 1.5 1.0 0.5 0.0 -0.5 -1.0
f1 (ppm)

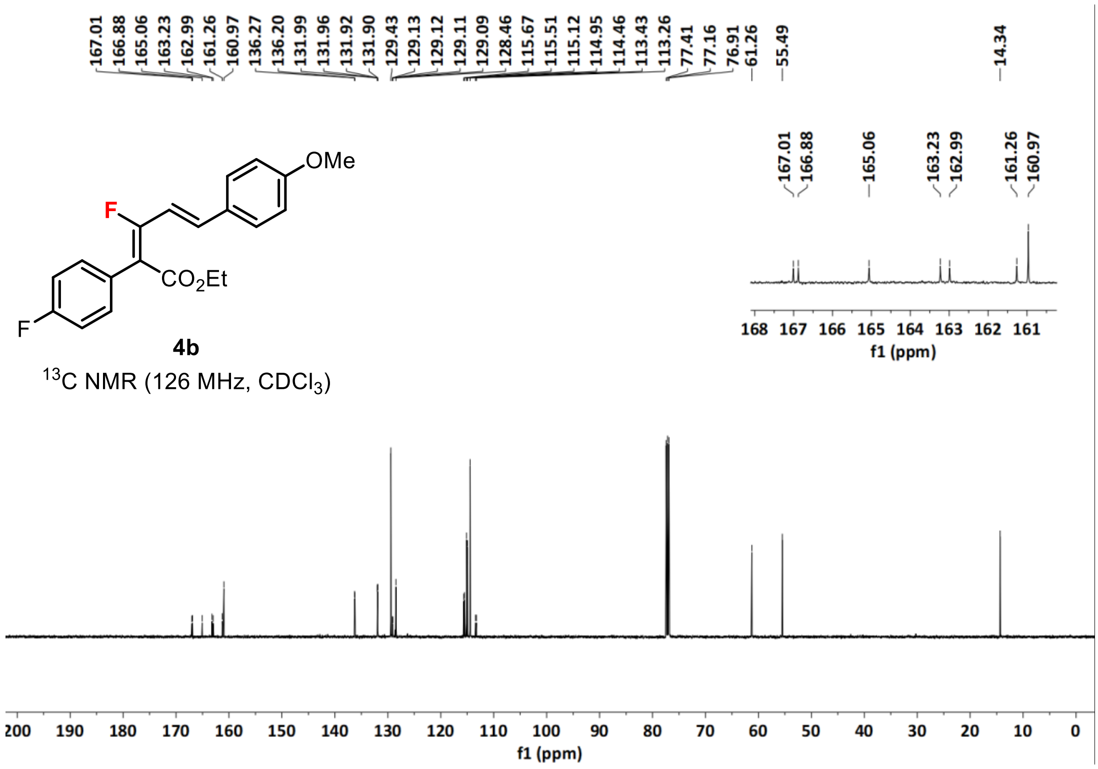
167.01 166.88 165.06 163.23 162.99 161.26 160.97 136.27 136.20 131.99 131.96 131.92 131.90 129.43 129.13 129.12 129.11 129.09 128.46 115.67 115.51 115.12 114.95 114.46 113.43 113.26 77.41 77.16 76.91 61.26
55.49
14.34
OMe
F
CO_2Et
F
4b
^{13}C NMR (126 MHz, $CDCl_3$)
167.01 166.88 165.06 163.23 162.99 161.26 160.97
168 167 166 165 164 163 162 161
f1 (ppm)
200 190 180 170 160 150 140 130 120 110 100 90 80 70 60 50 40 30 20 10 0
f1 (ppm)

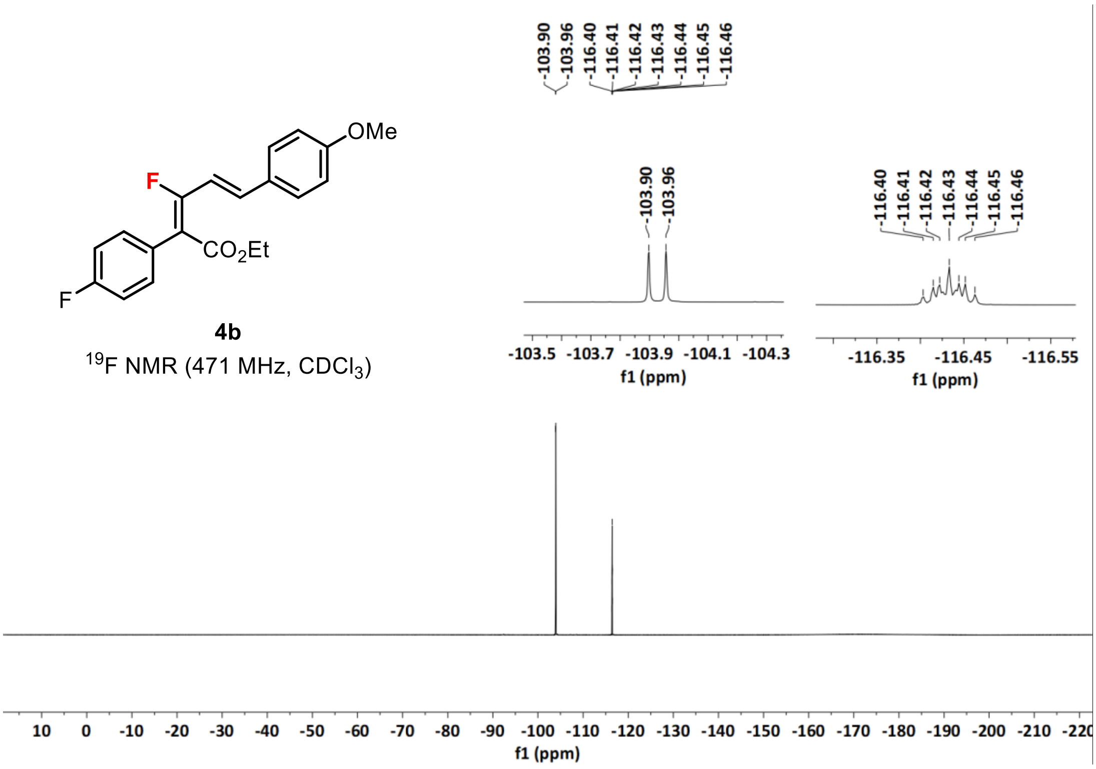

4b

^{19}F NMR (471 MHz, $CDCl_3$)

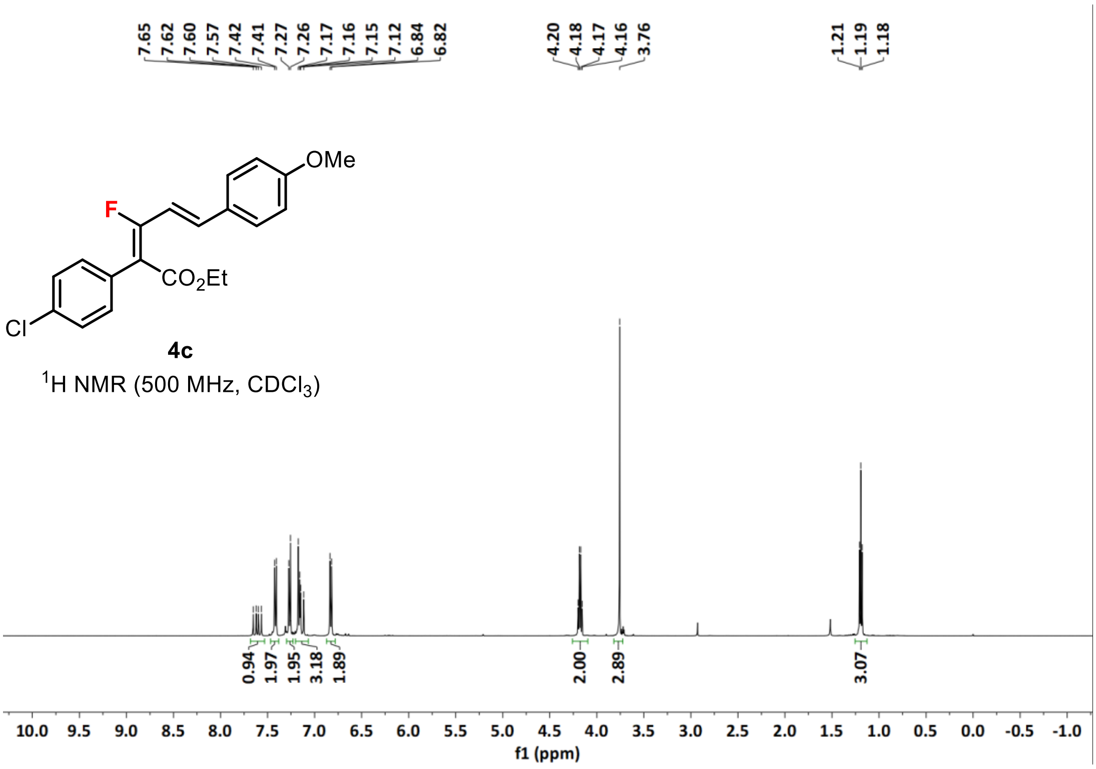

4c

^{1}H NMR (500 MHz, $CDCl_3$)

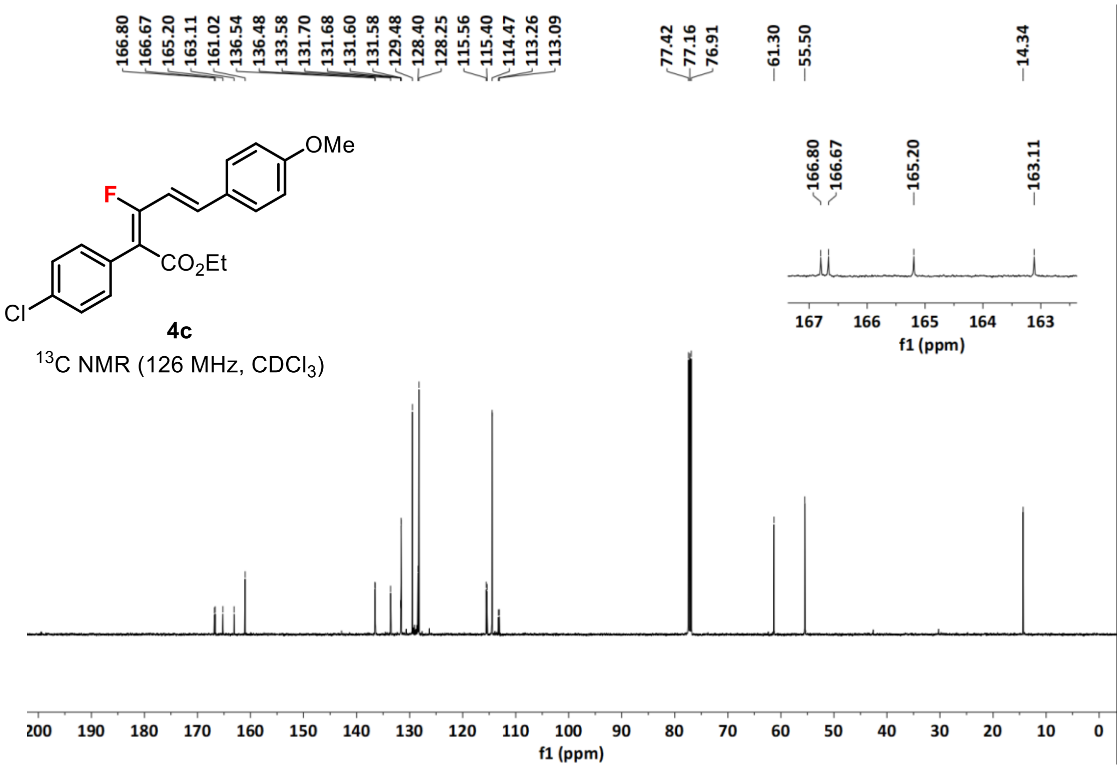
166.80
166.67
165.20
163.11
161.02
136.54
136.48
133.58
131.70
131.68
131.60
131.58
129.48
128.40
128.25
115.56
115.40
114.47
113.26
113.09
77.42
77.16
76.91
61.30
55.50
14.34
OMe
F
CO_2Et
Cl
4c
^{13}C NMR (126 MHz, $CDCl_3$)
167 166 165 164 163
f1 (ppm)
200 190 180 170 160 150 140 130 120 110 100 90 80 70 60 50 40 30 20 10 0
f1 (ppm)

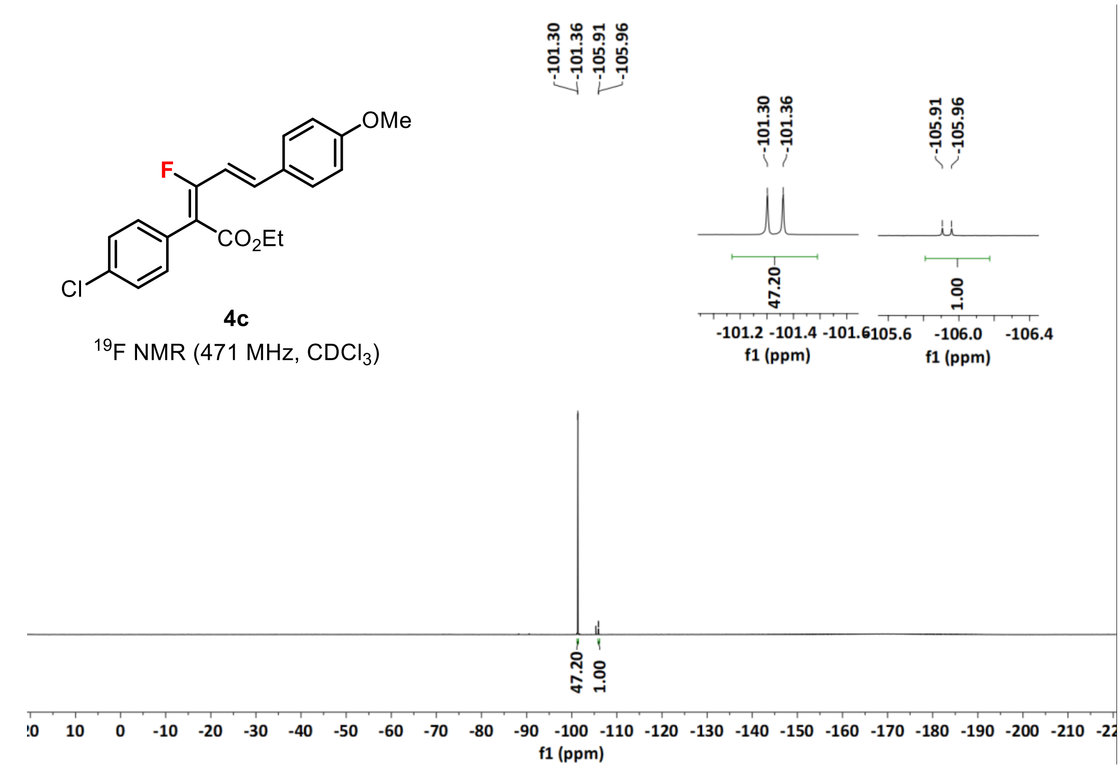
-101.30
-101.36
-105.91
-105.96
OMe
F
CO_2Et
Cl
4c
^{19}F NMR (471 MHz, $CDCl_3$)
47.20
1.00
-101.2 -101.4 -101.6
f1 (ppm)
-105.6 -106.0 -106.4
f1 (ppm)
10 0 -10 -20 -30 -40 -50 -60 -70 -80 -90 -100 -110 -120 -130 -140 -150 -160 -170 -180 -190 -200 -210
f1 (ppm)

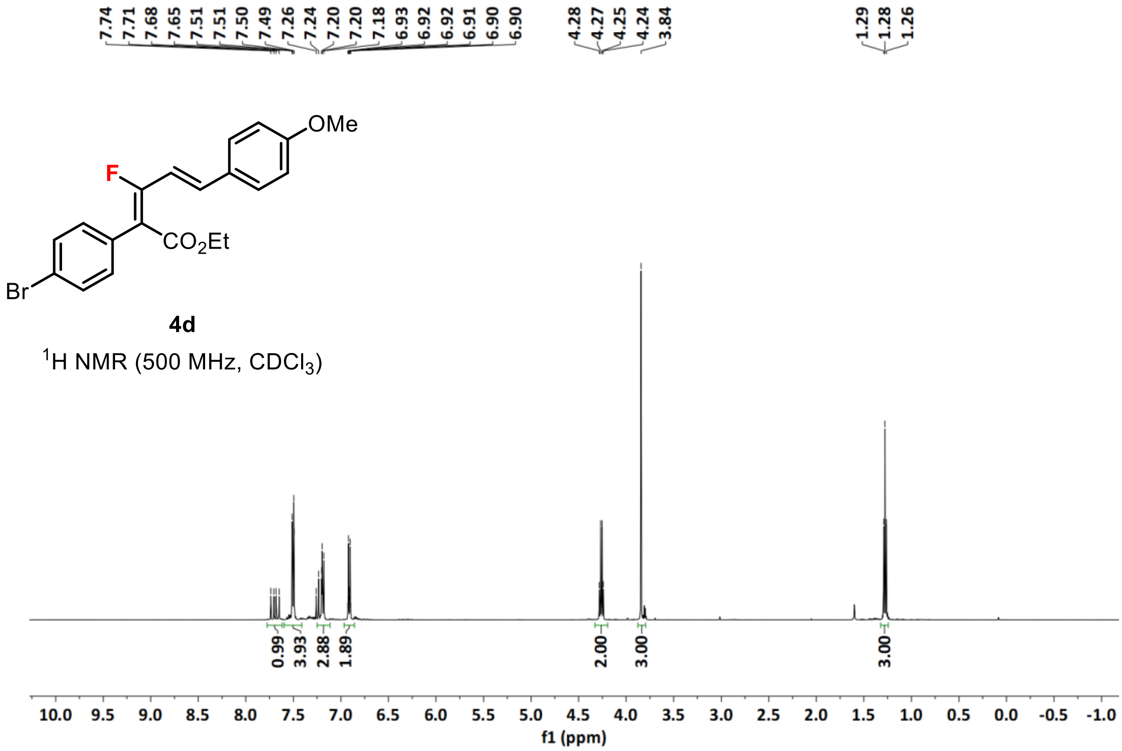
7.74 7.71 7.68 7.65 7.51 7.51 7.50 7.49 7.26 7.24 7.20 7.20 7.18 6.93 6.92 6.92 6.91 6.90 6.90
4.28 4.27 4.25 4.24 3.84
1.29 1.28 1.26
OMe
F
CO2Et
Br
4d
1H NMR (500 MHz, CDCl3)
0.99 3.93 2.88 1.89
2.00 3.00 3.00
10.0 9.5 9.0 8.5 8.0 7.5 7.0 6.5 6.0 5.5 5.0 4.5 4.0 3.5 3.0 2.5 2.0 1.5 1.0 0.5 0.0 -0.5 -1.0
f1 (ppm)

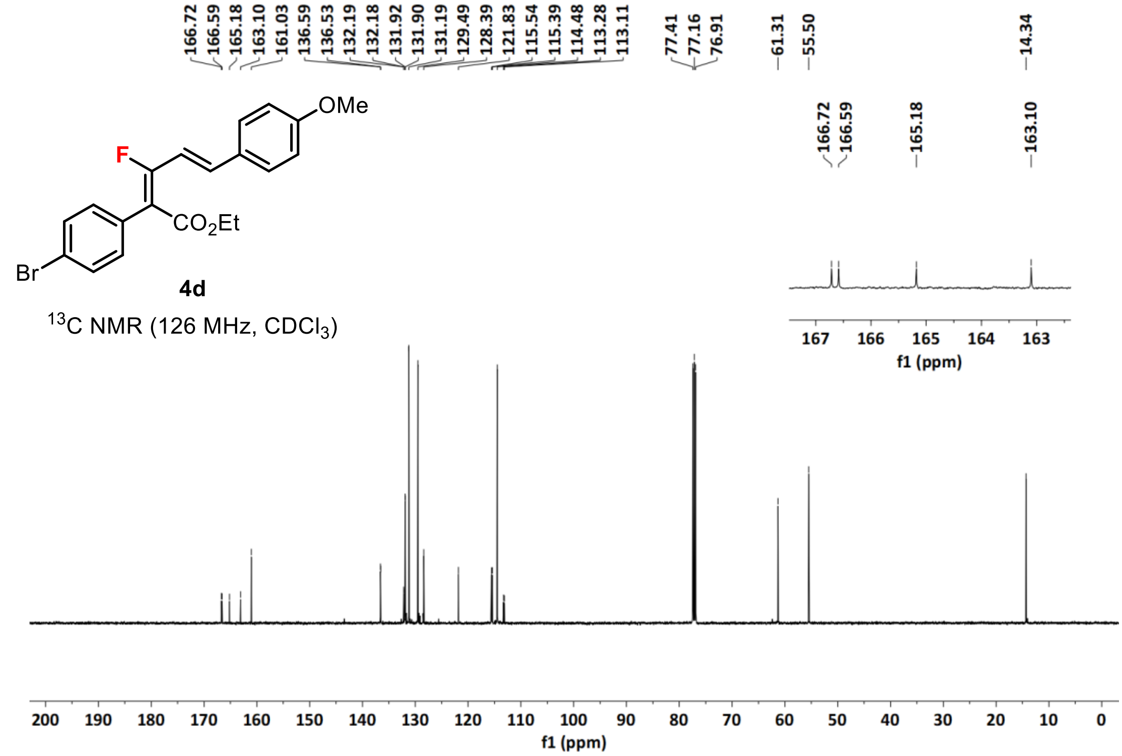
166.72 166.59 165.18 163.10 161.03 136.59 136.53 132.19 132.18 131.92 131.90 131.19 129.49 128.39 121.83 115.54 115.39 114.48 113.28 113.11
77.41 77.16 76.91
61.31
55.50
14.34
OMe
F
CO2Et
Br
4d
13C NMR (126 MHz, CDCl3)
166.72 166.59 165.18 163.10
167 166 165 164 163
f1 (ppm)
200 190 180 170 160 150 140 130 120 110 100 90 80 70 60 50 40 30 20 10 0
f1 (ppm)

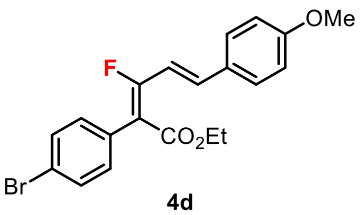

4d

^{19}F NMR (471 MHz, $CDCl_3$)

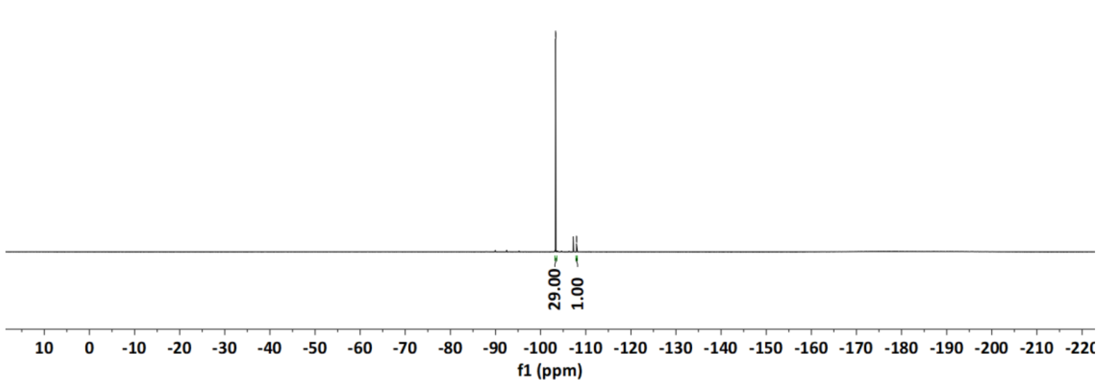

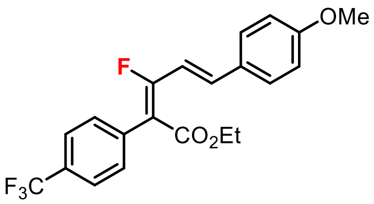

4e

^{1}H NMR (500 MHz, $CDCl_3$)

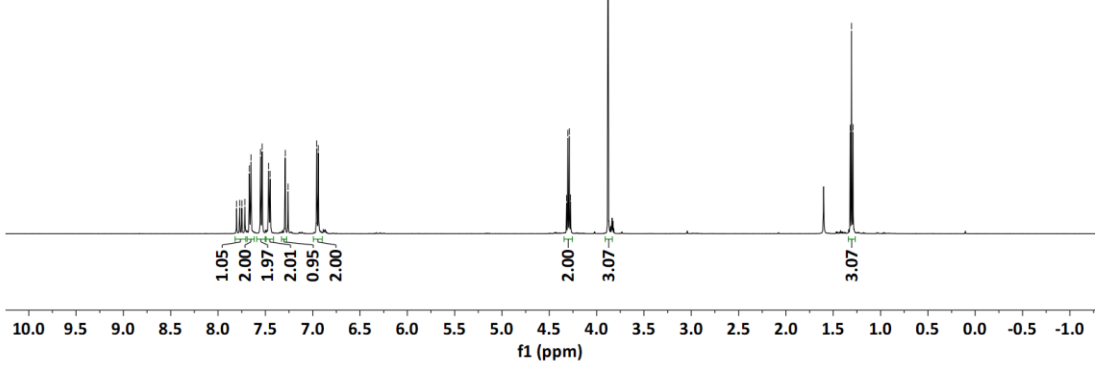

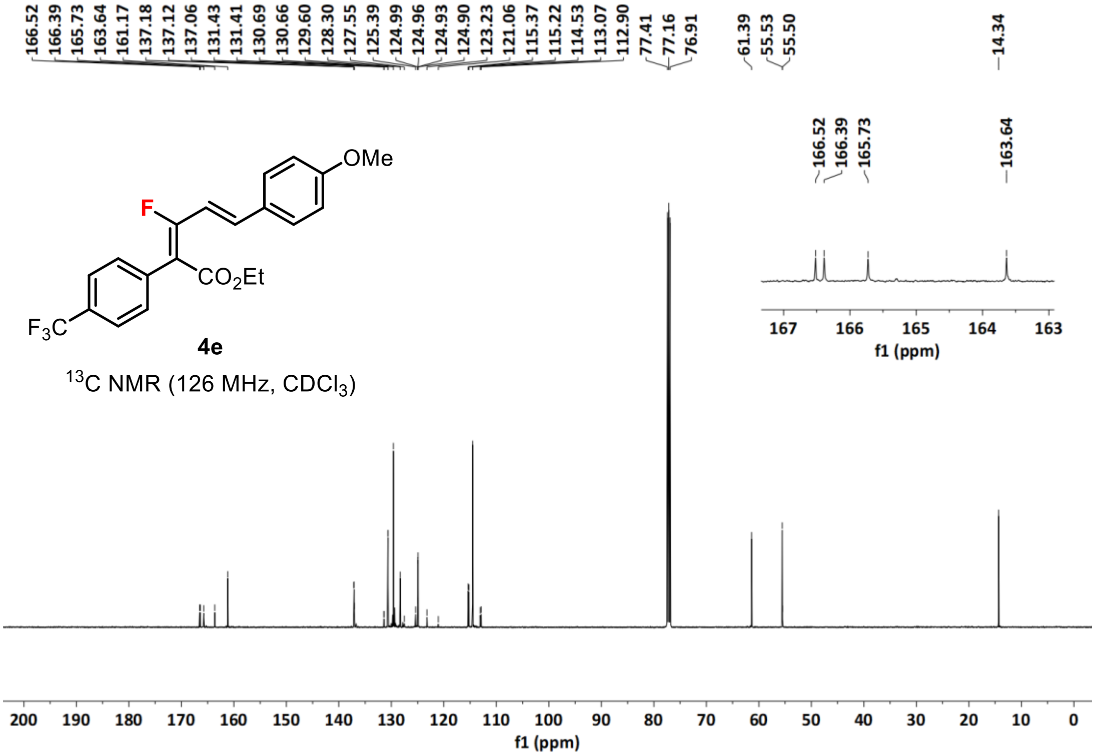
166.52 166.39 165.73 163.64 161.17 137.18 137.12 137.06 131.43 131.41 130.69 130.66 129.60 128.30 127.55 125.39 124.99 124.96 124.93 124.90 123.23 121.06 115.37 115.22 114.53 113.07 112.90 77.41 77.16 76.91 61.39 55.53 55.50 14.34
OMe
F
CO_2Et
F_3C
4e
^{13}C NMR (126 MHz, $CDCl_3$)
166.52 166.39 165.73 163.64
167 166 165 164 163
f1 (ppm)
200 190 180 170 160 150 140 130 120 110 100 90 80 70 60 50 40 30 20 10 0
f1 (ppm)

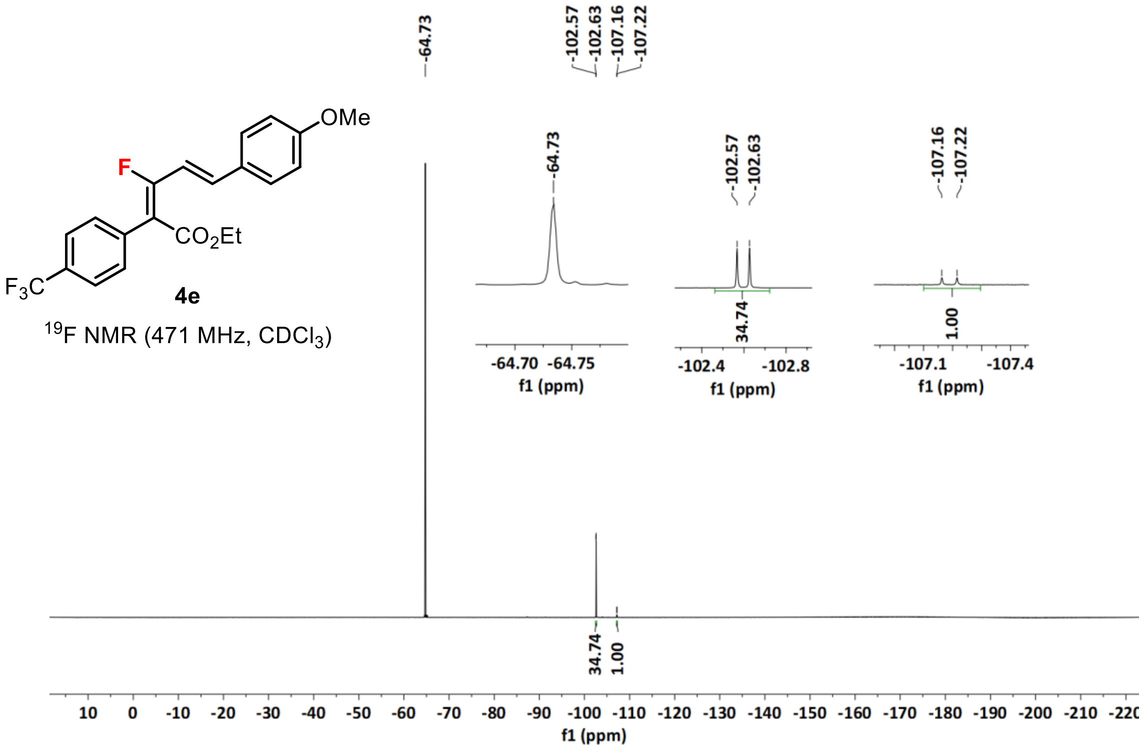
-64.73
-102.57 -102.63 -107.16 -107.22
OMe
F
CO_2Et
F_3C
4e
^{19}F NMR (471 MHz, $CDCl_3$)
-64.73
-64.70 -64.75
f1 (ppm)
-102.57 -102.63
34.74
-102.4 -102.8
f1 (ppm)
-107.16 -107.22
1.00
-107.1 -107.4
f1 (ppm)
34.74 1.00
10 0 -10 -20 -30 -40 -50 -60 -70 -80 -90 -100 -110 -120 -130 -140 -150 -160 -170 -180 -190 -200 -210 -220
f1 (ppm)

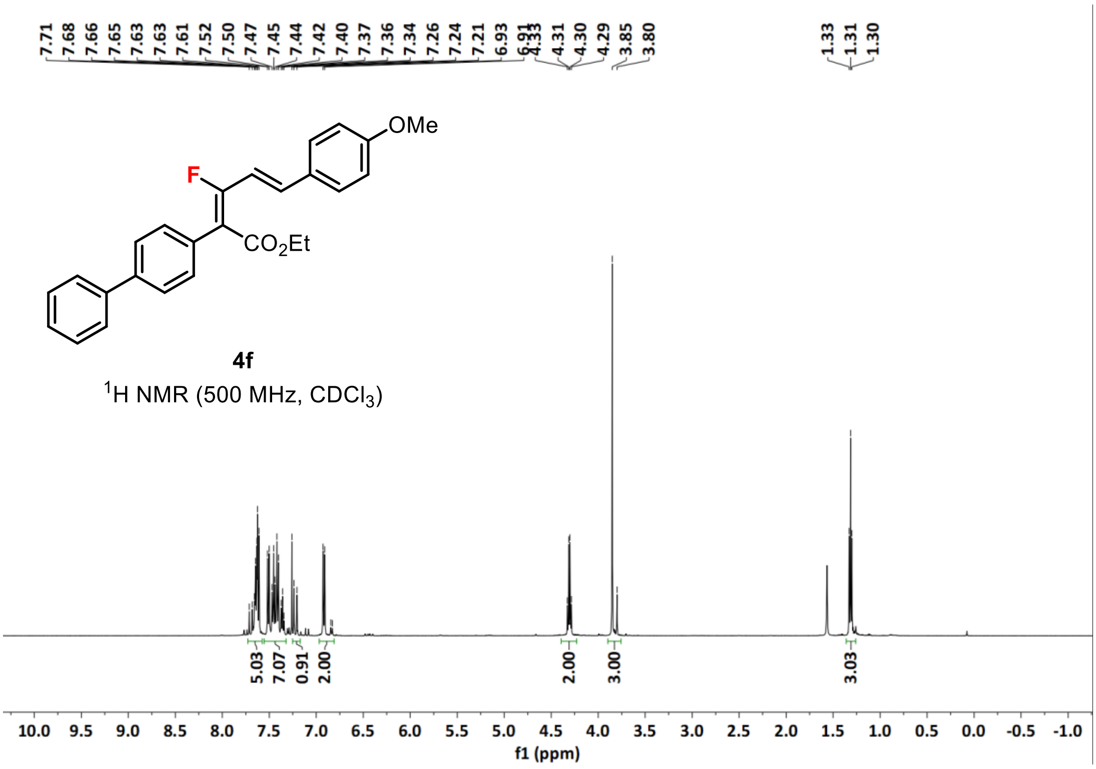

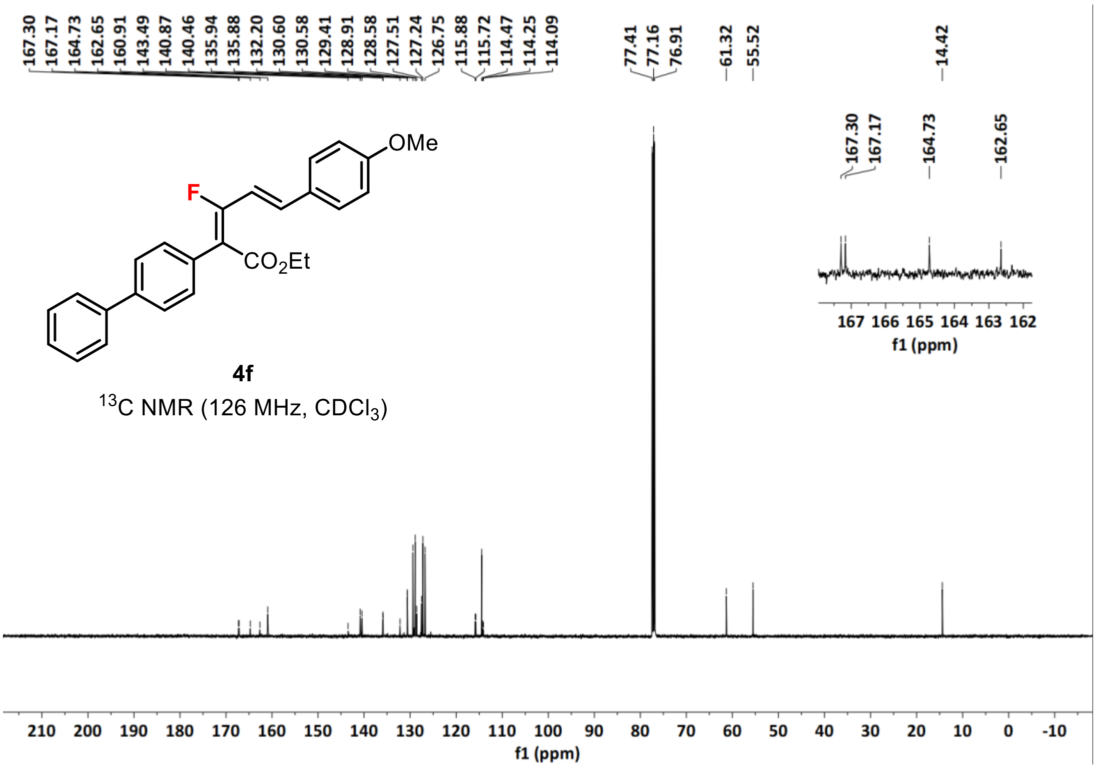

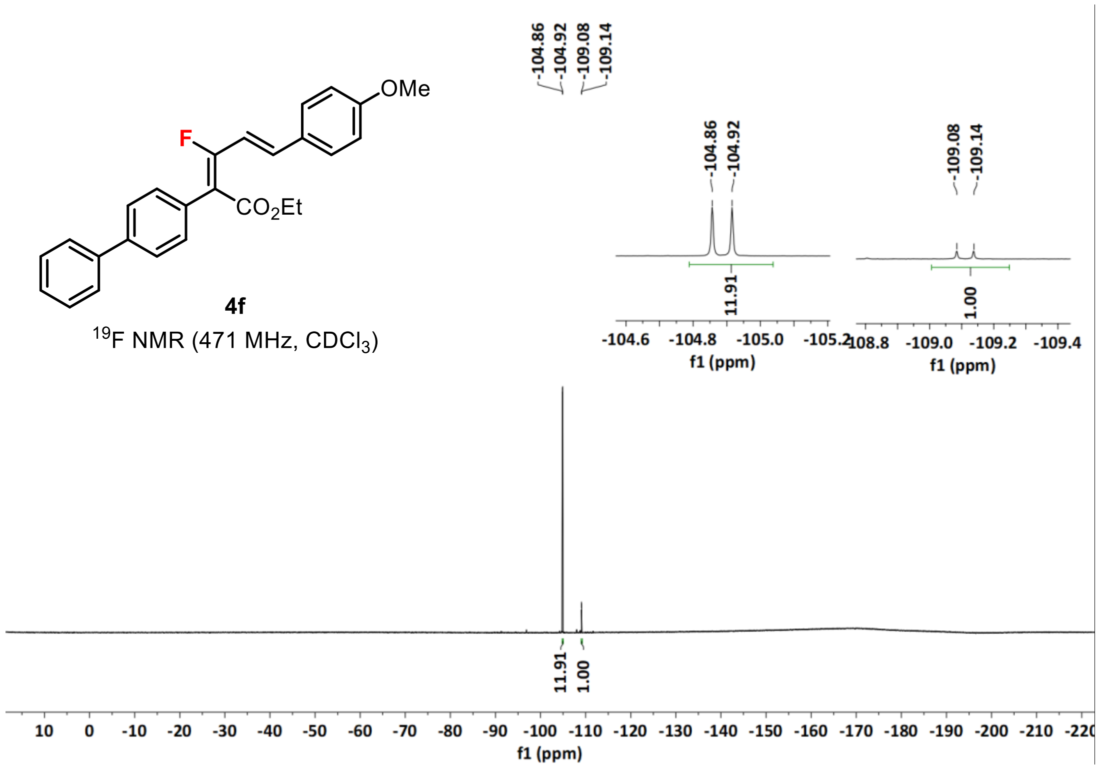

4f

^{19}F NMR (471 MHz, $CDCl_3$)

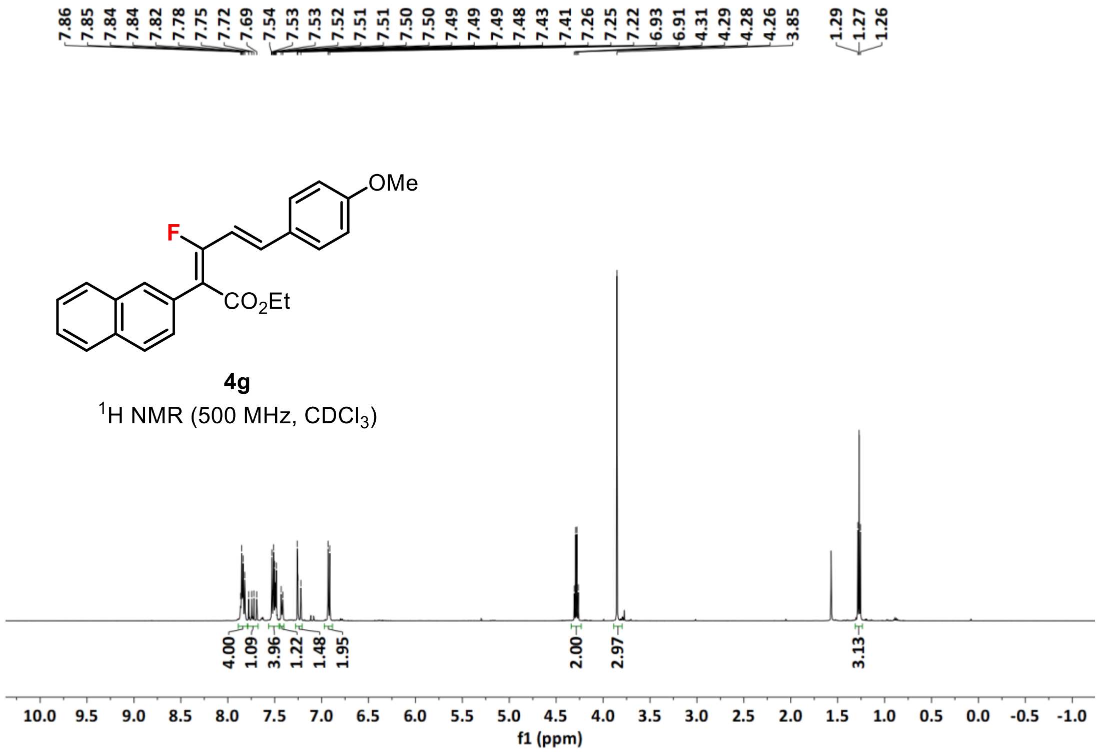

4g

^{1}H NMR (500 MHz, $CDCl_3$)

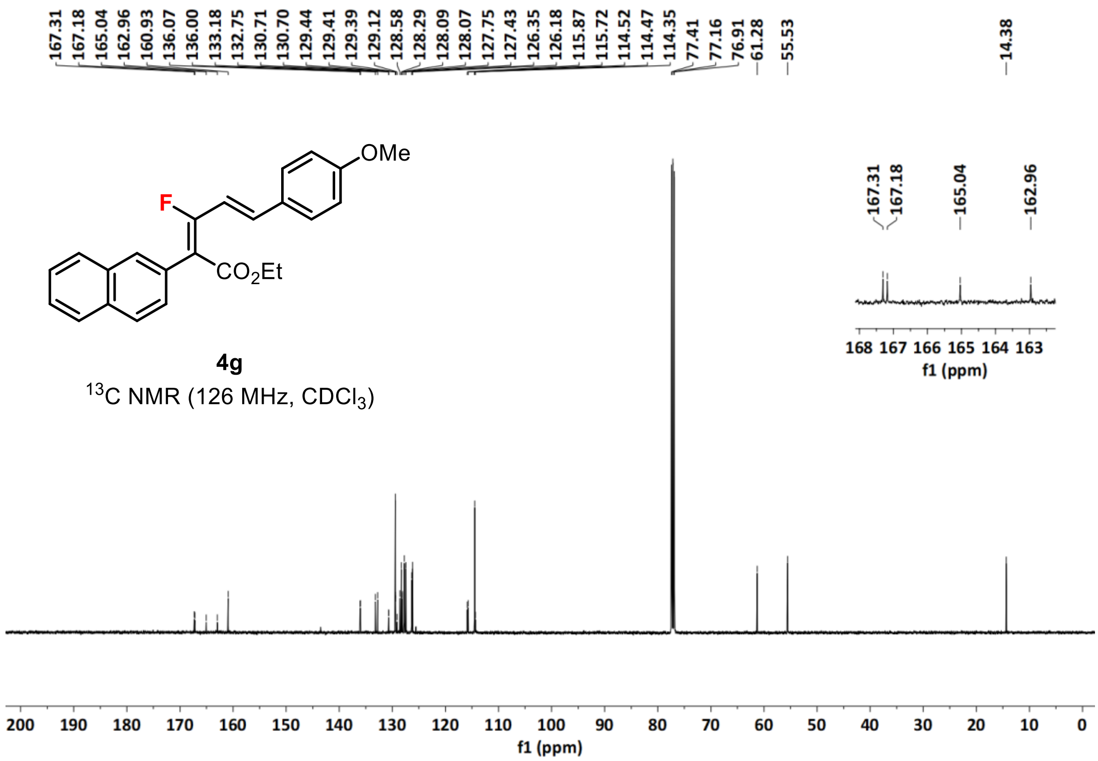
167.31
167.18
165.04
162.96
160.93
136.07
136.00
133.18
132.75
130.71
130.70
129.44
129.41
129.39
129.12
128.58
128.29
128.09
128.07
127.75
127.43
126.35
126.18
115.87
115.72
114.52
114.47
114.35
77.41
77.16
76.91
61.28
55.53
14.38
OMe
F
CO2Et
4g
13C NMR (126 MHz, CDCl3)
168 167 166 165 164 163
f1 (ppm)
200 190 180 170 160 150 140 130 120 110 100 90 80 70 60 50 40 30 20 10 0
f1 (ppm)

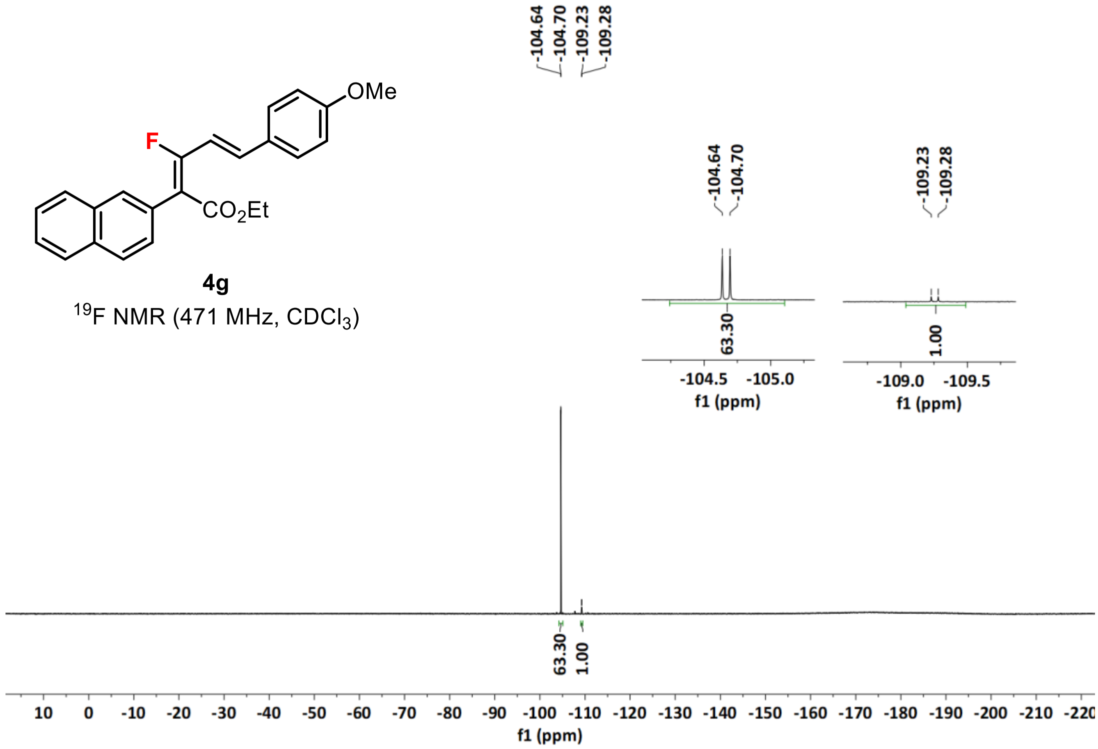
-104.64
-104.70
-109.23
-109.28
OMe
F
CO2Et
4g
19F NMR (471 MHz, CDCl3)
63.30
-104.5 -105.0
f1 (ppm)
1.00
-109.0 -109.5
f1 (ppm)
63.30
1.00
10 0 -10 -20 -30 -40 -50 -60 -70 -80 -90 -100 -110 -120 -130 -140 -150 -160 -170 -180 -190 -200 -210 -220
f1 (ppm)

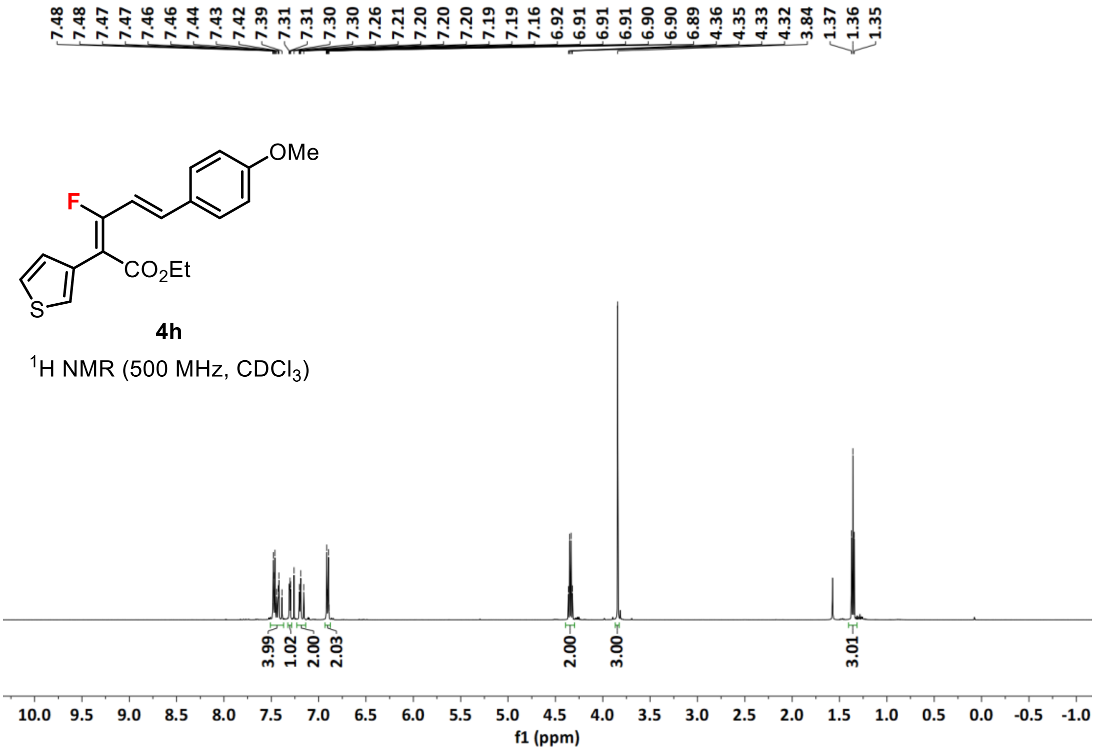

4h

^1H NMR (500 MHz, $CDCl_3$)

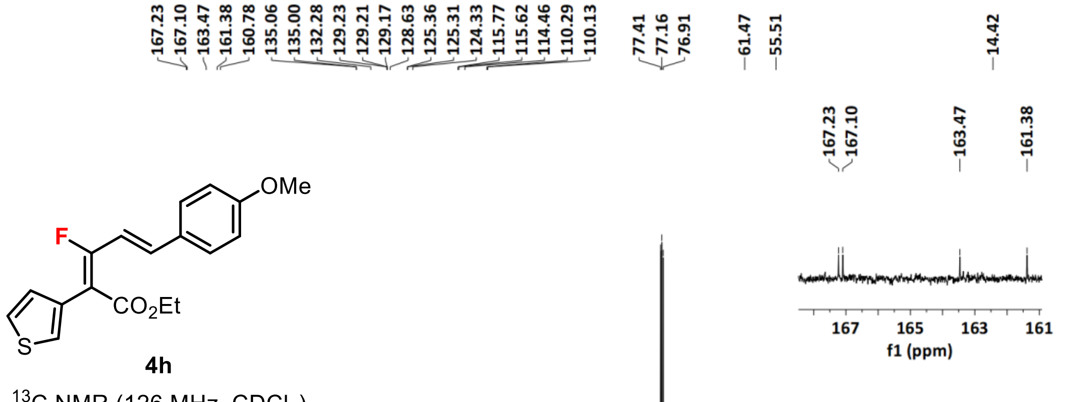

4h

^{13}C NMR (126 MHz, $CDCl_3$)

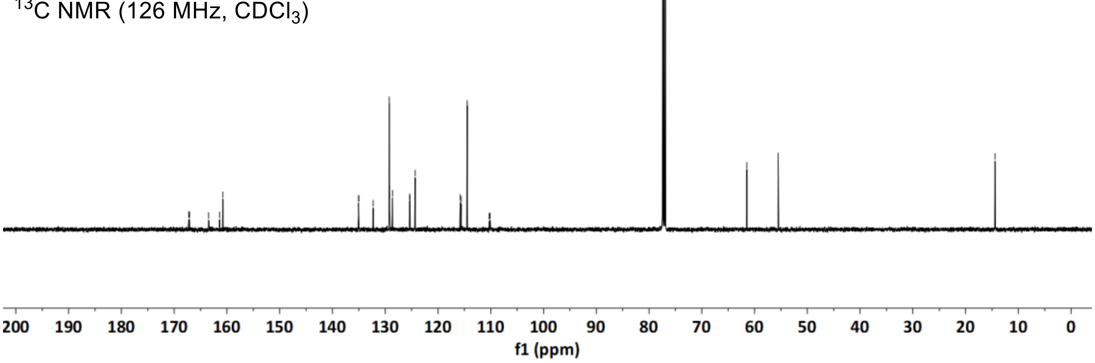

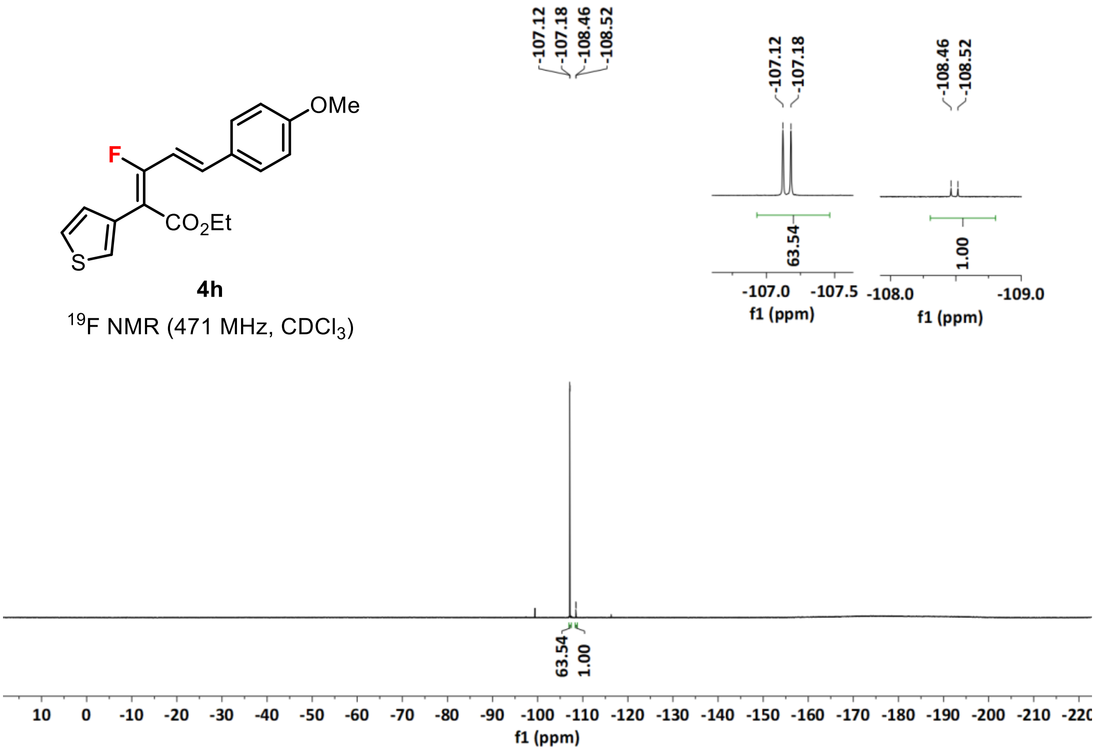

4h

^{19}F NMR (471 MHz, $CDCl_3$)

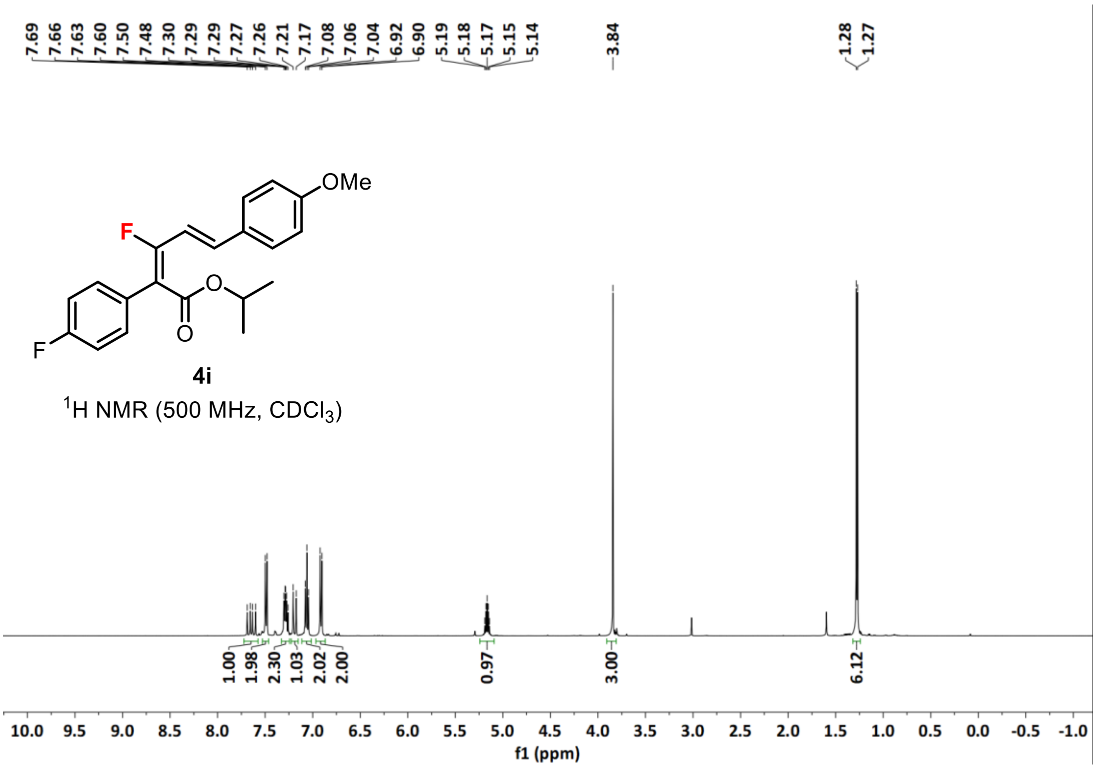

4i

^{1}H NMR (500 MHz, $CDCl_3$)

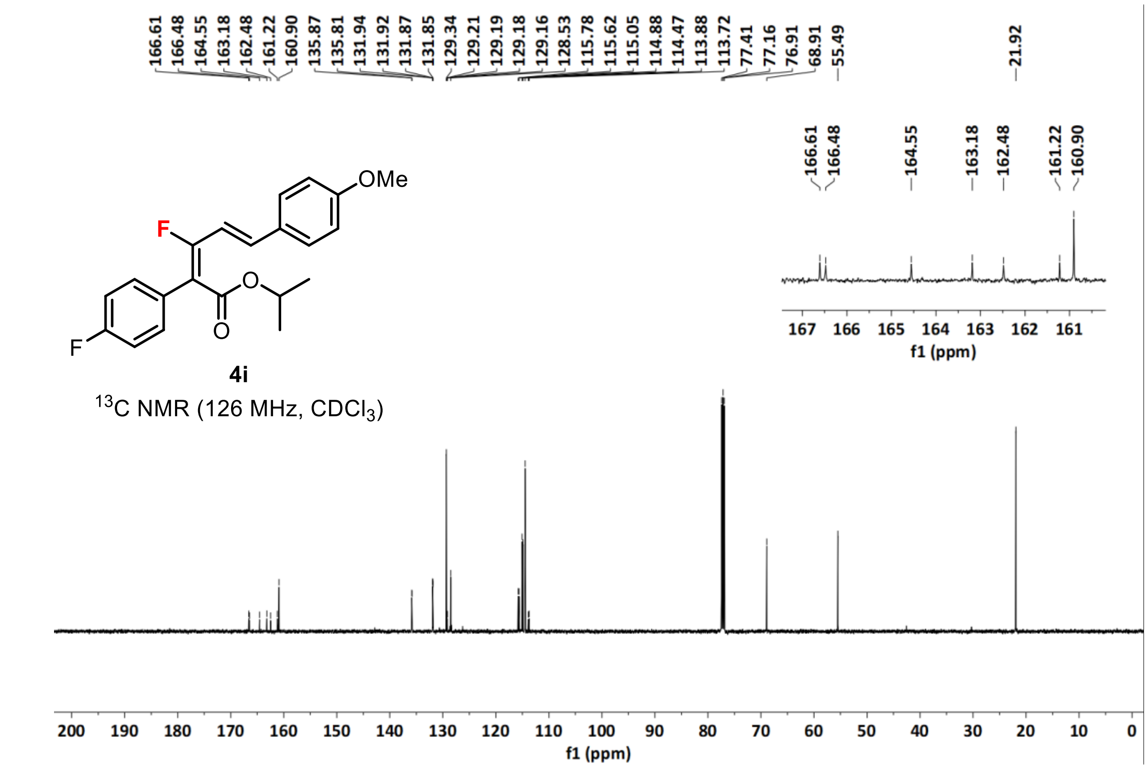
OMe
F
F
O
O
4i
^{13}C NMR (126 MHz, $CDCl_3$)
166.61 166.48 164.55 163.18 162.48 161.22 160.90 135.87 135.81 131.94 131.92 131.87 131.85 129.34 129.21 129.19 129.18 129.16 128.53 115.78 115.62 115.05 114.88 114.47 113.88 113.72 77.41 77.16 76.91 68.91 55.49 21.92
f1 (ppm)

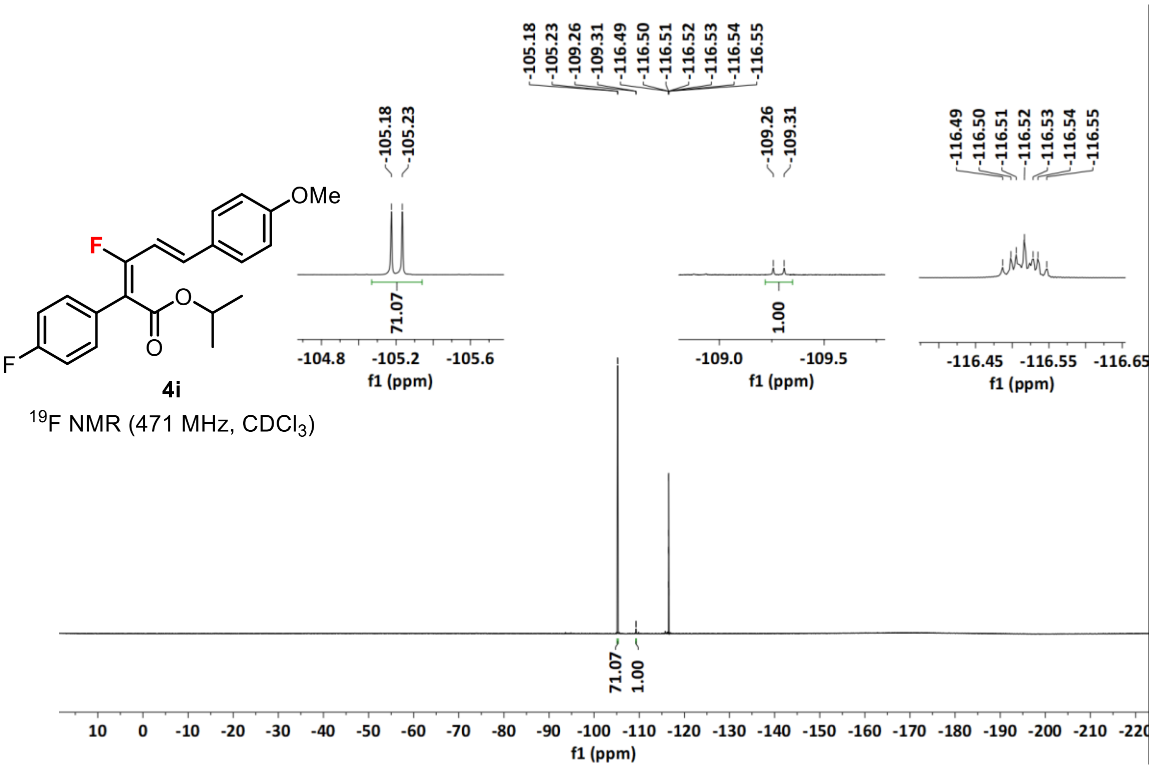
OMe
F
F
O
O
4i
^{19}F NMR (471 MHz, $CDCl_3$)
-105.18 -105.23 -109.26 -109.31 -116.49 -116.50 -116.51 -116.52 -116.53 -116.54 -116.55
71.07
1.00
f1 (ppm)

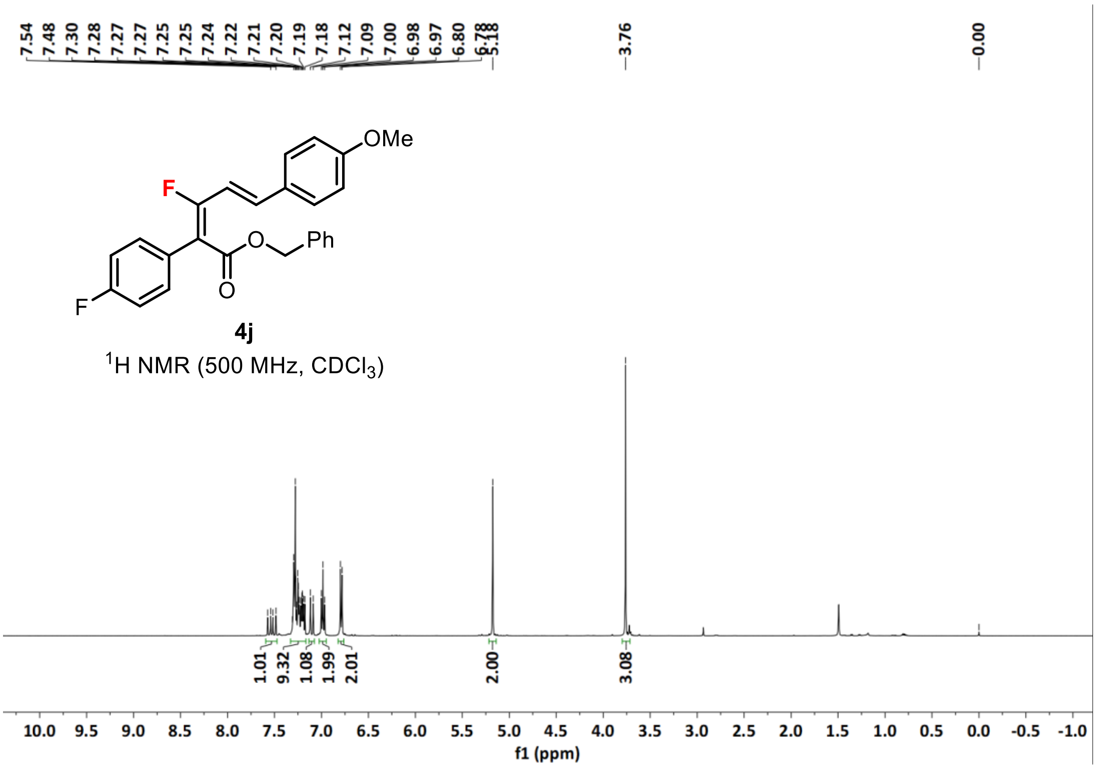
7.54
7.48
7.30
7.28
7.27
7.27
7.25
7.24
7.22
7.21
7.20
7.19
7.18
7.12
7.09
7.00
6.98
6.97
6.80
6.78
5.18
3.76
0.00
OMe
F
O
Ph
F
O
4j
1H NMR (500 MHz, CDCl3)
1.01
9.32
1.08
1.99
2.01
2.00
3.08
10.0 9.5 9.0 8.5 8.0 7.5 7.0 6.5 6.0 5.5 5.0 4.5 4.0 3.5 3.0 2.5 2.0 1.5 1.0 0.5 0.0 -0.5 -1.0
f1 (ppm)

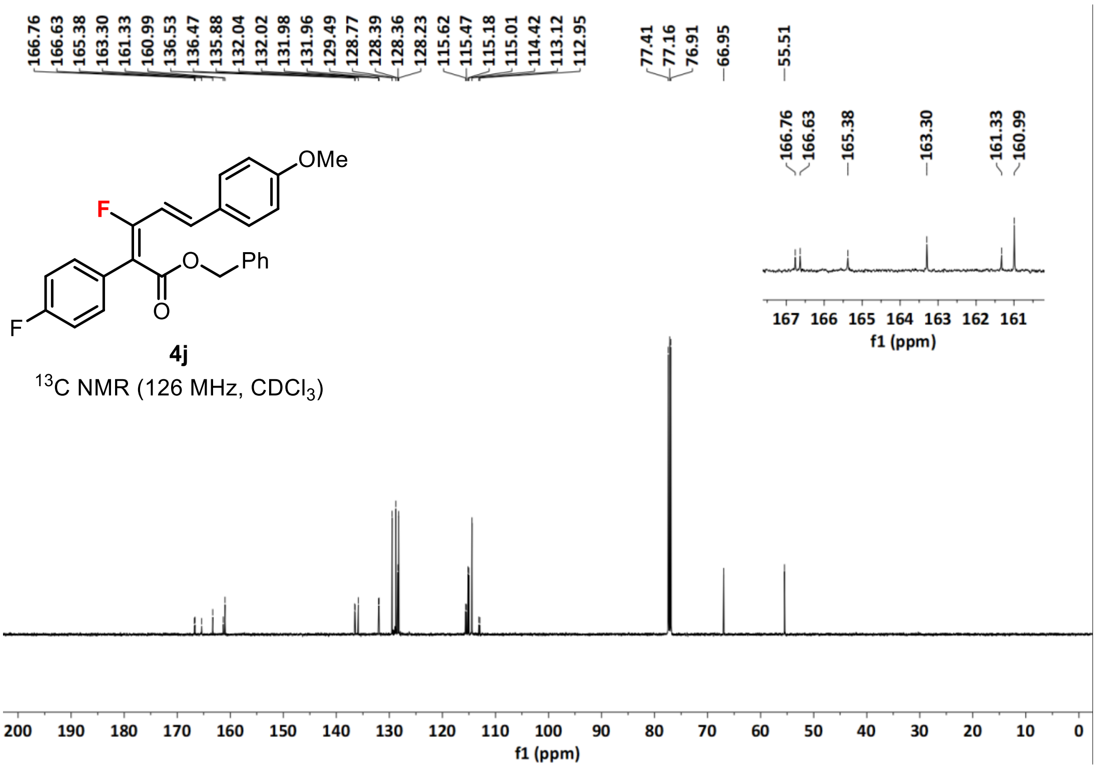
166.76
166.63
165.38
163.30
161.33
160.99
136.53
136.47
135.88
132.04
132.02
131.98
131.96
129.49
128.77
128.39
128.36
128.23
115.62
115.47
115.18
115.01
114.42
113.12
112.95
77.41
77.16
76.91
66.95
55.51
OMe
F
O
Ph
F
O
4j
13C NMR (126 MHz, CDCl3)
166.76
166.63
165.38
163.30
161.33
160.99
167 166 165 164 163 162 161
f1 (ppm)
200 190 180 170 160 150 140 130 120 110 100 90 80 70 60 50 40 30 20 10 0
f1 (ppm)

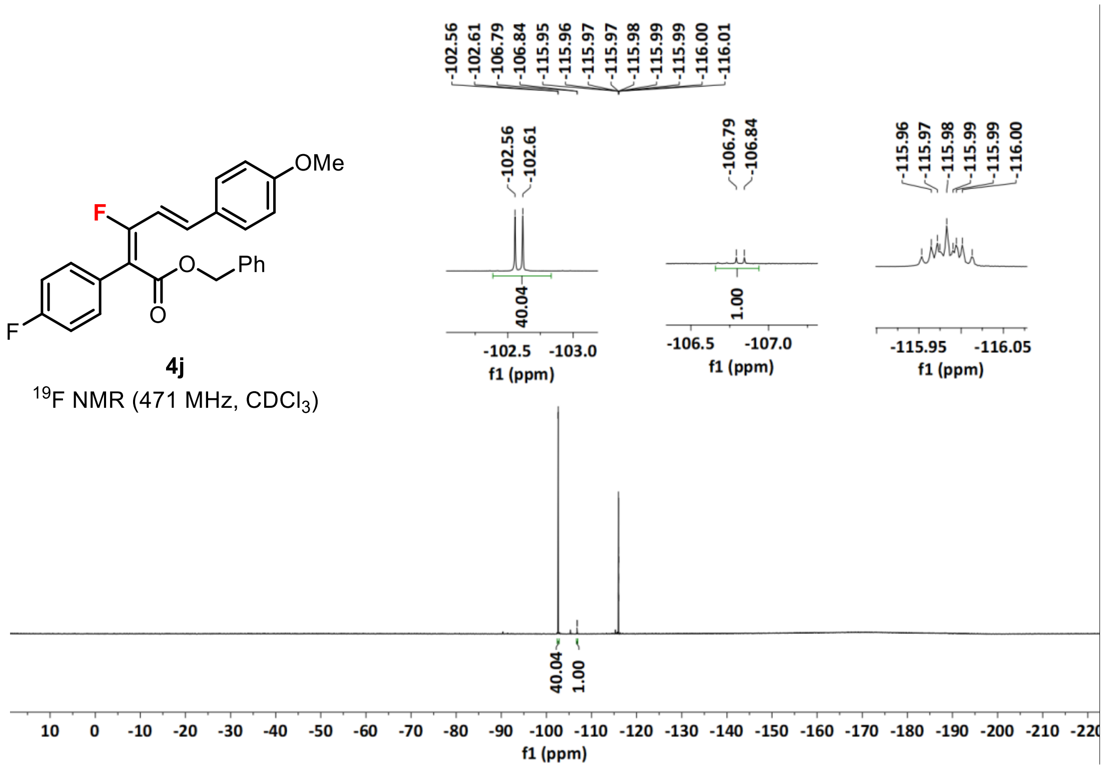

4j

^{19}F NMR (471 MHz, $CDCl_3$)

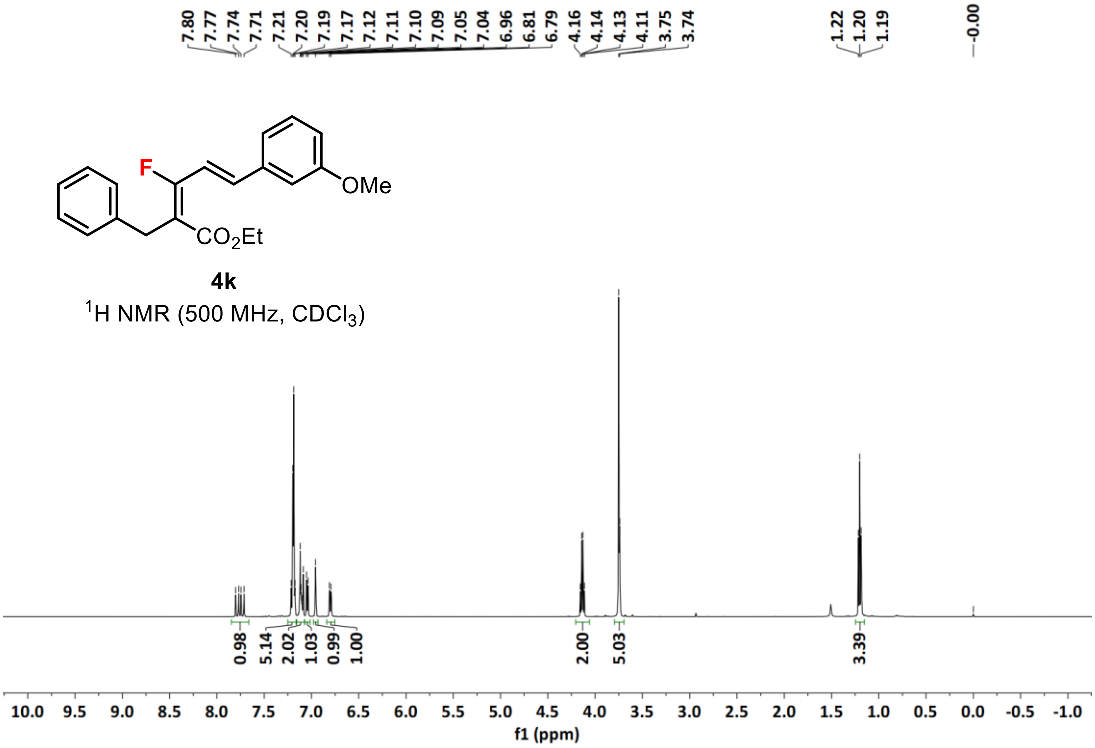

4k

^{1}H NMR (500 MHz, $CDCl_3$)

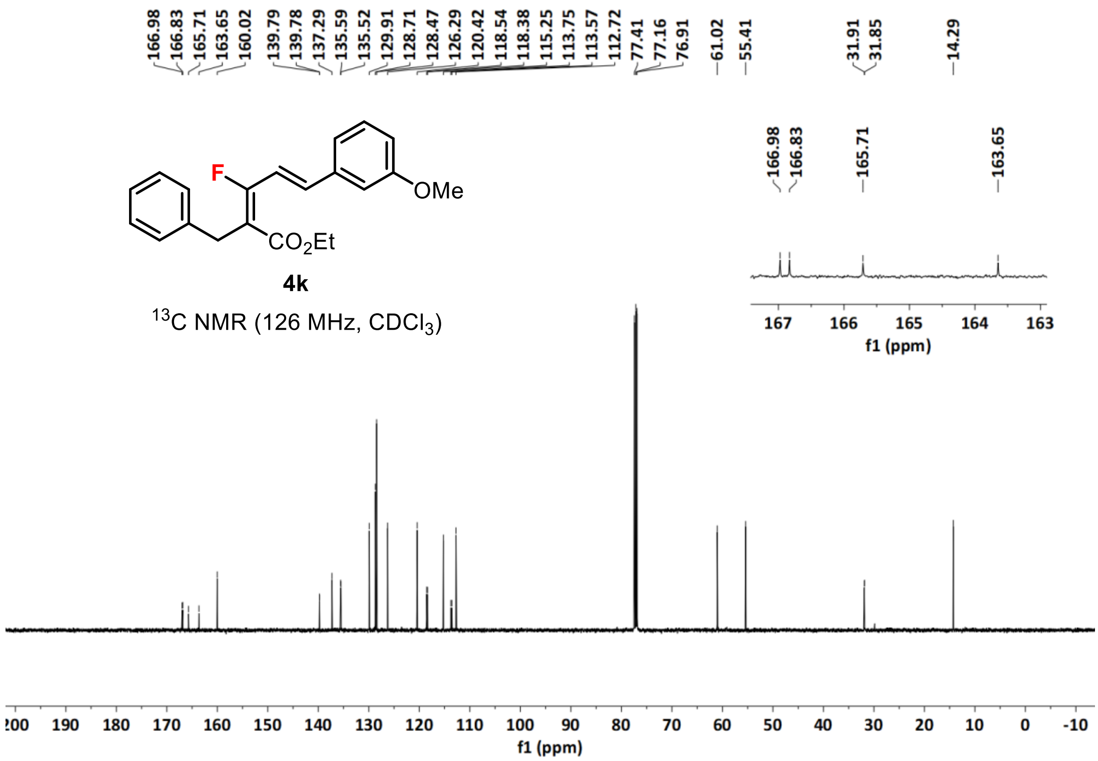
166.98
166.83
165.71
163.65
160.02
139.79
139.78
137.29
135.59
135.52
129.91
128.71
128.47
126.29
120.42
118.54
118.38
115.25
113.75
113.57
112.72
77.41
77.16
76.91
61.02
55.41
31.91
31.85
14.29
F
OMe
CO_2Et
4k
^{13}C NMR (126 MHz, $CDCl_3$)
f1 (ppm)

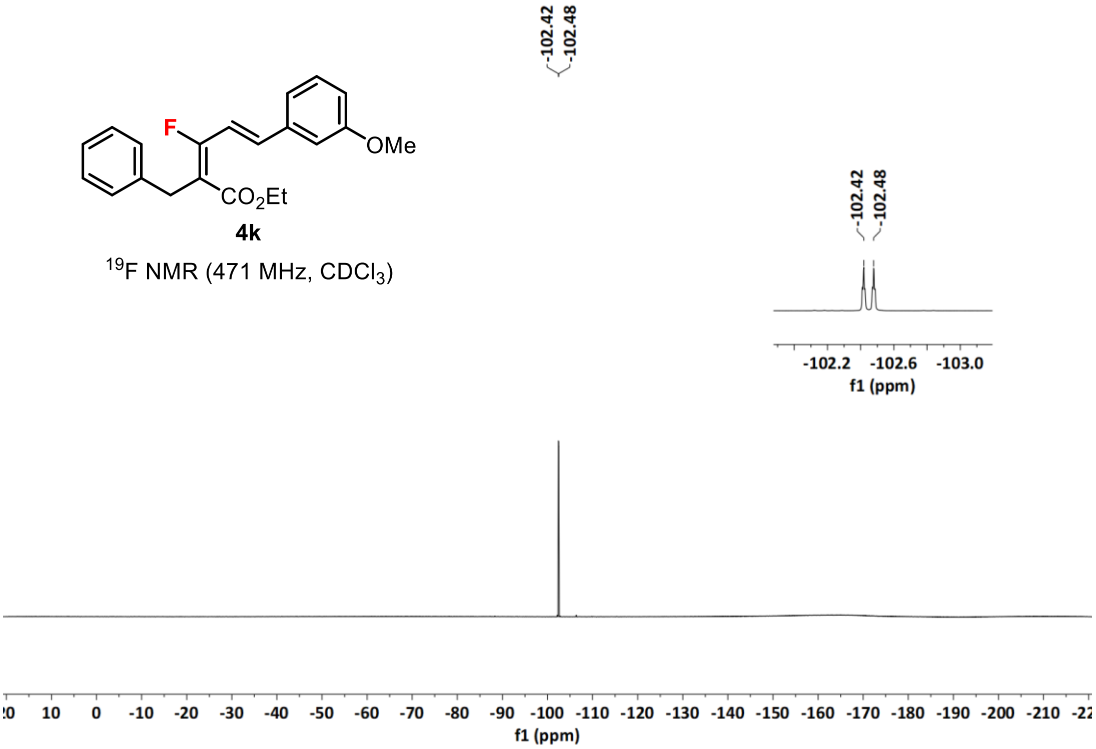
-102.42
-102.48
F
OMe
CO_2Et
4k
^{19}F NMR (471 MHz, $CDCl_3$)
f1 (ppm)

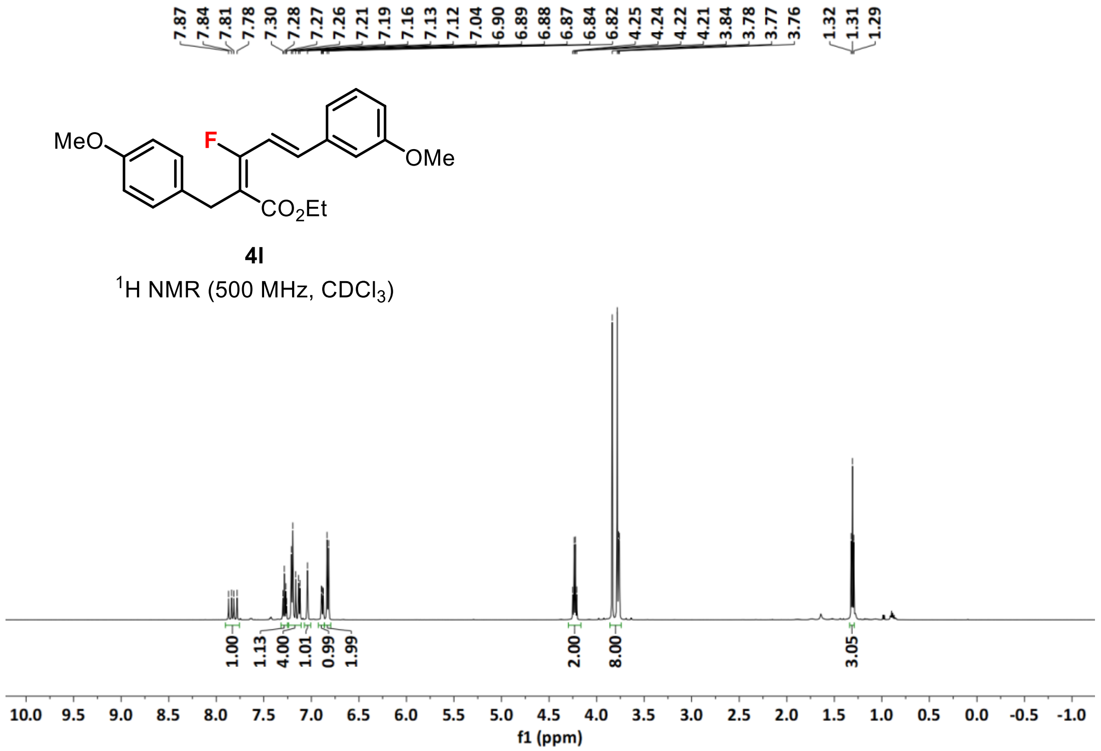

^{1}H NMR (500 MHz, $CDCl_3$)

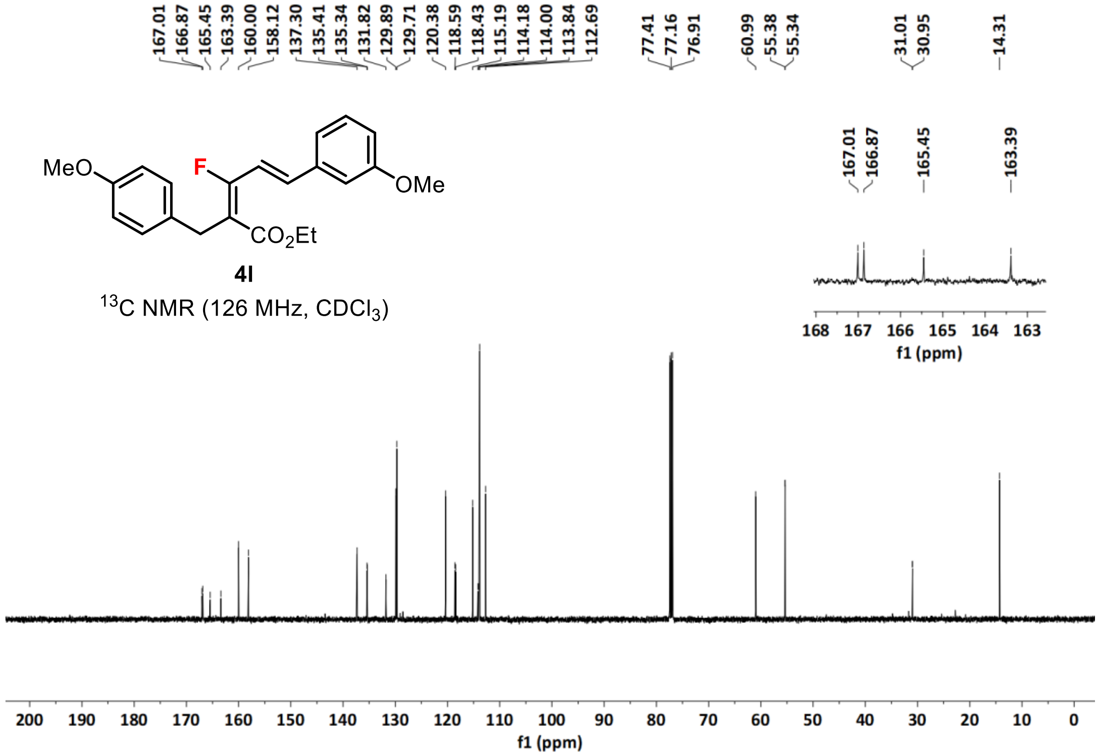

^{13}C NMR (126 MHz, $CDCl_3$)

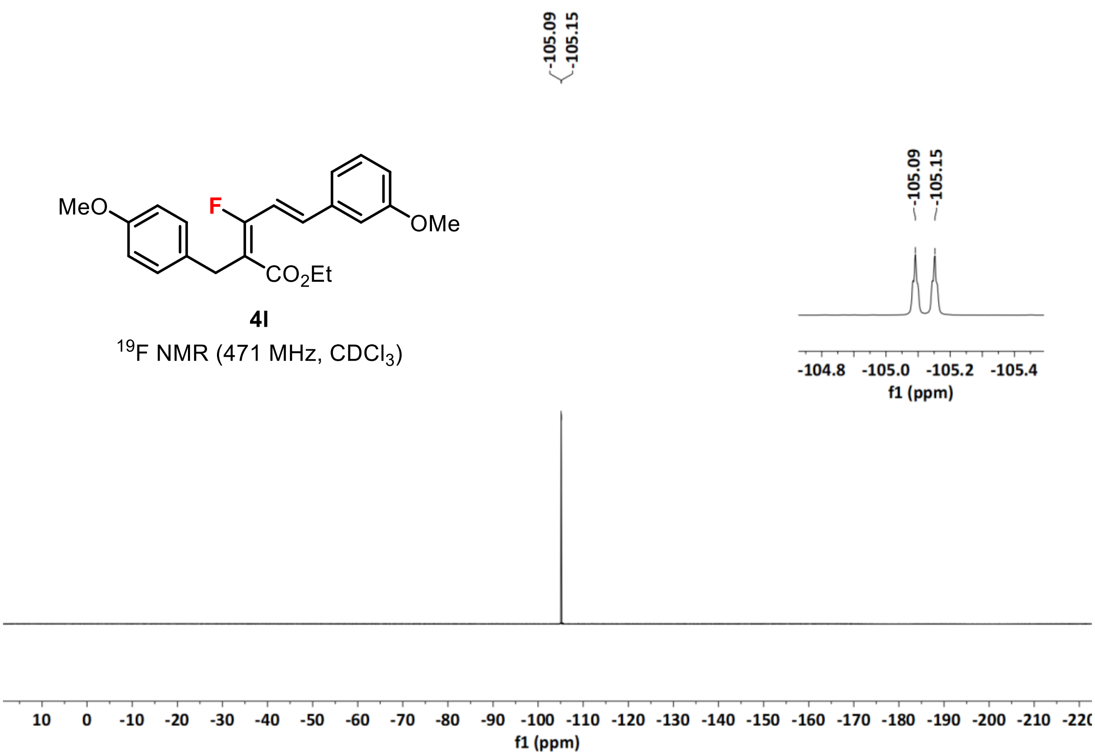

4l

^{19}F NMR (471 MHz, $CDCl_3$)

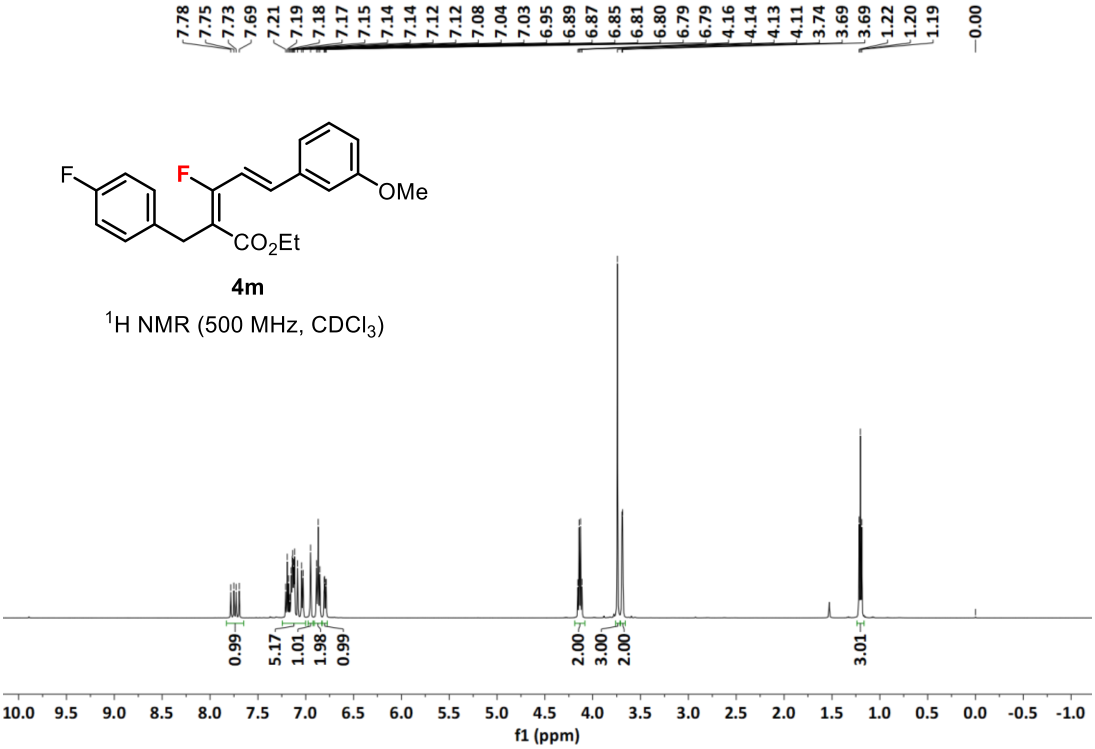

4m

^{1}H NMR (500 MHz, $CDCl_3$)

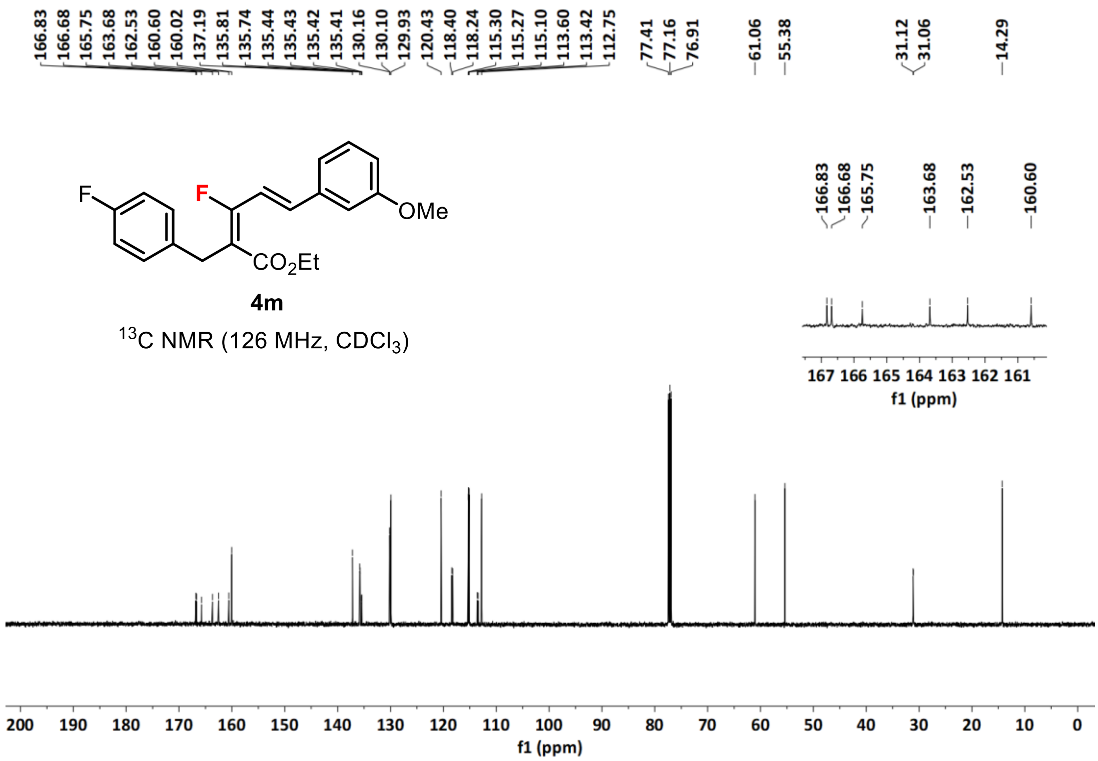

166.83
166.68
165.75
163.68
162.53
160.60
160.02
137.19
135.81
135.74
135.44
135.43
135.42
135.41
130.16
130.10
129.93
120.43
118.40
118.24
115.30
115.27
115.10
113.60
113.42
112.75
77.41
77.16
76.91
61.06
55.38
31.12
31.06
14.29
F
F
OMe
CO_2Et
4m
^{13}C NMR (126 MHz, $CDCl_3$)
166.83
166.68
165.75
163.68
162.53
160.60
167 166 165 164 163 162 161
f1 (ppm)
200 190 180 170 160 150 140 130 120 110 100 90 80 70 60 50 40 30 20 10 0
f1 (ppm)

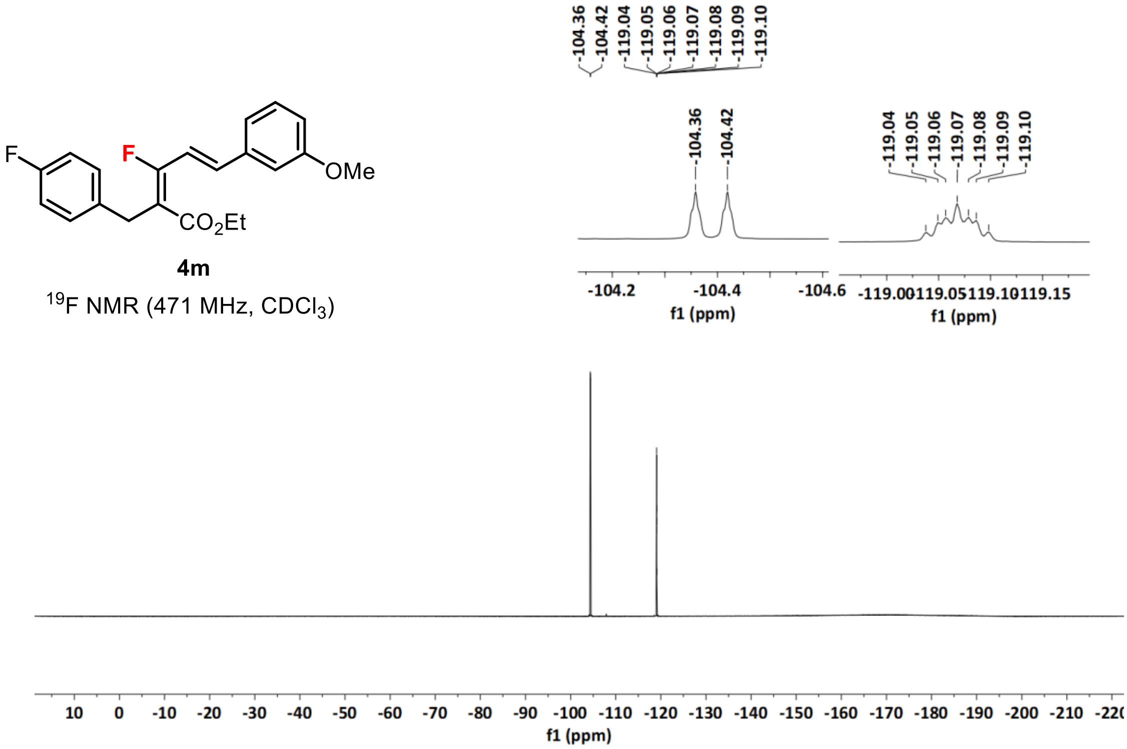

-104.36
-104.42
-119.04
-119.05
-119.06
-119.07
-119.08
-119.09
-119.10
F
F
OMe
CO_2Et
4m
^{19}F NMR (471 MHz, $CDCl_3$)
-104.36
-104.42
-119.04
-119.05
-119.06
-119.07
-119.08
-119.09
-119.10
-104.2 -104.4 -104.6
f1 (ppm)
-119.00119.05119.10119.15
f1 (ppm)
10 0 -10 -20 -30 -40 -50 -60 -70 -80 -90 -100 -110 -120 -130 -140 -150 -160 -170 -180 -190 -200 -210 -220
f1 (ppm)

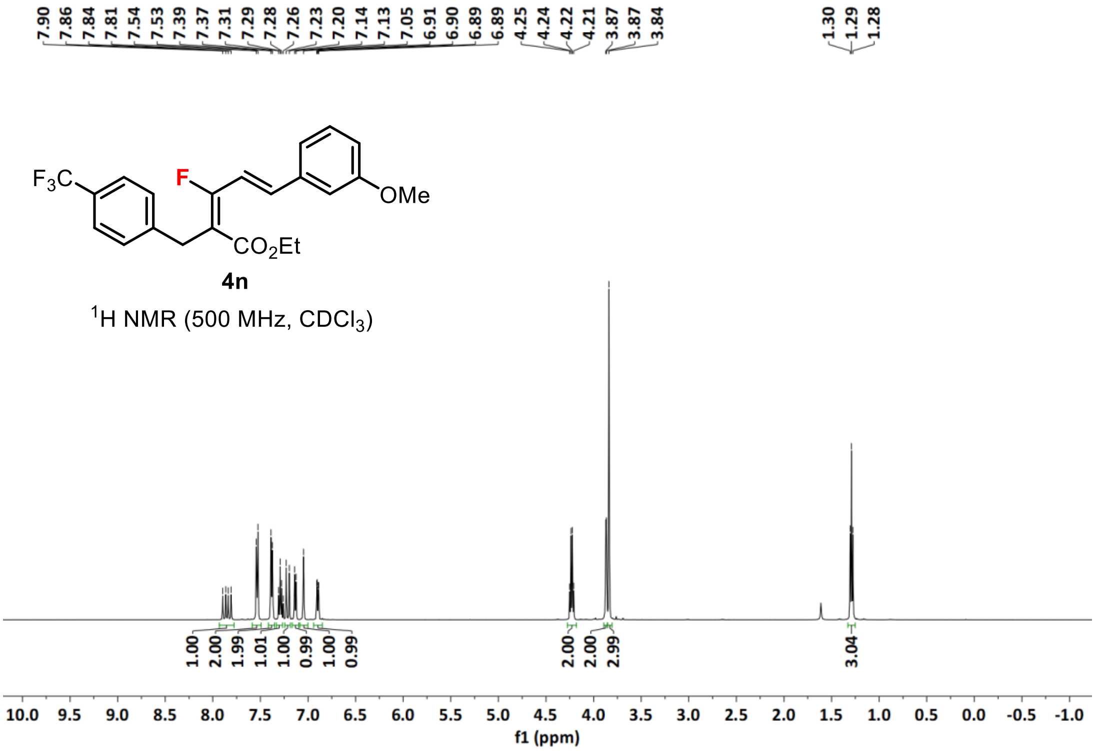
7.90 7.86 7.84 7.81 7.54 7.53 7.39 7.37 7.31 7.29 7.28 7.26 7.23 7.20 7.14 7.13 7.05 6.91 6.90 6.89 6.89 4.25 4.24 4.22 4.21 3.87 3.87 3.84 1.30 1.29 1.28
F₃C
F
OMe
CO₂Et
4n
¹H NMR (500 MHz, CDCl₃)
1.00 2.00 1.99 1.01 1.00 0.99 1.00 0.99 2.00 2.00 2.99 3.04
f1 (ppm)

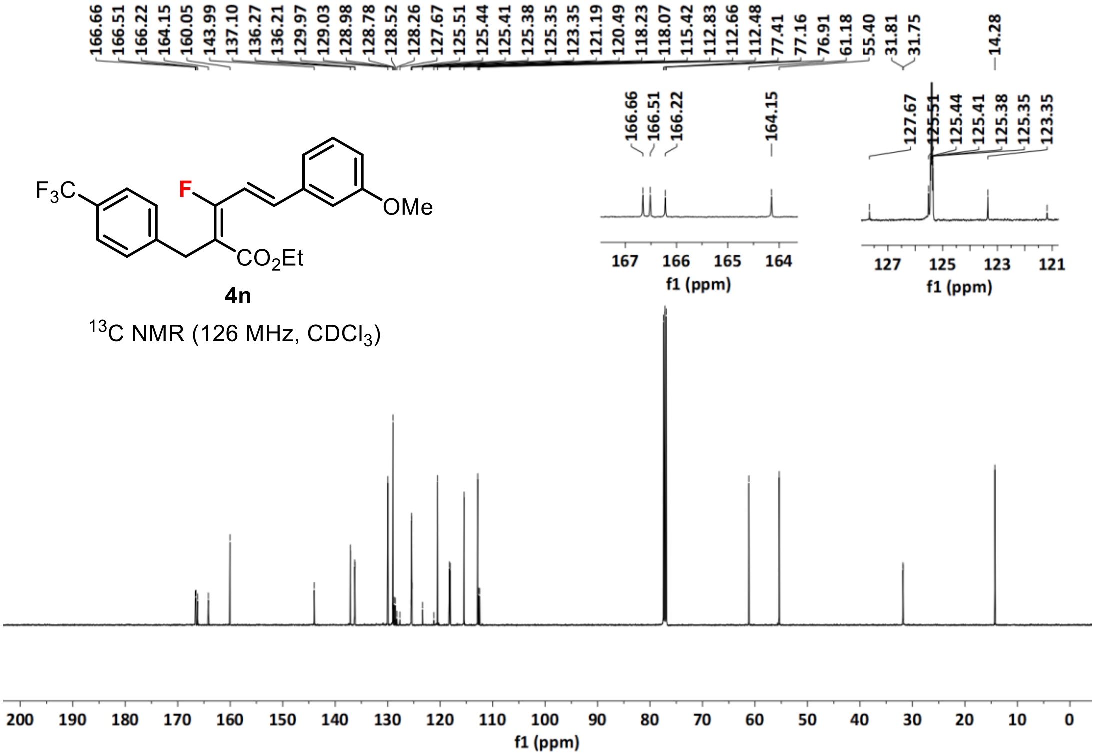
166.66 166.51 166.22 164.15 160.05 143.99 137.10 136.27 136.21 129.97 129.03 128.98 128.78 128.52 128.26 127.67 125.51 125.44 125.41 125.38 125.35 123.35 121.19 120.49 118.23 118.07 115.42 112.83 112.66 112.48 77.41 77.16 76.91 61.18 55.40 31.81 31.75 14.28
166.66 166.51 166.22 164.15
127.67 125.51 125.44 125.41 125.38 125.35 123.35
F₃C
F
OMe
CO₂Et
4n
¹³C NMR (126 MHz, CDCl₃)
f1 (ppm)

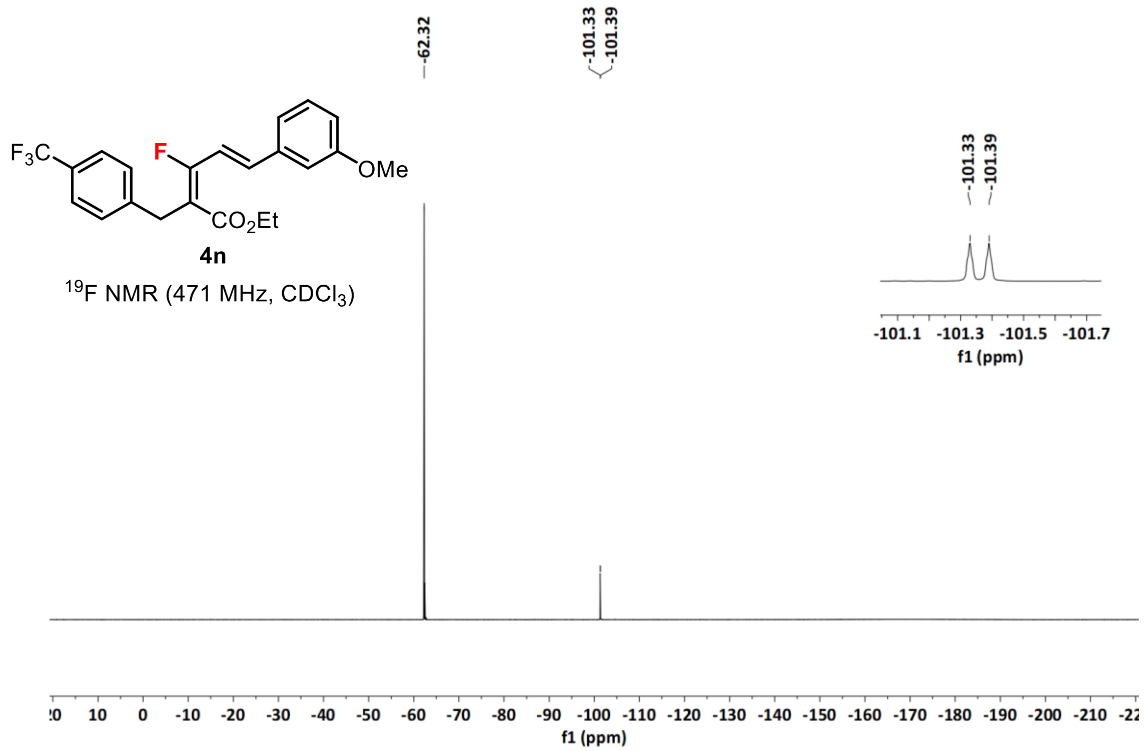

4n

^{19}F NMR (471 MHz, $CDCl_3$)

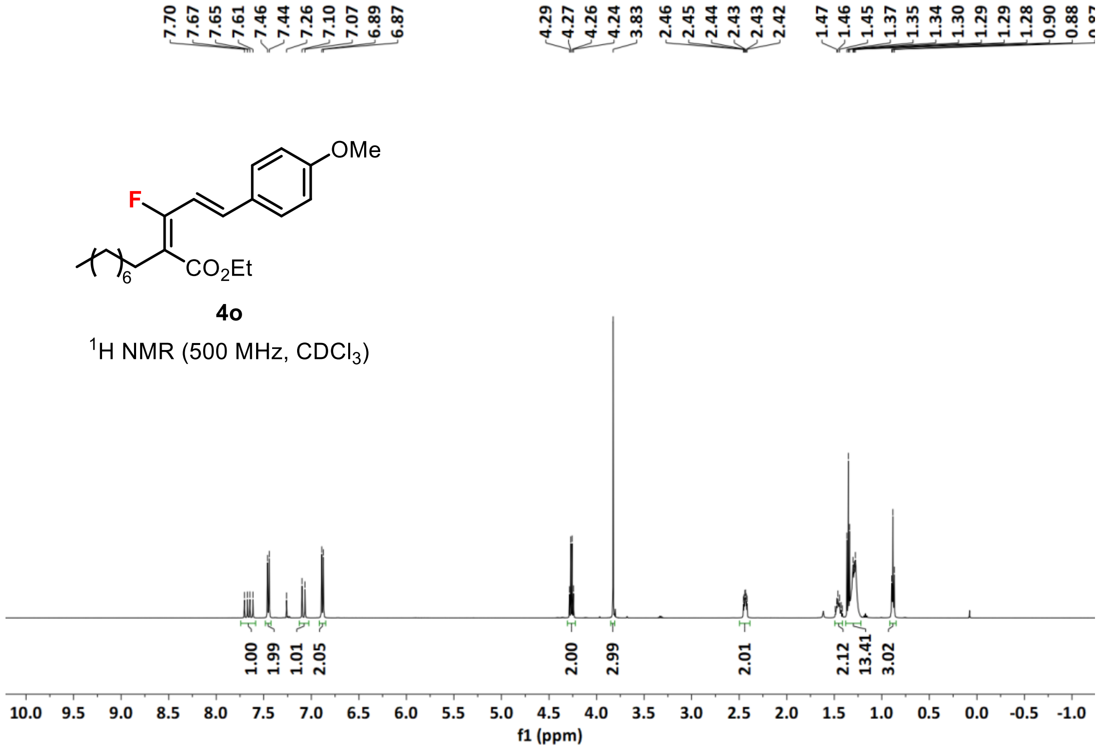

4o

^{1}H NMR (500 MHz, $CDCl_3$)

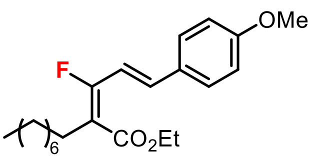

4o

^{13}C NMR (126 MHz, $CDCl_3$)

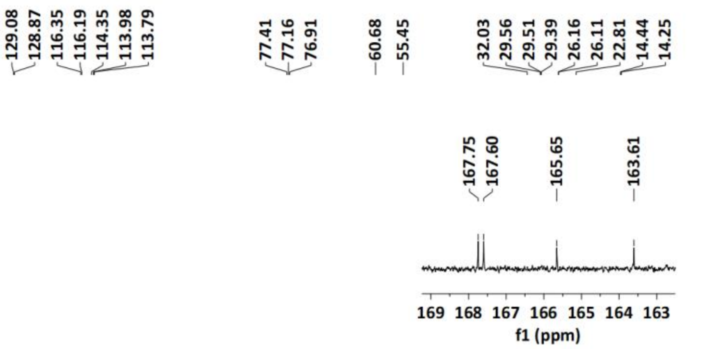

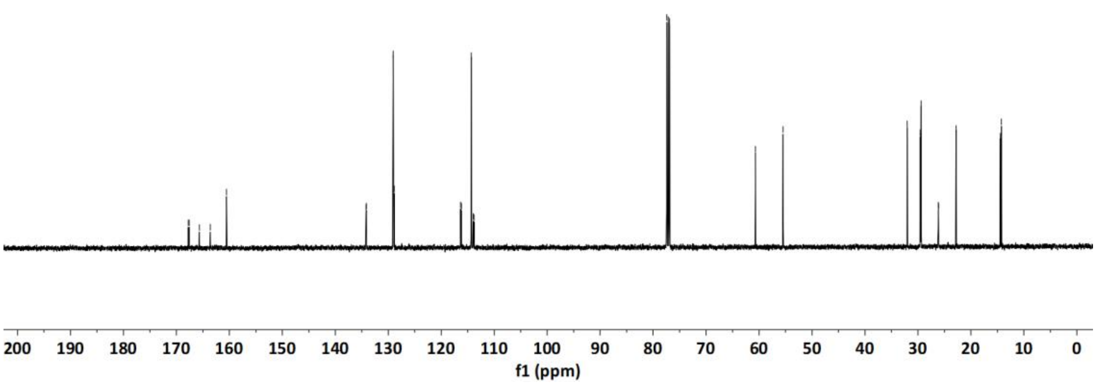

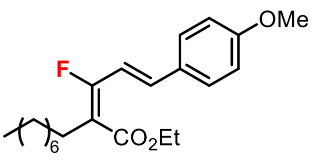

4o

^{19}F NMR (471 MHz, $CDCl_3$)

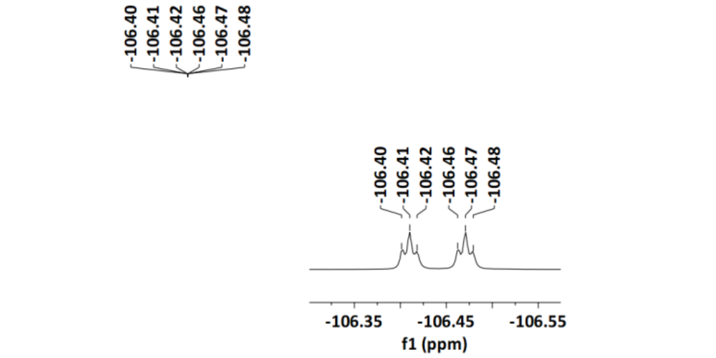

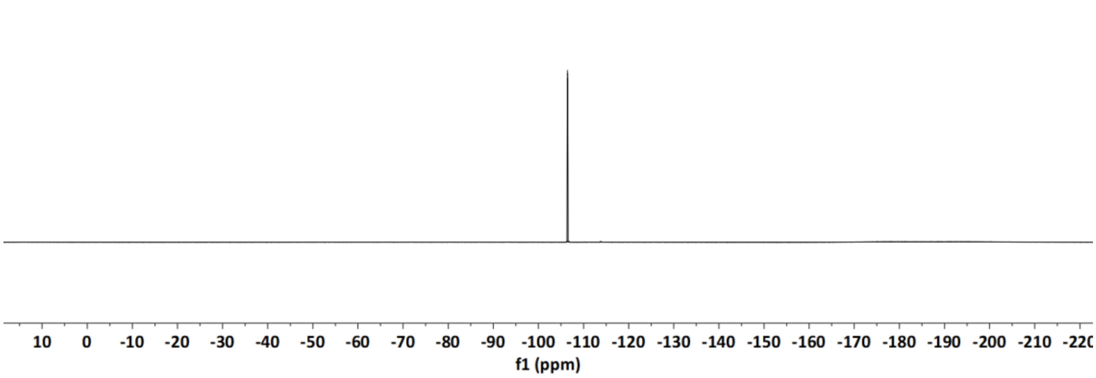

Supplement: Supplementary file 1 — ol3c02452_si_001.pdf [file ol3c02452_si_001.pdf]
